# Population Genomics of an Obligately Halophilic Basidiomycete *Wallemia ichthyophaga*

**DOI:** 10.3389/fmicb.2019.02019

**Published:** 2019-09-04

**Authors:** Cene Gostinčar, Xiaohuan Sun, Janja Zajc, Chao Fang, Yong Hou, Yonglun Luo, Nina Gunde-Cimerman, Zewei Song

**Affiliations:** ^1^Department of Biology, Biotechnical Faculty, University of Ljubljana, Ljubljana, Slovenia; ^2^Lars Bolund Institute of Regenerative Medicine, BGI-Qingdao, Qingdao, China; ^3^BGI-Shenzhen, Shenzhen, China; ^4^China National GeneBank, BGI-Shenzhen, Shenzhen, China; ^5^National Institute of Biology, Ljubljana, Slovenia; ^6^Department of Biomedicine, Aarhus University, Aarhus, Denmark

**Keywords:** population genomics, halotolerance, halophilic fungus, basidiomycete, specialist, recombination

## Abstract

**Background:**

*Wallemia ichthyophaga* is a highly specialized basidiomycetous fungus. It is one of the most halophilic fungi ever described, only able to grow at low water activity. This specialization is thought to explain why it is only rarely isolated from nature.

**Results:**

Genomes of 21 *W. ichthyophaga* strains were sequenced with PE150 reads on BGISEQ500 platform. The genomes shared high similarity with the reference genome of the species, they were all smaller than 10 Mbp and had a low number of predicted genes. Groups of strains isolated in the same location encompassed clones as well as very divergent strains. There was little concordance between phylogenies of predicted genes. Linkage disequilibrium of pairs of polymorphic loci decayed relatively quickly as a function of distance between the loci (LD decay distance 1270 bp). For the first time a putative mating-type locus was identified in the genomes of *W. ichthyophaga*.

**Conclusion:**

Based on the comparison of *W. ichthyophaga* genomes it appears that some phylogenetic lineages of the species can persist in the same location over at least several years. Apart from this, the differences between the strains do not reflect the isolation habitat or geographic location. Together with results supporting the existence of (sexual) recombination in *W. ichthyophaga*, the presented results indicate that strains of *W. ichthyophaga* can form a single recombining population even between different habitats and over large geographical distances.

## Introduction

Microbiological research of hypersaline environments has traditionally focused on prokaryotes and the alga *Dunaliella salina* (Zajc et al., 2017). Although halotolerant fungi were long known as contaminants of salted food (Pitt and Hocking, 1977), their presence in nature was not systematically investigated until the end of the 20^th^ century. In the last two decades it has become clear that certain fungal species form an integral part of microbial communities in different hypersaline environments around the world (Zajc et al., 2017). Even though these species grow at salinities exceeding 2.9M NaCl, most of them also grow in normal microbiological media with no added salt (i.e., they are halotolerant and not halophilic), unlike many prokaryotes from hypersaline environments, which are typically halophilic (Gostinčar et al., 2015). One of only a handful of true fungal halophiles is *Wallemia ichthyophaga*, one of the most extremotolerant fungal species with regard to low water activity and especially high concentrations of NaCl and other salts.

*Wallemia ichthyophaga* has been described in 2005 as part of a taxonomic revision of the *Wallemia sebi* species complex (Zalar et al., 2005). In addition to being phylogenetically and morphologically distinct from other species of the genus, the new species *W. ichthyophaga* differed from *W. sebi* in its requirement for low water activity, being unable to grow without the addition of solutes to the medium, preferably NaCl, with the lower growth limit at around 1.5M NaCl (Zalar et al., 2005; Kralj Kunčič et al., 2010) and optimum between 2.6 and 3.4M NaCl (Zajc et al., 2014b). *W. ichthyophaga* is one of the rare fungal species that strongly prefer high concentrations of ionic (e.g., salt) over non-ionic (e.g., sugar) solutes (Zajc et al., 2014b). In addition to this, it is able to grow in saturated NaCl, KCl, and MgSO_4_ solutions and even at around 2M MgCl_2_ (Zajc et al., 2014a), explaining its isolation from bittern, residual magnesium rich water left after commercial harvesting of the (predominantly NaCl) salt from the evaporation of sea water (Jančič et al., 2016).

Ecology of *W. ichthyophaga* is not well-understood due to only sporadic isolation from nature. A small number of strains have been isolated from sea water concentrated by evaporation in man-made outdoor pools for the purpose of harvesting salt and related habitats, and from salted food (Jančič et al., 2015). A second group of strains have been isolated from the air in two barns with horses and hay. Barns were identified as a rich source of other *Wallemia* spp. (Jančič et al., 2016), but the recovery of *W. ichthyophaga* from this environment was unexpected and occurred at only two of several sampled locations.

The small number of known isolates can partially be ascribed to their slow growth and requirement for media with very low water activity. Growth media with extremely high salinities (such as 15 to 20% NaCl) and prolonged incubation (at least 14 days) have been recommended for the successful isolation of *W. ichthyophaga* strains from nature (Zajc et al., 2014b). However, even a focused sampling of more than 300 low-water-activity substrates and using appropriate selective media yielded only a handful of new isolates (Jančič et al., 2016). It has thus been suggested that the rarity of isolation of *W. ichthyophaga* may simply reflect its low abundance in nature, which in turn is a consequence of the extreme specialization and low adaptability of the species (Gostinčar et al., 2010).

Genome sequencing of *W. ichthyophaga* in 2013 uncovered an unusually compact genome (9.6 Mbp) with a low number of predicted protein coding genes (4884) and high gene density – the genes occupied almost three quarters of the genome. The average genome size in Basidiomycota is 46.48 Mbp and an average genome contains more than 15000 genes (Mohanta and Bae, 2015). No discernible mating type locus was identified and only three out of eight homologs of meiosis-specific genes were found in *W. ichthyophaga* (Zajc et al., 2013). This seemed to be in agreement with previous studies, which failed to observe any kind of sexual reproduction in the species (Zalar et al., 2005).

To investigate the population characteristics of *W. ichthyophaga*, genomes of 22 strains of the species were sequenced. A strain of *Wallemia hederae*, a sister species of *W. ichthyophaga* (Jančič et al., 2016), was added to the dataset as an outgroup.

## Results

Twenty-two genomes of the obligately halophilic basidiomycete *W. ichthyophaga* and one genome of the closely related *W. hederae* have been sequenced and analyzed to investigate the population structure of the species and to facilitate future studies of its extremophilic physiology. Most of the currently known strains of *W. ichthyophaga* were sequenced (Table 1), including isolates from sea-water-related hypersaline environments (5 strains from ponds of concentrated sea water used for salt production, 5 strains from bittern, magnesium rich water left after harvesting the other salts in salt production, 1 strain from a crystal of sea salt), from air (9 strains from air in horse and hay barns), and from low activity food (2 strains). With the exception of one strain from Namibia and one from Lofoten Islands, Norway, all the rest originated from either Slovenia or Denmark. The sequences of genome 11 (EXF-8617) were found to contain (possibly contaminant) sequences with higher similarity to *W. mellicola* than *W. ichthyophaga*, therefore this genome was excluded from all downstream analyses.

**TABLE 1:** Strains sequenced in this study.

**Culture collection strain number**	**Number in this study**	**Isolation habitat**	**Sampling site location**
EXF-759 (CBS 116630)	1	Saltern: hypersaline water of saltern	Namibia
EXF-3555	2	Food: salted ham	Slovenia; Ljubljana
EXF-5676	3	Saltern: sea salt crystal	Slovenia; Sečovlje
EXF-6065	4	Saltern: hypersaline saltern water	Slovenia; Sečovlje
EXF-6066	5	Saltern: hypersaline saltern water	Slovenia; Sečovlje
EXF-6067	6	Saltern: hypersaline saltern water	Slovenia; Sečovlje
EXF-6068	7	Saltern: bittern water	Slovenia; Sečovlje
EXF-6069	8	Saltern: bittern water	Slovenia; Sečovlje
EXF-6070	9	Saltern: bittern water	Slovenia; Sečovlje
EXF-6200	10	Saltern: hypersaline saltern water	Slovenia; Sečovlje
EXF-8617	11	Air: air in horse barn	Denmark, Lyngby
EXF-8618	12	Air: air in horse barn	Denmark, Lyngby
EXF-8619	13	Air: air in horse barn	Denmark, Lyngby
EXF-8621	14	Air: air in horse barn	Denmark; Lillerød
EXF-8622	15	Air: air in horse barn	Denmark; Lillerød
EXF-8623	16	Air: air in horse barn	Denmark; Lillerød
EXF-8624	17	Air: air in horse barn	Denmark; Lillerød
EXF-8760	18	Air: air in hay barn	Denmark; Lillerød
EXF-8761	19	Air: air in hay barn	Denmark; Lillerød
EXF-10826	20	Saltern: bittern water	Slovenia; Sečovlje
EXF-10937	21	Saltern: bittern water	Slovenia; Sečovlje
EXF-12845	22	Food: *Gadus* sp. (Cod fish)	Norway; Nordland, Lofoten
EXF-5753^∗^	23	Plant: pollen *Hedera helix* L.	Slovenia

*^∗^*W. hederae.**

The sequencing yielded genome coverage greater than 300 × (± 99 × SD) on average and a minimum coverage above 200×. Reference-guided assembly produced on average 286 contigs (± 186 SD). Genomes of all sequenced strains shared many similarities (Table 2 and Supplementary Table S1). Like the reference genome assembly of *W. ichthyophaga* EXF-994, the newly sequenced genomes were very compact, with an average assembly size of 9.55 Mbp (± 0.05 Mbp SD) and with 4188 (± 14 SD) predicted coding sequences covering more than two thirds of the genomes (72.10 ± 0.64% SD). GC content (45.41 ± 0.03% SD) and intron length (68 ± 0.44 bp SD) were also very uniform across the genomes. Predicted proteomes contained on average 87.95% (± 0.61% SD) of complete basidiomycetous Benchmarking Universal Single-Copy Orthologs (BUSCOs), with less than one percent of them duplicated, and additional 5.95% (± 0.43% SD) fragmented BUSCOs. Only 5.70–6.90% BUSCOs were completely absent from the annotated genomes.

**TABLE 2:** Statistics for the sequenced *W. ichthyophaga* genomes.

**Statistic^∗^**	**Minimum^∗∗^**	**Mean^∗∗^**	**Maximum^∗∗^**	**Standard deviation^∗∗^**
Coverage	221	315	653	99
Genome assembly size (Mbp)	9.51	9.55	9.7	0.05
Number of contigs	194	286	962	186
Contig N50	31781	165956	198923	45849
GC content (%)	45.34%	45.41%	45.44%	0.03%
CDS total length (Mbp)	6.76	6.89	6.92	0.04
CDS total length (% of genome)	69.75%	72.10%	72.54%	0.64%
Gene models (*n*)	4165	4188	4225	14
CDS average length (bp)	1601	1645	1661	12
Exons per gene (average)	3.7	3.76	3.79	0.02
Intron average length (bp)	67	68	68	0.44
Complete BUSCOs (%)	86.10%	87.95%	88.50%	0.61%
Complete and single-copy BUSCOs (%)	86.00%	87.75%	88.20%	0.61%
Complete and duplicated BUSCOs (%)	0.10 %	0.20%	0.80%	0.15%
Fragmented BUSCOs (%)	5.50%	5.95%	7.40%	0.43%
Missing BUSCOs (%)	5.70%	6.10%	6.90%	0.27%
SNP density (SNPs per total genome size) (%)	0.13%	0.32%	0.40%	0.08%

*^∗^Complete data for each genome is available in Supplementary Table S1. ^∗∗^Calculated from 21 genomes, not including genomes 11 (failed sequencing) and 23 (outgroup species). BUSCOs, Benchmarking Universal Single-Copy Orthologs.*

The core genome of the sequenced strains and the reference genome strain as determined by the GET_HOMOLOGUES pipeline contained 2548 genes, with additional 598 genes missing in only one or two genomes. Several categories were found to be overrepresented in this dataset. Classified by molecular functions these were: ATPase activity, transferase activity (transferring phosphorus-containing groups), ion binding, ligase activity, DNA binding, oxidoreductase activity, transmembrane transporter activity, hydrolase activity (acting on ester bonds), protein binding, RNA binding. Biological processes: cell cycle, transport, cellular response to stimulus, cellular protein modification process, transcription (DNA-templated), regulation of metabolic process, cellular localization, cellular component organization. In the cellular component classification: chromatin, catalytic complex, endomembrane system, vacuole, plasma membrane, cytosol, nuclear lumen.

In the prediction of the isolation habitat or location of the *W. ichthyophaga* strains from the presence and copy number of automatically annotated genes using decision tree forests, the number of genes encoding lysophospholipases was found to have the highest predictive value. Strains 12–14 and 16–19 (all from air in Denmark) had two copies of the gene, strains 7, 8, and 20 (from bittern in Slovenia) had a single copy, and the rest of the strains (seven from hypersaline habitats in Slovenia and one in Namibia, two from food in Slovenia and one from air in Denmark) lacked the gene entirely. However, when the genomes were manually searched for the gene, the differences in gene copy numbers could be ascribed to the differences in annotation, with all genomes containing two copies except genomes 7 and 8, which alone had only one copy per genome.

**FIGURE 1:**
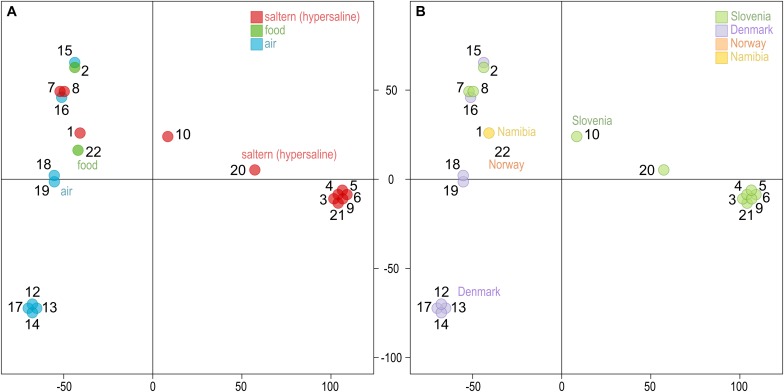
Clustering of the *Wallemia ichthyophaga* genomes. Principal component analysis of SNP data estimated by comparing the genomes sequenced in this study to the reference genome. The genomes are represented by circles, the color of which corresponds to the habitat **(A)** or sampling location **(B)** of the sequenced strains. The first two axes explain 40.6% (horizontal) and 23.4% (vertical) of variation.

Similar to other genome statistics, the density of single nucleotide polymorphisms (SNPs) was low when compared to the reference *W. ichthyophaga* genome (Table 2 and Supplementary Table S1). SNPs covered from 0.13 to 0.40% of genomes, with an average of 0.32%. The most similar to the reference genome were genomes 7 and 8, isolated from bittern. These two genomes also formed a cluster in the principal component analysis (PCA) of the SNP data (Figure 1), joined by the genome 16 isolated from air in Denmark. Three other clusters contained genomes 12, 13, 14, and 17 (all isolated from air in Denmark), genomes 3–6, 9, and 21 from hypersaline environments in Slovenia, and finally a cluster of genomes 18 and 19 again from air in Denmark. In all these cases the number of different SNP loci between pairs of genomes within clusters was below 200. Outside these clusters the minimal number of different loci was 12062 between genomes 2 and 5, while the average difference was 31448 SNPs. While clustering of some strains could be linked to habitat or isolation location, as described above, this was not absolute, with other strains from the same habitats and/or locations positioned far from the clusters. The first two axes of the PCA of SNP data explained 40.6 and 23.4% of the variation.

**FIGURE 2:**
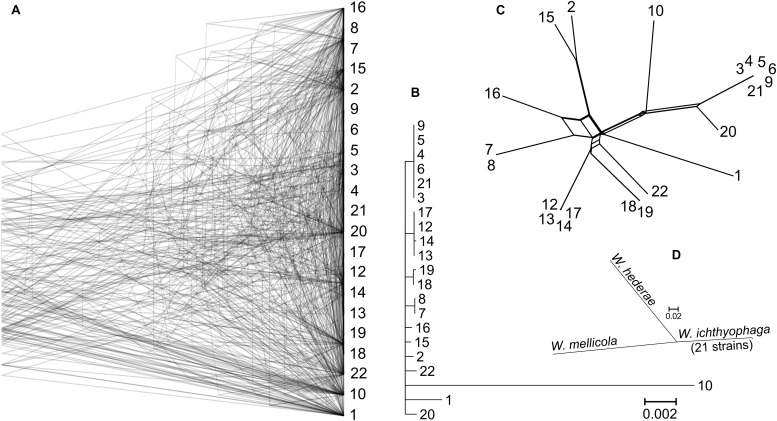
Phylogeny of *W. ichthyophaga* strains. **(A)** Overlay of 83 core Benchmarking Universal Single-Copy Ortholoe (BUSCO) gene trees estimated by PhyML 3.1 using the Hasegawa-Kishino-Yano 85 nucleotide substitution model and estimating the alpha parameter of the gamma distribution of the substitution rate categories and the proportion of invariable sites. **(B)** Majority rule consensus tree of 83 core gene trees described above. **(C)** Phylogenetic network reconstructed with the Neighbor-Net algorithm based on the dissimilarity distance matrix calculated from the SNP data. **(D)** Majority rule consensus tree of 690 gene trees of core BUSCOs from 21 genomes of *W. ichthyophaga*, with *W. hederae* and *W. mellicola* used as outgroups. Branches separating individual *W. ichthyophaga* strains are too short to be seen.

Gene trees were reconstructed for 83 BUSCOs that were present as single-copy genes in all sequenced genomes and that differed between strains by at least 15 nucleotides (on average per tree). The topologies of the trees differed substantially (Figure 2). As shown by the majority rule consensus tree, only three internal tree nodes were present in more than half of the trees – the nodes at the basis of the clusters seen also on the PCA plot of SNP data: cluster of strains 7 and 8; clusters 12, 13, 14, and 17; clusters 3–6, 9, and 21. The same clusters were also observed on the minimum spanning network. Here some (but not all) other strains from the same habitats/geographical locations were also positioned in the same parts of the network. These strains also clustered together in the analysis of the population structure with the Structure software (Supplementary Figure S1). The latter analysis indicated a possible existence of admixed individuals (e.g., 10, 16, 22). Some reticulation was visible on the phylogenetic network reconstructed from the SNP data (Figure 2). Phylogenetic trees of 690 core BUSCOs of genomes of *W. ichthyophaga*, *W. hederae*, and *W. mellicola* confirmed the distinct phylogenetic positions of each species (Figure 2).

**FIGURE 3:**
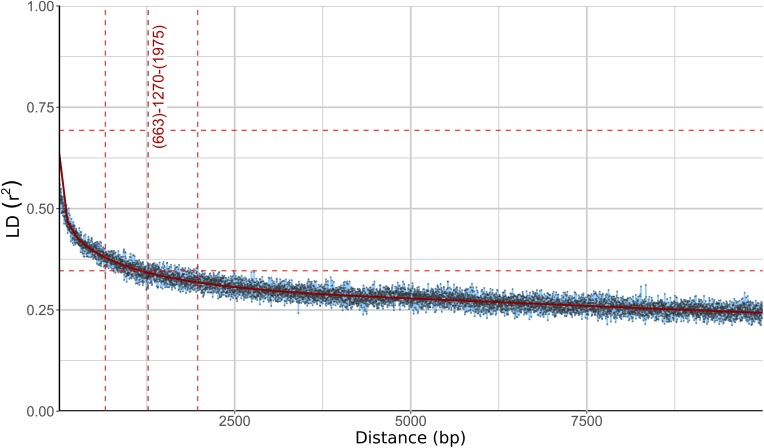
Linkage disequilibrium (LD) decay in *Wallemia ichthyophaga* estimated on all biallelic loci. Squared correlation coefficient (*r*^2^) between pairs of SNP loci is plotted against the physical distance of the loci in the genome. Horizontal lines mark the maximum observed value and half of the maximum observed value. Left and right vertical lines mark the interval of the physical distance delimited by the first point of the curve under and the last point above half of the maximum *r*^2^ value. The middle vertical line marks the point where the fitted curve intersects with half of the maximum *r*^2^ value.

Different phylogenetic histories of genes in the same individual can be a sign of recombination within the population. To investigate this possibility further, linkage disequilibrium (LD) between pairs of biallelic SNP loci was calculated and plotted against the distance between the loci. In recombining organisms LD is expected to decrease with increasing distance (and thus increased likelihood of recombination) between the loci. In *W. ichthyophaga* the squared correlation coefficient (*r*^2^) fell to half of its maximum value at the inter-loci distance of 663–1975 bp and the fitted curve intersected the half of the maximum *r*^2^ value at 1270 bp (Figure 3). The LD decay value could not be determined with great precision due to a relatively gentle slope of the curve and substantial amount of noise. Similarly, the LD decay value determined with the normalized coefficient of LD (*D*′) instead of *r*^2^ was between 128 and 2566 (Supplementary Figure S2).

**FIGURE 4:**
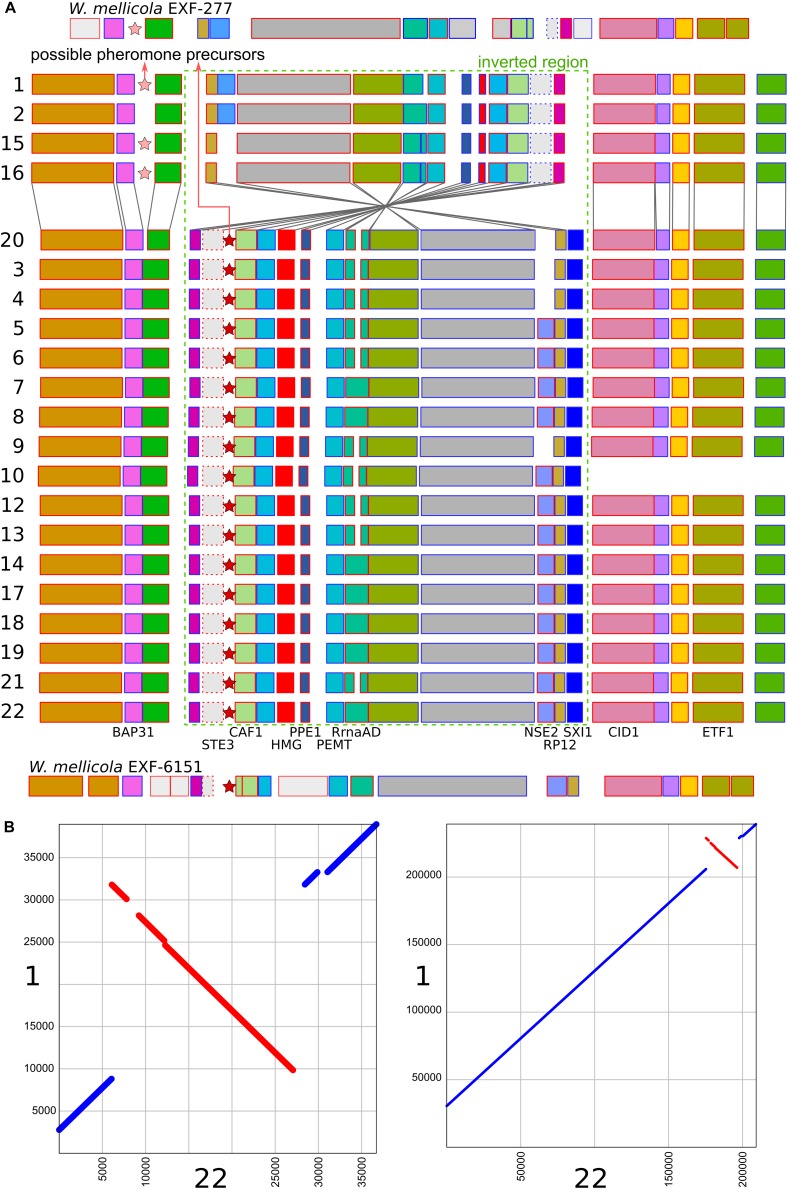
Putative mating-type loci in different strains of *Wallemia ichthyophaga*. **(A)** Annotated genes of the putative mating-type loci and their flanking regions. The orientation of the genes is marked with blue (left to right) and red (right to left) outlines of the rectangles. The region of the chromosomal inversion is marked with a dashed green rectangle, locations of possible pheromone precursor genes are marked with stars. Strain numbers are on the left. Mating loci from two strains of *W. mellicola* were added for comparison (adapted from Padamsee et al., 2012 and Sun et al., 2019). **(B)** Alignment of putative mating-type loci and their flanking regions from genomes 1 (x axis) and 22 (y axis) at two different magnifications.

A region similar to the putative mating-type locus identified by Padamsee et al. (2012) in *W. mellicola* was found in all of the here sequenced genomes (Figure 4 and Supplementary File S1). Both the gene encoding a putative homeodomain-motif transcription factor Sxi1 as well as a gene encoding a putative transcription factor with a High Mobility Group (HMG) DNA binding motif were found during the automatic annotation, while an additional manual search also identified the gene STE3 encoding a pheromone receptor. Compared to the majority of strains and also to the reference genome of *W. ichthyophaga* EXF-994, the putative mating locus was found to be inverted in the genomes 1, 2, 15, and 16. While the inverted and flanking regions were highly similar between the two groups of strains (“reference” and “inverted”), there were substantial differences at four locations: (i) only in the genomes 1, 2, 15, and 16 there was an additional 1150 bp long sequence flanking the BAP31 gene, with no similarity to any of the sequences in the GenBank database; (ii) the HMG motif gene and its upstream region lacked a 432 bp long sequence in the genomes 1, 2, 15 and 16, while the rest of the gene differed from “reference” homolog in 19% of aligned positions; (iii) the proportion of differing nucleotide positions in the alignment of STE3 from different mating loci was 53%; (iv) in the genomes 1, 2, 15, and 16 the SXI1 gene was replaced by a sequence with unidentifiable function (Supplementary File S1). For comparison: in the alignment of the other parts of the putative mating loci from all sequenced strains the proportion of variable positions was less than 1.5%. Two possible genes for pheromone precursors were found, both encoding proteins with a prenyl group binding CAAX motif at the C-terminus. The gene for a protein with the sequence MEPETLKNIFFQEASQDHLKDEAILFETSIGMPEISSEDLANS NNPINDSTGGDNADTMYCIIV was located in the region present only in the genomes 1, 2, 15, and 16 next to the BAP31 gene, while in all other genomes a region between STE3 and CAF1 contained a putative gene encoding a protein MIPEIEVSQIQVDQVHINVEREEPGENETYGSSSGCIIT (Figure 4 and Supplementary File S1). Strains 1, 2, 15, and 16 do not represent a clonal lineage. They originated from different habitats and geographic locations and are located far apart in the minimum spanning network of strains (Supplementary Figure S1). No other chromosomal inversions in the corresponding contigs were identified (Figure 4).

## Discussion

When the genome of *W. ichthyophaga* was first sequenced, it was found to differ from other basidiomycetes in several ways, confirming the isolated phylogenetic position of the sub-phylum *Wallemiomycotina*. Most striking was the compactness of its genome, which was among the smallest in sequenced Basidiomycota (9.63 Mbp) (Zajc et al., 2013) and almost five times smaller from an average basidiomycetous genome (Mohanta and Bae, 2015). Equally striking was the number of predicted genes, which, although covering more than two thirds of the genome, were in the range of bacterial genomes at 4884 predicted genes – the median protein count of *E. coli* genome at the time of writing is 4948 (GenBank April 9, 2019), while the average number of genes in a basidiomycetous genome as reported by Mohanta and Bae (2015) was 15432 (and 11129 in an average ascomycetous genome) (Mohanta and Bae, 2015). The 21 genomes sequenced here confirm this observation and also show a high similarity of genomes between different *W. ichthyophaga* strains (Table 2 and Supplementary Table S1). The genome sizes of these genomes were in the range 9.51–9.7 Mbp with a standard deviation of only 0.05 Mbp. Similarly, the average number of predicted genes was 4188 with a standard deviation of only 14. The number of predicted genes was smaller than in the reference genome, possibly due to different assembly and annotation approaches used here. The small number of genes was also reflected in the soft core genome of *W. ichthyophaga*, which, with only 3146 genes, was much smaller than the almost 5000-gene large core genome of *S. cerevisiae*, even though the latter was calculated on a much larger number of strains (Peter et al., 2018). Other genome characteristics were also similar to those of the reference genome (Table 2 and Supplementary Table S1).

The analysis of genomes uncovered four strain clusters with clonal origin, two from hypersaline habitats in Slovenia and two from air in Denmark (Figure 1 and Supplementary Table S2). The differences between the rest of the genomes did not clearly reflect either the isolation habitat or the geographical origin of the strains. Thus, while some clonal lineages were found in the same geographical location and some other strains clustered together in correspondence to their origin, there was no clear indication for the existence of cryptic (possibly inbreeding) populations, such as was observed in some other fungal species (Ellison et al., 2011; Branco et al., 2015, 2017; Duan et al., 2018; Peter et al., 2018). Apart from the above mentioned clonal clusters, the two strains with the fewest SNPs between them were 2 and 15, from different locations and different habitats, one from salt-preserved food in Slovenia, the other from air in Denmark. The genomes with the fewest SNPs compared to the reference genome were genomes 7 and 8, corresponding to strains isolated from a hypersaline habitat at the same geographical location as the reference strain. However, strains 7 and 8 were isolated in 2009, 10 years after the isolation of the reference genome and type strain *W. ichthyophaga* EXF-994 and strain 21 was isolated 6 years after the isolation of other clones in its cluster. This points to local persistence of a population of relatively closely related strains over at least several salt-harvesting seasons. This does not mean, however, that there is a lack of diversity even in areas as small as a few 100 m. The same bittern habitat sampled in the same location and year also yielded strain 9 that differed from strain 7 in 37464 SNPs – more than the difference between strains 7 and 18 (24556 SNPs), although the latter was isolated from the air over 1000 km away. Unfortunately, until more strains of *W. ichthyophaga* are isolated, the conclusions of such observations will necessarily be limited by the relatively small amount of obtainable data.

The soft core genome was enriched, as expected, with proteins involved in the fundamental cellular processes, such as cell cycle and transcription. It also contained a higher than expected number of proteins involved in transmembrane transport – a crucial cellular function, but also of utmost importance in hypersaline conditions, where maintaining physiological intracellular concentrations of inorganic and organic solutes becomes challenging (Plemenitaš et al., 2016). Retaining the full spectrum of available transporters may be of particular importance for *W. ichthyophaga*, since it was shown that unlike some other fungi from hypersaline environments, *W. ichthyophaga* does not contain a redundancy of transporters of inorganic cations (Zajc et al., 2013).

The consensus of phylogenies of core Benchmarking Universal Single-Copy Orthologs confirmed the distinct phylogenetic positions of *W. ichthyophaga*, *W. mellicola*, and *W. hederae* as reported by Jančič et al. (2016) based on a much smaller dataset. However, there was a lack of concordance between the gene phylogenies within *W. ichthyophaga* (Figure 2). Different phylogenies of the genes in the same organism are a good indicator of recombination (Tibayrenc and Ayala, 2012). Another well-established measure of the amount of recombination in a population is linkage disequilibrium (LD) decay – the distance over which LD falls to half of its maximum value due to the increased likelihood of recombination over larger distances (Taylor et al., 2015). In *W. ichthyophaga* the LD decay distance was around 1270 bp when measured by the squared correlation coefficient *r*^2^ (Figure 3). This value is somewhat larger than that observed by Nieuwenhuis and James (2016) for several sexually reproducing fungi, but still far below the values reported for highly clonal species such as *B. dendrobatidis* and *C. albicans* where LD decay distances surpass 100 or even 200 kbp (Nieuwenhuis and James, 2016). The finding of such relatively strong indications of recombination in *W. ichthyophaga* were surprising for two reasons. The first was the long-held belief that the species is asexual (Plemenitaš et al., 2014). While several genes with possible roles in mating were identified when the first genome was sequenced, a well-defined mating-type locus could not be discerned. Additionally, many of the genes involved in meiosis were lacking in the genome (Zajc et al., 2013). Here, however, we describe a putative mating-type locus found in all 21 sequenced *W. ichthyophaga* genomes (Figure 4). The locus is similar to the one described in *W. mellicola* (Padamsee et al., 2012) and is inverted in four of the sequenced strains. This inverted version of the locus contains a truncated (possibly non-functional) homolog of a High-Mobility Group protein encoding gene and lacks a discernible homolog of the homeodomain proteins SXI1 or SXI2. An additional insertion/deletion between the two mating types was observed outside of the inverted region, next to the gene BAP31. While most of the inverted region was highly similar between the strains, the variability was 10–40-times larger in the genes encoding proteins involved in mating – the HMG motif containing protein and the pheromone receptor STE3 (Supplementary File S1), additionally indicating that the two identified versions of the putative MAT locus might indeed represent two different mating types of *W. ichthyophaga*. Chromosomal rearrangements including inversions of the mating-type locus and its surrounding are not unusual (Coelho et al., 2017). In methylotrophic ascomycetous yeasts the mating-type locus is present in two copies and switching occurs by chromosomal inversion, placing one cope in proximity to centromeric or telomeric chromatin and thus silencing its expression (Maekawa and Kaneko, 2014). In case of *W. ichthyophaga*, however, only one copy of the putative mating-type locus was found in each genome and it was the only non-syntenic region on the corresponding contig when comparing different strains of *W. ichthyophaga* (Figure 4). A similar arrangement of the putative mating-type loci was found in the genomes of 25 *W. mellicola*, with a locus inversion in approximately half of the strains (Sun et al., 2019).

The second reason for the past assumption that *W. ichthyophaga* is strictly clonal was its ecology. The species is an obligate halophile, unable to grow at water activity above 0.96 (Zalar et al., 2005) and judging by its exceedingly rare isolation from nature even with targeted efforts (Jančič et al., 2016) its abundance in nature is low. While several strains used in this study have been isolated from air, which might seem to indicate that *W. ichthyophaga* is not limited to extreme habitats such as hypersaline water and could therefore be relatively widespread, it should be noted that despite considerable sampling efforts it has only been found in a very small share of all collected air samples (Jančič et al., 2016). One of the traits that has been suggested as advantageous in extreme environments is asexuality. Its proposed benefits range from avoiding the costly forming of sexual structures to maintaining successful genomic configurations, which would be broken by recombination (the so-called recombination load) (Otto, 2009; Gostinčar et al., 2010, 2015, 2018). Indeed, one of the most extremely halotolerant fungi, the black yeast *Hortaea werneckii*, appears to be largely devoid of recombination between intraspecific phylogenetic lineages, even though the formation of the current diploid strains of *H. werneckii* is best explained by rare hybridizations between haploid strains (Gostinčar et al., 2018). Contrary to this, *W. ichthyophaga* appears to be another species on the growing list of fungi that were thought to be asexual, but were later showed to be recombining based on population genetic analyses, starting with *Coccidioides immitis* (Burt et al., 1996; Taylor et al., 2015), *F. oxysporum* (Koenig et al., 1997), *Aspergillus flavus* (Geiser et al., 1998), and *Cenococcum geophilum* (LoBuglio and Taylor, 2002). With the employment of the more powerful population genomics for this purpose (Carreté et al., 2018), the list of cryptically recombining species is expected to become much longer.

If *W. ichthyophaga* is really capable of sexual recombination, its meiotic machinery possibly differs from that described in other fungi. Only three out of eight meiosis-specific homologs, which are generally thought to provide strong indications of capability for meiosis (Schurko et al., 2009), were identified in the genome of the reference *W. ichthyophaga* EXF-994 (Zajc et al., 2013). Another possibility is that the reported decay of LD is not a consequence of sexual recombination, but a result of recombination mechanisms that do not require meiosis, such as parasexuality followed by mitotic haploidization (Pontecorvo et al., 1953). What is the mechanism of recombination in *W. ichthyophaga*, how can recombination occur in a species that is seemingly limited to habitats that are rare and far apart, is *W. ichthyophaga* in fact more abundant and better adapted to long-distance dispersion than currently thought? – these are all questions that should be addressed by future research.

## Conclusion

*Wallemia ichthyophaga*, one of the most halophilic fungi ever described, is rarely isolated from nature. The existing isolates have been found in concentrated sea water, in air and on food preserved with low water activity. Genome sequencing of 21 *W. ichthyophaga* strains showed that the compact genome and low gene count are general characteristics of the species. Genome comparison indicated that while clonal lineages can be found in the same location, where they appear to persist over at least several years, phylogenetically distinct lineages can be present at the same location as well. The similarity between the genomes was not a good predictor of either the isolation habitat or geographic location. Finally, the lack of concordance between gene phylogenies, the decay of linkage disequilibrium, and the identification of a mating-type locus reported here, provide strong support to the existence of (sexual) recombination in *W. ichthyophaga.*

## Materials and Methods

### Culture, Medium, and Growth Conditions

Strains of the obligately halophilic basidiomycete *W. ichthyophaga* (Table 1) were obtained from the Ex Culture Collection of the Department of Biology, Biotechnical Faculty, University of Ljubljana (Slovenia). One strain of a closely related species *W. hederae* was added to the dataset as an outgroup. The cultivation and DNA isolation were performed as described previously (Gostinčar et al., 2018). In short, biomass was grown in the standard chemically defined Yeast Nitrogen Base medium (Qbiogene) with 0.5% (w/v) ammonium sulfate, and 2% (w/v) glucose. The pH was adjusted to 7.0 prior to autoclaving. For solid medium, 2% (w/v) agar was added. Liquid cultures were grown on a rotary shaker at 180 rpm and at 24°C. The cells were harvested in the mid-exponential growth phase (approximated by the lowering of the medium pH to pH 4) by centrifugation (5000 × *g* for 10 min). The cell pellets were frozen in liquid nitrogen and kept at −80°C until DNA isolation.

### DNA Isolation

Fungal biomass was homogenized using a pestle and mortar, while being kept frozen using liquid nitrogen. Then, 100 mg of homogenate was transferred to 2-ml microcentrifuge tubes, each of which contained a stainless steel ball. Further homogenization was performed for 1 min using Retsch Mixer Mill 301 (Thermo Fisher Scientific, United States) at 20 Hz, keeping the tubes in holders pre-cooled with liquid nitrogen. Then 300 μL MicroBead Solution buffer was added (provided in the UltraClean Microbial DNA isolation kit; see below), and the mixtures were completely thawed on ice. These homogenates were used for DNA extraction with UltraClean Microbial DNA isolation kit (MO BIO Laboratories, United States), according to the manufacturer instructions. Contaminating RNA was removed using RNAse A (Thermo Fisher Scientific, United States). The quantity, purity and integrity of the isolated DNA were evaluated using agarose electrophoresis, spectrophotometer (NanoDrop 2000; Thermo Fisher Scientific, United States), and by fluorometry (Qubit; Thermo Fisher Scientific, United States).

### Genome Sequencing

The genome sequencing was performed using the platform BGISEQ-500, with 2-bp × 150-bp libraries, prepared as described previously (Fang et al., 2018), with multiplexed barcodes. The resulting output was demultiplexed, the quality was checked with FastQC, and the reads were trimmed for adaptors and quality (removal of bases with Q < 20) using the ‘bbduk’ script^1^.

The sequencing reads, assembly and annotation data have been deposited in GenBank under BioProject PRJNA527765. Data are also deposited in the China National GenBank Sequence Archive, CNSA^2^ with accession code CNP0000446.

### Variant Calling

Sequencing reads were mapped to the reference genome of *W. ichthyophaga* EXF-994 (GenBank APLC00000000.1) (Zajc et al., 2013) with ‘bwa mem,’ using the default parameters. Samtools 1.6 was used for sorting the mapped reads and calculating their density (Li et al., 2009), and duplicates were identified with Picard 2.10.2. The density of the reference genome coverage by sequencing reads was visualized in R with ‘ggplot2’ (Wickham, 2009; R Development Core Team, 2017). Variant calling was performed with Genome Analysis Toolkit 4.1 (Alkan et al., 2011), following the ‘Genome Analysis Toolkit (GATK) Best Practices’ workflow, but using the ‘hard filtering’ option (the filtering parameters were ‘QD < 20.0 || MQ < 40.0 | | FS > 50.0 || SOR > 8.0 || DP > 5000’). Ploidy was set to haploid.

### Assembly and Annotation

The genomes were assembled using IDBA-Hybrid 1.1.3 (Peng et al., 2012). Genome of *W. ichthyophaga* EXF-994 (GenBank APLC00000000.1) (Zajc et al., 2013) was used as reference to guide the assembly process. The maximum k value selected was 120, the minimum support in each iteration was 2, the similarity threshold for alignment was 0.95, seed kmer was 20, maximum allowed gap in the reference was 100, and the minimum size of contigs included in the final assembly was 500.

Annotation of protein-coding and tRNA genes was performed using MAKER 2.31.8 (Campbell et al., 2014). The evidence for annotation was the fungal subset of the Swissprot database (recovered on 12. 06. 2018), and the predicted proteome of the reference *W. ichthyophaga* EXF-994 genome (Zajc et al., 2013). Two *ab initio* gene predictors were used in the MAKER pipeline: Augustus, with the training parameters for *Laccaria bicolor* (Stanke and Morgenstern, 2005); and Semi-HMM-based Nucleic Acid Parser (SNAP) (Korf, 2004), which was bootstrap-trained within MAKER as recommended by Campbell et al. (2014), based on the gene models derived from the alignment of the *W. ichthyophaga* EXF-994 proteins to the genome in the first bootstrap iteration, and with the Swissprot protein evidence added in the second and third training iteration.

The genome assembly and gene prediction completeness was evaluated with the BUSCO 3 software (Simão et al., 2015), in proteomic mode, using the dataset for basidiomycetes (Kriventseva et al., 2015). All of the parameters were used as the default values.

The files for submission to GenBank were prepared with the Genome Annotation Generator (GAG) 2.0.1 (Geib et al., 2018). All gene models with a codon region shorter than 150 bp or with introns shorter than 10 bp were removed from the final dataset.

### Variant-Based Analysis

Principal component analysis of the SNP data was performed with the ‘glPca’ function from the ‘adgenet’ package (Jombart and Ahmed, 2011). Linkage disequilibrium was estimated on a dataset of biallelic SNP loci. For each pair of loci, the normalized coefficient of LD (*D*′) and the squared correlation coefficient (*r*^2^) were calculated using ‘vcftools’ (Danecek et al., 2011). To investigate LD decay, *D*′ and *r*^2^ of loci within 10,000 nucleotides from each other were plotted as a function of distance and a generalized additive model (“gam”) fitted curve was added using ‘ggplot2’ in Wickham (2009) and R Development Core Team (2017). The LD decay range was determined as the interval outside which all of the arithmetic means of *D*′ or *r*^2^ were either higher (left interval border) or lower (right interval border) than half of the maximum observed *D*′ or *r*^2^ means. Population structure was investigated using the software Structure 2.3.4 (Pritchard et al., 2000), investigating from 1 to 6 maximum population numbers (*K*) with three runs for each *K*. The analysis was run on the whole dataset and also after exclusion of all but genome in each cluster of clonal strains.

### Phylogenetic Analysis

Gene phylogenetic trees were constructed from the predicted coding sequences of all complete and single-copy BUSCOs. Sequences were aligned with MAFFT 7.407, with the ‘*--*auto’ option and default parameters (Katoh and Toh, 2008). This alignment was optimized using Gblocks 0.91, with the options ‘−b3 = 10 −b4 = 3 −b5 = n’ (Talavera and Castresana, 2007); if the resulting alignment was longer than 200 nucleotides and the average number of nucleotide differences between the sequence pairs was larger than 15 [as counted by the ‘infoalign’ tool included in EMBOSS 6.6.0.0 (Rice et al., 2000)], the alignment was used for reconstruction of the phylogeny with PhyML 3.1 (Guindon et al., 2010). The Hasegawa-Kishino-Yano 85 (Hasegawa et al., 1985) nucleotide substitution model was used, with the alpha parameter of the gamma distribution of substitution rate categories and the proportion of invariable sites estimated by PhyML. The resulting trees were visualized using DensiTree 2.2.5 (Bouckaert, 2010). A majority rule consensus tree was calculated with the ‘consensus.edges’ function of the package ‘phytools’ in R, using the default parameters (Revell, 2012; R Development Core Team, 2017). A separate analysis was performed using the same workflow and including the BUSCOs from *W. hederae* (sequenced and annotated here) and *W. mellicola* strain CBS 633.66 (GenBank AFQX00000000.1) (Padamsee et al., 2012).

The networks were reconstructed from the SNP data. The dissimilarity distance matrix was calculated using the R package ‘poppr’ (Kamvar et al., 2015), and was used to construct the phylogenetic network with the Neighbor-Net algorithm, as implemented in the R package ‘phangorn’ (R Development Core Team, 2017; Schliep et al., 2017). A minimum spanning network based on SNP data was constructed with the ‘poppr.msn()’ function based on bitwise distances between the genomes (Kamvar et al., 2015). Each clonal lineage was clustered together into a single network node with the function ‘mlg.filter().’

### Core Genome, GO Enrichment

Pipeline GET_HOMOLOGUES 3.0.8 (Vinuesa and Contreras-Moreira, 2015) was used to estimate the core genome *W. ichthyophaga* from the predicted proteomes of all here sequenced strains and the reference strain *W. ichthyophaga* EXF-994 (Zajc et al., 2013) as a consensus of COGtriangle and OrthoMCL algorithms using default parameters. Representative sequences of each protein cluster were annotated using the PANTHER HMM scoring tools 2.1 and the HMM library version 13.1 (Thomas, 2003). Statistically significant enrichment of GO-Slim terms was investigated at www.pantherdb.org for the lists of core gene clusters (present in all 22 genomes) and soft core gene clusters (in at least 20 genomes) with a list of all gene clusters used as a reference list. Fisher’s Exact test and the False Discovery Rate correction were used.

Additionaly, gene clusters of GET_HOMOLOGUES (the number of homologs in the clusters) were used for the prediction of the habitat or isolation location. The R package ‘randomForest’ (Tang et al., 2004; R Development Core Team, 2017) was used for the construction of decision tree forests for the prediction of either condition (habitat or location) and the gene clusters that could best predict the condition were identified. The presence/absence of genes represented by these clusters was checked manually for each sequenced genome by searching the genomes using BLAST (Altschul, 1997).

### Mating-Type Loci

Homologs of the genes contained in the putative mating-type locus of *W. mellicola* (Padamsee et al., 2012) were identified by BLAST (Altschul, 1997) searches against the assembled and annotated *W. ichthyophaga* genomes and predicted proteomes. The functional annotations of the genes were assigned according to Padamsee et al. (2012). The approximate position of the gene STE3 was determined by manually searching the investigated region. The detailed comparison of the loci was performed by separately aligning the upstream and downstream regions of the inversion, and the inverted region itself, in the latter case using the reverse complement sequence of the strains 1, 2, 15, and 16. Alignment was performed with MAFFT 7.407, with the ‘*--*auto’ option and default parameters (Katoh and Toh, 2008). Putative mating loci and their flanking regions were visualized in R with ‘ggplot2’ (Wickham, 2009; R Development Core Team, 2017). The corresponding regions of the genomes were aligned and the alignments visualized using Mummer 3.23 (Kurtz et al., 2004).

## Data Availability

The datasets generated for this study can be found in GenBank and China National GeneBank Sequence Archive, CNSA (https://db.cngb.org/cnsa/), PRJNA527765 (GenBank), CNP0000446 (CNSA).

## Author Contributions

NG-C, ZS, and CG contributed to the conceptualization of the study. XS and JZ contributed to the experimental work. CG contributed to the bioinformatic analyses, data curation, preparation of the manuscript, and visualizations. ZS, NG-C, YL, and YH provided resources and acquired funding. CG, NG-C, XS, and ZS reviewed and edited the manuscript. ZS and NG-C supervised the project.

## Conflict of Interest Statement

The authors declare that the research was conducted in the absence of any commercial or financial relationships that could be construed as a potential conflict of interest.

## Footnotes

1 https://jgi.doe.gov/data-and-tools/bbtools/

2 https://db.cngb.org/cnsa/

## Supplementary Material

FIGURE S1 Minimum spanning networks of *W. ichthyophaga* genomes based on SNP data and colored according to their habitat **(A)** and country of origin **(B)**. Structure analysis of the *W. ichthyophaga* population based on SNP data using the whole dataset and a maximum of five populations **(C)** and a dataset with only one representative genome from each clonal lineage and a maximum of three **(D)** or four **(E)** populations.

FIGURE S2 Linkage disequilibrium (LD) decay in *Wallemia ichthyophaga* estimated on all biallelic loci measured by the normalized coefficient of LD (*D*′), plotted against the physical distance of the loci in the genome.

TABLE S1 Statistics of the sequenced *W. ichthyophaga* genomes.

TABLE S2 Number of different SNP loci between pairs of *W. ichthyophaga* genomes.

FILE S1 Aligned sequences of the mating-type loci and their flanking regions in different strains of *W. ichthyophaga*.
