# Supplementary figures and images for: Population Genomics of an Obligately Halophilic Basidiomycete Wallemia ichthyophaga
Source: Front Microbiol. 2019 Sep 4;10:2019. doi: 10.3389/fmicb.2019.02019 (PMC6738226; doi:10.3389/fmicb.2019.02019)

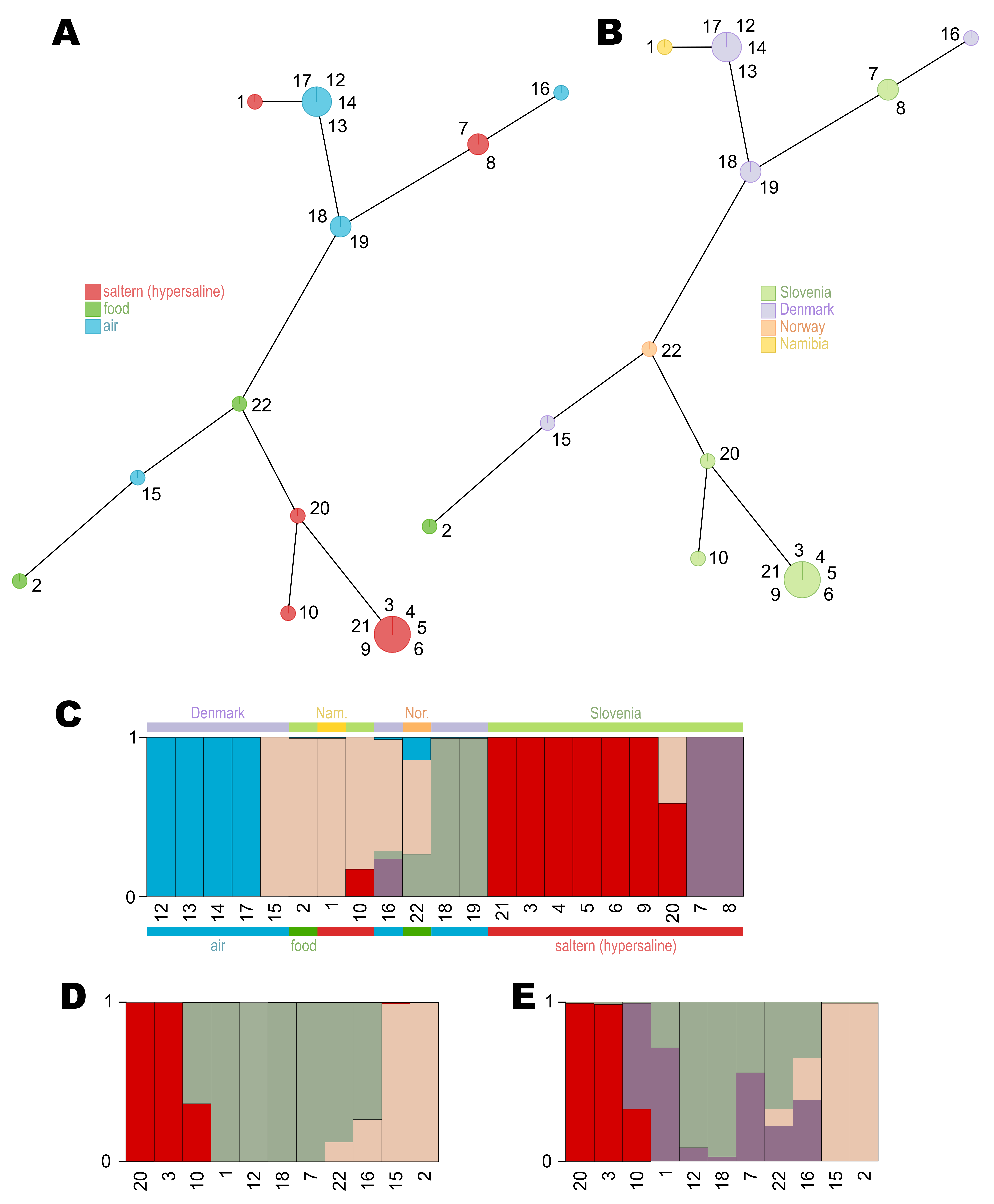

Supplement: FIGURE S1 — Minimum spanning networks of W. ichthyophaga genomes based on SNP data and colored according to their habitat (A) and country of origin (B). Structure analysis of the W. ichthyophaga population based on SNP data using the whole dataset and a maximum of five populations (C) and a dataset with only one representative genome from each clonal lineage and a maximum of three (D) or four (E) populations. [file Image_1.TIF]

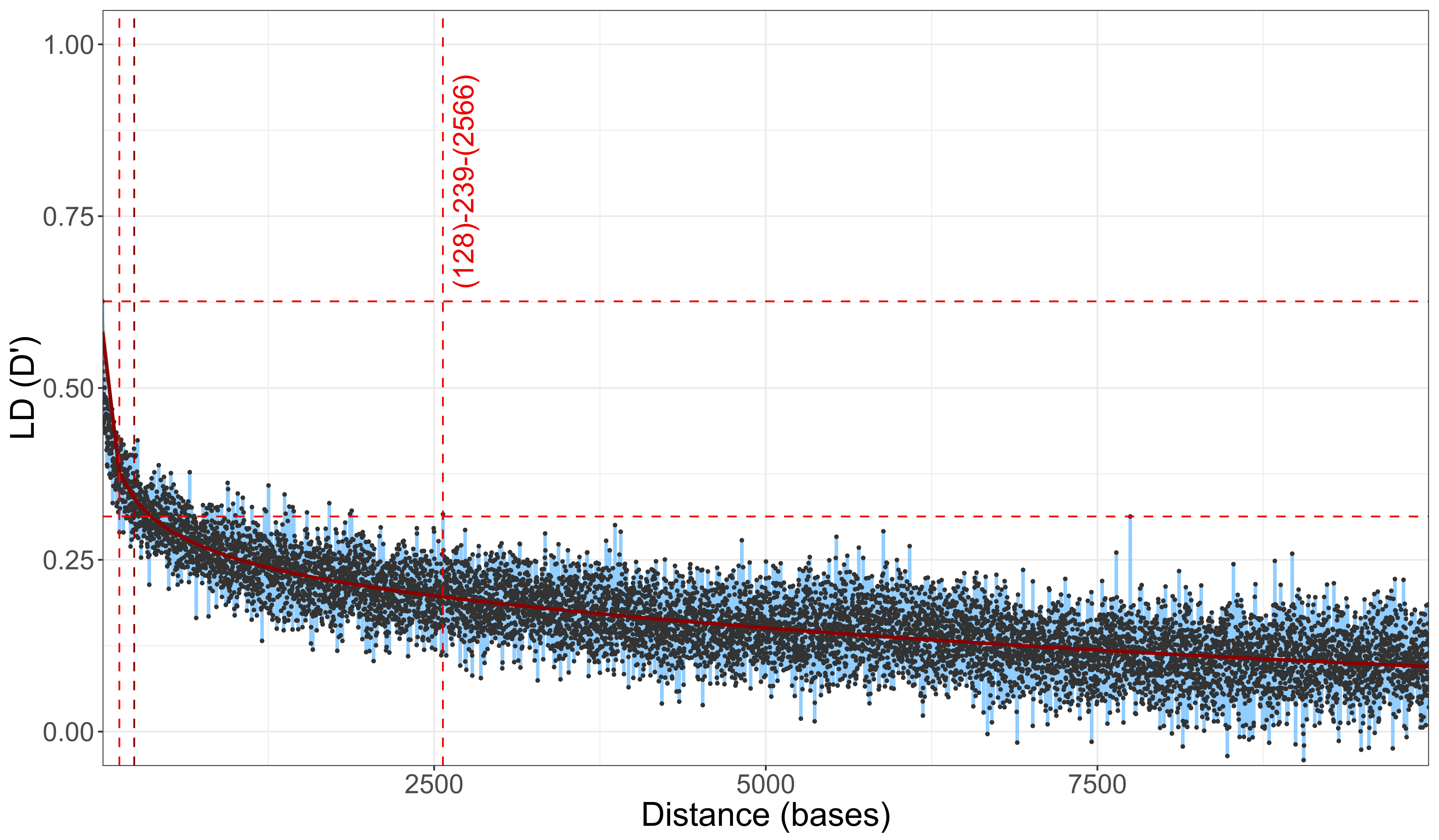

Supplement: FIGURE S2 — Linkage disequilibrium (LD) decay in Wallemia ichthyophaga estimated on all biallelic loci measured by the normalized coefficient of LD (D′), plotted against the physical distance of the loci in the genome. [file Image_2.TIF]
