# Supplementary material for: Population Genomics of an Obligately Halophilic Basidiomycete Wallemia ichthyophaga
Source: Front Microbiol. 2019 Sep 4;10:2019. doi: 10.3389/fmicb.2019.02019 (PMC6738226; doi:10.3389/fmicb.2019.02019)
Supplement: FILE S1 — Aligned sequences of the mating-type loci and their flanking regions in different strains of W. ichthyophaga. [file Table_3.DOC]

Alignment of mating-type loci of *Wallemia ichthyophaga* strains 1-22 with including and downstream regions. The marked genes correspond to named genes on Fig. 4. Introns are not marked.

**UPSTREAM**

1 ACCTCTCAATATAGCTATCAGCGTCATAAAGTTATTCAGCTGCTGACACTTGAAAGCAAG

16 ACCTCTCAATATAGCTATCAGCGTCATAAAGTTATTCAGCTGCTGACACTTGAAAGCAAG

15 ACCTCTCAATATAGCTATCAGCGTCATAAAGTTATTCAGCTGCTGACACTTGAAAGCAAG

2 ACCTCTCAATATAGCTATCAGCGTCATAAAGTTATTCAGCTGCTGACACTTGAAAGCAAG

10 ACCTCTCAATATAGCTATCAGCGTCATAAAGTTATTCAGCTGCTGACACTTGAAAGCAAG

17 ACCTCTCAATATAGCTATCAGCGTCATAAAGTTATTCAGCTGCTGACACTTGAAAGCAAG

14 ACCTCTCAATATAGCTATCAGCGTCATAAAGTTATTCAGCTGCTGACACTTGAAAGCAAG

13 ACCTCTCAATATAGCTATCAGCGTCATAAAGTTATTCAGCTGCTGACACTTGAAAGCAAG

12 ACCTCTCAATATAGCTATCAGCGTCATAAAGTTATTCAGCTGCTGACACTTGAAAGCAAG

8 ACCTCTCAATATAGCTATCAGCGTCATAAAGTTATTCAGCTGCTGACACTTGAAAGCAAG

7 ACCTCTCAATATAGCTATCAGCGTCATAAAGTTATTCAGCTGCTGACACTTGAAAGCAAG

19 ACCTCTCAATATAGCTATCAGCGTCATAAAGTTATTCAGCTGCTGACACTTGAAAGCAAG

18 ACCTCTCAATATAGCTATCAGCGTCATAAAGTTATTCAGCTGCTGACACTTGAAAGCAAG

22 ACCTCTCAATATAGCTATCAGCGTCATAAAGTTATTCAGCTGCTGACACTTGAAAGCAAG

21 ACCTCTCAATATAGCTATCAGCGTCATAAAGTTATTCAGCTGCTGACACTTGAAAGCAAG

9 ACCTCTCAATATAGCTATCAGCGTCATAAAGTTATTCAGCTGCTGACACTTGAAAGCAAG

6 ACCTCTCAATATAGCTATCAGCGTCATAAAGTTATTCAGCTGCTGACACTTGAAAGCAAG

5 ACCTCTCAATATAGCTATCAGCGTCATAAAGTTATTCAGCTGCTGACACTTGAAAGCAAG

4 ACCTCTCAATATAGCTATCAGCGTCATAAAGTTATTCAGCTGCTGACACTTGAAAGCAAG

3 ACCTCTCAATATAGCTATCAGCGTCATAAAGTTATTCAGCTGCTGACACTTGAAAGCAAG

20 ACCTCTCAATATAGCTATCAGCGTCATAAAGTTATTCAGCTGCTGACACTTGAAAGCAAG

************************************************************

1 TCGGATGAATTTACTGACAACAGCTGAGCGATCCTCTGGTGAGTCTGTGATGAGGATCTC

16 TCGGATGAATTTACTGACAACAGCTGAGCGATCCTCTGGTGAGTCTGTGATGAGGATCTC

15 TCGGATGAATTTACTGACAACAGCTGAGCGATCCTCTGGTGAGTCTGTGATGAGGATCTC

2 TCGGATGAATTTACTGACAACAGCTGAGCGATCCTCTGGTGAGTCTGTGATGAGGATCTC

10 TCGGATGAATTTACTGACAACAGCTGAGCGATCCTCTGGTGAGTCTGTGATGAGGATCTC

17 TCGGATGAATTTACTGACAACAGCTGAGCGATCCTCTGGTGAGTCTGTGATGAGGATCTC

14 TCGGATGAATTTACTGACAACAGCTGAGCGATCCTCTGGTGAGTCTGTGATGAGGATCTC

13 TCGGATGAATTTACTGACAACAGCTGAGCGATCCTCTGGTGAGTCTGTGATGAGGATCTC

12 TCGGATGAATTTACTGACAACAGCTGAGCGATCCTCTGGTGAGTCTGTGATGAGGATCTC

8 TCGGATGAATTTACTGACAACAGCTGAGCGATCCTCTGGTGAGTCTGTGATGAGGATCTC

7 TCGGATGAATTTACTGACAACAGCTGAGCGATCCTCTGGTGAGTCTGTGATGAGGATCTC

19 TCGGATGAATTTACTGACAACAGCTGAGCGATCCTCTGGTGAGTCTGTGATGAGGATCTC

18 TCGGATGAATTTACTGACAACAGCTGAGCGATCCTCTGGTGAGTCTGTGATGAGGATCTC

22 TCGGATGAATTTACTGACAACAGCTGAGCGATCCTCTGGTGAGTCTGTGATGAGGATCTC

21 TCGGATGAATTTACTGACAACAGCTGAGCGATCCTCTGGTGAGTCTGTGATGAGGATCTC

9 TCGGATGAATTTACTGACAACAGCTGAGCGATCCTCTGGTGAGTCTGTGATGAGGATCTC

6 TCGGATGAATTTACTGACAACAGCTGAGCGATCCTCTGGTGAGTCTGTGATGAGGATCTC

5 TCGGATGAATTTACTGACAACAGCTGAGCGATCCTCTGGTGAGTCTGTGATGAGGATCTC

4 TCGGATGAATTTACTGACAACAGCTGAGCGATCCTCTGGTGAGTCTGTGATGAGGATCTC

3 TCGGATGAATTTACTGACAACAGCTGAGCGATCCTCTGGTGAGTCTGTGATGAGGATCTC

20 TCGGATGAATTTACTGACAACAGCTGAGCGATCCTCTGGTGAGTCTGTGATGAGGATCTC

************************************************************

1 TGAAGCCACCCATCTCACTGTCATGTTGAATCTTGCTATCACGATCTTGATAGATGAGCC

16 TGAAGCCACCCATCTCACTGTCATGTTGAATCTTGCTATCACGATCTTGATAGATGAGCC

15 TGAAGCCACCCATCTCACTGTCATGTTGAATCTTGCTATCACGATCTTGATAGATGAGCC

2 TGAAGCCACCCATCTCACTGTCATGTTGAATCTTGCTATCACGATCTTGATAGATGAGCC

10 TGAAGCCACCCATCTCACTGTCATGTTGAATCTTGCTATCACGATCTTGATAGATGAGCC

17 TGAAGCCACCCATCTCACTGTCATGTTGAATCTTGCTATCACGATCTTGATAGATGAGCC

14 TGAAGCCACCCATCTCACTGTCATGTTGAATCTTGCTATCACGATCTTGATAGATGAGCC

13 TGAAGCCACCCATCTCACTGTCATGTTGAATCTTGCTATCACGATCTTGATAGATGAGCC

12 TGAAGCCACCCATCTCACTGTCATGTTGAATCTTGCTATCACGATCTTGATAGATGAGCC

8 TGAAGCCACCCATCTCACTGTCATGTTGAATCTTGCTATCACGATCTTGATAGATGAGCC

7 TGAAGCCACCCATCTCACTGTCATGTTGAATCTTGCTATCACGATCTTGATAGATGAGCC

19 TGAAGCCACCCATCTCACTGTCATGTTGAATCTTGCTATCACGATCTTGATAGATGAGCC

18 TGAAGCCACCCATCTCACTGTCATGTTGAATCTTGCTATCACGATCTTGATAGATGAGCC

22 TGAAGCCACCCATCTCACTGTCATGTTGAATCTTGCTATCACGATCTTGATAGATGAGCC

21 TGAAGCCACCCATCTCACTGTCATGTTGAATCTTGCTATCACGATCTTGATAGATGAGCC

9 TGAAGCCACCCATCTCACTGTCATGTTGAATCTTGCTATCACGATCTTGATAGATGAGCC

6 TGAAGCCACCCATCTCACTGTCATGTTGAATCTTGCTATCACGATCTTGATAGATGAGCC

5 TGAAGCCACCCATCTCACTGTCATGTTGAATCTTGCTATCACGATCTTGATAGATGAGCC

4 TGAAGCCACCCATCTCACTGTCATGTTGAATCTTGCTATCACGATCTTGATAGATGAGCC

3 TGAAGCCACCCATCTCACTGTCATGTTGAATCTTGCTATCACGATCTTGATAGATGAGCC

20 TGAAGCCACCCATCTCACTGTCATGTTGAATCTTGCTATCACGATCTTGATAGATGAGCC

************************************************************

1 GTGTAAGTTTTCATTACTGCTAACCTTCCTACGTGCTACTTCTTTCATGAATGATACCCA

16 GTGTAAGTTTTCATTACTGCTAACCTTCCTACGTGCTACTTCTTTCATGAATGATACCCA

15 GTGTAAGTTTTCATTACTGCTAACCTTCCTACGTGCTACTTCTTTCATGAATGATACCCA

2 GTGTAAGTTTTCATTACTGCTAACCTTCCTACGTGCTACTTCTTTCATGAATGATACCCA

10 GTGTAAGTTTTCATTACTGCTAACCTTCCTACGTGCTACTTCTTTCATGAATGATACCCA

17 GTGTAAGTTTTCATTACTGCTAACCTTCCTACGTGCTACTTCTTTCATGAATGATACCCA

14 GTGTAAGTTTTCATTACTGCTAACCTTCCTACGTGCTACTTCTTTCATGAATGATACCCA

13 GTGTAAGTTTTCATTACTGCTAACCTTCCTACGTGCTACTTCTTTCATGAATGATACCCA

12 GTGTAAGTTTTCATTACTGCTAACCTTCCTACGTGCTACTTCTTTCATGAATGATACCCA

8 GTGTAAGTTTTCATTACTGCTAACCTTCCTACGTGCTACTTCTTTCATGAATGATACCCA

7 GTGTAAGTTTTCATTACTGCTAACCTTCCTACGTGCTACTTCTTTCATGAATGATACCCA

19 GTGTAAGTTTTCATTACTGCTAACCTTCCTACGTGCTACTTCTTTCATGAATGATACCCA

18 GTGTAAGTTTTCATTACTGCTAACCTTCCTACGTGCTACTTCTTTCATGAATGATACCCA

22 GTGTAAGTTTTCATTACTGCTAACCTTCCTACGTGCTACTTCTTTCATGAATGATACCCA

21 GTGTAAGTTTTCATTACTGCTAACCTTCCTACGTGCTACTTCTTTCATGAATGATACCCA

9 GTGTAAGTTTTCATTACTGCTAACCTTCCTACGTGCTACTTCTTTCATGAATGATACCCA

6 GTGTAAGTTTTCATTACTGCTAACCTTCCTACGTGCTACTTCTTTCATGAATGATACCCA

5 GTGTAAGTTTTCATTACTGCTAACCTTCCTACGTGCTACTTCTTTCATGAATGATACCCA

4 GTGTAAGTTTTCATTACTGCTAACCTTCCTACGTGCTACTTCTTTCATGAATGATACCCA

3 GTGTAAGTTTTCATTACTGCTAACCTTCCTACGTGCTACTTCTTTCATGAATGATACCCA

20 GTGTAAGTTTTCATTACTGCTAACCTTCCTACGTGCTACTTCTTTCATGAATGATACCCA

************************************************************

1 ATTAATAATTGTGTCAAATTTAAGATACTCCTCCTTCCAATTGTCCAGCTTCTCTATATC

16 ATTAATAATTGTGTCAAATTTAAGATACTCCTCCTTCCAATTGTCCAGCTTCTCTATATC

15 ATTAATAATTGTGTCAAATTTAAGATACTCCTCCTTCCAATTGTCCAGCTTCTCTATATC

2 ATTAATAATTGTGTCAAATTTAAGATACTCCTCCTTCCAATTGTCCAGCTTCTCTATATC

10 ATTAATAATTGTGTCAAATTTAAGATACTCCTCCTTCCAATTGTCCAGCTTCTCTATATC

17 ATTAATAATTGTGTCAAATTTAAGATACTCCTCCTTCCAATTGTCCAGCTTCTCTATATC

14 ATTAATAATTGTGTCAAATTTAAGATACTCCTCCTTCCAATTGTCCAGCTTCTCTATATC

13 ATTAATAATTGTGTCAAATTTAAGATACTCCTCCTTCCAATTGTCCAGCTTCTCTATATC

12 ATTAATAATTGTGTCAAATTTAAGATACTCCTCCTTCCAATTGTCCAGCTTCTCTATATC

8 ATTAATAATTGTGTCAAATTTAAGATACTCCTCCTTCCAATTGTCCAGCTTCTCTATATC

7 ATTAATAATTGTGTCAAATTTAAGATACTCCTCCTTCCAATTGTCCAGCTTCTCTATATC

19 ATTAATAATTGTGTCAAATTTAAGATACTCCTCCTTCCAATTGTCCAGCTTCTCTATATC

18 ATTAATAATTGTGTCAAATTTAAGATACTCCTCCTTCCAATTGTCCAGCTTCTCTATATC

22 ATTAATAATTGTGTCAAATTTAAGATACTCCTCCTTCCAATTGTCCAGCTTCTCTATATC

21 ATTAATAATTGTGTCAAATTTAAGATACTCCTCCTTCCAATTGTCCAGCTTCTCTATATC

9 ATTAATAATTGTGTCAAATTTAAGATACTCCTCCTTCCAATTGTCCAGCTTCTCTATATC

6 ATTAATAATTGTGTCAAATTTAAGATACTCCTCCTTCCAATTGTCCAGCTTCTCTATATC

5 ATTAATAATTGTGTCAAATTTAAGATACTCCTCCTTCCAATTGTCCAGCTTCTCTATATC

4 ATTAATAATTGTGTCAAATTTAAGATACTCCTCCTTCCAATTGTCCAGCTTCTCTATATC

3 ATTAATAATTGTGTCAAATTTAAGATACTCCTCCTTCCAATTGTCCAGCTTCTCTATATC

20 ATTAATAATTGTGTCAAATTTAAGATACTCCTCCTTCCAATTGTCCAGCTTCTCTATATC

************************************************************

1 CTCGAACTTTATCTCGTTGAAAAGATCCTTCTCAATAAGGGAAAATTGTTTAGAGAGCTC

16 CTCGAACTTTATCTCGTTGAAAAGATCCTTCTCAATAAGGGAAAATTGTTTAGAGAGCTC

15 CTCGAACTTTATCTCGTTGAAAAGATCCTTCTCAATAAGGGAAAATTGTTTAGAGAGCTC

2 CTCGAACTTTATCTCGTTGAAAAGATCCTTCTCAATAAGGGAAAATTGTTTAGAGAGCTC

10 CTCGAACTTTATCTCGTTGAAAAGATCCTTCTCAATAAGGGAAAATTGTTTAGAGAGCTC

17 CTCGAACTTTATCTCGTTGAAAAGATCCTTCTCAATAAGGGAAAATTGTTTAGAGAGCTC

14 CTCGAACTTTATCTCGTTGAAAAGATCCTTCTCAATAAGGGAAAATTGTTTAGAGAGCTC

13 CTCGAACTTTATCTCGTTGAAAAGATCCTTCTCAATAAGGGAAAATTGTTTAGAGAGCTC

12 CTCGAACTTTATCTCGTTGAAAAGATCCTTCTCAATAAGGGAAAATTGTTTAGAGAGCTC

8 CTCGAACTTTATCTCGTTGAAAAGATCCTTCTCAATAAGGGAAAATTGTTTAGAGAGCTC

7 CTCGAACTTTATCTCGTTGAAAAGATCCTTCTCAATAAGGGAAAATTGTTTAGAGAGCTC

19 CTCGAACTTTATCTCGTTGAAAAGATCCTTCTCAATAAGGGAAAATTGTTTAGAGAGCTC

18 CTCGAACTTTATCTCGTTGAAAAGATCCTTCTCAATAAGGGAAAATTGTTTAGAGAGCTC

22 CTCGAACTTTATCTCGTTGAAAAGATCCTTCTCAATAAGGGAAAATTGTTTAGAGAGCTC

21 CTCGAACTTTATCTCGTTGAAAAGATCCTTCTCAATAAGGGAAAATTGTTTAGAGAGCTC

9 CTCGAACTTTATCTCGTTGAAAAGATCCTTCTCAATAAGGGAAAATTGTTTAGAGAGCTC

6 CTCGAACTTTATCTCGTTGAAAAGATCCTTCTCAATAAGGGAAAATTGTTTAGAGAGCTC

5 CTCGAACTTTATCTCGTTGAAAAGATCCTTCTCAATAAGGGAAAATTGTTTAGAGAGCTC

4 CTCGAACTTTATCTCGTTGAAAAGATCCTTCTCAATAAGGGAAAATTGTTTAGAGAGCTC

3 CTCGAACTTTATCTCGTTGAAAAGATCCTTCTCAATAAGGGAAAATTGTTTAGAGAGCTC

20 CTCGAACTTTATCTCGTTGAAAAGATCCTTCTCAATAAGGGAAAATTGTTTAGAGAGCTC

************************************************************

1 CTCCGTCTTGTAATCCAAAATCCACGATTTACACACAGATTTCTTTGTCGTGTTGTGGCT

16 CTCCGTCTTGTAATCCAAAATCCACGATTTACACACAGATTTCTTTGTCGTGTTGTGGCT

15 CTCCGTCTTGTAATCCAAAATCCACGATTTACACACAGATTTCTTTGTCGTGTTGTGGCT

2 CTCCGTCTTGTAATCCAAAATCCACGATTTACACACAGATTTCTTTGTCGTGTTGTGGCT

10 CTCCGTCTTGTAATCCAAAATCCACGATTTACACACAGATTTCTTTGTCGTGTTGTGGCT

17 CTCCGTCTTGTAATCCAAAATCCACGATTTACACACAGATTTCTTTGTCGTGTTGTGGCT

14 CTCCGTCTTGTAATCCAAAATCCACGATTTACACACAGATTTCTTTGTCGTGTTGTGGCT

13 CTCCGTCTTGTAATCCAAAATCCACGATTTACACACAGATTTCTTTGTCGTGTTGTGGCT

12 CTCCGTCTTGTAATCCAAAATCCACGATTTACACACAGATTTCTTTGTCGTGTTGTGGCT

8 CTCCGTCTTGTAATCCAAAATCCACGATTTACACACAGATTTCTTTGTCGTGTTGTGGCT

7 CTCCGTCTTGTAATCCAAAATCCACGATTTACACACAGATTTCTTTGTCGTGTTGTGGCT

19 CTCCGTCTTGTAATCCAAAATCCACGATTTACACACAGATTTCTTTGTCGTGTTGTGGCT

18 CTCCGTCTTGTAATCCAAAATCCACGATTTACACACAGATTTCTTTGTCGTGTTGTGGCT

22 CTCCGTCTTGTAATCCAAAATCCACGATTTACACACAGATTTCTTTGTCGTGTTGTGGCT

21 CTCCGTCTTGTAATCCAAAATCCACGATTTACACACAGATTTCTTTGTCGTGTTGTGGCT

9 CTCCGTCTTGTAATCCAAAATCCACGATTTACACACAGATTTCTTTGTCGTGTTGTGGCT

6 CTCCGTCTTGTAATCCAAAATCCACGATTTACACACAGATTTCTTTGTCGTGTTGTGGCT

5 CTCCGTCTTGTAATCCAAAATCCACGATTTACACACAGATTTCTTTGTCGTGTTGTGGCT

4 CTCCGTCTTGTAATCCAAAATCCACGATTTACACACAGATTTCTTTGTCGTGTTGTGGCT

3 CTCCGTCTTGTAATCCAAAATCCACGATTTACACACAGATTTCTTTGTCGTGTTGTGGCT

20 CTCCGTCTTGTAATCCAAAATCCACGATTTACACACAGATTTCTTTGTCGTGTTGTGGCT

************************************************************

1 ATCTATTATCGCGCTTGAGCCATGACTCAATCCTCTCTTTCTATTTATGGGTAGTGAAGG

16 ATCTATTATCGCGCTTGAGCCATGACTCAATCCTCTCTTTCTATTTATGGGTAGTGAAGG

15 ATCTATTATCGCGCTTGAGCCATGACTCAATCCTCTCTTTCTATTTATGGGTAGTGAAGG

2 ATCTATTATCGCGCTTGAGCCATGACTCAATCCTCTCTTTCTATTTATGGGTAGTGAAGG

10 ATCTATTATCGCGCTTGAGCCATGACTCAATCCTCTCTTTCTATTTATGGGTAGTGAAGG

17 ATCTATTATCGCGCTTGAGCCATGACTCAATCCTCTCTTTCTATTTATGGGTAGTGAAGG

14 ATCTATTATCGCGCTTGAGCCATGACTCAATCCTCTCTTTCTATTTATGGGTAGTGAAGG

13 ATCTATTATCGCGCTTGAGCCATGACTCAATCCTCTCTTTCTATTTATGGGTAGTGAAGG

12 ATCTATTATCGCGCTTGAGCCATGACTCAATCCTCTCTTTCTATTTATGGGTAGTGAAGG

8 ATCTATTATCGCGCTTGAGCCATGACTCAATCCTCTCTTTCTATTTATGGGTAGTGAAGG

7 ATCTATTATCGCGCTTGAGCCATGACTCAATCCTCTCTTTCTATTTATGGGTAGTGAAGG

19 ATCTATTATCGCGCTTGAGCCATGACTCAATCCTCTCTTTCTATTTATGGGTAGTGAAGG

18 ATCTATTATCGCGCTTGAGCCATGACTCAATCCTCTCTTTCTATTTATGGGTAGTGAAGG

22 ATCTATTATCGCGCTTGAGCCATGACTCAATCCTCTCTTTCTATTTATGGGTAGTGAAGG

21 ATCTATTATCGCGCTTGAGCCATGACTCAATCCTCTCTTTCTATTTATGGGTAGTGAAGG

9 ATCTATTATCGCGCTTGAGCCATGACTCAATCCTCTCTTTCTATTTATGGGTAGTGAAGG

6 ATCTATTATCGCGCTTGAGCCATGACTCAATCCTCTCTTTCTATTTATGGGTAGTGAAGG

5 ATCTATTATCGCGCTTGAGCCATGACTCAATCCTCTCTTTCTATTTATGGGTAGTGAAGG

4 ATCTATTATCGCGCTTGAGCCATGACTCAATCCTCTCTTTCTATTTATGGGTAGTGAAGG

3 ATCTATTATCGCGCTTGAGCCATGACTCAATCCTCTCTTTCTATTTATGGGTAGTGAAGG

20 ATCTATTATCGCGCTTGAGCCATGACTCAATCCTCTCTTTCTATTTATGGGTAGTGAAGG

************************************************************

1 TGAAGTGACTGCAACGGAGTGCGAACGAGGTTTTGGTTGTATGGTATTGCTAGTAAAACT

16 TGAAGTGACTGCAACGGAGTGCGAACGAGGTTTTGGTTGTATGGTATTGCTAGTAAAACT

15 TGAAGTGACTGCAACGGAGTGCGAACGAGGTTTTGGTTGTATGGTATTGCTAGTAAAACT

2 TGAAGTGACTGCAACGGAGTGCGAACGAGGTTTTGGTTGTATGGTATTGCTAGTAAAACT

10 TGAAGTGACTGCAACGGAGTGCGAACGAGGTTTTGGTTGTATGGTATTGCTAGTAAAACT

17 TGAAGTGACTGCAACGGAGTGCGAACGAGGTTTTGGTTGTATGGTATTGCTAGTAAAACT

14 TGAAGTGACTGCAACGGAGTGCGAACGAGGTTTTGGTTGTATGGTATTGCTAGTAAAACT

13 TGAAGTGACTGCAACGGAGTGCGAACGAGGTTTTGGTTGTATGGTATTGCTAGTAAAACT

12 TGAAGTGACTGCAACGGAGTGCGAACGAGGTTTTGGTTGTATGGTATTGCTAGTAAAACT

8 TGAAGTGACTGCAACGGAGTGCGAACGAGGTTTTGGTTGTATGGTATTGCTAGTAAAACT

7 TGAAGTGACTGCAACGGAGTGCGAACGAGGTTTTGGTTGTATGGTATTGCTAGTAAAACT

19 TGAAGTGACTGCAACGGAGTGCGAACGAGGTTTTGGTTGTATGGTATTGCTAGTAAAACT

18 TGAAGTGACTGCAACGGAGTGCGAACGAGGTTTTGGTTGTATGGTATTGCTAGTAAAACT

22 TGAAGTGACTGCAACGGAGTGCGAACGAGGTTTTGGTTGTATGGTATTGCTAGTAAAACT

21 TGAAGTGACTGCAACGGAGTGCGAACGAGGTTTTGGTTGTATGGTATTGCTAGTAAAACT

9 TGAAGTGACTGCAACGGAGTGCGAACGAGGTTTTGGTTGTATGGTATTGCTAGTAAAACT

6 TGAAGTGACTGCAACGGAGTGCGAACGAGGTTTTGGTTGTATGGTATTGCTAGTAAAACT

5 TGAAGTGACTGCAACGGAGTGCGAACGAGGTTTTGGTTGTATGGTATTGCTAGTAAAACT

4 TGAAGTGACTGCAACGGAGTGCGAACGAGGTTTTGGTTGTATGGTATTGCTAGTAAAACT

3 TGAAGTGACTGCAACGGAGTGCGAACGAGGTTTTGGTTGTATGGTATTGCTAGTAAAACT

20 TGAAGTGACTGCAACGGAGTGCGAACGAGGTTTTGGTTGTATGGTATTGCTAGTAAAACT

************************************************************

1 AGCACTTCCACCAAGCACGCTATTTGCGCGTATTATCGGTGATAAGTTGGGTGAAGAGTT

16 AGCACTTCCACCAAGCACGCTATTTGCGCGTATTATCGGTGATAAGTTGGGTGAAGAGTT

15 AGCACTTCCACCAAGCACGCTATTTGCGCGTATTATCGGTGATAAGTTGGGTGAAGAGTT

2 AGCACTTCCACCAAGCACGCTATTTGCGCGTATTATCGGTGATAAGTTGGGTGAAGAGTT

10 AGCACTTCCACCAAGCACGCTATTTGCGCGTATTATCGGTGATAAGTTGGGTGAAGAGTT

17 AGCACTTCCACCAAGCACGCTATTTGCGCGTATTATCGGTGATAAGTTGGGTGAAGAGTT

14 AGCACTTCCACCAAGCACGCTATTTGCGCGTATTATCGGTGATAAGTTGGGTGAAGAGTT

13 AGCACTTCCACCAAGCACGCTATTTGCGCGTATTATCGGTGATAAGTTGGGTGAAGAGTT

12 AGCACTTCCACCAAGCACGCTATTTGCGCGTATTATCGGTGATAAGTTGGGTGAAGAGTT

8 AGCACTTCCACCAAGCACGCTATTTGCGCGTATTATCGGTGATAAGTTGGGTGAAGAGTT

7 AGCACTTCCACCAAGCACGCTATTTGCGCGTATTATCGGTGATAAGTTGGGTGAAGAGTT

19 AGCACTTCCACCAAGCACGCTATTTGCGCGTATTATCGGTGATAAGTTGGGTGAAGAGTT

18 AGCACTTCCACCAAGCACGCTATTTGCGCGTATTATCGGTGATAAGTTGGGTGAAGAGTT

22 AGCACTTCCACCAAGCACGCTATTTGCGCGTATTATCGGTGATAAGTTGGGTGAAGAGTT

21 AGCACTTCCACCAAGCACGCTATTTGCGCGTATTATCGGTGATAAGTTGGGTGAAGAGTT

9 AGCACTTCCACCAAGCACGCTATTTGCGCGTATTATCGGTGATAAGTTGGGTGAAGAGTT

6 AGCACTTCCACCAAGCACGCTATTTGCGCGTATTATCGGTGATAAGTTGGGTGAAGAGTT

5 AGCACTTCCACCAAGCACGCTATTTGCGCGTATTATCGGTGATAAGTTGGGTGAAGAGTT

4 AGCACTTCCACCAAGCACGCTATTTGCGCGTATTATCGGTGATAAGTTGGGTGAAGAGTT

3 AGCACTTCCACCAAGCACGCTATTTGCGCGTATTATCGGTGATAAGTTGGGTGAAGAGTT

20 AGCACTTCCACCAAGCACGCTATTTGCGCGTATTATCGGTGATAAGTTGGGTGAAGAGTT

************************************************************

1 TTCACTTGCGTCGGTGCTAACTACACCCTGACTCTGACTCATTCTTTTCTTCTTGGACTT

16 TTCACTTGCGTCGGTGCTAACTACACCCTGACTCTGACTCATTCTTTTCTTCTTGGACTT

15 TTCACTTGCGTCGGTGCTAACTACACCCTGACTCTGACTCATTCTTTTCTTCTTGGACTT

2 TTCACTTGCGTCGGTGCTAACTACACCCTGACTCTGACTCATTCTTTTCTTCTTGGACTT

10 TTCACTTGCGTCGGTGCTAACTACACCCTGACTCTGACTCATTCTTTTCTTCTTGGACTT

17 TTCACTTGCGTCGGTGCTAACTACACCCTGACTCTGACTCATTCTTTTCTTCTTGGACTT

14 TTCACTTGCGTCGGTGCTAACTACACCCTGACTCTGACTCATTCTTTTCTTCTTGGACTT

13 TTCACTTGCGTCGGTGCTAACTACACCCTGACTCTGACTCATTCTTTTCTTCTTGGACTT

12 TTCACTTGCGTCGGTGCTAACTACACCCTGACTCTGACTCATTCTTTTCTTCTTGGACTT

8 TTCACTTGCGTCGGTGCTAACTACACCCTGACTCTGACTCATTCTTTTCTTCTTGGACTT

7 TTCACTTGCGTCGGTGCTAACTACACCCTGACTCTGACTCATTCTTTTCTTCTTGGACTT

19 TTCACTTGCGTCGGTGCTAACTACACCCTGACTCTGACTCATTCTTTTCTTCTTGGACTT

18 TTCACTTGCGTCGGTGCTAACTACACCCTGACTCTGACTCATTCTTTTCTTCTTGGACTT

22 TTCACTTGCGTCGGTGCTAACTACACCCTGACTCTGACTCATTCTTTTCTTCTTGGACTT

21 TTCACTTGCGTCGGTGCTAACTACACCCTGACTCTGACTCATTCTTTTCTTCTTGGACTT

9 TTCACTTGCGTCGGTGCTAACTACACCCTGACTCTGACTCATTCTTTTCTTCTTGGACTT

6 TTCACTTGCGTCGGTGCTAACTACACCCTGACTCTGACTCATTCTTTTCTTCTTGGACTT

5 TTCACTTGCGTCGGTGCTAACTACACCCTGACTCTGACTCATTCTTTTCTTCTTGGACTT

4 TTCACTTGCGTCGGTGCTAACTACACCCTGACTCTGACTCATTCTTTTCTTCTTGGACTT

3 TTCACTTGCGTCGGTGCTAACTACACCCTGACTCTGACTCATTCTTTTCTTCTTGGACTT

20 TTCACTTGCGTCGGTGCTAACTACACCCTGACTCTGACTCATTCTTTTCTTCTTGGACTT

************************************************************

1 TTGCATCATGGACTCGACTTTACGTAATTTTTGCTTTTGTTTCACTTTGTCGATGTGTCC

16 TTGCATCATGGACTCGACTTTACGTAATTTTTGCTTTTGTTTCACTTTGTCGATGTGTCC

15 TTGCATCATGGACTCGACTTTACGTAATTTTTGCTTTTGTTTCACTTTGTCGATGTGTCC

2 TTGCATCATGGACTCGACTTTACGTAATTTTTGCTTTTGTTTCACTTTGTCGATGTGTCC

10 TTGCATCATGGACTCGACTTTACGTAATTTTTGCTTTTGTTTCACTTTGTCGATGTGTCC

17 TTGCATCATGGACTCGACTTTACGTAATTTTTGCTTTTGTTTCACTTTGTCGATGTGTCC

14 TTGCATCATGGACTCGACTTTACGTAATTTTTGCTTTTGTTTCACTTTGTCGATGTGTCC

13 TTGCATCATGGACTCGACTTTACGTAATTTTTGCTTTTGTTTCACTTTGTCGATGTGTCC

12 TTGCATCATGGACTCGACTTTACGTAATTTTTGCTTTTGTTTCACTTTGTCGATGTGTCC

8 TTGCATCATGGACTCGACTTTACGTAATTTTTGCTTTTGTTTCACTTTGTCGATGTGTCC

7 TTGCATCATGGACTCGACTTTACGTAATTTTTGCTTTTGTTTCACTTTGTCGATGTGTCC

19 TTGCATCATGGACTCGACTTTACGTAATTTTTGCTTTTGTTTCACTTTGTCGATGTGTCC

18 TTGCATCATGGACTCGACTTTACGTAATTTTTGCTTTTGTTTCACTTTGTCGATGTGTCC

22 TTGCATCATGGACTCGACTTTACGTAATTTTTGCTTTTGTTTCACTTTGTCGATGTGTCC

21 TTGCATCATGGACTCGACTTTACGTAATTTTTGCTTTTGTTTCACTTTGTCGATGTGTCC

9 TTGCATCATGGACTCGACTTTACGTAATTTTTGCTTTTGTTTCACTTTGTCGATGTGTCC

6 TTGCATCATGGACTCGACTTTACGTAATTTTTGCTTTTGTTTCACTTTGTCGATGTGTCC

5 TTGCATCATGGACTCGACTTTACGTAATTTTTGCTTTTGTTTCACTTTGTCGATGTGTCC

4 TTGCATCATGGACTCGACTTTACGTAATTTTTGCTTTTGTTTCACTTTGTCGATGTGTCC

3 TTGCATCATGGACTCGACTTTACGTAATTTTTGCTTTTGTTTCACTTTGTCGATGTGTCC

20 TTGCATCATGGACTCGACTTTACGTAATTTTTGCTTTTGTTTCACTTTGTCGATGTGTCC

************************************************************

1 TTCCAACGCGCGTAACGCCGCATTCACATCTCCATCGTCTTCTTCATCAGATTCAAGCCC

16 TTCCAACGCGCGTAACGCCGCATTCACATCTCCATCGTCTTCTTCATCAGATTCAAGCCC

15 TTCCAACGCGCGTAACGCCGCATTCACATCTCCATCGTCTTCTTCATCAGATTCAAGCCC

2 TTCCAACGCGCGTAACGCCGCATTCACATCTCCATCGTCTTCTTCATCAGATTCAAGCCC

10 TTCCAACGCGCGTAACGCCGCATTCACATCTCCATCGTCTTCTTCATCAGATTCAAGCCC

17 TTCCAACGCGCGTAACGCCGCATTCACATCTCCATCGTCTTCTTCATCAGATTCAAGCCC

14 TTCCAACGCGCGTAACGCCGCATTCACATCTCCATCGTCTTCTTCATCAGATTCAAGCCC

13 TTCCAACGCGCGTAACGCCGCATTCACATCTCCATCGTCTTCTTCATCAGATTCAAGCCC

12 TTCCAACGCGCGTAACGCCGCATTCACATCTCCATCGTCTTCTTCATCAGATTCAAGCCC

8 TTCCAACGCGCGTAACGCCGCATTCACATCTCCATCGTCTTCTTCATCAGATTCAAGCCC

7 TTCCAACGCGCGTAACGCCGCATTCACATCTCCATCGTCTTCTTCATCAGATTCAAGCCC

19 TTCCAACGCGCGTAACGCCGCATTCACATCTCCATCGTCTTCTTCATCAGATTCAAGCCC

18 TTCCAACGCGCGTAACGCCGCATTCACATCTCCATCGTCTTCTTCATCAGATTCAAGCCC

22 TTCCAACGCGCGTAACGCCGCATTCACATCTCCATCGTCTTCTTCATCAGATTCAAGCCC

21 TTCCAACGCGCGTAACGCCGCATTCACATCTCCATCGTCTTCTTCATCAGATTCAAGCCC

9 TTCCAACGCGCGTAACGCCGCATTCACATCTCCATCGTCTTCTTCATCAGATTCAAGCCC

6 TTCCAACGCGCGTAACGCCGCATTCACATCTCCATCGTCTTCTTCATCAGATTCAAGCCC

5 TTCCAACGCGCGTAACGCCGCATTCACATCTCCATCGTCTTCTTCATCAGATTCAAGCCC

4 TTCCAACGCGCGTAACGCCGCATTCACATCTCCATCGTCTTCTTCATCAGATTCAAGCCC

3 TTCCAACGCGCGTAACGCCGCATTCACATCTCCATCGTCTTCTTCATCAGATTCAAGCCC

20 TTCCAACGCGCGTAACGCCGCATTCACATCTCCATCGTCTTCTTCATCAGATTCAAGCCC

************************************************************

1 CTCAATGGTGAATGTCTTCTGCCAATTATCCATATCTACTTGAGAATCTGTCATTGTTTC

16 CTCAATGGTGAATGTCTTCTGCCAATTATCCATATCTACTTGAGAATCTGTCATTGTTTC

15 CTCAATGGTGAATGTCTTCTGCCAATTATCCATATCTACTTGAGAATCTGTCATTGTTTC

2 CTCAATGGTGAATGTCTTCTGCCAATTATCCATATCTACTTGAGAATCTGTCATTGTTTC

10 CTCAATGGTGAATGTCTTCTGCCAATTATCCATATCTACTTGAGAATCTGTCATTGTTTC

17 CTCAATGGTGAATGTCTTCTGCCAATTATCCATATCTACTTGAGAATCTGTCATTGTTTC

14 CTCAATGGTGAATGTCTTCTGCCAATTATCCATATCTACTTGAGAATCTGTCATTGTTTC

13 CTCAATGGTGAATGTCTTCTGCCAATTATCCATATCTACTTGAGAATCTGTCATTGTTTC

12 CTCAATGGTGAATGTCTTCTGCCAATTATCCATATCTACTTGAGAATCTGTCATTGTTTC

8 CTCAATGGTGAATGTCTTCTGCCAATTATCCATATCTACTTGAGAATCTGTCATTGTTTC

7 CTCAATGGTGAATGTCTTCTGCCAATTATCCATATCTACTTGAGAATCTGTCATTGTTTC

19 CTCAATGGTGAATGTCTTCTGCCAATTATCCATATCTACTTGAGAATCTGTCATTGTTTC

18 CTCAATGGTGAATGTCTTCTGCCAATTATCCATATCTACTTGAGAATCTGTCATTGTTTC

22 CTCAATGGTGAATGTCTTCTGCCAATTATCCATATCTACTTGAGAATCTGTCATTGTTTC

21 CTCAATGGTGAATGTCTTCTGCCAATTATCCATATCTACTTGAGAATCTGTCATTGTTTC

9 CTCAATGGTGAATGTCTTCTGCCAATTATCCATATCTACTTGAGAATCTGTCATTGTTTC

6 CTCAATGGTGAATGTCTTCTGCCAATTATCCATATCTACTTGAGAATCTGTCATTGTTTC

5 CTCAATGGTGAATGTCTTCTGCCAATTATCCATATCTACTTGAGAATCTGTCATTGTTTC

4 CTCAATGGTGAATGTCTTCTGCCAATTATCCATATCTACTTGAGAATCTGTCATTGTTTC

3 CTCAATGGTGAATGTCTTCTGCCAATTATCCATATCTACTTGAGAATCTGTCATTGTTTC

20 CTCAATGGTGAATGTCTTCTGCCAATTATCCATATCTACTTGAGAATCTGTCATTGTTTC

************************************************************

1 GGAATGAGTGGTAAGTCGGGCTGCACCATCAGTATGTGGTAACTTCTTCCCTTTTACCGC

16 GGAATGAGTGGTAAGTCGGGCTGCACCATCAGTATGTGGTAACTTCTTCCCTTTTACCGC

15 GGAATGAGTGGTAAGTCGGGCTGCACCATCAGTATGTGGTAACTTCTTCCCTTTTACCGC

2 GGAATGAGTGGTAAGTCGGGCTGCACCATCAGTATGTGGTAACTTCTTCCCTTTTACCGC

10 GGAATGAGTGGTAAGTCGGGCTGCACCATCAGTATGTGGTAACTTCTTCCCTTTTACCGC

17 GGAATGAGTGGTAAGTCGAGCTGCACCATCAGTATGTGGTAACTTCTTCCCTTTTACCGC

14 GGAATGAGTGGTAAGTCGAGCTGCACCATCAGTATGTGGTAACTTCTTCCCTTTTACCGC

13 GGAATGAGTGGTAAGTCGAGCTGCACCATCAGTATGTGGTAACTTCTTCCCTTTTACCGC

12 GGAATGAGTGGTAAGTCGAGCTGCACCATCAGTATGTGGTAACTTCTTCCCTTTTACCGC

8 GGAATGAGTGGTAAGTCGAGCTGCACCATCAGTATGTGGTAACTTCTTCCCTTTTACCGC

7 GGAATGAGTGGTAAGTCGAGCTGCACCATCAGTATGTGGTAACTTCTTCCCTTTTACCGC

19 GGAATGAGTGGTAAGTCGAGCTGCACCATCAGTATGTGGTAACTTCTTCCCTTTTACCGC

18 GGAATGAGTGGTAAGTCGAGCTGCACCATCAGTATGTGGTAACTTCTTCCCTTTTACCGC

22 GGAATGAGTGGCAAGTCGGGCTGCACCATCAGTATGTGGTAACTTCTTCCCTTTTACCGC

21 GGAATGAGTGGTAAGTCGGGCTGCACCATCAGTATGTGGTAACTTCTTCCCTTTTACCGC

9 GGAATGAGTGGTAAGTCGGGCTGCACCATCAGTATGTGGTAACTTCTTCCCTTTTACCGC

6 GGAATGAGTGGTAAGTCGGGCTGCACCATCAGTATGTGGTAACTTCTTCCCTTTTACCGC

5 GGAATGAGTGGTAAGTCGGGCTGCACCATCAGTATGTGGTAACTTCTTCCCTTTTACCGC

4 GGAATGAGTGGTAAGTCGGGCTGCACCATCAGTATGTGGTAACTTCTTCCCTTTTACCGC

3 GGAATGAGTGGTAAGTCGGGCTGCACCATCAGTATGTGGTAACTTCTTCCCTTTTACCGC

20 GGAATGAGTGGTAAGTCGGGCTGCACCATCAGTATGTGGTAACTTCTTCCCTTTTACCGC

***********.******.*****************************************

1 TGAATCATCCACATCTTCATCCACCTCCTCATCACTATCAATATCGCTGTCTATATCATC

16 TGAATCATCCACATCTTCATCCACCTCCTCATCACTATCAATATCGCTGTCTATATCATC

15 TGAATCATCCACATCTTCATCCACCTCCTCATCACTATCAATATCGCTGTCTATATCATC

2 TGAATCATCCACATCTTCATCCACCTCCTCATCACTATCAATATCGCTGTCTATATCATC

10 TGAATCATCCACATCTTCATCCACCTCCTCATCACTATCAATATCGCTGTCTATATCATC

17 TGAATCATCCACATCTTCATCCACCTCCTCATCACTATCAATATCGCTGTCTATATCATC

14 TGAATCATCCACATCTTCATCCACCTCCTCATCACTATCAATATCGCTGTCTATATCATC

13 TGAATCATCCACATCTTCATCCACCTCCTCATCACTATCAATATCGCTGTCTATATCATC

12 TGAATCATCCACATCTTCATCCACCTCCTCATCACTATCAATATCGCTGTCTATATCATC

8 TGAATCATCCACATCTTCATCCACCTCCTCATCACTATCAATATCGCTGTCTATATCATC

7 TGAATCATCCACATCTTCATCCACCTCCTCATCACTATCAATATCGCTGTCTATATCATC

19 TGAATCATCCACATCTTCATCCACCTCCTCATCACTATCAATATCGCTGTCTATATCATC

18 TGAATCATCCACATCTTCATCCACCTCCTCATCACTATCAATATCGCTGTCTATATCATC

22 TGAATCATCCACATCTTCATCCACCTCCTCATCACTATCAATATCGCTGTCTATATCATC

21 TGAATCATCCACATCTTCATCCACCTCCTCATCACTATCAATATCGCTGTCTATATCATC

9 TGAATCATCCACATCTTCATCCACCTCCTCATCACTATCAATATCGCTGTCTATATCATC

6 TGAATCATCCACATCTTCATCCACCTCCTCATCACTATCAATATCGCTGTCTATATCATC

5 TGAATCATCCACATCTTCATCCACCTCCTCATCACTATCAATATCGCTGTCTATATCATC

4 TGAATCATCCACATCTTCATCCACCTCCTCATCACTATCAATATCGCTGTCTATATCATC

3 TGAATCATCCACATCTTCATCCACCTCCTCATCACTATCAATATCGCTGTCTATATCATC

20 TGAATCATCCACATCTTCATCCACCTCCTCATCACTATCAATATCGCTGTCTATATCATC

************************************************************

1 TAATTGTAAAAATTTGGTCATAATTGAGTTATTTTTGATTTTGTCATTACTTTCATCATA

16 TAATTGTAAAAATTTGGTCATAATTGAGTTATTTTTGATTTTGTCATTACTTTCATCATC

15 TAATTGTAAAAATTTGGTCATAATTGAGTTATTTTTGATTTTGTCATTACTTTCATCATC

2 TAATTGTAAAAATTTGGTCATAATTGAGTTATTTTTGATTTTGTCATTACTTTCATCATC

10 TAATTGTAAAAATTTGGTCATAATTGAGTTATTTTTGATTTTGTCATTACTTTCATCATC

17 TAATTGTAAAAATTTGGTCATAATTGAGTTATTTTTGATTTTGTCATTACTTTCATCATC

14 TAATTGTAAAAATTTGGTCATAATTGAGTTATTTTTGATTTTGTCATTACTTTCATCATC

13 TAATTGTAAAAATTTGGTCATAATTGAGTTATTTTTGATTTTGTCATTACTTTCATCATC

12 TAATTGTAAAAATTTGGTCATAATTGAGTTATTTTTGATTTTGTCATTACTTTCATCATC

8 TAATTGTAAAAATTTGGTCATAATTGAGTTATTTTTGATTTTGTCATTACTTTCATCATC

7 TAATTGTAAAAATTTGGTCATAATTGAGTTATTTTTGATTTTGTCATTACTTTCATCATC

19 TAATTGTAAAAATTTGGTCATAATTGAGTTATTTTTGATTTTGTCATTACTTTCATCATC

18 TAATTGTAAAAATTTGGTCATAATTGAGTTATTTTTGATTTTGTCATTACTTTCATCATC

22 TAATTGTAAAAATTTGGTCATAATTGAGTTATTTTTGATTTTGTCATTACTTTCATCATC

21 TAATTGTAAAAATTTGGTCATAATTGAGTTATTTTTGATTTTGTCATTACTTTCATCATC

9 TAATTGTAAAAATTTGGTCATAATTGAGTTATTTTTGATTTTGTCATTACTTTCATCATC

6 TAATTGTAAAAATTTGGTCATAATTGAGTTATTTTTGATTTTGTCATTACTTTCATCATC

5 TAATTGTAAAAATTTGGTCATAATTGAGTTATTTTTGATTTTGTCATTACTTTCATCATC

4 TAATTGTAAAAATTTGGTCATAATTGAGTTATTTTTGATTTTGTCATTACTTTCATCATC

3 TAATTGTAAAAATTTGGTCATAATTGAGTTATTTTTGATTTTGTCATTACTTTCATCATC

20 TAATTGTAAAAATTTGGTCATAATTGAGTTATTTTTGATTTTGTCATTACTTTCATCATC

***********************************************************

1 CTTGTCTGTATTCTTGGGTATAATATTGTTTACATCAATCTCCTGGAGTTGACAGAACTC

16 CTTGTCTGTATTCTTGGGTATAATATTGTTTACATCAATCTCCTGGAGTTGACAGAACTC

15 CTTGTCTGTATTCTTGGGTATAATATTGTTTACATCAATCTCCTGGAGTTGACAGAACTC

2 CTTGTCTGTATTCTTGGGTATAATATTGTTTACATCAATCTCCTGGAGTTGACAGAACTC

10 CTTGTCTGTATTCTTGGGTATAATATTGTTTACATCAATCTCCTGGAGTTGACAGAACTC

17 CTTGTCTGTATTCTTGGGTATAATATTGTTTACATCAATCTCCTGGAGTTGACAGAACTC

14 CTTGTCTGTATTCTTGGGTATAATATTGTTTACATCAATCTCCTGGAGTTGACAGAACTC

13 CTTGTCTGTATTCTTGGGTATAATATTGTTTACATCAATCTCCTGGAGTTGACAGAACTC

12 CTTGTCTGTATTCTTGGGTATAATATTGTTTACATCAATCTCCTGGAGTTGACAGAACTC

8 CTTGTCTGTATTCTTGGGTATAATATTGTTTACATCAATCTCCTGGAGTTGACAGAACTC

7 CTTGTCTGTATTCTTGGGTATAATATTGTTTACATCAATCTCCTGGAGTTGACAGAACTC

19 CTTGTCTGTATTCTTGGGTATAATATTGTTTACATCAATCTCCTGGAGTTGACAGAACTC

18 CTTGTCTGTATTCTTGGGTATAATATTGTTTACATCAATCTCCTGGAGTTGACAGAACTC

22 CTTGTCTGTATTCTTGGGTATAATATTGTTTACATCAATCTCCTGGAGTTGACAGAACTC

21 CTTGTCTGTATTCTTGGGTATAATATTGTTTACATCAATCTCCTGGAGTTGACAGAACTC

9 CTTGTCTGTATTCTTGGGTATAATATTGTTTACATCAATCTCCTGGAGTTGACAGAACTC

6 CTTGTCTGTATTCTTGGGTATAATATTGTTTACATCAATCTCCTGGAGTTGACAGAACTC

5 CTTGTCTGTATTCTTGGGTATAATATTGTTTACATCAATCTCCTGGAGTTGACAGAACTC

4 CTTGTCTGTATTCTTGGGTATAATATTGTTTACATCAATCTCCTGGAGTTGACAGAACTC

3 CTTGTCTGTATTCTTGGGTATAATATTGTTTACATCAATCTCCTGGAGTTGACAGAACTC

20 CTTGTCTGTATTCTTGGGTATAATATTGTTTACATCAATCTCCTGGAGTTGACAGAACTC

************************************************************

1 CTGAAACGATATCGTTACTGTGTACTCATCTCTGAATTGATCCTCATTGTTATCTTGATC

16 CTGAAACGATATCGTTACTGTGTACTCATCTCTGAATTGATCCTCATTGTTATCTTGATC

15 CTGAAACGATATCGTTACTGTGTACTCATCTCTGAATTGATCCTCATTGTTATCTTGATC

2 CTGAAACGATATCGTTACTGTGTACTCATCTCTGAATTGATCCTCATTGTTATCTTGATC

10 CTGAAACGATATCGTTACTGTGTACTCATCTCTGAATTGATCCTCATTGTTATCTTGATC

17 CTGAAACGATATCGTTACTGTGTACTCATCTCTGAATTGATCCTCATTGTTATCTTGATC

14 CTGAAACGATATCGTTACTGTGTACTCATCTCTGAATTGATCCTCATTGTTATCTTGATC

13 CTGAAACGATATCGTTACTGTGTACTCATCTCTGAATTGATCCTCATTGTTATCTTGATC

12 CTGAAACGATATCGTTACTGTGTACTCATCTCTGAATTGATCCTCATTGTTATCTTGATC

8 CTGAAACGATATCGTTACTGTGTACTCATCTCTGAATTGATCCTCATTGTTATCTTGATC

7 CTGAAACGATATCGTTACTGTGTACTCATCTCTGAATTGATCCTCATTGTTATCTTGATC

19 CTGAAACGATATCGTTACTGTGTACTCATCTCTGAATTGATCCTCATTGTTATCTTGATC

18 CTGAAACGATATCGTTACTGTGTACTCATCTCTGAATTGATCCTCATTGTTATCTTGATC

22 CTGAAACGATATCGTTACTGTGTACTCATCTCTGAATTGATCCTCATTGTTATCTTGATC

21 CTGAAACGATATCGTTACTGTGTACTCATCTCTGAATTGATCCTCATTGTTATCTTGATC

9 CTGAAACGATATCGTTACTGTGTACTCATCTCTGAATTGATCCTCATTGTTATCTTGATC

6 CTGAAACGATATCGTTACTGTGTACTCATCTCTGAATTGATCCTCATTGTTATCTTGATC

5 CTGAAACGATATCGTTACTGTGTACTCATCTCTGAATTGATCCTCATTGTTATCTTGATC

4 CTGAAACGATATCGTTACTGTGTACTCATCTCTGAATTGATCCTCATTGTTATCTTGATC

3 CTGAAACGATATCGTTACTGTGTACTCATCTCTGAATTGATCCTCATTGTTATCTTGATC

20 CTGAAACGATATCGTTACTGTGTACTCATCTCTGAATTGATCCTCATTGTTATCTTGATC

************************************************************

1 ACTATCGAATACCTGTGGCTTGTTGGACATTATACGCTTACCAATTTTGCCAATTTTGCC

16 ACTATCGAATACCTGTGGCTTGTTGGACATTATACGCTTACCAATTTTGCCAATTTTGCC

15 ACTATCGAATACCTGTGGCTTGTTGGACATTATACGCTTACCAATTTTGCCAATTTTGCC

2 ACTATCGAATACCTGTGGCTTGTTGGACATTATACGCTTACCAATTTTGCCAATTTTGCC

10 ACTATCGAATACCTGTGGCTTGTTGGACATTATACGCTTACCAATTTTGCCAATTTTGCC

17 ACTATCGAATACCTGTGGCTTGTTGGACATTATACGCTTACCAATTTTGCCAATTTTGCC

14 ACTATCGAATACCTGTGGCTTGTTGGACATTATACGCTTACCAATTTTGCCAATTTTGCC

13 ACTATCGAATACCTGTGGCTTGTTGGACATTATACGCTTACCAATTTTGCCAATTTTGCC

12 ACTATCGAATACCTGTGGCTTGTTGGACATTATACGCTTACCAATTTTGCCAATTTTGCC

8 ACTATCGAATACCTGTGGCTTGTTGGACATTATACGCTTACCAATTTTGCCAATTTTGCC

7 ACTATCGAATACCTGTGGCTTGTTGGACATTATACGCTTACCAATTTTGCCAATTTTGCC

19 ACTATCGAATACCTGTGGCTTGTTGGACATTATACGCTTACCAATTTTGCCAATTTTGCC

18 ACTATCGAATACCTGTGGCTTGTTGGACATTATACGCTTACCAATTTTGCCAATTTTGCC

22 ACTATCGAATACCTGTGGCTTGTTGGACATTATACGCTTACCAATTTTGCCAATTTTGCC

21 ACTATCGAATACCTGTGGCTTGTTGGACATTATACGCTTACCAATTTTGCCAATTTTGCC

9 ACTATCGAATACCTGTGGCTTGTTGGACATTATACGCTTACCAATTTTGCCAATTTTGCC

6 ACTATCGAATACCTGTGGCTTGTTGGACATTATACGCTTACCAATTTTGCCAATTTTGCC

5 ACTATCGAATACCTGTGGCTTGTTGGACATTATACGCTTACCAATTTTGCCAATTTTGCC

4 ACTATCGAATACCTGTGGCTTGTTGGACATTATACGCTTACCAATTTTGCCAATTTTGCC

3 ACTATCGAATACCTGTGGCTTGTTGGACATTATACGCTTACCAATTTTGCCAATTTTGCC

20 ACTATCGAATACCTGTGGCTTGTTGGACATTATACGCTTACCAATTTTGCCAATTTTGCC

************************************************************

1 GACTTTGTTAAACGTTTTGACGAATGTGTCAGCAATTGAATCGTTATGCTCTGATGCATT

16 GACTTTGTTAAACGTTTTGATGAATGTGTCAGCAATTGAATCGTTATGCTCTGATGCATT

15 GACTTTGTTAAACGTTTTGACGAATGTGTCAGCAATTGAATCGTTATGCTCTGATGCATT

2 GACTTTGTTAAACGTTTTGACGAATGTGTCAGCAATTGAATCGTTATGCTCTGATGCATT

10 GACTTTGTTAAACGTTTTGACGAATGTGTCAGCAATTGAATCGTTATGCTCTGATGCATT

17 GACTTTGTTAAACGTTTTGACGAATGTGTCAGCAATTGAATCGTTATGCTCTGATGCATT

14 GACTTTGTTAAACGTTTTGACGAATGTGTCAGCAATTGAATCGTTATGCTCTGATGCATT

13 GACTTTGTTAAACGTTTTGACGAATGTGTCAGCAATTGAATCGTTATGCTCTGATGCATT

12 GACTTTGTTAAACGTTTTGACGAATGTGTCAGCAATTGAATCGTTATGCTCTGATGCATT

8 GACTTTGTTAAACGTTTTGACGAATGTGTCAGCAATTGAATCGTTATGCTCTGATGCATT

7 GACTTTGTTAAACGTTTTGACGAATGTGTCAGCAATTGAATCGTTATGCTCTGATGCATT

19 GACTTTGTTAAACGTTTTGACGAATGTGTCAGCAATTGAATCGTTATGCTCTGATGCATT

18 GACTTTGTTAAACGTTTTGACGAATGTGTCAGCAATTGAATCGTTATGCTCTGATGCATT

22 GACTTTGTTAAACGTTTTGACGAATGTGTCAGCAATTGAATCGTTATGCTCTGATGCATT

21 GACTTTGTTAAACGTTTTGACGAATGTGTCAGCAATTGAATCGTTATGCTCTGATGCATT

9 GACTTTGTTAAACGTTTTGACGAATGTGTCAGCAATTGAATCGTTATGCTCTGATGCATT

6 GACTTTGTTAAACGTTTTGACGAATGTGTCAGCAATTGAATCGTTATGCTCTGATGCATT

5 GACTTTGTTAAACGTTTTGACGAATGTGTCAGCAATTGAATCGTTATGCTCTGATGCATT

4 GACTTTGTTAAACGTTTTGACGAATGTGTCAGCAATTGAATCGTTATGCTCTGATGCATT

3 GACTTTGTTAAACGTTTTGACGAATGTGTCAGCAATTGAATCGTTATGCTCTGATGCATT

20 GACTTTGTTAAACGTTTTGACGAATGTGTCAGCAATTGAATCGTTATGCTCTGATGCATT

********************.***************************************

1 TACCGGTGATGACGTTGATGGTGCTGTGCTCGCTGTCATTCTAGACGATGTGGAAGAGGG

16 TACCGGTGATGACGTTGATGGTGCTGTGCTCGCTGTCATTCTAGACGATGTGGAAGAGGG

15 TACCGGTGATGACGTTGATGGTGCTGTGCTCGCTGTCATTCTAGACGATGTGGAAGAGGG

2 TACCGGTGATGACGTTGATGGTGCTGTGCTCGCTGTCATTCTAGACGATGTGGAAGAGGG

10 TACCGGTGATGACGTTGATGGTGCTGTGCTCGCTGTCATTCTAGACGATGTGGAAGAGGG

17 TACCGGTGATGACGTTGATGGTGCTGTGCTCGCTGTCATTCTAGACGATGTGGAAGAGGG

14 TACCGGTGATGACGTTGATGGTGCTGTGCTCGCTGTCATTCTAGACGATGTGGAAGAGGG

13 TACCGGTGATGACGTTGATGGTGCTGTGCTCGCTGTCATTCTAGACGATGTGGAAGAGGG

12 TACCGGTGATGACGTTGATGGTGCTGTGCTCGCTGTCATTCTAGACGATGTGGAAGAGGG

8 TACCGGTGATGACGTTGATGGTGCTGTGCTCGCTGTCATTCTAGACGATGTGGAAGAGGG

7 TACCGGTGATGACGTTGATGGTGCTGTGCTCGCTGTCATTCTAGACGATGTGGAAGAGGG

19 TACCGGTGATGACGTTGATGGTGCTGTGCTCGCTGTCATTCTAGACGATGTGGAAGAGGG

18 TACCGGTGATGACGTTGATGGTGCTGTGCTCGCTGTCATTCTAGACGATGTGGAAGAGGG

22 TACCGGTGATGACGTTGATGGTGCTGTGCTCGCTGTCATTCTAGACGATGTGGAAGAGGG

21 TACCGGTGATGACGTTGATGGTGCTGTGCTCGCTGTCATTCTAGACGATGTGGAAGAGGG

9 TACCGGTGATGACGTTGATGGTGCTGTGCTCGCTGTCATTCTAGACGATGTGGAAGAGGG

6 TACCGGTGATGACGTTGATGGTGCTGTGCTCGCTGTCATTCTAGACGATGTGGAAGAGGG

5 TACCGGTGATGACGTTGATGGTGCTGTGCTCGCTGTCATTCTAGACGATGTGGAAGAGGG

4 TACCGGTGATGACGTTGATGGTGCTGTGCTCGCTGTCATTCTAGACGATGTGGAAGAGGG

3 TACCGGTGATGACGTTGATGGTGCTGTGCTCGCTGTCATTCTAGACGATGTGGAAGAGGG

20 TACCGGTGATGACGTTGATGGTGCTGTGCTCGCTGTCATTCTAGACGATGTGGAAGAGGG

************************************************************

1 AAGAGAGGTTGGTGGACCTGGGTCAAAGTCCAGACTGAGGTGTTTGTCGGTGCTGGAATT

16 AAGAGAGGTTGGTGGACCTGGGTCAAAGTCCAGACTGAGGTGTTTGTCGGTGCTGGAATT

15 AAGAGAGGTTGGTGGACCTGGGTCAAAGTCCAGACTGAGGTGTTTGTCGGTGCTGGAATT

2 AAGAGAGGTTGGTGGACCTGGGTCAAAGTCCAGACTGAGGTGTTTGTCGGTGCTGGAATT

10 AAGAGAGGTTGGTGGACCTGGGTCAAAGTCCAGACTGAGGTGTTTGTCGGTGCTGGAATT

17 AAGATAGGTTGGTGGACCTGGGTCAAAGTCCAGACTGAGGTGTTTGTCGGTGCTGGAATT

14 AAGATAGGTTGGTGGACCTGGGTCAAAGTCCAGACTGAGGTGTTTGTCGGTGCTGGAATT

13 AAGATAGGTTGGTGGACCTGGGTCAAAGTCCAGACTGAGGTGTTTGTCGGTGCTGGAATT

12 AAGATAGGTTGGTGGACCTGGGTCAAAGTCCAGACTGAGGTGTTTGTCGGTGCTGGAATT

8 AAGATAGGTTGGTGGACCTGGGTCAAAGTCCAGACTGAGGTGTTTGTCGGTGCTGGAATT

7 AAGATAGGTTGGTGGACCTGGGTCAAAGTCCAGACTGAGGTGTTTGTCGGTGCTGGAATT

19 AAGATAGGTTGGTGGACCTGGGTCAAAGTCCAGACTGAGGTGTTTGTCGGTGCTGGAATT

18 AAGATAGGTTGGTGGACCTGGGTCAAAGTCCAGACTGAGGTGTTTGTCGGTGCTGGAATT

22 AAGAGAGGTTGGTGGACCTGGGTCAAAGTCCAGACTGAGGTGTTTGTCGGTGCTGGAATT

21 AAGAGAGGTTGGTGGACCTGGGTCAAAGTCCAGACTGAGGTGTTTGTCGGTGCTGGAATT

9 AAGAGAGGTTGGTGGACCTGGGTCAAAGTCCAGACTGAGGTGTTTGTCGGTGCTGGAATT

6 AAGAGAGGTTGGTGGACCTGGGTCAAAGTCCAGACTGAGGTGTTTGTCGGTGCTGGAATT

5 AAGAGAGGTTGGTGGACCTGGGTCAAAGTCCAGACTGAGGTGTTTGTCGGTGCTGGAATT

4 AAGAGAGGTTGGTGGACCTGGGTCAAAGTCCAGACTGAGGTGTTTGTCGGTGCTGGAATT

3 AAGAGAGGTTGGTGGACCTGGGTCAAAGTCCAGACTGAGGTGTTTGTCGGTGCTGGAATT

20 AAGAGAGGTTGGTGGACCTGGGTCAAAGTCCAGACTGAGGTGTTTGTCGGTGCTGGAATT

**** *******************************************************

1 TCTGCTGGAATTGCTACTATCAAACGACGACAAATTACCATCATGCGAATGTTTCTTGTA

16 TCTGCTGGAATTGCTACTATCAAACGACGACAAATTACCATCATGCGAATGTTTCTTGTA

15 TCTGCTGGAATTGCTACTATCAAACGACGACAAATTACCATCATGCGAATGTTTCTTGTA

2 TCTGCTGGAATTGCTACTATCAAACGACGACAAATTACCATCATGCGAATGTTTCTTGTA

10 TCTGCTGGAATTGCTACTATCAAACGACGACAAATTACCATCATGCGAATGTTTCTTGTA

17 TCTGCTGGAATTGCTACTATCAAACGACGACAAATTACCATCATGCGAATGTTTCTTGTA

14 TCTGCTGGAATTGCTACTATCAAACGACGACAAATTACCATCATGCGAATGTTTCTTGTA

13 TCTGCTGGAATTGCTACTATCAAACGACGACAAATTACCATCATGCGAATGTTTCTTGTA

12 TCTGCTGGAATTGCTACTATCAAACGACGACAAATTACCATCATGCGAATGTTTCTTGTA

8 TCTGCTGGAATTGCTACTATCAAACGACGACAAATTACCATCATGCGAATGTTTCTTGTA

7 TCTGCTGGAATTGCTACTATCAAACGACGACAAATTACCATCATGCGAATGTTTCTTGTA

19 TCTGCTGGAATTGCTACTATCAAACGACGACAAATTACCATCATGCGAATGTTTCTTGTA

18 TCTGCTGGAATTGCTACTATCAAACGACGACAAATTACCATCATGCGAATGTTTCTTGTA

22 TCTGCTGGAATTGCTACTATCAAACGACGACAAATTACCATCATGCGAATGTTTCTTGTA

21 TCTGCTGGAATTGCTACTATCAAACGACGACAAATTACCATCATGCGAATGTTTCTTGTA

9 TCTGCTGGAATTGCTACTATCAAACGACGACAAATTACCATCATGCGAATGTTTCTTGTA

6 TCTGCTGGAATTGCTACTATCAAACGACGACAAATTACCATCATGCGAATGTTTCTTGTA

5 TCTGCTGGAATTGCTACTATCAAACGACGACAAATTACCATCATGCGAATGTTTCTTGTA

4 TCTGCTGGAATTGCTACTATCAAACGACGACAAATTACCATCATGCGAATGTTTCTTGTA

3 TCTGCTGGAATTGCTACTATCAAACGACGACAAATTACCATCATGCGAATGTTTCTTGTA

20 TCTGCTGGAATTGCTACTATCAAACGACGACAAATTACCATCATGCGAATGTTTCTTGTA

************************************************************

1 AATTTCATAAACACTTTGACGCTCCTTGGCTATATTGATGAGCTTATCGATCATCTTTAC

16 AATTTCATAAACACTTTGACGCTCCTTGGCTATATTGATGAGCTTATCGATCATCTTTAC

15 AATTTCATAAACACTTTGACGCTCCTTGGCTATATTGATGAGCTTATCGATCATCTTTAC

2 AATTTCATAAACACTTTGACGCTCCTTGGCTATATTGATGAGCTTATCGATCATCTTTAC

10 AATTTCATAAACACTTTGACGCTCCTTGGCTATATTGATGAGCTTATCGATCATCTTTAC

17 AATTTCATAAACACTTTGACGCTCCTTGGCTATATTGATGAGCTTATCGATCATCTTTAC

14 AATTTCATAAACACTTTGACGCTCCTTGGCTATATTGATGAGCTTATCGATCATCTTTAC

13 AATTTCATAAACACTTTGACGCTCCTTGGCTATATTGATGAGCTTATCGATCATCTTTAC

12 AATTTCATAAACACTTTGACGCTCCTTGGCTATATTGATGAGCTTATCGATCATCTTTAC

8 AATTTCATAAACACTTTGACGCTCCTTGGCTATATTGATGAGCTTATCGATCATCTTTAC

7 AATTTCATAAACACTTTGACGCTCCTTGGCTATATTGATGAGCTTATCGATCATCTTTAC

19 AATTTCATAAACACTTTGACGCTCCTTGGCTATATTGATGAGCTTATCGATCATCTTTAC

18 AATTTCATAAACACTTTGACGCTCCTTGGCTATATTGATGAGCTTATCGATCATCTTTAC

22 AATTTCATAAACACTTTGACGCTCCTTGGCTATATTGATGAGCTTATCGATCATCTTTAC

21 AATTTCATAAACACTTTGACGCTCCTTGGCTATATTGATGAGCTTATCGATCATCTTTAC

9 AATTTCATAAACACTTTGACGCTCCTTGGCTATATTGATGAGCTTATCGATCATCTTTAC

6 AATTTCATAAACACTTTGACGCTCCTTGGCTATATTGATGAGCTTATCGATCATCTTTAC

5 AATTTCATAAACACTTTGACGCTCCTTGGCTATATTGATGAGCTTATCGATCATCTTTAC

4 AATTTCATAAACACTTTGACGCTCCTTGGCTATATTGATGAGCTTATCGATCATCTTTAC

3 AATTTCATAAACACTTTGACGCTCCTTGGCTATATTGATGAGCTTATCGATCATCTTTAC

20 AATTTCATAAACACTTTGACGCTCCTTGGCTATATTGATGAGCTTATCGATCATCTTTAC

************************************************************

1 GGACTTGTCATTCTGTATCTTCCTCTGTTCATTAATCCATCGTGACAACTTAAGACGGAG

16 GGACTTGTCATTCTGTATCTTCCTCTGTTCATTAATCCATCGTGACAACTTAAGACGGAG

15 GGACTTGTCATTCTGTATCTTCCTCTGTTCATTAATCCATCGTGACAACTTAAGACGGAG

2 GGACTTGTCATTCTGTATCTTCCTCTGTTCATTAATCCATCGTGACAACTTAAGACGGAG

10 GGACTTGTCATTCTGTATCTTCCTCTGTTCATTAATCCATCGTGACAACTTAAGACGGAG

17 GGACTTGTCATTCTGTATCTTCCTCTGTTCATTAATCCATCGTGACAACTTAAGACGGAG

14 GGACTTGTCATTCTGTATCTTCCTCTGTTCATTAATCCATCGTGACAACTTAAGACGGAG

13 GGACTTGTCATTCTGTATCTTCCTCTGTTCATTAATCCATCGTGACAACTTAAGACGGAG

12 GGACTTGTCATTCTGTATCTTCCTCTGTTCATTAATCCATCGTGACAACTTAAGACGGAG

8 GGACTTGTCATTCTGTATCTTCCTCTGTTCATTAATCCATCGTGACAACTTAAGACGGAG

7 GGACTTGTCATTCTGTATCTTCCTCTGTTCATTAATCCATCGTGACAACTTAAGACGGAG

19 GGACTTGTCATTCTGTATCTTCCTCTGTTCATTAATCCATCGTGACAACTTAAGACGGAG

18 GGACTTGTCATTCTGTATCTTCCTCTGTTCATTAATCCATCGTGACAACTTAAGACGGAG

22 GGACTTGTCATTCTGTATCTTCCTCTGTTCATTAATCCATCGTGACAACTTAAGACGGAG

21 GGACTTGTCATTCTGTATCTTCCTCTGTTCATTAATCCATCGTGACAACTTAAGACGGAG

9 GGACTTGTCATTCTGTATCTTCCTCTGTTCATTAATCCATCGTGACAACTTAAGACGGAG

6 GGACTTGTCATTCTGTATCTTCCTCTGTTCATTAATCCATCGTGACAACTTAAGACGGAG

5 GGACTTGTCATTCTGTATCTTCCTCTGTTCATTAATCCATCGTGACAACTTAAGACGGAG

4 GGACTTGTCATTCTGTATCTTCCTCTGTTCATTAATCCATCGTGACAACTTAAGACGGAG

3 GGACTTGTCATTCTGTATCTTCCTCTGTTCATTAATCCATCGTGACAACTTAAGACGGAG

20 GGACTTGTCATTCTGTATCTTCCTCTGTTCATTAATCCATCGTGACAACTTAAGACGGAG

************************************************************

1 GTCTAAGTTGGGCACGAAATCAAAGCCAAAGTAGTTGGAGATCCATGATCGGAGCATGAC

16 GTCTAAGTTGGGCACGAAATCAAAGCCAAAGTAGTTGGAGATCCATGATCGGAGCATGAC

15 GTCTAAGTTGGGCACGAAATCAAAGCCAAAGTAGTTGGAGATCCATGATCGGAGCATGAC

2 GTCTAAGTTGGGCACGAAATCAAAGCCAAAGTAGTTGGAGATCCATGATCGGAGCATGAC

10 GTCTAAGTTGGGCACGAAATCAAAGCCAAAGTAGTTGGAGATCCATGATCGGAGCATGAC

17 GTCTAAGTTGGGCACGAAATCAAAGCCAAAGTAGTTGGAGATCCATGATCGGAGCATGAC

14 GTCTAAGTTGGGCACGAAATCAAAGCCAAAGTAGTTGGAGATCCATGATCGGAGCATGAC

13 GTCTAAGTTGGGCACGAAATCAAAGCCAAAGTAGTTGGAGATCCATGATCGGAGCATGAC

12 GTCTAAGTTGGGCACGAAATCAAAGCCAAAGTAGTTGGAGATCCATGATCGGAGCATGAC

8 GTCTAAGTTGGGCACGAAATCAAAGCCAAAGTAGTTGGAGATCCATGATCGGAGCATGAC

7 GTCTAAGTTGGGCACGAAATCAAAGCCAAAGTAGTTGGAGATCCATGATCGGAGCATGAC

19 GTCTAAGTTGGGCACGAAATCAAAGCCAAAGTAGTTGGAGATCCATGATCGGAGCATGAC

18 GTCTAAGTTGGGCACGAAATCAAAGCCAAAGTAGTTGGAGATCCATGATCGGAGCATGAC

22 GTCTAAGTTGGGCACGAAATCAAAGCCAAAGTAGTTGGAGATCCATGATCGGAGCATGAC

21 GTCTAAGTTGGGCACGAAATCAAAGCCAAAGTAGTTGGAGATCCATGATCGGAGCATGAC

9 GTCTAAGTTGGGCACGAAATCAAAGCCAAAGTAGTTGGAGATCCATGATCGGAGCATGAC

6 GTCTAAGTTGGGCACGAAATCAAAGCCAAAGTAGTTGGAGATCCATGATCGGAGCATGAC

5 GTCTAAGTTGGGCACGAAATCAAAGCCAAAGTAGTTGGAGATCCATGATCGGAGCATGAC

4 GTCTAAGTTGGGCACGAAATCAAAGCCAAAGTAGTTGGAGATCCATGATCGGAGCATGAC

3 GTCTAAGTTGGGCACGAAATCAAAGCCAAAGTAGTTGGAGATCCATGATCGGAGCATGAC

20 GTCTAAGTTGGGCACGAAATCAAAGCCAAAGTAGTTGGAGATCCATGATCGGAGCATGAC

************************************************************

1 AAATGTCCTCACCCTGACTAGTTCTAAAGCAGGCTCATCATCACCACCCTGCAGCGTCCA

16 AAATGTCCTCACCCTGACTAGTTCTAAAGCAGGCTCATCATCACCACCCTGCAGCGTCCA

15 AAATGTCCTCACCCTGACTAGTTCTAAAGCAGGCTCATCATCACCACCCTGCAGCGTCCA

2 AAATGTCCTCACCCTGACTAGTTCTAAAGCAGGCTCATCATCACCACCCTGCAGCGTCCA

10 AAATGTCCTCACCCTGACTAGTTCTAAAGCAGGCTCATCATCACCACCCTGCAGCGTCCA

17 AAATGTCCTCACCCTGACTAGTTCTAAAGCAGGCTCATCATCACCACCCTGCAGCGTCCA

14 AAATGTCCTCACCCTGACTAGTTCTAAAGCAGGCTCATCATCACCACCCTGCAGCGTCCA

13 AAATGTCCTCACCCTGACTAGTTCTAAAGCAGGCTCATCATCACCACCCTGCAGCGTCCA

12 AAATGTCCTCACCCTGACTAGTTCTAAAGCAGGCTCATCATCACCACCCTGCAGCGTCCA

8 AAATGTCCTCACCCTGACTAGTTCTAAAGCAGGCTCATCATCACCACCCTGCAGCGTCCA

7 AAATGTCCTCACCCTGACTAGTTCTAAAGCAGGCTCATCATCACCACCCTGCAGCGTCCA

19 AAATGTCCTCACCCTGACTAGTTCTAAAGCAGGCTCATCATCACCACCCTGCAGCGTCCA

18 AAATGTCCTCACCCTGACTAGTTCTAAAGCAGGCTCATCATCACCACCCTGCAGCGTCCA

22 AAATGTCCTCACCCTGACTAGTTCTAAAGCAGGCTCATCATCACCACCCTGCAGCGTCCA

21 AAATGTCCTCACCCTGACTAGTTCTAAAGCAGGCTCATCATCACCACCCTGCAGCGTCCA

9 AAATGTCCTCACCCTGACTAGTTCTAAAGCAGGCTCATCATCACCACCCTGCAGCGTCCA

6 AAATGTCCTCACCCTGACTAGTTCTAAAGCAGGCTCATCATCACCACCCTGCAGCGTCCA

5 AAATGTCCTCACCCTGACTAGTTCTAAAGCAGGCTCATCATCACCACCCTGCAGCGTCCA

4 AAATGTCCTCACCCTGACTAGTTCTAAAGCAGGCTCATCATCACCACCCTGCAGCGTCCA

3 AAATGTCCTCACCCTGACTAGTTCTAAAGCAGGCTCATCATCACCACCCTGCAGCGTCCA

20 AAATGTCCTCACCCTGACTAGTTCTAAAGCAGGCTCATCATCACCACCCTGCAGCGTCCA

************************************************************

1 CTCGAATCTCTTAAATAATAAGTTGGCCAACTCCATCGAGCTGAGGTATAGTCTATAAGT

16 CTCGAATCTCTTAAATAATAAGTTGGCCAACTCCATCGAGCTGAGGTATAGTCTATAAGT

15 CTCGAATCTCTTAAATAATAAGTTGGCCAACTCCATCGAGCTGAGGTATAGTCTATAAGT

2 CTCGAATCTCTTAAATAATAAGTTGGCCAACTCCATCGAGCTGAGGTATAGTCTATAAGT

10 CTCGAATCTCTTAAATAATAAGTTGGCCAACTCCATCGAGCTGAGGTATAGTCTATAAGT

17 CTCGAATCTCTTAAATAATAAGTTGGCCAACTCCATCGAGCTGAGGTATAGTCTATAAGT

14 CTCGAATCTCTTAAATAATAAGTTGGCCAACTCCATCGAGCTGAGGTATAGTCTATAAGT

13 CTCGAATCTCTTAAATAATAAGTTGGCCAACTCCATCGAGCTGAGGTATAGTCTATAAGT

12 CTCGAATCTCTTAAATAATAAGTTGGCCAACTCCATCGAGCTGAGGTATAGTCTATAAGT

8 CTCGAATCTCTTAAATAATAAGTTGGCCAACTCCATCGAGCTGAGGTATAGTCTATAAGT

7 CTCGAATCTCTTAAATAATAAGTTGGCCAACTCCATCGAGCTGAGGTATAGTCTATAAGT

19 CTCGAATCTCTTAAATAATAAGTTGGCCAACTCCATCGAGCTGAGGTATAGTCTATAAGT

18 CTCGAATCTCTTAAATAATAAGTTGGCCAACTCCATCGAGCTGAGGTATAGTCTATAAGT

22 CTCGAATCTCTTAAATAATAAGTTGGCCAACTCCATCGAGCTGAGGTATAGTCTATAAGT

21 CTCGAATCTCTTAAATAATAAGTTGGCCAACTCCATCGAGCTGAGGTATAGTCTATAAGT

9 CTCGAATCTCTTAAATAATAAGTTGGCCAACTCCATCGAGCTGAGGTATAGTCTATAAGT

6 CTCGAATCTCTTAAATAATAAGTTGGCCAACTCCATCGAGCTGAGGTATAGTCTATAAGT

5 CTCGAATCTCTTAAATAATAAGTTGGCCAACTCCATCGAGCTGAGGTATAGTCTATAAGT

4 CTCGAATCTCTTAAATAATAAGTTGGCCAACTCCATCGAGCTGAGGTATAGTCTATAAGT

3 CTCGAATCTCTTAAATAATAAGTTGGCCAACTCCATCGAGCTGAGGTATAGTCTATAAGT

20 CTCGAATCTCTTAAATAATAAGTTGGCCAACTCCATCGAGCTGAGGTATAGTCTATAAGT

************************************************************

1 GTTGAAAAATAATTCTAAATCGTCAAATAAGAACTCTGAGCTAAGTTGAGAAATCCATCT

16 GTTGAAAAATAATTCTAAATCGTCAAATAAGAACTCTGAGCTAAGTTGAGAAATCCATCT

15 GTTGAAAAATAATTCTAAATCGTCAAATAAGAACTCTGAGCTAAGTTGAGAAATCCATCT

2 GTTGAAAAATAATTCTAAATCGTCAAATAAGAACTCTGAGCTAAGTTGAGAAATCCATCT

10 GTTGAAAAATAATTCTAAATCGTCAAATAAGAACTCTGAGCTAAGTTGAGAAATCCATCT

17 GTTGAAAAATAATTCTAAATCGTCAAATAAGAACTCTGAGCTAAGTTGAGAAATCCATCT

14 GTTGAAAAATAATTCTAAATCGTCAAATAAGAACTCTGAGCTAAGTTGAGAAATCCATCT

13 GTTGAAAAATAATTCTAAATCGTCAAATAAGAACTCTGAGCTAAGTTGAGAAATCCATCT

12 GTTGAAAAATAATTCTAAATCGTCAAATAAGAACTCTGAGCTAAGTTGAGAAATCCATCT

8 GTTGAAAAATAATTCTAAATCGTCAAATAAGAACTCTGAGCTAAGTTGAGAAATCCATCT

7 GTTGAAAAATAATTCTAAATCGTCAAATAAGAACTCTGAGCTAAGTTGAGAAATCCATCT

19 GTTGAAAAATAATTCTAAATCGTCAAATAAGAACTCTGAGCTAAGTTGAGAAATCCATCT

18 GTTGAAAAATAATTCTAAATCGTCAAATAAGAACTCTGAGCTAAGTTGAGAAATCCATCT

22 GTTGAAAAATAATTCTAAATCGTCAAATAAGAACTCTGAGCTAAGTTGAGAAATCCATCT

21 GTTGAAAAATAATTCTAAATCGTCAAATAAGAACTCTGAGCTAAGTTGAGAAATCCATCT

9 GTTGAAAAATAATTCTAAATCGTCAAATAAGAACTCTGAGCTAAGTTGAGAAATCCATCT

6 GTTGAAAAATAATTCTAAATCGTCAAATAAGAACTCTGAGCTAAGTTGAGAAATCCATCT

5 GTTGAAAAATAATTCTAAATCGTCAAATAAGAACTCTGAGCTAAGTTGAGAAATCCATCT

4 GTTGAAAAATAATTCTAAATCGTCAAATAAGAACTCTGAGCTAAGTTGAGAAATCCATCT

3 GTTGAAAAATAATTCTAAATCGTCAAATAAGAACTCTGAGCTAAGTTGAGAAATCCATCT

20 GTTGAAAAATAATTCTAAATCGTCAAATAAGAACTCTGAGCTAAGTTGAGAAATCCATCT

************************************************************

1 ATATATTGATGCGCAGAGTATCGTTCCATCGTTGAAATAAATATCCTTGTCCTTGTAAGA

16 ATATATTGATGCGCAGAGTATCGTTCCATCGTTGAAATAAATATCCTTGTCCTTGTAAGA

15 ATATATTGATGCGCAGAGTATCGTTCCATCGTTGAAATAAATATCCTTGTCCTTGTAAGA

2 ATATATTGATGCGCAGAGTATCGTTCCATCGTTGAAATAAATATCCTTGTCCTTGTAAGA

10 ATATATTGATGCGCAGAGTATCGTTCCATCGTTGAAATAAATATCCTTGTCCTTGTAAGA

17 ATATATTGATGCGCAGAGTATCGTTCCATCGTTGAAATAAATATCCTTGTCCTTGTAAGA

14 ATATATTGATGCGCAGAGTATCGTTCCATCGTTGAAATAAATATCCTTGTCCTTGTAAGA

13 ATATATTGATGCGCAGAGTATCGTTCCATCGTTGAAATAAATATCCTTGTCCTTGTAAGA

12 ATATATTGATGCGCAGAGTATCGTTCCATCGTTGAAATAAATATCCTTGTCCTTGTAAGA

8 ATATATTGATGCGCAGAGTATCGTTCCATCGTTGAAATAAATATCCTTGTCCTTGTAAGA

7 ATATATTGATGCGCAGAGTATCGTTCCATCGTTGAAATAAATATCCTTGTCCTTGTAAGA

19 ATATATTGATGCGCAGAGTATCGTTCCATCGTTGAAATAAATATCCTTGTCCTTGTAAGA

18 ATATATTGATGCGCAGAGTATCGTTCCATCGTTGAAATAAATATCCTTGTCCTTGTAAGA

22 ATATATTGATGCGCAGAGTATCGTTCCATCGTTGAAATAAATATCCTTGTCCTTGTAAGA

21 ATATATTGATGCGCAGAGTATCGTTCCATCGTTGAAATAAATATCCTTGTCCTTGTAAGA

9 ATATATTGATGCGCAGAGTATCGTTCCATCGTTGAAATAAATATCCTTGTCCTTGTAAGA

6 ATATATTGATGCGCAGAGTATCGTTCCATCGTTGAAATAAATATCCTTGTCCTTGTAAGA

5 ATATATTGATGCGCAGAGTATCGTTCCATCGTTGAAATAAATATCCTTGTCCTTGTAAGA

4 ATATATTGATGCGCAGAGTATCGTTCCATCGTTGAAATAAATATCCTTGTCCTTGTAAGA

3 ATATATTGATGCGCAGAGTATCGTTCCATCGTTGAAATAAATATCCTTGTCCTTGTAAGA

20 ATATATTGATGCGCAGAGTATCGTTCCATCGTTGAAATAAATATCCTTGTCCTTGTAAGA

************************************************************

1 TTGGTATTGATTGAATAAATAATATAATGCGTCTGCATCTGTATCTGCGTCTGTACCTCC

16 TTGGTATTGATTGAATAAATAATATAATGCGTCTGCATCTGTATCTGCGTCTGTACCTCC

15 TTGGTATTGATTGAATAAATAATATAATGCGTCTGCATCTGTATCTGCGTCTGTACCTCC

2 TTGGTATTGATTGAATAAATAATATAATGCGTCTGCATCTGTATCTGCGTCTGTACCTCC

10 TTGGTATTGATTGAATAAATAATATAATGCGTCTGCATCTGTATCTGCGTCTGTACCTCC

17 TTGGTATTGATTGAATAAATAATATAATGCGTCTGCATCTGTATCTGCGTCTGTACCTCC

14 TTGGTATTGATTGAATAAATAATATAATGCGTCTGCATCTGTATCTGCGTCTGTACCTCC

13 TTGGTATTGATTGAATAAATAATATAATGCGTCTGCATCTGTATCTGCGTCTGTACCTCC

12 TTGGTATTGATTGAATAAATAATATAATGCGTCTGCATCTGTATCTGCGTCTGTACCTCC

8 TTGGTATTGATTGAATAAATAATATAATGCGTCTGCATCTGTATCTGCGTCTGTACCTCC

7 TTGGTATTGATTGAATAAATAATATAATGCGTCTGCATCTGTATCTGCGTCTGTACCTCC

19 TTGGTATTGATTGAATAAATAATATAATGCGTCTGCATCTGTATCTGCGTCTGTACCTCC

18 TTGGTATTGATTGAATAAATAATATAATGCGTCTGCATCTGTATCTGCGTCTGTACCTCC

22 TTGGTATTGATTGAATAAATAATATAATGCGTCTGCATCTGTATCTGCGTCTGTACCTCC

21 TTGGTATTGATTGAATAAATAATATAATGCGTCTGCATCTGTATCTGCGTCTGTACCTCC

9 TTGGTATTGATTGAATAAATAATATAATGCGTCTGCATCTGTATCTGCGTCTGTACCTCC

6 TTGGTATTGATTGAATAAATAATATAATGCGTCTGCATCTGTATCTGCGTCTGTACCTCC

5 TTGGTATTGATTGAATAAATAATATAATGCGTCTGCATCTGTATCTGCGTCTGTACCTCC

4 TTGGTATTGATTGAATAAATAATATAATGCGTCTGCATCTGTATCTGCGTCTGTACCTCC

3 TTGGTATTGATTGAATAAATAATATAATGCGTCTGCATCTGTATCTGCGTCTGTACCTCC

20 TTGGTATTGATTGAATAAATAATATAATGCGTCTGCATCTGTATCTGCGTCTGTACCTCC

************************************************************

1 AAATGCAGTCTCCACCGCGCCTCCACTTGGGCAGCCATCTGCAGCCAAAAATTCATTGTC

16 AAATGCAGTCTCCACCGCGCCTCCACTTGGGCAGCCATCTGCAGCCAAAAATTCATTGTC

15 AAATGCAGTCTCCACCGCGCCTCCACTTGGGCAGCCATCTGCAGCCAAAAATTCATTGTC

2 AAATGCAGTCTCCACCGCGCCTCCACTTGGGCAGCCATCTGCAGCCAAAAATTCATTGTC

10 AAATGCAGTCTCCACCGCGCCTCCACTTGGGCAGCCATCTGCAGCCAAAAATTCATTGTC

17 AAATGCAGTCTCCACCGCGCCTCCACTTGGGCAGCCATCTGCAGCCAAAAATTCATTGTC

14 AAATGCAGTCTCCACCGCGCCTCCACTTGGGCAGCCATCTGCAGCCAAAAATTCATTGTC

13 AAATGCAGTCTCCACCGCGCCTCCACTTGGGCAGCCATCTGCAGCCAAAAATTCATTGTC

12 AAATGCAGTCTCCACCGCGCCTCCACTTGGGCAGCCATCTGCAGCCAAAAATTCATTGTC

8 AAATGCAGTCTCCACCGCGCCTCCACTTGGGCAGCCATCTGCAGCCAAAAATTCATTGTC

7 AAATGCAGTCTCCACCGCGCCTCCACTTGGGCAGCCATCTGCAGCCAAAAATTCATTGTC

19 AAATGCAGTCTCCACCGCGCCTCCACTTGGGCAGCCATCTGCAGCCAAAAATTCATTGTC

18 AAATGCAGTCTCCACCGCGCCTCCACTTGGGCAGCCATCTGCAGCCAAAAATTCATTGTC

22 AAATGCAGTCTCCACCGCGCCTCCACTTGGGCAGCCATCTGCAGCCAAAAATTCATTGTC

21 AAATGCAGTCTCCACCGCGCCTCCACTTGGGCAGCCATCTGCAGCCAAAAATTCATTGTC

9 AAATGCAGTCTCCACCGCGCCTCCACTTGGGCAGCCATCTGCAGCCAAAAATTCATTGTC

6 AAATGCAGTCTCCACCGCGCCTCCACTTGGGCAGCCATCTGCAGCCAAAAATTCATTGTC

5 AAATGCAGTCTCCACCGCGCCTCCACTTGGGCAGCCATCTGCAGCCAAAAATTCATTGTC

4 AAATGCAGTCTCCACCGCGCCTCCACTTGGGCAGCCATCTGCAGCCAAAAATTCATTGTC

3 AAATGCAGTCTCCACCGCGCCTCCACTTGGGCAGCCATCTGCAGCCAAAAATTCATTGTC

20 AAATGCAGTCTCCACCGCGCCTCCACTTGGGCAGCCATCTGCAGCCAAAAATTCATTGTC

************************************************************

1 GACGGGTGTTCTTGTATTACCGAATAAATCATTGCGCACTGGCTTAGCCAGTAATTCTAT

16 GACGGGTGTTCTTGTATTACCGAATAAATCATTGCGCACTGGCTTAGCCAGTAATTCTAT

15 GACGGGTGTTCTTGTATTACCGAATAAATCATTGCGCACTGGCTTAGCCAGTAATTCTAT

2 GACGGGTGTTCTTGTATTACCGAATAAATCATTGCGCACTGGCTTAGCCAGTAATTCTAT

10 GACGGGTGTTCTTGTATTACCGAATAAATCATTGCGCACTGGCTTAGCCAGTAATTCTAT

17 GACGGGTGTTCTTGTATTACCGAATAAATCATTGCGCACTGGCTTAGCCAGTAATTCTAT

14 GACGGGTGTTCTTGTATTACCGAATAAATCATTGCGCACTGGCTTAGCCAGTAATTCTAT

13 GACGGGTGTTCTTGTATTACCGAATAAATCATTGCGCACTGGCTTAGCCAGTAATTCTAT

12 GACGGGTGTTCTTGTATTACCGAATAAATCATTGCGCACTGGCTTAGCCAGTAATTCTAT

8 GACGGGTGTTCTTGTATTACCGAATAAATCATTGCGCACTGGCTTAGCCAGTAATTCTAT

7 GACGGGTGTTCTTGTATTACCGAATAAATCATTGCGCACTGGCTTAGCCAGTAATTCTAT

19 GACGGGTGTTCTTGTATTACCGAATAAATCATTGCGCACTGGCTTAGCCAGTAATTCTAT

18 GACGGGTGTTCTTGTATTACCGAATAAATCATTGCGCACTGGCTTAGCCAGTAATTCTAT

22 GACGGGTGTTCTTGTATTACCGAATAAATCATTGCGCACTGGCTTAGCCAGTAATTCTAT

21 GACGGGTGTTCTTGTATTACCGAATAAATCATTGCGCACTGGCTTAGCCAGTAATTCTAT

9 GACGGGTGTTCTTGTATTACCGAATAAATCATTGCGCACTGGCTTAGCCAGTAATTCTAT

6 GACGGGTGTTCTTGTATTACCGAATAAATCATTGCGCACTGGCTTAGCCAGTAATTCTAT

5 GACGGGTGTTCTTGTATTACCGAATAAATCATTGCGCACTGGCTTAGCCAGTAATTCTAT

4 GACGGGTGTTCTTGTATTACCGAATAAATCATTGCGCACTGGCTTAGCCAGTAATTCTAT

3 GACGGGTGTTCTTGTATTACCGAATAAATCATTGCGCACTGGCTTAGCCAGTAATTCTAT

20 GACGGGTGTTCTTGTATTACCGAATAAATCATTGCGCACTGGCTTAGCCAGTAATTCTAT

************************************************************

1 CAAGTAATTTTTCAGATTGTCGAATGTATTTGGGAGTTGTAGCCTGAGATCCATAGTACT

16 CAAGTAATTTTTCAGATTGTCGAATGTATTTGGGAGTTGTAGCCTGAGATCCATAGTACT

15 CAAGTAATTTTTCAGATTGTCGAATGTATTTGGGAGTTGTAGCCTGAGATCCATAGTACT

2 CAAGTAATTTTTCAGATTGTCGAATGTATTTGGGAGTTGTAGCCTGAGATCCATAGTACT

10 CAAGTAATTTTTCAGATTGTCGAATGTATTTGGGAGTTGTAGCCTGAGATCCATAGTACT

17 CAAGTAATTTTTCAGATTGTCGAATGTATTTGGGAGTTGTAGCCTGAGATCCATAGTACT

14 CAAGTAATTTTTCAGATTGTCGAATGTATTTGGGAGTTGTAGCCTGAGATCCATAGTACT

13 CAAGTAATTTTTCAGATTGTCGAATGTATTTGGGAGTTGTAGCCTGAGATCCATAGTACT

12 CAAGTAATTTTTCAGATTGTCGAATGTATTTGGGAGTTGTAGCCTGAGATCCATAGTACT

8 CAAGTAATTTTTCAGATTGTCGAATGTATTTGGGAGTTGTAGCCTGAGATCCATAGTACT

7 CAAGTAATTTTTCAGATTGTCGAATGTATTTGGGAGTTGTAGCCTGAGATCCATAGTACT

19 CAAGTAATTTTTCAGATTGTCGAATGTATTTGGGAGTTGTAGCCTGAGATCCATAGTACT

18 CAAGTAATTTTTCAGATTGTCGAATGTATTTGGGAGTTGTAGCCTGAGATCCATAGTACT

22 CAAGTAATTTTTCAGATTGTCGAATGTATTTGGGAGTTGTAGCCTGAGATCCATAGTACT

21 CAAGTAATTTTTCAGATTGTCGAATGTATTTGGGAGTTGTAGCCTGAGATCCATAGTACT

9 CAAGTAATTTTTCAGATTGTCGAATGTATTTGGGAGTTGTAGCCTGAGATCCATAGTACT

6 CAAGTAATTTTTCAGATTGTCGAATGTATTTGGGAGTTGTAGCCTGAGATCCATAGTACT

5 CAAGTAATTTTTCAGATTGTCGAATGTATTTGGGAGTTGTAGCCTGAGATCCATAGTACT

4 CAAGTAATTTTTCAGATTGTCGAATGTATTTGGGAGTTGTAGCCTGAGATCCATAGTACT

3 CAAGTAATTTTTCAGATTGTCGAATGTATTTGGGAGTTGTAGCCTGAGATCCATAGTACT

20 CAAGTAATTTTTCAGATTGTCGAATGTATTTGGGAGTTGTAGCCTGAGATCCATAGTACT

************************************************************

1 CGAGTTTTTAGGTCTGAACAGGTGATGAGTATATTTGCATCACTGTAAAACACTCCTCAT

16 CGAGTTTTTAGGTCTGAACAGGTGATGAGTACATTTGCATCACTGTAAAACACTCCTCAT

15 CGAGTTTTTAGGTCTGAACAGGTGATGAGTACATTTGCATCACTGTAAAACACTCCTCAT

2 CGAGTTTTTAGGTCTGAACAGGTGATGAGTACATTTGCATCACTGTAAAACACTCCTCAT

10 CGAGTTTTTAGGTCTGAACAGGTGATGAGTACATTTGCATCACTGTAAAACACTCCTCAT

17 CGAGTTTTTAGGTCTGAACAGGTGATGAGTACATTTGCATCACTGTAAAACACTCCTCAT

14 CGAGTTTTTAGGTCTGAACAGGTGATGAGTACATTTGCATCACTGTAAAACACTCCTCAT

13 CGAGTTTTTAGGTCTGAACAGGTGATGAGTACATTTGCATCACTGTAAAACACTCCTCAT

12 CGAGTTTTTAGGTCTGAACAGGTGATGAGTACATTTGCATCACTGTAAAACACTCCTCAT

8 CGAGTTTTTAGGTCTGAACAGGTGATGAGTACATTTGCATCACTGTAAAACACTCCTCAT

7 CGAGTTTTTAGGTCTGAACAGGTGATGAGTACATTTGCATCACTGTAAAACACTCCTCAT

19 CGAGTTTTTAGGTCTGAACAGGTGATGAGTACATTTGCATCACTGTAAAACACTCCTCAT

18 CGAGTTTTTAGGTCTGAACAGGTGATGAGTACATTTGCATCACTGTAAAACACTCCTCAT

22 CGAGTTTTTAGGTCTGAACAGGTGATGAGTACATTTGCATCACTGTAAAACACTCCTCAT

21 CGAGTTTTTAGGTCTGAACAGGTGATGAGTACATTTGCATCACTGTAAAACACTCCTCAT

9 CGAGTTTTTAGGTCTGAACAGGTGATGAGTACATTTGCATCACTGTAAAACACTCCTCAT

6 CGAGTTTTTAGGTCTGAACAGGTGATGAGTACATTTGCATCACTGTAAAACACTCCTCAT

5 CGAGTTTTTAGGTCTGAACAGGTGATGAGTACATTTGCATCACTGTAAAACACTCCTCAT

4 CGAGTTTTTAGGTCTGAACAGGTGATGAGTACATTTGCATCACTGTAAAACACTCCTCAT

3 CGAGTTTTTAGGTCTGAACAGGTGATGAGTACATTTGCATCACTGTAAAACACTCCTCAT

20 CGAGTTTTTAGGTCTGAACAGGTGATGAGTACATTTGCATCACTGTAAAACACTCCTCAT

*******************************.****************************

1 ACCTAACTACATACTCATTCTCAGTTTTTATCTGTATGGTCAAGCCACTGGACTTGATGT

16 ACCTAACTACATACTCATTCTCAGTTTTTATCTGTATGGTCAAGCCACTGGACTTGATGT

15 ACCTAACTACATACTCATTCTCAGTTTTTATCTGTATGGTCAAGCCACTGGACTTGATGT

2 ACCTAACTACATACTCATTCTCAGTTTTTATCTGTATGGTCAAGCCACTGGACTTGATGT

10 ACCTAACTACATACTCATTCTCAGTTTTTATCTGTATGGTCAAGCCACTGGACTTGATGT

17 ACCTAACTACATACTCATTCTCAGTTTTTATCTGTATGGTCAAGCCACTGGACTTGATGT

14 ACCTAACTACATACTCATTCTCAGTTTTTATCTGTATGGTCAAGCCACTGGACTTGATGT

13 ACCTAACTACATACTCATTCTCAGTTTTTATCTGTATGGTCAAGCCACTGGACTTGATGT

12 ACCTAACTACATACTCATTCTCAGTTTTTATCTGTATGGTCAAGCCACTGGACTTGATGT

8 ACCTAACTACATACTCATTCTCAGTTTTTATCTGTATGGTCAAGCCACTGGACTTGATGT

7 ACCTAACTACATACTCATTCTCAGTTTTTATCTGTATGGTCAAGCCACTGGACTTGATGT

19 ACCTAACTACATACTCATTCTCAGTTTTTATCTGTATGGTCAAGCCACTGGACTTGATGT

18 ACCTAACTACATACTCATTCTCAGTTTTTATCTGTATGGTCAAGCCACTGGACTTGATGT

22 ACCTAACTACATACTCATTCTCAGTTTTTATCTGTATGGTCAAGCCACTGGACTTGATGT

21 ACCTAACTACATACTCATTCTCAGTTTTTATCTGTATGGTCAAGCCACTGGACTTGATGT

9 ACCTAACTACATACTCATTCTCAGTTTTTATCTGTATGGTCAAGCCACTGGACTTGATGT

6 ACCTAACTACATACTCATTCTCAGTTTTTATCTGTATGGTCAAGCCACTGGACTTGATGT

5 ACCTAACTACATACTCATTCTCAGTTTTTATCTGTATGGTCAAGCCACTGGACTTGATGT

4 ACCTAACTACATACTCATTCTCAGTTTTTATCTGTATGGTCAAGCCACTGGACTTGATGT

3 ACCTAACTACATACTCATTCTCAGTTTTTATCTGTATGGTCAAGCCACTGGACTTGATGT

20 ACCTAACTACATACTCATTCTCAGTTTTTATCTGTATGGTCAAGCCACTGGACTTGATGT

************************************************************

1 GTCCATCGACAGAGCATCCAACACCGTACTTGAGATCGAGAAGCCTCTTCACCTTCCTTG

16 GTCCATCGACAGAGCATCCAACACCGTACTTGAGATCGAGAAGCCTCTTCACCTTCCTTG

15 GTCCATCGACAGAGCATCCAACACCGTACTTGAGATCGAGAAGCCTCTTCACCTTCCTTG

2 GTCCATCGACAGAGCATCCAACACCGTACTTGAGATCGAGAAGCCTCTTCACCTTCCTTG

10 GTCCATCGACAGAGCATCCAACACCGTACTTGAGATCGAGAAGCCTCTTCACCTTCCTTG

17 GTCCATCGACAGAGCATCCAACACCGTACTTGAGATCGAGAAGCCTCTTCACCTTCCTTG

14 GTCCATCGACAGAGCATCCAACACCGTACTTGAGATCGAGAAGCCTCTTCACCTTCCTTG

13 GTCCATCGACAGAGCATCCAACACCGTACTTGAGATCGAGAAGCCTCTTCACCTTCCTTG

12 GTCCATCGACAGAGCATCCAACACCGTACTTGAGATCGAGAAGCCTCTTCACCTTCCTTG

8 GTCCATCGACAGAGCATCCAACACCGTACTTGAGATCGAGAAGCCTCTTCACCTTCCTTG

7 GTCCATCGACAGAGCATCCAACACCGTACTTGAGATCGAGAAGCCTCTTCACCTTCCTTG

19 GTCCATCGACAGAGCATCCAACACCGTACTTGAGATCGAGAAGCCTCTTCACCTTCCTTG

18 GTCCATCGACAGAGCATCCAACACCGTACTTGAGATCGAGAAGCCTCTTCACCTTCCTTG

22 GTCCATCGACAGAGCATCCAACACCGTACTTGAGATCGAGAAGCCTCTTCACCTTCCTTG

21 GTCCATCGACAGAGCATCCAACACCGTACTTGAGATCGAGAAGCCTCTTCACCTTCCTTG

9 GTCCATCGACAGAGCATCCAACACCGTACTTGAGATCGAGAAGCCTCTTCACCTTCCTTG

6 GTCCATCGACAGAGCATCCAACACCGTACTTGAGATCGAGAAGCCTCTTCACCTTCCTTG

5 GTCCATCGACAGAGCATCCAACACCGTACTTGAGATCGAGAAGCCTCTTCACCTTCCTTG

4 GTCCATCGACAGAGCATCCAACACCGTACTTGAGATCGAGAAGCCTCTTCACCTTCCTTG

3 GTCCATCGACAGAGCATCCAACACCGTACTTGAGATCGAGAAGCCTCTTCACCTTCCTTG

20 GTCCATCGACAGAGCATCCAACACCGTACTTGAGATCGAGAAGCCTCTTCACCTTCCTTG

************************************************************

1 CTGTCTCACCTTCACCCGTAATACAATAGTTCCCGTCTAAACTTATTTTAATTTCTTCCA

16 CTGTCTCACCTTCACCCGTAATACAATAGTTCCCGTCTAAACTTATTTTAATTTCTTCCA

15 CTGTCTCACCTTCACCCGTAATACAATAGTTCCCGTCTAAACTTATTTTAATTTCTTCCA

2 CTGTCTCACCTTCACCCGTAATACAATAGTTCCCGTCTAAACTTATTTTAATTTCTTCCA

10 CTGTCTCACCTTCACCCGTAATACAATAGTTCCCGTCTAAACTTATTTTAATTTCTTCCA

17 CTGTCTCACCTTCACCCGTAATACAATAGTTCCCGTCTAAACTTATTTTAATTTCTTCCA

14 CTGTCTCACCTTCACCCGTAATACAATAGTTCCCGTCTAAACTTATTTTAATTTCTTCCA

13 CTGTCTCACCTTCACCCGTAATACAATAGTTCCCGTCTAAACTTATTTTAATTTCTTCCA

12 CTGTCTCACCTTCACCCGTAATACAATAGTTCCCGTCTAAACTTATTTTAATTTCTTCCA

8 CTGTCTCACCTTCACCCGTAATACAATAGTTCCCGTCTAAACTTATTTTAATTTCTTCCA

7 CTGTCTCACCTTCACCCGTAATACAATAGTTCCCGTCTAAACTTATTTTAATTTCTTCCA

19 CTGTCTCACCTTCACCCGTAATACAATAGTTCCCGTCTAAACTTATTTTAATTTCTTCCA

18 CTGTCTCACCTTCACCCGTAATACAATAGTTCCCGTCTAAACTTATTTTAATTTCTTCCA

22 CTGTCTCACCTTCACCCGTAATACAATAGTTCCCGTCTAAACTTATTTTAATTTCTTCCA

21 CTGTCTCACCTTCACCCGTAATACAATAGTTCCCGTCTAAACTTATTTTAATTTCTTCCA

9 CTGTCTCACCTTCACCCGTAATACAATAGTTCCCGTCTAAACTTATTTTAATTTCTTCCA

6 CTGTCTCACCTTCACCCGTAATACAATAGTTCCCGTCTAAACTTATTTTAATTTCTTCCA

5 CTGTCTCACCTTCACCCGTAATACAATAGTTCCCGTCTAAACTTATTTTAATTTCTTCCA

4 CTGTCTCACCTTCACCCGTAATACAATAGTTCCCGTCTAAACTTATTTTAATTTCTTCCA

3 CTGTCTCACCTTCACCCGTAATACAATAGTTCCCGTCTAAACTTATTTTAATTTCTTCCA

20 CTGTCTCACCTTCACCCGTAATACAATAGTTCCCGTCTAAACTTATTTTAATTTCTTCCA

************************************************************

1 TATGACATCACTAAGTTGAACCAGTAAAGTAGTAGTATTTTTATTTTTAAGATGAAATTT

16 TATGACATCACTAAGTTGAACCAGTAAAGTAGTAGTATTTTTATTTTTAAGATGAAATTT

15 TATGACATCACTAAGTTGAACCAGTAAAGTAGTAGTATTTTTATTTTTAAGATGAAATTT

2 TATGACATCACTAAGTTGAACCAGTAAAGTAGTAGTATTTTTATTTTTAAGATGAAATTT

10 TATGACATCACTAAGTTGAACCAGTAAAGTAGTAGTATTTTTATTTTTAAGATGAAATTT

17 TATGACATCACTAAGTTGAACCAGTAAAGTAGTAGTATTTTTATTTTTAAGATGAAATTT

14 TATGACATCACTAAGTTGAACCAGTAAAGTAGTAGTATTTTTATTTTTAAGATGAAATTT

13 TATGACATCACTAAGTTGAACCAGTAAAGTAGTAGTATTTTTATTTTTAAGATGAAATTT

12 TATGACATCACTAAGTTGAACCAGTAAAGTAGTAGTATTTTTATTTTTAAGATGAAATTT

8 TATGACATCACTAAGTTGAACCAGTAAAGTAGTAGTATTTTTATTTTTAAGATGAAATTT

7 TATGACATCACTAAGTTGAACCAGTAAAGTAGTAGTATTTTTATTTTTAAGATGAAATTT

19 TATGACATCACTAAGTTGAACCAGTAAAGTAGTAGTATTTTTATTTTTAAGATGAAATTT

18 TATGACATCACTAAGTTGAACCAGTAAAGTAGTAGTATTTTTATTTTTAAGATGAAATTT

22 TATGACATCACTAAGTTGAACCAGTAAAGTAGTAGTATTTTTATTTTTAAGATGAAATTT

21 TATGACATCACTAAGTTGAACCAGTAAAGTAGTAGTATTTTTATTTTTAAGATGAAATTT

9 TATGACATCACTAAGTTGAACCAGTAAAGTAGTAGTATTTTTATTTTTAAGATGAAATTT

6 TATGACATCACTAAGTTGAACCAGTAAAGTAGTAGTATTTTTATTTTTAAGATGAAATTT

5 TATGACATCACTAAGTTGAACCAGTAAAGTAGTAGTATTTTTATTTTTAAGATGAAATTT

4 TATGACATCACTAAGTTGAACCAGTAAAGTAGTAGTATTTTTATTTTTAAGATGAAATTT

3 TATGACATCACTAAGTTGAACCAGTAAAGTAGTAGTATTTTTATTTTTAAGATGAAATTT

20 TATGACATCACTAAGTTGAACCAGTAAAGTAGTAGTATTTTTATTTTTAAGATGAAATTT

************************************************************

1 TTATAAATGTCTACAACTTTGCTTTATATAATAATAGGTAGGCTACACTATCTTCACCGC

16 TTATAAATGTCTACAACTTTGCTTTATATAATAATAGGTAGGCTACACTATCTTCACCGC

15 TTATAAATGTCTACAACTTTGCTTTATATAATAATAGGTAGGCTACACTATCTTCACCGC

2 TTATAAATGTCTACAACTTTGCTTTATATAATAATAGGTAGGCTACACTATCTTCACCGC

10 TTATAAATGTCTACAACTTTGCTTTATATAATAATAGGTAGGCTACACTATCTTCACCGC

17 TTATAAATGTCTACAACTTTGCTTTATATAATAATAGGTAGGCTACACTATCTTCACCGC

14 TTATAAATGTCTACAACTTTGCTTTATATAATAATAGGTAGGCTACACTATCTTCACCGC

13 TTATAAATGTCTACAACTTTGCTTTATATAATAATAGGTAGGCTACACTATCTTCACCGC

12 TTATAAATGTCTACAACTTTGCTTTATATAATAATAGGTAGGCTACACTATCTTCACCGC

8 TTATAAATGTCTACAACTTTGCTTTATATAATAATAGGTAGGCTACACTATCTTCACCGC

7 TTATAAATGTCTACAACTTTGCTTTATATAATAATAGGTAGGCTACACTATCTTCACCGC

19 TTATAAATGTCTACAACTTTGCTTTATATAATAATAGGTAGGCTACACTATCTTCACCGC

18 TTATAAATGTCTACAACTTTGCTTTATATAATAATAGGTAGGCTACACTATCTTCACCGC

22 TTATAAATGTCTACAACTTTGCTTTATATAATAATAGGTAGGCTACACTATCTTCACCGC

21 TTATAAATGTCTACAACTTTGCTTTATATAATAATAGGTAGGCTACACTATCTTCACCGC

9 TTATAAATGTCTACAACTTTGCTTTATATAATAATAGGTAGGCTACACTATCTTCACCGC

6 TTATAAATGTCTACAACTTTGCTTTATATAATAATAGGTAGGCTACACTATCTTCACCGC

5 TTATAAATGTCTACAACTTTGCTTTATATAATAATAGGTAGGCTACACTATCTTCACCGC

4 TTATAAATGTCTACAACTTTGCTTTATATAATAATAGGTAGGCTACACTATCTTCACCGC

3 TTATAAATGTCTACAACTTTGCTTTATATAATAATAGGTAGGCTACACTATCTTCACCGC

20 TTATAAATGTCTACAACTTTGCTTTATATAATAATAGGTAGGCTACACTATCTTCACCGC

************************************************************

1 CTCCTTCCAATTGGGCAATTTTGTCTTTGGATACTTTGGTGACCTTGTTGTCGTCGAATT

16 CTCCTTCCAATTGGGCAATTTTGTCTTTGGATACTTTGGTGACCTTGTTGTCGTCGAATT

15 CTCCTTCCAATTGGGCAATTTTGTCTTTGGATACTTTGGTGACCTTGTTGTCGTCGAATT

2 CTCCTTCCAATTGGGCAATTTTGTCTTTGGATACTTTGGTGACCTTGTTGTCGTCGAATT

10 CTCCTTCCAATTGGGCAATTTTGTCTTTGGATACTTTGGTGACCTTGTTGTCGTCGAATT

17 CTCCTTCCAATTGGGCAATTTTGTCTTTGGATACTTTGGTGACCTTGTTGTCGTCGAATT

14 CTCCTTCCAATTGGGCAATTTTGTCTTTGGATACTTTGGTGACCTTGTTGTCGTCGAATT

13 CTCCTTCCAATTGGGCAATTTTGTCTTTGGATACTTTGGTGACCTTGTTGTCGTCGAATT

12 CTCCTTCCAATTGGGCAATTTTGTCTTTGGATACTTTGGTGACCTTGTTGTCGTCGAATT

8 CTCCTTCCAATTGGGCAATTTTGTCTTTGGATACTTTGGTGACCTTGTTGTCGTCGAATT

7 CTCCTTCCAATTGGGCAATTTTGTCTTTGGATACTTTGGTGACCTTGTTGTCGTCGAATT

19 CTCCTTCCAATTGGGCAATTTTGTCTTTGGATACTTTGGTGACCTTGTTGTCGTCGAATT

18 CTCCTTCCAATTGGGCAATTTTGTCTTTGGATACTTTGGTGACCTTGTTGTCGTCGAATT

22 CTCCTTCCAATTGGGCAATTTTGTCTTTGGATACTTTGGTGACCTTGTTGTCGTCGAATT

21 CTCCTTCCAATTGGGCAATTTTGTCTTTGGATACTTTGGTGACCTTGTTGTCGTCGAATT

9 CTCCTTCCAATTGGGCAATTTTGTCTTTGGATACTTTGGTGACCTTGTTGTCGTCGAATT

6 CTCCTTCCAATTGGGCAATTTTGTCTTTGGATACTTTGGTGACCTTGTTGTCGTCGAATT

5 CTCCTTCCAATTGGGCAATTTTGTCTTTGGATACTTTGGTGACCTTGTTGTCGTCGAATT

4 CTCCTTCCAATTGGGCAATTTTGTCTTTGGATACTTTGGTGACCTTGTTGTCGTCGAATT

3 CTCCTTCCAATTGGGCAATTTTGTCTTTGGATACTTTGGTGACCTTGTTGTCGTCGAATT

20 CTCCTTCCAATTGGGCAATTTTGTCTTTGGATACTTTGGTGACCTTGTTGTCGTCGAATT

************************************************************

1 TGTACCAATCGTCCTTGTCCTCTTCCGACTTGTGCACATCCCCTCTGACCCAGGCTATGT

16 TGTACCAATCGTCCTTGTCCTCTTCCGACTTGTGCACATCCCCTCTGACCCAGGCTATGT

15 TGTACCAATCGTCCTTGTCCTCTTCCGACTTGTGCACATCCCCTCTGACCCAGGCTATGT

2 TGTACCAATCGTCCTTGTCCTCTTCCGACTTGTGCACATCCCCTCTGACCCAGGCTATGT

10 TGTACCAATCGTCCTTGTCCTCTTCCGACTTGTGCACATCCCCTCTGACCCAGGCTATGT

17 TGTACCAATCGTCCTTGTCCTCTTCCGACTTGTGCACATCCCCTCTGACCCAGGCTATGT

14 TGTACCAATCGTCCTTGTCCTCTTCCGACTTGTGCACATCCCCTCTGACCCAGGCTATGT

13 TGTACCAATCGTCCTTGTCCTCTTCCGACTTGTGCACATCCCCTCTGACCCAGGCTATGT

12 TGTACCAATCGTCCTTGTCCTCTTCCGACTTGTGCACATCCCCTCTGACCCAGGCTATGT

8 TGTACCAATCGTCCTTGTCCTCTTCCGACTTGTGCACATCCCCTCTGACCCAGGCTATGT

7 TGTACCAATCGTCCTTGTCCTCTTCCGACTTGTGCACATCCCCTCTGACCCAGGCTATGT

19 TGTACCAATCGTCCTTGTCCTCTTCCGACTTGTGCACATCCCCTCTGACCCAGGCTATGT

18 TGTACCAATCGTCCTTGTCCTCTTCCGACTTGTGCACATCCCCTCTGACCCAGGCTATGT

22 TGTACCAATCGTCCTTGTCCTCTTCCGACTTGTGCACATCCCCTCTGACCCAGGCTATGT

21 TGTACCAATCGTCCTTGTCCTCTTCCGACTTGTGCACATCCCCTCTGACCCAGGCTATGT

9 TGTACCAATCGTCCTTGTCCTCTTCCGACTTGTGCACATCCCCTCTGACCCAGGCTATGT

6 TGTACCAATCGTCCTTGTCCTCTTCCGACTTGTGCACATCCCCTCTGACCCAGGCTATGT

5 TGTACCAATCGTCCTTGTCCTCTTCCGACTTGTGCACATCCCCTCTGACCCAGGCTATGT

4 TGTACCAATCGTCCTTGTCCTCTTCCGACTTGTGCACATCCCCTCTGACCCAGGCTATGT

3 TGTACCAATCGTCCTTGTCCTCTTCCGACTTGTGCACATCCCCTCTGACCCAGGCTATGT

20 TGTACCAATCGTCCTTGTCCTCTTCCGACTTGTGCACATCCCCTCTGACCCAGGCTATGT

************************************************************

1 AGTGACCGCCATCTGCGTTTACACCTTTGTGCGTCACTATACCCACCAAATCGTACAAGC

16 AGTGACCGCCATCTGCGTTTACACCTTTGTGCGTCACTATACCCACCAAATCGTACAAGC

15 AGTGACCGCCATCTGCGTTTACACCTTTGTGCGTCACTATACCCACCAAATCGTACAAGC

2 AGTGACCGCCATCTGCGTTTACACCTTTGTGCGTCACTATACCCACCAAATCGTACAAGC

10 AGTGACCGCCATCTGCGTTTACACCTTTGTGCGTCACTATACCCACCAAATCGTACAAGC

17 AGTGACCGCCATCTGCGTTTACACCTTTGTGCGTCACTATACCCACCAAATCGTACAAGC

14 AGTGACCGCCATCTGCGTTTACACCTTTGTGCGTCACTATACCCACCAAATCGTACAAGC

13 AGTGACCGCCATCTGCGTTTACACCTTTGTGCGTCACTATACCCACCAAATCGTACAAGC

12 AGTGACCGCCATCTGCGTTTACACCTTTGTGCGTCACTATACCCACCAAATCGTACAAGC

8 AGTGACCGCCATCTGCGTTTACACCTTTGTGCGTCACTATACCCACCAAATCGTACAAGC

7 AGTGACCGCCATCTGCGTTTACACCTTTGTGCGTCACTATACCCACCAAATCGTACAAGC

19 AGTGACCGCCATCTGCGTTTACACCTTTGTGCGTCACTATACCCACCAAATCGTACAAGC

18 AGTGACCGCCATCTGCGTTTACACCTTTGTGCGTCACTATACCCACCAAATCGTACAAGC

22 AGTGACCGCCATCTGCGTTTACACCTTTGTGCGTCACTATACCCACCAAATCGTACAAGC

21 AGTGACCGCCATCTGCGTTTACACCTTTGTGCGTCACTATACCCACCAAATCGTACAAGC

9 AGTGACCGCCATCTGCGTTTACACCTTTGTGCGTCACTATACCCACCAAATCGTACAAGC

6 AGTGACCGCCATCTGCGTTTACACCTTTGTGCGTCACTATACCCACCAAATCGTACAAGC

5 AGTGACCGCCATCTGCGTTTACACCTTTGTGCGTCACTATACCCACCAAATCGTACAAGC

4 AGTGACCGCCATCTGCGTTTACACCTTTGTGCGTCACTATACCCACCAAATCGTACAAGC

3 AGTGACCGCCATCTGCGTTTACACCTTTGTGCGTCACTATACCCACCAAATCGTACAAGC

20 AGTGACCGCCATCTGCGTTTACACCTTTGTGCGTCACTATACCCACCAAATCGTACAAGC

************************************************************

1 CACTCTGTGAGCAACCAGTGTCATTTTTTAAGTCATTATTGACAAGCTCATCAAGCTCTT

16 CACTCTGTGAGCAACCAGTGTCATTTTTTAAGTCATTATTGACAAGCTCATCAAGCTCTT

15 CACTCTGTGAGCAACCAGTGTCATTTTTTAAGTCATTATTGACAAGCTCATCAAGCTCTT

2 CACTCTGTGAGCAACCAGTGTCATTTTTTAAGTCATTATTGACAAGCTCATCAAGCTCTT

10 CACTCTGTGAGCAACCAGTGTCATTTTTTAAGTCATTATTGACAAGCTCATCAAGCTCTT

17 CACTCTGTGAGCAACCAGTGTCATTTTTTAAGTCATTATTGACAAGCTCATCAAGCTCTT

14 CACTCTGTGAGCAACCAGTGTCATTTTTTAAGTCATTATTGACAAGCTCATCAAGCTCTT

13 CACTCTGTGAGCAACCAGTGTCATTTTTTAAGTCATTATTGACAAGCTCATCAAGCTCTT

12 CACTCTGTGAGCAACCAGTGTCATTTTTTAAGTCATTATTGACAAGCTCATCAAGCTCTT

8 CACTCTGTGAGCAACCAGTGTCATTTTTTAAGTCATTATTGACAAGCTCATCAAGCTCTT

7 CACTCTGTGAGCAACCAGTGTCATTTTTTAAGTCATTATTGACAAGCTCATCAAGCTCTT

19 CACTCTGTGAGCAACCAGTGTCATTTTTTAAGTCATTATTGACAAGCTCATCAAGCTCTT

18 CACTCTGTGAGCAACCAGTGTCATTTTTTAAGTCATTATTGACAAGCTCATCAAGCTCTT

22 CACTCTGTGAGCAACCAGTGTCATTTTTTAAGTCATTATTGACAAGCTCATCAAGCTCTT

21 CACTCTGTGAGCAACCAGTGTCATTTTTTAAGTCATTATTGACAAGCTCATCAAGCTCTT

9 CACTCTGTGAGCAACCAGTGTCATTTTTTAAGTCATTATTGACAAGCTCATCAAGCTCTT

6 CACTCTGTGAGCAACCAGTGTCATTTTTTAAGTCATTATTGACAAGCTCATCAAGCTCTT

5 CACTCTGTGAGCAACCAGTGTCATTTTTTAAGTCATTATTGACAAGCTCATCAAGCTCTT

4 CACTCTGTGAGCAACCAGTGTCATTTTTTAAGTCATTATTGACAAGCTCATCAAGCTCTT

3 CACTCTGTGAGCAACCAGTGTCATTTTTTAAGTCATTATTGACAAGCTCATCAAGCTCTT

20 CACTCTGTGAGCAACCAGTGTCATTTTTTAAGTCATTATTGACAAGCTCATCAAGCTCTT

************************************************************

1 TCTTCTCTCTCTCCTCATAAACGTTCTCATCGAGGAGCTCCACGTCACCAATCTTGCCCT

16 TCTTCTCTCTCTCCTCATAAACGTTCTCATCGAGGAGCTCCACGTCACCAATCTTGCCCT

15 TCTTCTCTCTCTCCTCATAAACGTTCTCATCGAGGAGCTCCACGTCACCAATCTTGCCCT

2 TCTTCTCTCTCTCCTCATAAACGTTCTCATCGAGGAGCTCCACGTCACCAATCTTGCCCT

10 TCTTCTCTCTCTCCTCATAAACGTTCTCATCGAGGAGCTCCACGTCACCAATCTTGCCCT

17 TCTTCTCTCTCTCCTCATAAACGTTCTCATCGAGGAGCTCCACGTCACCAATCTTGCCCT

14 TCTTCTCTCTCTCCTCATAAACGTTCTCATCGAGGAGCTCCACGTCACCAATCTTGCCCT

13 TCTTCTCTCTCTCCTCATAAACGTTCTCATCGAGGAGCTCCACGTCACCAATCTTGCCCT

12 TCTTCTCTCTCTCCTCATAAACGTTCTCATCGAGGAGCTCCACGTCACCAATCTTGCCCT

8 TCTTCTCTCTCTCCTCATAAACGTTCTCATCGAGGAGCTCCACGTCACCAATCTTGCCCT

7 TCTTCTCTCTCTCCTCATAAACGTTCTCATCGAGGAGCTCCACGTCACCAATCTTGCCCT

19 TCTTCTCTCTCTCCTCATAAACGTTCTCATCGAGGAGCTCCACGTCACCAATCTTGCCCT

18 TCTTCTCTCTCTCCTCATAAACGTTCTCATCGAGGAGCTCCACGTCACCAATCTTGCCCT

22 TCTTCTCTCTCTCCTCATAAACGTTCTCATCGAGGAGCTCCACGTCACCAATCTTGCCCT

21 TCTTCTCTCTCTCCTCATAAACGTTCTCATCGAGGAGCTCCACGTCACCAATCTTGCCCT

9 TCTTCTCTCTCTCCTCATAAACGTTCTCATCGAGGAGCTCCACGTCACCAATCTTGCCCT

6 TCTTCTCTCTCTCCTCATAAACGTTCTCATCGAGGAGCTCCACGTCACCAATCTTGCCCT

5 TCTTCTCTCTCTCCTCATAAACGTTCTCATCGAGGAGCTCCACGTCACCAATCTTGCCCT

4 TCTTCTCTCTCTCCTCATAAACGTTCTCATCGAGGAGCTCCACGTCACCAATCTTGCCCT

3 TCTTCTCTCTCTCCTCATAAACGTTCTCATCGAGGAGCTCCACGTCACCAATCTTGCCCT

20 TCTTCTCTCTCTCCTCATAAACGTTCTCATCGAGGAGCTCCACGTCACCAATCTTGCCCT

************************************************************

1 GCTCATCTTTGATTTCATCGATCAACATATCGTCGGTGTTTTTAGCCTCGTTGGCTTTTT

16 GCTCATCTTTGATTTCATCGATCAACATATCGTCGGTGTTTTTAGCCTCGTTGGCTTTTT

15 GCTCATCTTTGATTTCATCGATCAACATATCGTCGGTGTTTTTAGCCTCGTTGGCTTTTT

2 GCTCATCTTTGATTTCATCGATCAACATATCGTCGGTGTTTTTAGCCTCGTTGGCTTTTT

10 GCTCATCTTTGATTTCATCGATCAACATATCGTCGGTGTTTTTAGCCTCGTTGGCTTTTT

17 GCTCATCTTTGATTTCATCGATCAACATATCGTCGGTGTTTTTAGCCTCGTTGGCTTTTT

14 GCTCATCTTTGATTTCATCGATCAACATATCGTCGGTGTTTTTAGCCTCGTTGGCTTTTT

13 GCTCATCTTTGATTTCATCGATCAACATATCGTCGGTGTTTTTAGCCTCGTTGGCTTTTT

12 GCTCATCTTTGATTTCATCGATCAACATATCGTCGGTGTTTTTAGCCTCGTTGGCTTTTT

8 GCTCATCTTTGATTTCATCGATCAACATATCGTCGGTGTTTTTAGCCTCGTTGGCTTTTT

7 GCTCATCTTTGATTTCATCGATCAACATATCGTCGGTGTTTTTAGCCTCGTTGGCTTTTT

19 GCTCATCTTTGATTTCATCGATCAACATATCGTCGGTGTTTTTAGCCTCGTTGGCTTTTT

18 GCTCATCTTTGATTTCATCGATCAACATATCGTCGGTGTTTTTAGCCTCGTTGGCTTTTT

22 GCTCATCTTTGATTTCATCGATCAACATATCGTCGGTGTTTTTAGCCTCGTTGGCTTTTT

21 GCTCATCTTTGATTTCATCGATCAACATATCGTCGGTGTTTTTAGCCTCGTTGGCTTTTT

9 GCTCATCTTTGATTTCATCGATCAACATATCGTCGGTGTTTTTAGCCTCGTTGGCTTTTT

6 GCTCATCTTTGATTTCATCGATCAACATATCGTCGGTGTTTTTAGCCTCGTTGGCTTTTT

5 GCTCATCTTTGATTTCATCGATCAACATATCGTCGGTGTTTTTAGCCTCGTTGGCTTTTT

4 GCTCATCTTTGATTTCATCGATCAACATATCGTCGGTGTTTTTAGCCTCGTTGGCTTTTT

3 GCTCATCTTTGATTTCATCGATCAACATATCGTCGGTGTTTTTAGCCTCGTTGGCTTTTT

20 GCTCATCTTTGATTTCATCGATCAACATATCGTCGGTGTTTTTAGCCTCGTTGGCTTTTT

************************************************************

1 GTTCGAGATCGGTGCGTGTCTTGATACGCTTCCTCAATCTTCTTCTCTCCTCTTTATCTT

16 GTTCGAGATCGGTGCGTGTCTTGATACGCTTCCTCAATCTTCTTCTCTCCTCTTTATCTT

15 GTTCGAGATCGGTGCGTGTCTTGATACGCTTCCTCAATCTTCTTCTCTCATCTTTATCTT

2 GTTCGAGATCGGTGCGTGTCTTGATACGCTTCCTCAATCTTCTTCTCTCATCTTTATCTT

10 GTTCGAGATCGGTGCGTGTCTTGATACGCTTCCTCAATCTTCTTCTCTCCTCTTTATCTT

17 GTTCGAGATCGGTGCGTGTCTTGATACGCTTCCTCAATCTTCTTCTCTCCTCTTTATCTT

14 GTTCGAGATCGGTGCGTGTCTTGATACGCTTCCTCAATCTTCTTCTCTCCTCTTTATCTT

13 GTTCGAGATCGGTGCGTGTCTTGATACGCTTCCTCAATCTTCTTCTCTCCTCTTTATCTT

12 GTTCGAGATCGGTGCGTGTCTTGATACGCTTCCTCAATCTTCTTCTCTCCTCTTTATCTT

8 GTTCGAGATCGGTGCGTGTCTTGATACGCTTCCTCAATCTTCTTCTCTCCTCTTTATCTT

7 GTTCGAGATCGGTGCGTGTCTTGATACGCTTCCTCAATCTTCTTCTCTCCTCTTTATCTT

19 GTTCGAGATCGGTGCGTGTCTTGATACGCTTCCTCAATCTTCTTCTCTCCTCTTTATCTT

18 GTTCGAGATCGGTGCGTGTCTTGATACGCTTCCTCAATCTTCTTCTCTCCTCTTTATCTT

22 GTTCGAGATCGGTGCGTGTCTTGATACGCTTCCTCAATCTTCTTCTCTCCTCTTTATCTT

21 GTTCGAGATCGGTGCGTGTCTTGATACGCTTCCTCAATCTTCTTCTCTCCTCTTTATCTT

9 GTTCGAGATCGGTGCGTGTCTTGATACGCTTCCTCAATCTTCTTCTCTCCTCTTTATCTT

6 GTTCGAGATCGGTGCGTGTCTTGATACGCTTCCTCAATCTTCTTCTCTCCTCTTTATCTT

5 GTTCGAGATCGGTGCGTGTCTTGATACGCTTCCTCAATCTTCTTCTCTCCTCTTTATCTT

4 GTTCGAGATCGGTGCGTGTCTTGATACGCTTCCTCAATCTTCTTCTCTCCTCTTTATCTT

3 GTTCGAGATCGGTGCGTGTCTTGATACGCTTCCTCAATCTTCTTCTCTCCTCTTTATCTT

20 GTTCGAGATCGGTGCGTGTCTTGATACGCTTCCTCAATCTTCTTCTCTCCTCTTTATCTT

************************************************* **********

1 TGTTGATTGACAGAAGCTTCTTGTTGACCGGGTTGATCTTAGCCTTGAGATCGTCACTGA

16 TGTTGATTGACAGAAGCTTCTTGTTGACCGGGTTGATCTTAGCCTTGAGATCGTCACTGA

15 TGTTGATTGACAGAAGCTTCTTGTTGACCGGGTTGATCTTAGCCTTGAGATCGTCACTGA

2 TGTTGATTGACAGAAGCTTCTTGTTGACCGGGTTGATCTTAGCCTTGAGATCGTCACTGA

10 TGTTGATTGACAGAAGCTTCTTGTTGACCGGGTTGATCTTAGCCTTGAGATCGTCACTGA

17 TGTTGATTGACAGAAGCTTCTTGTTGACCGGGTTGATCTTAGCCTTGAGATCGTCACTGA

14 TGTTGATTGACAGAAGCTTCTTGTTGACCGGGTTGATCTTAGCCTTGAGATCGTCACTGA

13 TGTTGATTGACAGAAGCTTCTTGTTGACCGGGTTGATCTTAGCCTTGAGATCGTCACTGA

12 TGTTGATTGACAGAAGCTTCTTGTTGACCGGGTTGATCTTAGCCTTGAGATCGTCACTGA

8 TGTTGATTGACAGAAGCTTCTTGTTGACCGGGTTGATCTTAGCCTTGAGATCGTCACTGA

7 TGTTGATTGACAGAAGCTTCTTGTTGACCGGGTTGATCTTAGCCTTGAGATCGTCACTGA

19 TGTTGATTGACAGAAGCTTCTTGTTGACCGGGTTGATCTTAGCCTTGAGATCGTCACTGA

18 TGTTGATTGACAGAAGCTTCTTGTTGACCGGGTTGATCTTAGCCTTGAGATCGTCACTGA

22 TGTTGATTGACAGAAGCTTCTTGTTGACCGGGTTGATCTTAGCCTTGAGATCGTCACTGA

21 TGTTGATTGACAGAAGCTTCTTGTTGACCGGGTTGATCTTAGCCTTGAGATCGTCACTGA

9 TGTTGATTGACAGAAGCTTCTTGTTGACCGGGTTGATCTTAGCCTTGAGATCGTCACTGA

6 TGTTGATTGACAGAAGCTTCTTGTTGACCGGGTTGATCTTAGCCTTGAGATCGTCACTGA

5 TGTTGATTGACAGAAGCTTCTTGTTGACCGGGTTGATCTTAGCCTTGAGATCGTCACTGA

4 TGTTGATTGACAGAAGCTTCTTGTTGACCGGGTTGATCTTAGCCTTGAGATCGTCACTGA

3 TGTTGATTGACAGAAGCTTCTTGTTGACCGGGTTGATCTTAGCCTTGAGATCGTCACTGA

20 TGTTGATTGACAGAAGCTTCTTGTTGACCGGGTTGATCTTAGCCTTGAGATCGTCACTGA

************************************************************

1 CGAGATCACTGGCATCATAATCCATTGGGAACTTTACTTTGCGGAGGATTTTTGCCTTTT

16 CGAGATCACTGGCATCATACTCCATTGGGAACTTTACTTTGCGGAGGATTTTTGCCTTTT

15 CGAGATCACTGGCATCATACTCCATTGGGAACTTTACTTTGCGGAGGATTTTTGCCTTTT

2 CGAGATCACTGGCATCATACTCCATTGGGAACTTTACTTTGCGGAGGATTTTTGCCTTTT

10 CGAGATCACTGGCATCATACTCCATTGGGAACTTTACTTTGCGGAGGATTTTTGCCTTTT

17 CGAGATCACTGGCATCATACTCCATTGGGAACTTTACTTTGCGGAGGATTTTTGCCTTTT

14 CGAGATCACTGGCATCATACTCCATTGGGAACTTTACTTTGCGGAGGATTTTTGCCTTTT

13 CGAGATCACTGGCATCATACTCCATTGGGAACTTTACTTTGCGGAGGATTTTTGCCTTTT

12 CGAGATCACTGGCATCATACTCCATTGGGAACTTTACTTTGCGGAGGATTTTTGCCTTTT

8 CGAGATCACTGGCATCATACTCCATTGGGAACTTTACTTTGCGGAGGATTTTTGCCTTTT

7 CGAGATCACTGGCATCATACTCCATTGGGAACTTTACTTTGCGGAGGATTTTTGCCTTTT

19 CGAGATCACTGGCATCATACTCCATTGGGAACTTTACTTTGCGGAGGATTTTTGCCTTTT

18 CGAGATCACTGGCATCATACTCCATTGGGAACTTTACTTTGCGGAGGATTTTTGCCTTTT

22 CGAGATCACTGGCATCATACTCCATTGGGAACTTTACTTTGCGGAGGATTTTTGCCTTTT

21 CGAGATCACTGGCATCATACTCCATTGGGAACTTTACTTTGCGGACGATTTTTGCCTTTT

9 CGAGATCACTGGCATCATACTCCATTGGGAACTTTACTTTGCGGACGATTTTTGCCTTTT

6 CGAGATCACTGGCATCATACTCCATTGGGAACTTTACTTTGCGGACGATTTTTGCCTTTT

5 CGAGATCACTGGCATCATACTCCATTGGGAACTTTACTTTGCGGACGATTTTTGCCTTTT

4 CGAGATCACTGGCATCATACTCCATTGGGAACTTTACTTTGCGGACGATTTTTGCCTTTT

3 CGAGATCACTGGCATCATACTCCATTGGGAACTTTACTTTGCGGACGATTTTTGCCTTTT

20 CGAGATCACTGGCATCATACTCCATTGGGAACTTTACTTTGCGGACGATTTTTGCCTTTT

******************* ************************* **************

1 TGCCTAAATCAGCTCTCCAATAGAATCTGACGAGATGGACGGAGAGGTATGCTGGCAATC

16 TGCCTAAATCAGCTCTCCAATAGAATCTGACGAGATGGACGGAGAGGTATGCTGGCAATC

15 TGCCTAAATCAGCTCTCCAATAGAATCTGACGAGATGGACGGAGAGGTATGCTGGCAATC

2 TGCCTAAATCAGCTCTCCAATAGAATCTGACGAGATGGACGGAGAGGTATGCTGGCAATC

10 TGCCTAAATCAGCTCTCCAATAGAATCTGACGAGATGGACGGAGAGGTATGCTGGCAATC

17 TGCCTAAATCAGCTCTCCAATAGAATCTGACGAGATGGACGGAGAGGTATGCTGGCAATC

14 TGCCTAAATCAGCTCTCCAATAGAATCTGACGAGATGGACGGAGAGGTATGCTGGCAATC

13 TGCCTAAATCAGCTCTCCAATAGAATCTGACGAGATGGACGGAGAGGTATGCTGGCAATC

12 TGCCTAAATCAGCTCTCCAATAGAATCTGACGAGATGGACGGAGAGGTATGCTGGCAATC

8 TGCCTAAATCAGCTCTCCAATAGAATCTGACGAGATGGACGGAGAGGTATGCTGGCAATC

7 TGCCTAAATCAGCTCTCCAATAGAATCTGACGAGATGGACGGAGAGGTATGCTGGCAATC

19 TGCCTAAATCAGCTCTCCAATAGAATCTGACGAGATGGACGGAGAGGTATGCTGGCAATC

18 TGCCTAAATCAGCTCTCCAATAGAATCTGACGAGATGGACGGAGAGGTATGCTGGCAATC

22 TGCCTAAATCAGCTCTCCAATAGAATCTGACGAGATGGACGGAGAGGTATGCTGGCAATC

21 TGCCTAAATCAGCTCTCCAATAGAATCTGACGAGATGGACGGAGAGGTATGCTGGCAATC

9 TGCCTAAATCAGCTCTCCAATAGAATCTGACGAGATGGACGGAGAGGTATGCTGGCAATC

6 TGCCTAAATCAGCTCTCCAATAGAATCTGACGAGATGGACGGAGAGGTATGCTGGCAATC

5 TGCCTAAATCAGCTCTCCAATAGAATCTGACGAGATGGACGGAGAGGTATGCTGGCAATC

4 TGCCTAAATCAGCTCTCCAATAGAATCTGACGAGATGGACGGAGAGGTATGCTGGCAATC

3 TGCCTAAATCAGCTCTCCAATAGAATCTGACGAGATGGACGGAGAGGTATGCTGGCAATC

20 TGCCTAAATCAGCTCTCCAATAGAATCTGACGAGATGGACGGAGAGGTATGCTGGCAATC

************************************************************

1 TATTAATTCTCGATTTCTGTGTATATTGAGCGTTTCTATTGAGTGTTGAGGAGTGTTTTG

16 TATTAATTCTCGATTTCTGTGTATATTGAGCGTTTCTATTGAGTGTTGAGGAGTGTTTTG

15 TATTAATTCTCGATTTCTGTGTATATTGAGCGTTTCTATTGAGTGTTGAGGAGTGTTTTG

2 TATTAATTCTCGATTTCTGTGTATATTGAGCGTTTCTATTGAGTGTTGAGGAGTGTTTTG

10 TATTAATTCTCGATTTCTGTGTATATTGAGCGTTTCTATTGAGTGTTGAGGAGTGTTTTG

17 TATTAATTCTCGATTTCTGTGTATATTGAGCGTTTCTATTGAGTGTTGAGGAGTGTTTTG

14 TATTAATTCTCGATTTCTGTGTATATTGAGCGTTTCTATTGAGTGTTGAGGAGTGTTTTG

13 TATTAATTCTCGATTTCTGTGTATATTGAGCGTTTCTATTGAGTGTTGAGGAGTGTTTTG

12 TATTAATTCTCGATTTCTGTGTATATTGAGCGTTTCTATTGAGTGTTGAGGAGTGTTTTG

8 TATTAATTCTCGATTTCTGTGTATATTGAGCGTTTCTATTGAGTGTTGAGGAGTGTTTTG

7 TATTAATTCTCGATTTCTGTGTATATTGAGCGTTTCTATTGAGTGTTGAGGAGTGTTTTG

19 TATTAATTCTCGATTTCTGTGTATATTGAGCGTTTCTATTGAGTGTTGAGGAGTGTTTTG

18 TATTAATTCTCGATTTCTGTGTATATTGAGCGTTTCTATTGAGTGTTGAGGAGTGTTTTG

22 TATTAATTCTCGATTTCTGTGTATATTGAGCGTTTCTATTGAGTGTTGAGGAGTGTTTTG

21 TATTAATTCTCGATTTCTGTGTATATTGAGCGTTTCTATTGAGTGTTGAGGAGTGTTTTG

9 TATTAATTCTCGATTTCTGTGTATATTGAGCGTTTCTATTGAGTGTTGAGGAGTGTTTTG

6 TATTAATTCTCGATTTCTGTGTATATTGAGCGTTTCTATTGAGTGTTGAGGAGTGTTTTG

5 TATTAATTCTCGATTTCTGTGTATATTGAGCGTTTCTATTGAGTGTTGAGGAGTGTTTTG

4 TATTAATTCTCGATTTCTGTGTATATTGAGCGTTTCTATTGAGTGTTGAGGAGTGTTTTG

3 TATTAATTCTCGATTTCTGTGTATATTGAGCGTTTCTATTGAGTGTTGAGGAGTGTTTTG

20 TATTAATTCTCGATTTCTGTGTATATTGAGCGTTTCTATTGAGTGTTGAGGAGTGTTTTG

************************************************************

1 TAACCTGCTCATCCAAACCATCTGTTAAACCTTGCTGGAGGTAGTTGGTGGATGAAGAGA

16 TAACCTGCTCATCCAAACCATCTGTTAAACCTTGCTGGAGGTAGTTGGTGGATGAAGAGA

15 TAACCTGCTCATCCAAACCATCTGTTAAACCTTGCTGGAGGTAGTTGGTGGATGAAGAGA

2 TAACCTGCTCATCCAAACCATCTGTTAAACCTTGCTGGAGGTAGTTGGTGGATGAAGAGA

10 TAACCTGCTCATCCAAACCATCTGTTAAACCTTGCTGGAGGTAGTTGGTGGATGAAGAGA

17 TAACCTGCTCATCCAAACCATCTGTTAAACCTTGCTGGAGGTAGTTGGTGGATGAAGAGA

14 TAACCTGCTCATCCAAACCATCTGTTAAACCTTGCTGGAGGTAGTTGGTGGATGAAGAGA

13 TAACCTGCTCATCCAAACCATCTGTTAAACCTTGCTGGAGGTAGTTGGTGGATGAAGAGA

12 TAACCTGCTCATCCAAACCATCTGTTAAACCTTGCTGGAGGTAGTTGGTGGATGAAGAGA

8 TAACCTGCTCATCCAAACCATCTGTTAAACCTTGCTGGAGGTAGTTGGTGGATGAAGAGA

7 TAACCTGCTCATCCAAACCATCTGTTAAACCTTGCTGGAGGTAGTTGGTGGATGAAGAGA

19 TAACCTGCTCATCCAAACCATCTGTTAAACCTTGCTGGAGGTAGTTGGTGGATGAAGAGA

18 TAACCTGCTCATCCAAACCATCTGTTAAACCTTGCTGGAGGTAGTTGGTGGATGAAGAGA

22 TAACCTGCTCATCCAAACCATCTGTTAAACCTTGCTGGAGGTAGTTGGTGGATGAAGAGA

21 TAACCTGCTCATCCAAACCATCTGTTAAACCTTGCTGGAGGTAGTTGGTGGATGAAGAGA

9 TAACCTGCTCATCCAAACCATCTGTTAAACCTTGCTGGAGGTAGTTGGTGGATGAAGAGA

6 TAACCTGCTCATCCAAACCATCTGTTAAACCTTGCTGGAGGTAGTTGGTGGATGAAGAGA

5 TAACCTGCTCATCCAAACCATCTGTTAAACCTTGCTGGAGGTAGTTGGTGGATGAAGAGA

4 TAACCTGCTCATCCAAACCATCTGTTAAACCTTGCTGGAGGTAGTTGGTGGATGAAGAGA

3 TAACCTGCTCATCCAAACCATCTGTTAAACCTTGCTGGAGGTAGTTGGTGGATGAAGAGA

20 TAACCTGCTCATCCAAACCATCTGTTAAACCTTGCTGGAGGTAGTTGGTGGATGAAGAGA

************************************************************

1 TGTTGCAACCCAATTTAGTAAACTCTTCAATTGAGTAAGAAGGCTCTTCAAGTGTTGATT

16 TGTTGCAACCCAATTTAGTAAACTCTTCAATTGAGTAAGAAGGCTCTTCAAGTGTTGATT

15 TGTTGCAACCCAATTTAGTAAACTCTTCAATTGAGTAAGAAGGCTCTTCAAGTGTTGATT

2 TGTTGCAACCCAATTTAGTAAACTCTTCAATTGAGTAAGAAGGCTCTTCAAGTGTTGATT

10 TGTTGCAACCCAATTTAGTAAACTCTTCAATTGAGTAAGAAGGCTCTTCAAGTGTTGATT

17 TGTTGCAACCCAATTTAGTAAACTCTTCAATTGAGTAAGAAGGCTCTTCAAGTGTTGATT

14 TGTTGCAACCCAATTTAGTAAACTCTTCAATTGAGTAAGAAGGCTCTTCAAGTGTTGATT

13 TGTTGCAACCCAATTTAGTAAACTCTTCAATTGAGTAAGAAGGCTCTTCAAGTGTTGATT

12 TGTTGCAACCCAATTTAGTAAACTCTTCAATTGAGTAAGAAGGCTCTTCAAGTGTTGATT

8 TGTTGCAACCCAATTTAGTAAACTCTTCAATTGAGTAAGAAGGCTCTTCAAGTGTTGATT

7 TGTTGCAACCCAATTTAGTAAACTCTTCAATTGAGTAAGAAGGCTCTTCAAGTGTTGATT

19 TGTTGCAACCCAATTTAGTAAACTCTTCAATTGAGTAAGAAGGCTCTTCAAGTGTTGATT

18 TGTTGCAACCCAATTTAGTAAACTCTTCAATTGAGTAAGAAGGCTCTTCAAGTGTTGATT

22 TGTTGCAACCCAATTTAGTAAACTCTTCAATTGAGTAAGAAGGCTCTTCAAGTGTTGATT

21 TGTTGCAACCCAATTTAGTAAACTCTTCAATTGAGTAAGAAGGCTCTTCAAGTGTTGATT

9 TGTTGCAACCCAATTTAGTAAACTCTTCAATTGAGTAAGAAGGCTCTTCAAGTGTTGATT

6 TGTTGCAACCCAATTTAGTAAACTCTTCAATTGAGTAAGAAGGCTCTTCAAGTGTTGATT

5 TGTTGCAACCCAATTTAGTAAACTCTTCAATTGAGTAAGAAGGCTCTTCAAGTGTTGATT

4 TGTTGCAACCCAATTTAGTAAACTCTTCAATTGAGTAAGAAGGCTCTTCAAGTGTTGATT

3 TGTTGCAACCCAATTTAGTAAACTCTTCAATTGAGTAAGAAGGCTCTTCAAGTGTTGATT

20 TGTTGCAACCCAATTTAGTAAACTCTTCAATTGAGTAAGAAGGCTCTTCAAGTGTTGATT

************************************************************

1 CAGGCGTAGACATCTCTTTCACGAAAGTACCCTTCATGTATTGATCAACGAATGATTTAC

16 CAGGCGTAGACATCTCTTTCACGAAAGTACCCTTCATGTATTGATCAACGAATGATTTAC

15 CAGGCGTAGACATCTCTTTCACGAAAGTACCCTTCATGTATTGATCAACGAATGATTTAC

2 CAGGCGTAGACATCTCTTTCACGAAAGTACCCTTCATGTATTGATCAACGAATGATTTAC

10 CAGGCGTAGACATCTCTTTCACGAAAGTACCCTTCATGTATTGATCAACGAATGATTTAC

17 CAGGCGTAGACATCTCTTTCACGAAAGTACCCTTCATGTATTGATCAACGAATGATTTAC

14 CAGGCGTAGACATCTCTTTCACGAAAGTACCCTTCATGTATTGATCAACGAATGATTTAC

13 CAGGCGTAGACATCTCTTTCACGAAAGTACCCTTCATGTATTGATCAACGAATGATTTAC

12 CAGGCGTAGACATCTCTTTCACGAAAGTACCCTTCATGTATTGATCAACGAATGATTTAC

8 CAGGCGTAGACATCTCTTTCACGAAAGTACCCTTCATGTATTGATCAACGAATGATTTAC

7 CAGGCGTAGACATCTCTTTCACGAAAGTACCCTTCATGTATTGATCAACGAATGATTTAC

19 CAGGCGTAGACATCTCTTTCACGAAAGTACCCTTCATGTATTGATCAACGAATGATTTAC

18 CAGGCGTAGACATCTCTTTCACGAAAGTACCCTTCATGTATTGATCAACGAATGATTTAC

22 CAGGCGTAGACATCTCTTTCACGAAAGTACCCTTCATGTATTGATCAACGAATGATTTAC

21 CAGGCGTAGACATCTCTTTCACGAAAGTACCCTTCATGTATTGATCAACGAATGATTTAC

9 CAGGCGTAGACATCTCTTTCACGAAAGTACCCTTCATGTATTGATCAACGAATGATTTAC

6 CAGGCGTAGACATCTCTTTCACGAAAGTACCCTTCATGTATTGATCAACGAATGATTTAC

5 CAGGCGTAGACATCTCTTTCACGAAAGTACCCTTCATGTATTGATCAACGAATGATTTAC

4 CAGGCGTAGACATCTCTTTCACGAAAGTACCCTTCATGTATTGATCAACGAATGATTTAC

3 CAGGCGTAGACATCTCTTTCACGAAAGTACCCTTCATGTATTGATCAACGAATGATTTAC

20 CAGGCGTAGACATCTCTTTCACGAAAGTACCCTTCATGTATTGATCAACGAATGATTTAC

************************************************************

1 CCTGTGACGTCATCAACTCTGATCCCAAAGAGTTGAGGATTGCGCCCCAAGCTTCTTCAG

16 CCTGTGACGTCATCAACTCTGATCCCAAAGAGTTGAGGATTGCGCCCCAAGCTTCTTCAG

15 CCTGTGACGTCATCAACTCTGATCCCAAAGAGTTGAGGATTGCGCCCCAAGCTTCTTCAG

2 CCTGTGACGTCATCAACTCTGATCCCAAAGAGTTGAGGATTGCGCCCCAAGCTTCTTCAG

10 CCTGTGACGTCATCAACTCTGATCCCAAAGAGTTGAGGATTGCGCCCCAAGCTTCTTCAG

17 CCTGTGACGTCATCAACTCTGATCCCAAAGAGTTGAGGATTGCGCCCCAAGCTTCTTCAG

14 CCTGTGACGTCATCAACTCTGATCCCAAAGAGTTGAGGATTGCGCCCCAAGCTTCTTCAG

13 CCTGTGACGTCATCAACTCTGATCCCAAAGAGTTGAGGATTGCGCCCCAAGCTTCTTCAG

12 CCTGTGACGTCATCAACTCTGATCCCAAAGAGTTGAGGATTGCGCCCCAAGCTTCTTCAG

8 CCTGTGACGTCATCAACTCTGATCCCAAAGAGTTGAGGATTGCGCCCCAAGCTTCTTCAG

7 CCTGTGACGTCATCAACTCTGATCCCAAAGAGTTGAGGATTGCGCCCCAAGCTTCTTCAG

19 CCTGTGACGTCATCAACTCTGATCCCAAAGAGTTGAGGATTGCGCCCCAAGCTTCTTCAG

18 CCTGTGACGTCATCAACTCTGATCCCAAAGAGTTGAGGATTGCGCCCCAAGCTTCTTCAG

22 CCTGTGACGTCATCAACTCTGATCCCAAAGAGTTGAGGATTGCGCCCCAAGCTTCTTCAG

21 CCTGTGACGTCATCAACTCTGATCCCAAAGAGTTGAGGATTGCGCCCCAAGCTTCTTCAG

9 CCTGTGACGTCATCAACTCTGATCCCAAAGAGTTGAGGATTGCGCCCCAAGCTTCTTCAG

6 CCTGTGACGTCATCAACTCTGATCCCAAAGAGTTGAGGATTGCGCCCCAAGCTTCTTCAG

5 CCTGTGACGTCATCAACTCTGATCCCAAAGAGTTGAGGATTGCGCCCCAAGCTTCTTCAG

4 CCTGTGACGTCATCAACTCTGATCCCAAAGAGTTGAGGATTGCGCCCCAAGCTTCTTCAG

3 CCTGTGACGTCATCAACTCTGATCCCAAAGAGTTGAGGATTGCGCCCCAAGCTTCTTCAG

20 CCTGTGACGTCATCAACTCTGATCCCAAAGAGTTGAGGATTGCGCCCCAAGCTTCTTCAG

************************************************************

1 CGTCTTGTTGAGCGAACTGACCGTATCTATCAGTTTCAGTGAATTGTTGGTAAGAGGATC

16 CGTCTTGTTGAGCGAACTGACCGTATCTATCAGTTTCAGTGAATTGTTGGTAAGAGGATC

15 CGTCTTGTTGAGCGAACTGACCGTATCTATCAGTTTCAGTGAATTGTTGGTAAGAGGATC

2 CGTCTTGTTGAGCGAACTGACCGTATCTATCAGTTTCAGTGAATTGTTGGTAAGAGGATC

10 CGTCTTGTTGAGCGAACTGACCGTATCTATCAGTTTCAGTGAATTGTTGGTAAGAGGATC

17 CGTCTTGTTGAGCGAACTGACCGTATCTATCAGTTTCAGTGAATTGTTGGTAAGAGGATC

14 CGTCTTGTTGAGCGAACTGACCGTATCTATCAGTTTCAGTGAATTGTTGGTAAGAGGATC

13 CGTCTTGTTGAGCGAACTGACCGTATCTATCAGTTTCAGTGAATTGTTGGTAAGAGGATC

12 CGTCTTGTTGAGCGAACTGACCGTATCTATCAGTTTCAGTGAATTGTTGGTAAGAGGATC

8 CGTCTTGTTGAGCGAACTGACCGTATCTATCAGTTTCAGTGAATTGTTGGTAAGAGGATC

7 CGTCTTGTTGAGCGAACTGACCGTATCTATCAGTTTCAGTGAATTGTTGGTAAGAGGATC

19 CGTCTTGTTGAGCGAACTGACCGTATCTATCAGTTTCAGTGAATTGTTGGTAAGAGGATC

18 CGTCTTGTTGAGCGAACTGACCGTATCTATCAGTTTCAGTGAATTGTTGGTAAGAGGATC

22 CGTCTTGTTGAGCGAACTGACCGTATCTATCAGTTTCAGTGAATTGTTGGTAAGAGGATC

21 CGTCTTGTTGAGCGAACTGACCGTATCTATCAGTTTCAGTGAATTGTTGGTAAGAGGATC

9 CGTCTTGTTGAGCGAACTGACCGTATCTATCAGTTTCAGTGAATTGTTGGTAAGAGGATC

6 CGTCTTGTTGAGCGAACTGACCGTATCTATCAGTTTCAGTGAATTGTTGGTAAGAGGATC

5 CGTCTTGTTGAGCGAACTGACCGTATCTATCAGTTTCAGTGAATTGTTGGTAAGAGGATC

4 CGTCTTGTTGAGCGAACTGACCGTATCTATCAGTTTCAGTGAATTGTTGGTAAGAGGATC

3 CGTCTTGTTGAGCGAACTGACCGTATCTATCAGTTTCAGTGAATTGTTGGTAAGAGGATC

20 CGTCTTGTTGAGCGAACTGACCGTATCTATCAGTTTCAGTGAATTGTTGGTAAGAGGATC

************************************************************

1 TTAGCATTGTGAGGAATGCAGTTGGAGTGATTGCGTCAGTTGAGTTGTTGAGCGAGGAAA

16 TTAGCATTGTGAGGAATGCAGTTGGAGTGATTGCGTCAGTTGAGTTGTTGAGCGAGGAAA

15 TTAGCATTGTGAGGAATGCAGTTGGAGTGATTGCGTCAGTTGAGTTGTTGAGCGAGGAAA

2 TTAGCATTGTGAGGAATGCAGTTGGAGTGATTGCGTCAGTTGAGTTGTTGAGCGAGGAAA

10 TTAGCATTGTGAGGAATGCAGTTGGAGTGATTGCGTCAGTTGAGTTGTTGAGCGAGGAAA

17 TTAGCATTGTGAGGAATGCAGTTGGAGTGATTGCGTCAGTTGAGTTGTTGAGCGAGGAAA

14 TTAGCATTGTGAGGAATGCAGTTGGAGTGATTGCGTCAGTTGAGTTGTTGAGCGAGGAAA

13 TTAGCATTGTGAGGAATGCAGTTGGAGTGATTGCGTCAGTTGAGTTGTTGAGCGAGGAAA

12 TTAGCATTGTGAGGAATGCAGTTGGAGTGATTGCGTCAGTTGAGTTGTTGAGCGAGGAAA

8 TTAGCATTGTGAGGAATGCAGTTGGAGTGATTGCGTCAGTTGAGTTGTTGAGCGAGGAAA

7 TTAGCATTGTGAGGAATGCAGTTGGAGTGATTGCGTCAGTTGAGTTGTTGAGCGAGGAAA

19 TTAGCATTGTGAGGAATGCAGTTGGAGTGATTGCGTCAGTTGAGTTGTTGAGCGAGGAAA

18 TTAGCATTGTGAGGAATGCAGTTGGAGTGATTGCGTCAGTTGAGTTGTTGAGCGAGGAAA

22 TTAGCATTGTGAGGAATGCAGTTGGAGTGATTGCGTCAGTTGAGTTGTTGAGCGAGGAAA

21 TTAGCATTGTGAGGAATGCAGTTGGAGTGATTGCGTCAGTAGAGTTGTTGAGCGAGGAAA

9 TTAGCATTGTGAGGAATGCAGTTGGAGTGATTGCGTCAGTAGAGTTGTTGAGCGAGGAAA

6 TTAGCATTGTGAGGAATGCAGTTGGAGTGATTGCGTCAGTAGAGTTGTTGAGCGAGGAAA

5 TTAGCATTGTGAGGAATGCAGTTGGAGTGATTGCGTCAGTAGAGTTGTTGAGCGAGGAAA

4 TTAGCATTGTGAGGAATGCAGTTGGAGTGATTGCGTCAGTAGAGTTGTTGAGCGAGGAAA

3 TTAGCATTGTGAGGAATGCAGTTGGAGTGATTGCGTCAGTAGAGTTGTTGAGCGAGGAAA

20 TTAGCATTGTGAGGAATGCAGTTGGAGTGATTGCGTCAGTAGAGTTGTTGAGCGAGGAAA

**************************************** *******************

1 AGAGGGATTGAAGGGAGCGTAATAAGCCAGAGTTTTGTGTGTTACTCAGAGTGCTCTGTA

16 AGAGGGATTGAAGGGAGCGTAATAAGCCAGAGTTTTGTGTGTTACTCAGAGTGCTCTGTA

15 AGAGGGATTGAAGGGAGCGTAATAAGCCAGAGTTTTGTGTGTTACTCAGAGTGCTCTGTA

2 AGAGGGATTGAAGGGAGCGTAATAAGCCAGAGTTTTGTGTGTTACTCAGAGTGCTCTGTA

10 AGAGGGATTGAAGGGAGCGTAATAAGCCAGAGTTTTGTGTGTTACTCAGAGTGCTCTGTA

17 AGAGGGATTGAAGGGAGCGTAATAAGCCAGAGTTTTGTGTGTTACTCAGAGTGCTCTGTA

14 AGAGGGATTGAAGGGAGCGTAATAAGCCAGAGTTTTGTGTGTTACTCAGAGTGCTCTGTA

13 AGAGGGATTGAAGGGAGCGTAATAAGCCAGAGTTTTGTGTGTTACTCAGAGTGCTCTGTA

12 AGAGGGATTGAAGGGAGCGTAATAAGCCAGAGTTTTGTGTGTTACTCAGAGTGCTCTGTA

8 AGAGGGATTGAAGGGAGCGTAATAAGCCAGAGTTTTGTGTGTTACTCAGAGTGCTCTGTA

7 AGAGGGATTGAAGGGAGCGTAATAAGCCAGAGTTTTGTGTGTTACTCAGAGTGCTCTGTA

19 AGAGGGATTGAAGGGAGCGTAATAAGCCAGAGTTTTGTGTGTTACTCAGAGTGCTCTGTA

18 AGAGGGATTGAAGGGAGCGTAATAAGCCAGAGTTTTGTGTGTTACTCAGAGTGCTCTGTA

22 AGAGGGATTGAAGGGAGCGTAATAAGCCAGAGTTTTGTGTGTTACTCAGAGTGCTCTGTA

21 AGAGGGATTGAAGGGAGCGTAATAAGCCAGAGTTTTGTGTGTTACTCAGAGTGCTCTGTA

9 AGAGGGATTGAAGGGAGCGTAATAAGCCAGAGTTTTGTGTGTTACTCAGAGTGCTCTGTA

6 AGAGGGATTGAAGGGAGCGTAATAAGCCAGAGTTTTGTGTGTTACTCAGAGTGCTCTGTA

5 AGAGGGATTGAAGGGAGCGTAATAAGCCAGAGTTTTGTGTGTTACTCAGAGTGCTCTGTA

4 AGAGGGATTGAAGGGAGCGTAATAAGCCAGAGTTTTGTGTGTTACTCAGAGTGCTCTGTA

3 AGAGGGATTGAAGGGAGCGTAATAAGCCAGAGTTTTGTGTGTTACTCAGAGTGCTCTGTA

20 AGAGGGATTGAAGGGAGCGTAATAAGCCAGAGTTTTGTGTGTTACTCAGAGTGCTCTGTA

************************************************************

1 GCTCAGGTATGGCTCTGAGAACTTGAACTGATGAGTTTAAATAACAAGTGTTACCGAGAT

16 GCTCAGGTATGGCTCTGAGAACTTGAACTGATGAGTTTAAATAACAAGTGTTACCGAGAT

15 GCTCAGGTATGGCTCTGAGAACTTGAACTGATGAGTTTAAATAACAAGTGTTACCGAGAT

2 GCTCAGGTATGGCTCTGAGAACTTGAACTGATGAGTTTAAATAACAAGTGTTACCGAGAT

10 GCTCAGGTATGGCTCTGAGAACTTGAACTGATGAGTTTAAATAACAAGTGTTACCGAGAT

17 GCTCAGGTATGGCTCTGAGAACTTGAACTGATGAGTTTAAATAACAAGTGTTACCGAGAT

14 GCTCAGGTATGGCTCTGAGAACTTGAACTGATGAGTTTAAATAACAAGTGTTACCGAGAT

13 GCTCAGGTATGGCTCTGAGAACTTGAACTGATGAGTTTAAATAACAAGTGTTACCGAGAT

12 GCTCAGGTATGGCTCTGAGAACTTGAACTGATGAGTTTAAATAACAAGTGTTACCGAGAT

8 GCTCAGGTATGGCTCTGAGAACTTGAACTGATGAGTTTAAATAACAAGTGTTACCGAGAT

7 GCTCAGGTATGGCTCTGAGAACTTGAACTGATGAGTTTAAATAACAAGTGTTACCGAGAT

19 GCTCAGGTATGGCTCTGAGAACTTGAACTGATGAGTTTAAATAACAAGTGTTACCGAGAT

18 GCTCAGGTATGGCTCTGAGAACTTGAACTGATGAGTTTAAATAACAAGTGTTACCGAGAT

22 GCTCAGGTATGGCTCTGAGAACTTGAACTGATGAGTTTAAATAACAAGTGTTACCGAGAT

21 GCTCAGGTATGGCTCTGAGAACTTGAACTGATGAGTTTAAATAACAAGTGTTACCGAGAT

9 GCTCAGGTATGGCTCTGAGAACTTGAACTGATGAGTTTAAATAACAAGTGTTACCGAGAT

6 GCTCAGGTATGGCTCTGAGAACTTGAACTGATGAGTTTAAATAACAAGTGTTACCGAGAT

5 GCTCAGGTATGGCTCTGAGAACTTGAACTGATGAGTTTAAATAACAAGTGTTACCGAGAT

4 GCTCAGGTATGGCTCTGAGAACTTGAACTGATGAGTTTAAATAACAAGTGTTACCGAGAT

3 GCTCAGGTATGGCTCTGAGAACTTGAACTGATGAGTTTAAATAACAAGTGTTACCGAGAT

20 GCTCAGGTATGGCTCTGAGAACTTGAACTGATGAGTTTAAATAACAAGTGTTACCGAGAT

************************************************************

1 TAGTCAAACCGACTGGGTTTTTCAAGGCTTGCGATAAATCCTTCTCACTGAGGTCTTCCA

16 TAGTCAAACCGACTGGGTTTTTCAAGGCTTGCGATAAATCCTTCTCACTGAGGTCTTCCA

15 TAGTCAAACCGACTGGGTTTTTCAAGGCTTGCGATAAATCCTTCTCACTGAGGTCTTCCA

2 TAGTCAAACCGACTGGGTTTTTCAAGGCTTGCGATAAATCCTTCTCACTGAGGTCTTCCA

10 TAGTCAAACCGACTGGGTTTTTCAAGGCTTGCGATAAATCCTTCTCACTGAGGTCTTCCA

17 TAGTCAAACCGACTGGGTTTTTCAAGGCTTGCGATAAATCCTTCTCACTGAGGTCTTCCA

14 TAGTCAAACCGACTGGGTTTTTCAAGGCTTGCGATAAATCCTTCTCACTGAGGTCTTCCA

13 TAGTCAAACCGACTGGGTTTTTCAAGGCTTGCGATAAATCCTTCTCACTGAGGTCTTCCA

12 TAGTCAAACCGACTGGGTTTTTCAAGGCTTGCGATAAATCCTTCTCACTGAGGTCTTCCA

8 TAGTCAAACCGACTGGGTTTTTCAAGGCTTGCGATAAATCCTTCTCACTGAGGTCTTCCA

7 TAGTCAAACCGACTGGGTTTTTCAAGGCTTGCGATAAATCCTTCTCACTGAGGTCTTCCA

19 TAGTCAAACCGACTGGGTTTTTCAAGGCTTGCGATAAATCCTTCTCACTGAGGTCTTCCA

18 TAGTCAAACCGACTGGGTTTTTCAAGGCTTGCGATAAATCCTTCTCACTGAGGTCTTCCA

22 TAGTCAAACCGACTGGGTTTTTCAAGGCTTGCGATAAATCCTTCTCACTGAGGTCTTCCA

21 TAGTCAAACCGACTGGGTTTTTCAAGGCTTGCGATAAATCCTTCTCACTGAGGTCTTCCA

9 TAGTCAAACCGACTGGGTTTTTCAAGGCTTGCGATAAATCCTTCTCACTGAGGTCTTCCA

6 TAGTCAAACCGACTGGGTTTTTCAAGGCTTGCGATAAATCCTTCTCACTGAGGTCTTCCA

5 TAGTCAAACCGACTGGGTTTTTCAAGGCTTGCGATAAATCCTTCTCACTGAGGTCTTCCA

4 TAGTCAAACCGACTGGGTTTTTCAAGGCTTGCGATAAATCCTTCTCACTGAGGTCTTCCA

3 TAGTCAAACCGACTGGGTTTTTCAAGGCTTGCGATAAATCCTTCTCACTGAGGTCTTCCA

20 TAGTCAAACCGACTGGGTTTTTCAAGGCTTGCGATAAATCCTTCTCACTGAGGTCTTCCA

************************************************************

1 TGAAAACTACTTTTTGATTTGGAGCTTCTGGTAGAGGCCCTGCAGCGCCAATGACGATGA

16 TGAAAACTACTTTTTGATTTGGAGCTTCTGGTAGAGGCCCTGCAGCGCCAATGACGATGA

15 TGAAAACTACTTTTTGATTTGGAGCTTCGGGTAGAGGCCCTGCAGCGCCAATGACGATGA

2 TGAAAACTACTTTTTGATTTGGAGCTTCGGGTAGAGGCCCTGCAGCGCCAATGACGATGA

10 TGAAAACTACTTTTTGATTTGGAGCTTCTGGTAGAGGCCCTGCAGCGCCAATGACGATGA

17 TGAAAACTACTTTTTGATTTGGAGCTTCTGGTAGAGGCCCTGCAGCGCCAATGACGATGA

14 TGAAAACTACTTTTTGATTTGGAGCTTCTGGTAGAGGCCCTGCAGCGCCAATGACGATGA

13 TGAAAACTACTTTTTGATTTGGAGCTTCTGGTAGAGGCCCTGCAGCGCCAATGACGATGA

12 TGAAAACTACTTTTTGATTTGGAGCTTCTGGTAGAGGCCCTGCAGCGCCAATGACGATGA

8 TGAAAACTACTTTTTGATTTGGAGCTTCTGGTAGAGGCCCTGCAGCGCCAATGACGATGA

7 TGAAAACTACTTTTTGATTTGGAGCTTCTGGTAGAGGCCCTGCAGCGCCAATGACGATGA

19 TGAAAACTACTTTTTGATTTGGAGCTTCTGGTAGAGGCCCTGCAGCGCCAATGACGATGA

18 TGAAAACTACTTTTTGATTTGGAGCTTCTGGTAGAGGCCCTGCAGCGCCAATGACGATGA

22 TGAAAACTACTTTTTGATTTGGAGCTTCTGGTAGAGGCCCTGCAGCGCCAATGACGATGA

21 TGAAAACTACTTTTTGATTTGGAGCTTCTGGTAGAGGCCCTGCAGCGCCAATGACGATGA

9 TGAAAACTACTTTTTGATTTGGAGCTTCTGGTAGAGGCCCTGCAGCGCCAATGACGATGA

6 TGAAAACTACTTTTTGATTTGGAGCTTCTGGTAGAGGCCCTGCAGCGCCAATGACGATGA

5 TGAAAACTACTTTTTGATTTGGAGCTTCTGGTAGAGGCCCTGCAGCGCCAATGACGATGA

4 TGAAAACTACTTTTTGATTTGGAGCTTCTGGTAGAGGCCCTGCAGCGCCAATGACGATGA

3 TGAAAACTACTTTTTGATTTGGAGCTTCTGGTAGAGGCCCTGCAGCGCCAATGACGATGA

20 TGAAAACTACTTTTTGATTTGGAGCTTCTGGTAGAGGCCCTGCAGCGCCAATGACGATGA

**************************** *******************************

1 ACAAGTGACCAGGCTTGATCGATGATCCTAACTTTTCCATATCCGTATTATCCTTGAGCA

16 ACAAGTGACCAGGCTTGATCGATGATCCTAACTTTTCCATATCCGTATTATCCTTGAGCA

15 ACAAGTGACCAGGCTTGATCGATGATCCTAACTTTTCCATATCCGTATTATCCTTGAGCA

2 ACAAGTGACCAGGCTTGATCGATGATCCTAACTTTTCCATATCCGTATTATCCTTGAGCA

10 ACAAGTGACCAGGCTTGATCGATGATCCTAACTTTTCCATATCCGTATTATCCTTGAGCA

17 ACAAGTGACCAGGCTTGATCGATGATCCTAACTTTTCCATATCCGTATTATCCTTGAGCA

14 ACAAGTGACCAGGCTTGATCGATGATCCTAACTTTTCCATATCCGTATTATCCTTGAGCA

13 ACAAGTGACCAGGCTTGATCGATGATCCTAACTTTTCCATATCCGTATTATCCTTGAGCA

12 ACAAGTGACCAGGCTTGATCGATGATCCTAACTTTTCCATATCCGTATTATCCTTGAGCA

8 ACAAGTGACCAGGCTTGATCGATGATCCTAACTTTTCCATATCCGTATTATCCTTGAGCA

7 ACAAGTGACCAGGCTTGATCGATGATCCTAACTTTTCCATATCCGTATTATCCTTGAGCA

19 ACAAGTGACCAGGCTTGATCGATGATCCTAACTTTTCCATATCCGTATTATCCTTGAGCA

18 ACAAGTGACCAGGCTTGATCGATGATCCTAACTTTTCCATATCCGTATTATCCTTGAGCA

22 ACAAGTGACCAGGCTTGATCGATGATCCTAACTTTTCCATATCCGTATTATCCTTGAGCA

21 ACAAGTGACCAGGCTTGATCGATGATCCTAACTTTTCCATATCCGTATTATCCTTGAGCA

9 ACAAGTGACCAGGCTTGATCGATGATCCTAACTTTTCCATATCCGTATTATCCTTGAGCA

6 ACAAGTGACCAGGCTTGATCGATGATCCTAACTTTTCCATATCCGTATTATCCTTGAGCA

5 ACAAGTGACCAGGCTTGATCGATGATCCTAACTTTTCCATATCCGTATTATCCTTGAGCA

4 ACAAGTGACCAGGCTTGATCGATGATCCTAACTTTTCCATATCCGTATTATCCTTGAGCA

3 ACAAGTGACCAGGCTTGATCGATGATCCTAACTTTTCCATATCCGTATTATCCTTGAGCA

20 ACAAGTGACCAGGCTTGATCGATGATCCTAACTTTTCCATATCCGTATTATCCTTGAGCA

************************************************************

1 TTCCACCTTTAATCATAACTTTCTGCCTGTCGAGGATGTTAAGGGGGAATGCCACAGTGA

16 TTCCACCTTTAATCATAACTTTCTGCCTGTCGAGGATGTTAAGGGTGAATGCCACAGTGA

15 TTCCACCTTTAATCATAACTTTCTGCCTGTCGAGGATGTTAAGGGTGAATGCCACAGTGA

2 TTCCACCTTTAATCATAACTTTCTGCCTGTCGAGGATGTTAAGGGTGAATGCCACAGTGA

10 TTCCACCTTTAATCATAACTTTCTGCCTGTCGAGGATGTTAAGGGGGAATGCCACAGTGA

17 TTCCACCTTTAATCATAACTTTCTGCCTGTCGAGGATGTTAAGGGGGAATGCCACAGTGA

14 TTCCACCTTTAATCATAACTTTCTGCCTGTCGAGGATGTTAAGGGGGAATGCCACAGTGA

13 TTCCACCTTTAATCATAACTTTCTGCCTGTCGAGGATGTTAAGGGGGAATGCCACAGTGA

12 TTCCACCTTTAATCATAACTTTCTGCCTGTCGAGGATGTTAAGGGGGAATGCCACAGTGA

8 TTCCACCTTTAATCATAACTTTCTGCCTGTCGAGGATGTTAAGGGGGAATGCCACAGTGA

7 TTCCACCTTTAATCATAACTTTCTGCCTGTCGAGGATGTTAAGGGGGAATGCCACAGTGA

19 TTCCACCTTTAATCATAACTTTCTGCCTGTCGAGGATGTTAAGGGGGAATGCCACAGTGA

18 TTCCACCTTTAATCATAACTTTCTGCCTGTCGAGGATGTTAAGGGGGAATGCCACAGTGA

22 TTCCACCTTTAATCATAACTTTCTGCCTGTCGAGGATGTTAAGGGGGAATGCCACAGTGA

21 TTCCACCTTTAATCATAACTTTCTGCCTGTCGAGGATGTTAAGGGGGAATGCCACAGTGA

9 TTCCACCTTTAATCATAACTTTCTGCCTGTCGAGGATGTTAAGGGGGAATGCCACAGTGA

6 TTCCACCTTTAATCATAACTTTCTGCCTGTCGAGGATGTTAAGGGGGAATGCCACAGTGA

5 TTCCACCTTTAATCATAACTTTCTGCCTGTCGAGGATGTTAAGGGGGAATGCCACAGTGA

4 TTCCACCTTTAATCATAACTTTCTGCCTGTCGAGGATGTTAAGGGGGAATGCCACAGTGA

3 TTCCACCTTTAATCATAACTTTCTGCCTGTCGAGGATGTTAAGGGGGAATGCCACAGTGA

20 TTCCACCTTTAATCATAACTTTCTGCCTGTCGAGGATGTTAAGGGGGAATGCCACAGTGA

********************************************* **************

1 TACGTAGTGTGACCCACCTATCTGGAGCCACGCCAGTTAGAGAATGAATTTGATTCTTGA

16 TACGTAGTGTGACCCACCTATCTGGAGCCACGCCAGTTAGAGAATGAATTTGATTCTTGA

15 TACGTAGTGTGACCCACCTATCTGGAGCCACGCCAGTTAGAGAATGAATTTGATTCTTGA

2 TACGTAGTGTGACCCACCTATCTGGAGCCACGCCAGTTAGAGAATGAATTTGATTCTTGA

10 TACGTAGTGTGACCCACCTATCTGGAGCCACGCCAGTTAGAGAATGAATTTGATTCTTGA

17 TACGTAGTGTGACCCACCTATCTGGAGCCACGCCAGTTAGAGAATGAATTTGATTCTTGA

14 TACGTAGTGTGACCCACCTATCTGGAGCCACGCCAGTTAGAGAATGAATTTGATTCTTGA

13 TACGTAGTGTGACCCACCTATCTGGAGCCACGCCAGTTAGAGAATGAATTTGATTCTTGA

12 TACGTAGTGTGACCCACCTATCTGGAGCCACGCCAGTTAGAGAATGAATTTGATTCTTGA

8 TACGTAGTGTGACCCACCTATCTGGAGCCACGCCAGTTAGAGAATGAATTTGATTCTTGA

7 TACGTAGTGTGACCCACCTATCTGGAGCCACGCCAGTTAGAGAATGAATTTGATTCTTGA

19 TACGTAGTGTGACCCACCTATCTGGAGCCACGCCAGTTAGAGAATGAATTTGATTCTTGA

18 TACGTAGTGTGACCCACCTATCTGGAGCCACGCCAGTTAGAGAATGAATTTGATTCTTGA

22 TACGTAGTGTGACCCACCTATCTGGAGCCACGCCAGTTAGAGAATGAATTTGATTCTTGA

21 TACGTAGTGTGACCCACCTATCTGGAGCCACGCCAGTTAGAGAATGAATTTGATTCTTGA

9 TACGTAGTGTGACCCACCTATCTGGAGCCACGCCAGTTAGAGAATGAATTTGATTCTTGA

6 TACGTAGTGTGACCCACCTATCTGGAGCCACGCCAGTTAGAGAATGAATTTGATTCTTGA

5 TACGTAGTGTGACCCACCTATCTGGAGCCACGCCAGTTAGAGAATGAATTTGATTCTTGA

4 TACGTAGTGTGACCCACCTATCTGGAGCCACGCCAGTTAGAGAATGAATTTGATTCTTGA

3 TACGTAGTGTGACCCACCTATCTGGAGCCACGCCAGTTAGAGAATGAATTTGATTCTTGA

20 TACGTAGTGTGACCCACCTATCTGGAGCCACGCCAGTTAGAGAATGAATTTGATTCTTGA

************************************************************

1 AGTCTAAACCGGTGTACTGAGTGTTGAATTCCAAATCGAATTTCTTTCCTGCGTGTTTAA

16 AGTCTAAACCGGTGTACTGAGTGTTGAATTCCAAATCGAATTTCTTTCCTGCGTGTTTAA

15 AGTCTAAACCGGTGTACTGAGTGTTGAATTCCAAATCGAATTTCTTTCCTGCGTGTTTAA

2 AGTCTAAACCGGTGTACTGAGTGTTGAATTCCAAATCGAATTTCTTTCCTGCGTGTTTAA

10 AGTCTAAACCGGTGTACTGAGTGTTGAATTCCAAATCGAATTTCTTTCCTGCGTGTTTAA

17 AGTCTAAACCGGTGTACTGAGTGTTGAATTCCAAATCGAATTTCTTTCCTGCGTGTTTAA

14 AGTCTAAACCGGTGTACTGAGTGTTGAATTCCAAATCGAATTTCTTTCCTGCGTGTTTAA

13 AGTCTAAACCGGTGTACTGAGTGTTGAATTCCAAATCGAATTTCTTTCCTGCGTGTTTAA

12 AGTCTAAACCGGTGTACTGAGTGTTGAATTCCAAATCGAATTTCTTTCCTGCGTGTTTAA

8 AGTCTAAACCGGTGTACTGAGTGTTGAATTCCAAATCGAATTTCTTTCCTGCGTGTTTAA

7 AGTCTAAACCGGTGTACTGAGTGTTGAATTCCAAATCGAATTTCTTTCCTGCGTGTTTAA

19 AGTCTAAACCGGTGTACTGAGTGTTGAATTCCAAATCGAATTTCTTTCCTGCGTGTTTAA

18 AGTCTAAACCGGTGTACTGAGTGTTGAATTCCAAATCGAATTTCTTTCCTGCGTGTTTAA

22 AGTCTAAACCGGTGTACTGAGTGTTGAATTCCAAATCGAATTTCTTTCCTGCGTGTTTAA

21 AGTCTAAACCGGTGTACTGAGTGTTGAATTCCAAATCGAATTTCTTTCCTGCGTGTTTAA

9 AGTCTAAACCGGTGTACTGAGTGTTGAATTCCAAATCGAATTTCTTTCCTGCGTGTTTAA

6 AGTCTAAACCGGTGTACTGAGTGTTGAATTCCAAATCGAATTTCTTTCCTGCGTGTTTAA

5 AGTCTAAACCGGTGTACTGAGTGTTGAATTCCAAATCGAATTTCTTTCCTGCGTGTTTAA

4 AGTCTAAACCGGTGTACTGAGTGTTGAATTCCAAATCGAATTTCTTTCCTGCGTGTTTAA

3 AGTCTAAACCGGTGTACTGAGTGTTGAATTCCAAATCGAATTTCTTTCCTGCGTGTTTAA

20 AGTCTAAACCGGTGTACTGAGTGTTGAATTCCAAATCGAATTTCTTTCCTGCGTGTTTAA

************************************************************

1 TTGACACTGGGAATTTAGTCATCCTGGGTGTTGAGGAGAGAAAGTGAAAGGAAGGCGTGG

16 TTGACACTGGGAATTTAGTCATCCTGGGTGTTGAGGAGAGAAAGTGAAAGGAAGGCGTGG

15 TTGACACTGGGAATTTAGTCATCCTGGGTGTTGAGGAGAGAAAGTGAAAGGAAGGCGTGG

2 TTGACACTGGGAATTTAGTCATCCTGGGTGTTGAGGAGAGAAAGTGAAAGGAAGGCGTGG

10 TTGACACTGGGAATTTAGTCATCCTGGGTGTTGAGGAGAGAAAGTGAAAGGAAGGCGTGG

17 TTGACACTGGGAATTTAGTCATCCTGGGTGTTGAGGAGAGAAAGTGAAAGGAAGGCGTGG

14 TTGACACTGGGAATTTAGTCATCCTGGGTGTTGAGGAGAGAAAGTGAAAGGAAGGCGTGG

13 TTGACACTGGGAATTTAGTCATCCTGGGTGTTGAGGAGAGAAAGTGAAAGGAAGGCGTGG

12 TTGACACTGGGAATTTAGTCATCCTGGGTGTTGAGGAGAGAAAGTGAAAGGAAGGCGTGG

8 TTGACACTGGGAATTTAGTCATCCTGGGTGTTGAGGAGAGAAAGTGAAAGGAAGGCGTGG

7 TTGACACTGGGAATTTAGTCATCCTGGGTGTTGAGGAGAGAAAGTGAAAGGAAGGCGTGG

19 TTGACACTGGGAATTTAGTCATCCTGGGTGTTGAGGAGAGAAAGTGAAAGGAAGGCGTGG

18 TTGACACTGGGAATTTAGTCATCCTGGGTGTTGAGGAGAGAAAGTGAAAGGAAGGCGTGG

22 TTGACACTGGGAATTTAGTCATCCTGGGTGTTGAGGAGAGAAAGTGAAAGGAAGGCGTGG

21 TTGACACTGGGAATTTAGTCATCCTGGGTGTTGAGGAGAGAAAGTGAAAGGAAGGCGTGG

9 TTGACACTGGGAATTTAGTCATCCTGGGTGTTGAGGAGAGAAAGTGAAAGGAAGGCGTGG

6 TTGACACTGGGAATTTAGTCATCCTGGGTGTTGAGGAGAGAAAGTGAAAGGAAGGCGTGG

5 TTGACACTGGGAATTTAGTCATCCTGGGTGTTGAGGAGAGAAAGTGAAAGGAAGGCGTGG

4 TTGACACTGGGAATTTAGTCATCCTGGGTGTTGAGGAGAGAAAGTGAAAGGAAGGCGTGG

3 TTGACACTGGGAATTTAGTCATCCTGGGTGTTGAGGAGAGAAAGTGAAAGGAAGGCGTGG

20 TTGACACTGGGAATTTAGTCATCCTGGGTGTTGAGGAGAGAAAGTGAAAGGAAGGCGTGG

************************************************************

1 CCGTAATTAGTGAATAAAATAATCAAAATATCTCGATAAATTACAAATAAAAACAAGAAA

16 CCGTAATTAGTGAATAAAATAATCAAAATATCTCGATAAATTACAAATAAAAACAAGAAA

15 CCGTAATTAGTGAATAAAATAATCAAAATATCTCGATAAATTACAAATAAAAACAAGAAA

2 CCGTAATTAGTGAATAAAATAATCAAAATATCTCGATAAATTACAAATAAAAACAAGAAA

10 CCGTAATTAATGAATAAAATAATCAAAATATCTCGATAAATTACAAATAAAAACAAGAAA

17 CCGTAATTAATGAATAAAATAATCAAAATATCTCGATAAATTACAAATAAAAACAAGAAA

14 CCGTAATTAATGAATAAAATAATCAAAATATCTCGATAAATTACAAATAAAAACAAGAAA

13 CCGTAATTAATGAATAAAATAATCAAAATATCTCGATAAATTACAAATAAAAACAAGAAA

12 CCGTAATTAATGAATAAAATAATCAAAATATCTCGATAAATTACAAATAAAAACAAGAAA

8 CCGTAATTAATGAATAAAATAATCAAAATATCTCGATAAATTACAAATAAAAACAAGAAA

7 CCGTAATTAATGAATAAAATAATCAAAATATCTCGATAAATTACAAATAAAAACAAGAAA

19 CCGTAATTAATGAATAAAATAATCAAAATATCTCGATAAATTACAAATAAAAACAAGAAA

18 CCGTAATTAATGAATAAAATAATCAAAATATCTCGATAAATTACAAATAAAAACAAGAAA

22 CCGTAATTAATGAATAAAATAATCAAAATATCTCGATAAATTACAAATAAAAACAAGAAA

21 CCGTAATTAATGAATAAAATAATCAAAATATCTCGATAAATTACAAATAAAAACAAGAAA

9 CCGTAATTAATGAATAAAATAATCAAAATATCTCGATAAATTACAAATAAAAACAAGAAA

6 CCGTAATTAATGAATAAAATAATCAAAATATCTCGATAAATTACAAATAAAAACAAGAAA

5 CCGTAATTAATGAATAAAATAATCAAAATATCTCGATAAATTACAAATAAAAACAAGAAA

4 CCGTAATTAATGAATAAAATAATCAAAATATCTCGATAAATTACAAATAAAAACAAGAAA

3 CCGTAATTAATGAATAAAATAATCAAAATATCTCGATAAATTACAAATAAAAACAAGAAA

20 CCGTAATTAATGAATAAAATAATCAAAATATCTCGATAAATTACAAATAAAAACAAGAAA

*********.**************************************************

1 TCAAATTGACGTCAACTTGAGTTAAACTTCTTGGTCTATTACATCGACGTAATTAAATTT

16 TCAAATTGACGTCAACTTGAGTTAAACTTCTTGGTCTATTACATCGACGTAATTAAATTT

15 TCAAATTGACGTCAACTTGAGTTAAACTTCTTGGTCTATTACATCGACGTAATTAAATTT

2 TCAAATTGACGTCAACTTGAGTTAAACTTCTTGGTCTATTACATCGACGTAATTAAATTT

10 TCAAATTGACGTCAACTTGAGTTAAACTTCTTGGTCTATTACATCGACGTAATTAAATTT

17 TCAAATTGACGTCAACTTGAGTTAAACTTCTTGGTCTATTACATCGACGTAATTAAATTT

14 TCAAATTGACGTCAACTTGAGTTAAACTTCTTGGTCTATTACATCGACGTAATTAAATTT

13 TCAAATTGACGTCAACTTGAGTTAAACTTCTTGGTCTATTACATCGACGTAATTAAATTT

12 TCAAATTGACGTCAACTTGAGTTAAACTTCTTGGTCTATTACATCGACGTAATTAAATTT

8 TCAAATTGACGTCAACTTGAGTTAAACTTCTTGGTCTATTACATCGACGTAATTAAATTT

7 TCAAATTGACGTCAACTTGAGTTAAACTTCTTGGTCTATTACATCGACGTAATTAAATTT

19 TCAAATTGACGTCAACTTGAGTTAAACTTCTTGGTCTATTACATCGACGTAATTAAATTT

18 TCAAATTGACGTCAACTTGAGTTAAACTTCTTGGTCTATTACATCGACGTAATTAAATTT

22 TCAAATTGACGTCAACTTGAGTTAAACTTCTTGGTCTATTACATCGACGTAATTAAATTT

21 TCAAATTGACGTCAACTTGAGTTAAACTTCTTGGTCTATTACATCGACGTAATTAAATTT

9 TCAAATTGACGTCAACTTGAGTTAAACTTCTTGGTCTATTACATCGACGTAATTAAATTT

6 TCAAATTGACGTCAACTTGAGTTAAACTTCTTGGTCTATTACATCGACGTAATTAAATTT

5 TCAAATTGACGTCAACTTGAGTTAAACTTCTTGGTCTATTACATCGACGTAATTAAATTT

4 TCAAATTGACGTCAACTTGAGTTAAACTTCTTGGTCTATTACATCGACGTAATTAAATTT

3 TCAAATTGACGTCAACTTGAGTTAAACTTCTTGGTCTATTACATCGACGTAATTAAATTT

20 TCAAATTGACGTCAACTTGAGTTAAACTTCTTGGTCTATTACATCGACGTAATTAAATTT

************************************************************

1 AAATTATGACAGTGAGCTTTTTTTATATATTTAACTACAAATCTAAACGGAATTCAAGCT

16 AAATTATGACAGTGAGCTTTTTTTATATATTTAACTACAAATCTAAACGGAATTCAAGCT

15 AAATTATGACAGTGAGCTTTTTTTATATATTTAACTAAAAATCTAAACGGAATTCAAGCT

2 AAATTATGACAGTGAGCTTTTTTTATATATTTAACTAAAAATCTAAACGGAATTCAAGCT

10 AAATTATGACAGTGAGCTTTTTTTATATATTTAACTACAAATCTAAACGGAATTCAAGCT

17 AAATTATGACAGTGAGCTTTTTTTATATATTTAACTACAAATCTAAACGGAATTCAAGCT

14 AAATTATGACAGTGAGCTTTTTTTATATATTTAACTACAAATCTAAACGGAATTCAAGCT

13 AAATTATGACAGTGAGCTTTTTTTATATATTTAACTACAAATCTAAACGGAATTCAAGCT

12 AAATTATGACAGTGAGCTTTTTTTATATATTTAACTACAAATCTAAACGGAATTCAAGCT

8 AAATTATGACAGTGAGCTTTTTTTATATATTTAACTACAAATCTAAACGGAATTCAAGCT

7 AAATTATGACAGTGAGCTTTTTTTATATATTTAACTACAAATCTAAACGGAATTCAAGCT

19 AAATTATGACAGTGAGCTTTTTTTATATATTTAACTACAAATCTAAACGGAATTCAAGCT

18 AAATTATGACAGTGAGCTTTTTTTATATATTTAACTACAAATCTAAACGGAATTCAAGCT

22 AAATTATGACAGTGAGCTTTTTTTATATATTTAACTACAAATCTAAACGGAATTCAAGCT

21 AAATTATGACAGTGAGCTTTTTTTATATATTTAACTACAAATCTAAACGGAATTCAAGCT

9 AAATTATGACAGTGAGCTTTTTTTATATATTTAACTACAAATCTAAACGGAATTCAAGCT

6 AAATTATGACAGTGAGCTTTTTTTATATATTTAACTACAAATCTAAACGGAATTCAAGCT

5 AAATTATGACAGTGAGCTTTTTTTATATATTTAACTACAAATCTAAACGGAATTCAAGCT

4 AAATTATGACAGTGAGCTTTTTTTATATATTTAACTACAAATCTAAACGGAATTCAAGCT

3 AAATTATGACAGTGAGCTTTTTTTATATATTTAACTACAAATCTAAACGGAATTCAAGCT

20 AAATTATGACAGTGAGCTTTTTTTATATATTTAACTACAAATCTAAACGGAATTCAAGCT

************************************* **********************

**BAP31>>>>>>>>>>>>>>>>>>>>>>>>>>>>>>>>>>>>>>>>>>>>>>>>>>**

1 TTATTATAGCTTAGTGTTAGTTGTCTTACAGAGGCACAATCCACTCTAACACCTTTATCA

16 TTATTATAGCTTAGTGTTAGTTGTCTTACAGAGGCACAATCCACTCTAACACCTTTATCA

15 TTATTATAGCTTAGTGTTAGTTGTCTTACAGAGGCACAATCCACTCTAACACCTTTATCA

2 TTATTATAGCTTAGTGTTAGTTGTCTTACAGAGGCACAATCCACTCTAACACCTTTATCA

10 TTATTATAGCTTAGTGTTAGTTGTCTTACAGAGGCACAATCCACTCTAACACCTTTATCA

17 TTATTATAGCTTAGTGTTAGTTGTCTTACAGAGGCACAATCCACTCTAACACCTTTATCA

14 TTATTATAGCTTAGTGTTAGTTGTCTTACAGAGGCACAATCCACTCTAACACCTTTATCA

13 TTATTATAGCTTAGTGTTAGTTGTCTTACAGAGGCACAATCCACTCTAACACCTTTATCA

12 TTATTATAGCTTAGTGTTAGTTGTCTTACAGAGGCACAATCCACTCTAACACCTTTATCA

8 TTATTATAGCTTAGTGTTAGTTGTCTTACAGAGGCACAATCCACTCTAACACCTTTATCA

7 TTATTATAGCTTAGTGTTAGTTGTCTTACAGAGGCACAATCCACTCTAACACCTTTATCA

19 TTATTATAGCTTAGTGTTAGTTGTCTTACAGAGGCACAATCCACTCTAACACCTTTATCA

18 TTATTATAGCTTAGTGTTAGTTGTCTTACAGAGGCACAATCCACTCTAACACCTTTATCA

22 TTATTATAGCTTAGTGTTAGTTGTCTTACAGAGGCACAATCCACTCTAACACCTTTATCA

21 TTATTATAGCTTAGTGTTAGTTGTCTTACAGAGGCACAATCCACTCTAACACCTTTATCA

9 TTATTATAGCTTAGTGTTAGTTGTCTTACAGAGGCACAATCCACTCTAACACCTTTATCA

6 TTATTATAGCTTAGTGTTAGTTGTCTTACAGAGGCACAATCCACTCTAACACCTTTATCA

5 TTATTATAGCTTAGTGTTAGTTGTCTTACAGAGGCACAATCCACTCTAACACCTTTATCA

4 TTATTATAGCTTAGTGTTAGTTGTCTTACAGAGGCACAATCCACTCTAACACCTTTATCA

3 TTATTATAGCTTAGTGTTAGTTGTCTTACAGAGGCACAATCCACTCTAACACCTTTATCA

20 TTATTATAGCTTAGTGTTAGTTGTCTTACAGAGGCACAATCCACTCTAACACCTTTATCA

************************************************************

**>>>>>>>>>>>>>>>>>>>>>>>>>>>>>>>>>>>>>>>>>>>>>>>>>>>>>>>>>>>>**

1 GATTTGGTTTACTCAGCGCAGAGTTAGTCAATCGGCTATTAACATACACACTCTAATACC

16 GATTTGGTTTACTCAGCGCAGAGTTAGTCAATCGGCTATTAACATACACACTCTAATACC

15 GATTTGGTTTACTCAGCGCAGAGTTAGTCAATCGGCTATTAACATACACACTCTAATACC

2 GATTTGGTTTACTCAGCGCAGAGTTAGTCAATCGGCTATTAACATACACACTCTAATACC

10 GATTTGGTTTACTCAGCGCAGAGTTAGTCAATCGGCTATTAACATACACACTCTAATACC

17 GATTTGGTTTACTCAGCGCAGAGTTAGTCAATCGGCTATTAACATACACACTCTAATAAC

14 GATTTGGTTTACTCAGCGCAGAGTTAGTCAATCGGCTATTAACATACACACTCTAATAAC

13 GATTTGGTTTACTCAGCGCAGAGTTAGTCAATCGGCTATTAACATACACACTCTAATAAC

12 GATTTGGTTTACTCAGCGCAGAGTTAGTCAATCGGCTATTAACATACACACTCTAATAAC

8 GATTTGGTTTACTCAGCGCAGAGTTAGTCAATCGGCTATTAACATACACACTCTAATAAC

7 GATTTGGTTTACTCAGCGCAGAGTTAGTCAATCGGCTATTAACATACACACTCTAATAAC

19 GATTTGGTTTACTCAGCGCAGAGTTAGTCAATCGGCTATTAACATACACACTCTAATAAC

18 GATTTGGTTTACTCAGCGCAGAGTTAGTCAATCGGCTATTAACATACACACTCTAATAAC

22 GATTTGGTTTACTCAGCGCAGAGTTAGTCAATCGGCTATTAACATACACACTCTAATACC

21 GATTTGGTTTACTCAGCGCAGAGTTAGTCAATCGGCTATTAACATACACACTCTAATACC

9 GATTTGGTTTACTCAGCGCAGAGTTAGTCAATCGGCTATTAACATACACACTCTAATACC

6 GATTTGGTTTACTCAGCGCAGAGTTAGTCAATCGGCTATTAACATACACACTCTAATACC

5 GATTTGGTTTACTCAGCGCAGAGTTAGTCAATCGGCTATTAACATACACACTCTAATACC

4 GATTTGGTTTACTCAGCGCAGAGTTAGTCAATCGGCTATTAACATACACACTCTAATACC

3 GATTTGGTTTACTCAGCGCAGAGTTAGTCAATCGGCTATTAACATACACACTCTAATACC

20 GATTTGGTTTACTCAGCGCAGAGTTAGTCAATCGGCTATTAACATACACACTCTAATACC

********************************************************** *

**>>>>>>>>>>>>>>>>>>>>>>>>>>>>>>>>>>>>>>>>>>>>>>>>>>>>>>>>>>>>**

1 TCCTCAGAGTACTTACCTTCTTGGTCCTCGTTGCACCAATGCCTTTCGCTTTCAAGCAAC

16 TCCTCAGAGTACTTACCTTCTTGGTCCTCGTTGCACCAATGCCTTTCGCTTTCAAGCAAC

15 TCCTCAGAGTACTTACCTTCTTGGTCCTCGTTGCACCAATGCCTTTCGCTTTCAAGCAAC

2 TCCTCAGAGTACTTACCTTCTTGGTCCTCGTTGCACCAATGCCTTTCGCTTTCAAGCAAC

10 GCCTCAGAGTCCTTACCTTCTTGGTCCTCGTTGCACCAATGCCTTTCGCTTTCAAGCAAC

17 GCCTCAGAGTCCTTACCTTCTTGGTCCTCGTTGCACCAATGCCTTTCGCTTTCAAGCAAC

14 GCCTCAGAGTCCTTACCTTCTTGGTCCTCGTTGCACCAATGCCTTTCGCTTTCAAGCAAC

13 GCCTCAGAGTCCTTACCTTCTTGGTCCTCGTTGCACCAATGCCTTTCGCTTTCAAGCAAC

12 GCCTCAGAGTCCTTACCTTCTTGGTCCTCGTTGCACCAATGCCTTTCGCTTTCAAGCAAC

8 GCCTCAGAGTCCTTACCTTCTTGGTCCTCGTTGCACCAATGCCTTTCGCTTTCAAGCAAC

7 GCCTCAGAGTCCTTACCTTCTTGGTCCTCGTTGCACCAATGCCTTTCGCTTTCAAGCAAC

19 GCCTCAGAGTCCTTACCTTCTTGGTCCTCGTTGCACCAATGCCTTTCGCTTTCAAGCAAC

18 GCCTCAGAGTCCTTACCTTCTTGGTCCTCGTTGCACCAATGCCTTTCGCTTTCAAGCAAC

22 GCCTCAGAGTCCTTACCTTCTTGGTCCTCGTTGCACCAATGCCTTTCGCTTTCAAGCAAC

21 GCCTCAGAGTCCTTACCTTCTTGGTCCTCGTTGCACCAATGCCTTTCGCTTTCAAGCAAC

9 GCCTCAGAGTCCTTACCTTCTTGGTCCTCGTTGCACCAATGCCTTTCGCTTTCAAGCAAC

6 GCCTCAGAGTCCTTACCTTCTTGGTCCTCGTTGCACCAATGCCTTTCGCTTTCAAGCAAC

5 GCCTCAGAGTCCTTACCTTCTTGGTCCTCGTTGCACCAATGCCTTTCGCTTTCAAGCAAC

4 GCCTCAGAGTCCTTACCTTCTTGGTCCTCGTTGCACCAATGCCTTTCGCTTTCAAGCAAC

3 GCCTCAGAGTCCTTACCTTCTTGGTCCTCGTTGCACCAATGCCTTTCGCTTTCAAGCAAC

20 GCCTCAGAGTCCTTACCTTCTTGGTCCTCGTTGCACCAATGCCTTTCGCTTTCAAGCAAC

********* *************************************************

**>>>>>>>>>>>>>>>>>>>>>>>>>>>>>>>>>>>>>>>>>>>>>>>>>>>>>>>>>>>>**

1 GCTTCTTCAAATTCCTCAGCACCAACCCATTGGTTGCTAAATTACAATATTCCTTGAAAA

16 GCTTCTTCAAATTCCTCAGCACCAACCCATTGGTTGCTAAATTACAATATTCCTTGAAAA

15 GCTTCTTCAAATTCCTCAGCACCAACCCATTGGTTGCCAAATTACAATATTCCTTGAAAA

2 GCTTCTTCAAATTCCTCAGCACCAACCCATTGGTTGCCAAATTACAATATTCCTTGAAAA

10 GCTTCTTCAAATTCCTCAGCACCAACCCATTGGTTGCTAAATTACAATATTCCTTGAAAA

17 GCTTCTTCAAATTCCTCAGCACCAACCCATTGGTTGCTAAATTACAATATTCCTTGAAAA

14 GCTTCTTCAAATTCCTCAGCACCAACCCATTGGTTGCTAAATTACAATATTCCTTGAAAA

13 GCTTCTTCAAATTCCTCAGCACCAACCCATTGGTTGCTAAATTACAATATTCCTTGAAAA

12 GCTTCTTCAAATTCCTCAGCACCAACCCATTGGTTGCTAAATTACAATATTCCTTGAAAA

8 GCTTCTTCAAATTCCTCAGCACCAACCCATTGGTTGCTAAATTACAATATTCCTTGAAAA

7 GCTTCTTCAAATTCCTCAGCACCAACCCATTGGTTGCTAAATTACAATATTCCTTGAAAA

19 GCTTCTTCAAATTCCTCAGCACCAACCCATTGGTTGCTAAATTACAATATTCCTTGAAAA

18 GCTTCTTCAAATTCCTCAGCACCAACCCATTGGTTGCTAAATTACAATATTCCTTGAAAA

22 GCTTCTTCAAATTCCTCAGCACCAACCCATTGGTTGCTAAATTACAATATTCCTTGAAAA

21 GCTTCTTCAAATTCCTCAGCACCAACCCATTGGTTGCTAAATTACAATATTCCTTGAAAA

9 GCTTCTTCAAATTCCTCAGCACCAACCCATTGGTTGCTAAATTACAATATTCCTTGAAAA

6 GCTTCTTCAAATTCCTCAGCACCAACCCATTGGTTGCTAAATTACAATATTCCTTGAAAA

5 GCTTCTTCAAATTCCTCAGCACCAACCCATTGGTTGCTAAATTACAATATTCCTTGAAAA

4 GCTTCTTCAAATTCCTCAGCACCAACCCATTGGTTGCTAAATTACAATATTCCTTGAAAA

3 GCTTCTTCAAATTCCTCAGCACCAACCCATTGGTTGCTAAATTACAATATTCCTTGAAAA

20 GCTTCTTCAAATTCCTCAGCACCAACCCATTGGTTGCTAAATTACAATATTCCTTGAAAA

*************************************.**********************

**>>>>>>>>>>>>>>>>>>>>>>>>>>>>>>>>>>>>>>>>>>>>>>>>>>>>>>>>>>>>**

1 TATCTCTTATTTTCACTTCAATCTTATTCTTTGATGCTGTTCAAAGGATGCTCAAAGTCG

16 TATCTCTTATTTTCACTTCAATCTTATTCTTTGATGCTGTTCAAAGGATGCTCAAAGTCG

15 TATCTCTTATTTTCACTTCAATCTTATTCTTTGATGCTGTTCAAAGGATGCTCAAAGTCG

2 TATCTCTTATTTTCACTTCAATCTTATTCTTTGATGCTGTTCAAAGGATGCTCAAAGTCG

10 TATCTCTTATTTTCACTTCAATCTTATTCTTTGATGCTGTTCAAAGGATGCTCAAGGTCG

17 TATCTCTTATTTTCACTTCAATCTTATTCTTTGATGCTGTTCAAAGGATGCTCAAAGTCG

14 TATCTCTTATTTTCACTTCAATCTTATTCTTTGATGCTGTTCAAAGGATGCTCAAAGTCG

13 TATCTCTTATTTTCACTTCAATCTTATTCTTTGATGCTGTTCAAAGGATGCTCAAAGTCG

12 TATCTCTTATTTTCACTTCAATCTTATTCTTTGATGCTGTTCAAAGGATGCTCAAAGTCG

8 TATCTCTTATTTTCACTTCAATCTTATTCTTTGATGCTGTTCAAAGGATGCTCAAAGTCG

7 TATCTCTTATTTTCACTTCAATCTTATTCTTTGATGCTGTTCAAAGGATGCTCAAAGTCG

19 TATCTCTTATTTTCACTTCAATCTTATTCTTTGATGCTGTTCAAAGGATGCTCAAAGTCG

18 TATCTCTTATTTTCACTTCAATCTTATTCTTTGATGCTGTTCAAAGGATGCTCAAAGTCG

22 TATCTCTTATTTTCACTTCAATCTTATTCTTTGATGCTGTTCAAAGGATGCTCAAAGTCG

21 TATCTCTTATTTTCACTTCAATCTTATTCTTTGATGCTGTTCAAAGGATGCTCAAAGTCG

9 TATCTCTTATTTTCACTTCAATCTTATTCTTTGATGCTGTTCAAAGGATGCTCAAAGTCG

6 TATCTCTTATTTTCACTTCAATCTTATTCTTTGATGCTGTTCAAAGGATGCTCAAAGTCG

5 TATCTCTTATTTTCACTTCAATCTTATTCTTTGATGCTGTTCAAAGGATGCTCAAAGTCG

4 TATCTCTTATTTTCACTTCAATCTTATTCTTTGATGCTGTTCAAAGGATGCTCAAAGTCG

3 TATCTCTTATTTTCACTTCAATCTTATTCTTTGATGCTGTTCAAAGGATGCTCAAAGTCG

20 TATCTCTTATTTTCACTTCAATCTTATTCTTTGATGCTGTTCAAAGGATGCTCAAAGTCG

*******************************************************.****

**>>>>>>>>>>>>>>>>>>>>>>>>>>>>>>>>>>>>>>>>>>>>>>>>>>>>>>>>>>>>**

1 TCAAAGAAGGCCAAGCTGCTAAGGAAGAGCACTCTTTCAATGATGTACGCTCTGAATCTA

16 TCAAAGAAGGCCAAGCTGCTAAGGAAGAGCACTCTTTCAATGATGTACGCTCTGAATCTA

15 TCAAAGAAGGCCAAGCTGCTAAGGAAGAGCACTCTTTCAATGATGTACGCTCTGAATCTA

2 TCAAAGAAGGCCAAGCTGCTAAGGAAGAGCACTCTTTCAATGATGTACGCTCTGAATCTA

10 TCAAAGAAGGCCAAGCTGCTAAGGAAGAGCACTCTTTCAATGATGTACGCTCTGAATCTA

17 TCAAAGAAGGCCAAGCTGCTAAGGAAGAGCACTCTTTCAATGATGTACGCTCTGAATCTA

14 TCAAAGAAGGCCAAGCTGCTAAGGAAGAGCACTCTTTCAATGATGTACGCTCTGAATCTA

13 TCAAAGAAGGCCAAGCTGCTAAGGAAGAGCACTCTTTCAATGATGTACGCTCTGAATCTA

12 TCAAAGAAGGCCAAGCTGCTAAGGAAGAGCACTCTTTCAATGATGTACGCTCTGAATCTA

8 TCAAAGAAGGCCAAGCTGCTAAGGAAGAGCACTCTTTCAATGATGTACGCTCTGAATCTA

7 TCAAAGAAGGCCAAGCTGCTAAGGAAGAGCACTCTTTCAATGATGTACGCTCTGAATCTA

19 TCAAAGAAGGCCAAGCTGCTAAGGAAGAGCACTCTTTCAATGATGTACGCTCTGAATCTA

18 TCAAAGAAGGCCAAGCTGCTAAGGAAGAGCACTCTTTCAATGATGTACGCTCTGAATCTA

22 TCAAAGAAGGCCAAGCTGCTAAGGAAGAGCACTCTTTCAATGATGTACGCTCTGAATCTA

21 TCAAAGAAGGCCAAGCTGCTAAGGAAGAGCACTCTTTCAATGATGTACGCTCTGAATCTA

9 TCAAAGAAGGCCAAGCTGCTAAGGAAGAGCACTCTTTCAATGATGTACGCTCTGAATCTA

6 TCAAAGAAGGCCAAGCTGCTAAGGAAGAGCACTCTTTCAATGATGTACGCTCTGAATCTA

5 TCAAAGAAGGCCAAGCTGCTAAGGAAGAGCACTCTTTCAATGATGTACGCTCTGAATCTA

4 TCAAAGAAGGCCAAGCTGCTAAGGAAGAGCACTCTTTCAATGATGTACGCTCTGAATCTA

3 TCAAAGAAGGCCAAGCTGCTAAGGAAGAGCACTCTTTCAATGATGTACGCTCTGAATCTA

20 TCAAAGAAGGCCAAGCTGCTAAGGAAGAGCACTCTTTCAATGATGTACGCTCTGAATCTA

************************************************************

**>>>>>>>>>>>>>>>>>>>>>>>>>>>>>>>>>>>>>>>>>>>>>>>>>>>>>>>>>>>>**

1 ACTTTGCTGCACGCAAGTACGTCATCTTCTTTTT-AAAAAGGAATGTCTCTTATTTTCAA

16 ACTTTGCTGCACGCAAGTACGTCATCTTCTTTTT-AAAAAGGAATGTCTCTTATTTTCAA

15 ACTTTGCTGCACGCAAGTACGTCATCTTCTTTTTTAAAAAGGAATGTCTCTTATTTTCAA

2 ACTTTGCTGCACGCAAGTACGTCATCTTCTTTTTTAAAAAGGAATGTCTCTTATTTTCAA

10 ACTTTGCTGCACGCAAGTACGTCATCTTCTTTTT-AAAAAGGAATGTCTCTTATTTTCAA

17 ACTTTGCTGCACGCAAGTACGTCATCTTCTTTTT-AAAAAGGAATGTCTCTTATTTTCAA

14 ACTTTGCTGCACGCAAGTACGTCATCTTCTTTTT-AAAAAGGAATGTCTCTTATTTTCAA

13 ACTTTGCTGCACGCAAGTACGTCATCTTCTTTTT-AAAAAGGAATGTCTCTTATTTTCAA

12 ACTTTGCTGCACGCAAGTACGTCATCTTCTTTTT-AAAAAGGAATGTCTCTTATTTTCAA

8 ACTTTGCTGCACGCAAGTACGTCATCTTCTTTTT-AAAAAGGAATGTCTCTTATTTTCAA

7 ACTTTGCTGCACGCAAGTACGTCATCTTCTTTTT-AAAAAGGAATGTCTCTTATTTTCAA

19 ACTTTGCTGCACGCAAGTACGTCATCTTCTTTTT-AAAAAGGAATGTCTCTTATTTTCAA

18 ACTTTGCTGCACGCAAGTACGTCATCTTCTTTTT-AAAAAGGAATGTCTCTTATTTTCAA

22 ACTTTGCTGCACGCAAGTACGTCATCTTCTTTTT-AAAAAGGAATGTCTCTTATTTTCAA

21 ACTTTGCTGCACGCAAGTACGTCATCTTCTTTTT--AAAAGGAATGTCTCTTATTTTCAA

9 ACTTTGCTGCACGCAAGTACGTCATCTTCTTTTT--AAAAGGAATGTCTCTTATTTTCAA

6 ACTTTGCTGCACGCAAGTACGTCATCTTCTTTTT--AAAAGGAATGTCTCTTATTTTCAA

5 ACTTTGCTGCACGCAAGTACGTCATCTTCTTTTT--AAAAGGAATGTCTCTTATTTTCAA

4 ACTTTGCTGCACGCAAGTACGTCATCTTCTTTTT--AAAAGGAATGTCTCTTATTTTCAA

3 ACTTTGCTGCACGCAAGTACGTCATCTTCTTTTT--AAAAGGAATGTCTCTTATTTTCAA

20 ACTTTGCTGCACGCAAGTACGTCATCTTCTTTTT--AAAAGGAATGTCTCTTATTTTCAA

********************************** ************************

**>>>>>>>>>>>>>>>>>>>>>>>>>>>>>>>>>>>>>>>>>>>>>>>>>>>>>>>>>>>>**

1 ACCTAGATTTTACTCACAACGTAATGTCTACTTGACTGGTACGTCTTCATCACATTCAAT

16 ACCTAGATTTTACTCACAACGTAATGTCTACTTGACTGGTACGTCTTCATCACATTCAAT

15 ACCTAGATTTTACTCACAACGTAATGTCTACTTGACTGGTACGTCTTCATCACATTCAAT

2 ACCTAGATTTTACTCACAACGTAATGTCTACTTGACTGGTACGTCTTCATCACATTCAAT

10 ACCTAGATTTTACTCACAACGTAATGTCTACTTGACTGGTACGTCTTCATCACATTCAAT

17 ACCTAGATTTTACTCACAACGTAATGTCTACTTGACTGGTACGTCTTCATCACATTCAAT

14 ACCTAGATTTTACTCACAACGTAATGTCTACTTGACTGGTACGTCTTCATCACATTCAAT

13 ACCTAGATTTTACTCACAACGTAATGTCTACTTGACTGGTACGTCTTCATCACATTCAAT

12 ACCTAGATTTTACTCACAACGTAATGTCTACTTGACTGGTACGTCTTCATCACATTCAAT

8 ACCTAGATTTTACTCACAACGTAATGTCTACTTGACTGGTACGTCTTCATCACATTCAAT

7 ACCTAGATTTTACTCACAACGTAATGTCTACTTGACTGGTACGTCTTCATCACATTCAAT

19 ACCTAGATTTTACTCACAACGTAATGTCTACTTGACTGGTACGTCTTCATCACATTCAAT

18 ACCTAGATTTTACTCACAACGTAATGTCTACTTGACTGGTACGTCTTCATCACATTCAAT

22 ACCTAGATTTTACTCACAACGTAATGTCTACTTGACTGGTACGTCTTCATCACATTCAAT

21 ACCTAGATTTTACTCACAACGTAATGTCTACTTGACTGGTACGTCTTCATCACATTCAAT

9 ACCTAGATTTTACTCACAACGTAATGTCTACTTGACTGGTACGTCTTCATCACATTCAAT

6 ACCTAGATTTTACTCACAACGTAATGTCTACTTGACTGGTACGTCTTCATCACATTCAAT

5 ACCTAGATTTTACTCACAACGTAATGTCTACTTGACTGGTACGTCTTCATCACATTCAAT

4 ACCTAGATTTTACTCACAACGTAATGTCTACTTGACTGGTACGTCTTCATCACATTCAAT

3 ACCTAGATTTTACTCACAACGTAATGTCTACTTGACTGGTACGTCTTCATCACATTCAAT

20 ACCTAGATTTTACTCACAACGTAATGTCTACTTGACTGGTACGTCTTCATCACATTCAAT

************************************************************

**>>>>>>>>>>>>>>>>>>>>>>>>>>>>>>>>>>>>>>>>>>>>>>>>>>>>>>>>>>>>**

1 TATCTCATCCTTAAAATCATTCAGGTTTCACTCTCTTCTTAAGCTTGTCAGCTTTTGTTG

16 TATCTCATCCTTAAAATCATTCAGGTTTCACTCTCTTCTTAAGCTTGTCAGCTTTTGTTG

15 TATCTCATCCTTAAAATCATTCAGGTTTCACTCTCTTCTTAAGCTTGTCAGCTTTTGTTG

2 TATCTCATCCTTAAAATCATTCAGGTTTCACTCTCTTCTTAAGCTTGTCAGCTTTTGTTG

10 TATCTCATCCTTAAAATCATTCAGGTTTCACTCTCTTCTTAAGCTTGTCAGCTTTTGTTG

17 TATCTCATCCTTAAAATCATTCAGGTTTCACTCTCTTCTTAAGCTTGTCAGCTTTTGTTG

14 TATCTCATCCTTAAAATCATTCAGGTTTCACTCTCTTCTTAAGCTTGTCAGCTTTTGTTG

13 TATCTCATCCTTAAAATCATTCAGGTTTCACTCTCTTCTTAAGCTTGTCAGCTTTTGTTG

12 TATCTCATCCTTAAAATCATTCAGGTTTCACTCTCTTCTTAAGCTTGTCAGCTTTTGTTG

8 TATCTCATCCTTAAAATCATTCAGGTTTCACTCTCTTCTTAAGCTTGTCAGCTTTTGTTG

7 TATCTCATCCTTAAAATCATTCAGGTTTCACTCTCTTCTTAAGCTTGTCAGCTTTTGTTG

19 TATCTCATCCTTAAAATCATTCAGGTTTCACTCTCTTCTTAAGCTTGTCAGCTTTTGTTG

18 TATCTCATCCTTAAAATCATTCAGGTTTCACTCTCTTCTTAAGCTTGTCAGCTTTTGTTG

22 TATCTCATCCTTAAAATCATTCAGGTTTCACTCTCTTCTTAAGCTTGTCAGCTTTTGTTG

21 TATCTCATCCTTAAAATCATTCAGGTTTCACTCTCTTCTTAAGCTTGTCAGCTTTTGTTG

9 TATCTCATCCTTAAAATCATTCAGGTTTCACTCTCTTCTTAAGCTTGTCAGCTTTTGTTG

6 TATCTCATCCTTAAAATCATTCAGGTTTCACTCTCTTCTTAAGCTTGTCAGCTTTTGTTG

5 TATCTCATCCTTAAAATCATTCAGGTTTCACTCTCTTCTTAAGCTTGTCAGCTTTTGTTG

4 TATCTCATCCTTAAAATCATTCAGGTTTCACTCTCTTCTTAAGCTTGTCAGCTTTTGTTG

3 TATCTCATCCTTAAAATCATTCAGGTTTCACTCTCTTCTTAAGCTTGTCAGCTTTTGTTG

20 TATCTCATCCTTAAAATCATTCAGGTTTCACTCTCTTCTTAAGCTTGTCAGCTTTTGTTG

************************************************************

**>>>>>>>>>>>>>>>>>>>>>>>>>>>>>>>>>>>>>>>>>>>>>>>>>>>>>>>>>>>>**

1 ATTCCCTAAGCAAATACCCCTCACACATCCCACAGGATCCTCTCACGCGTATTCGGCATC

16 ATTCCCTAAGCAAATACCCCTCACACATCCCACAGGATCCTCTCACGCGTATTCGGCATC

15 ATTCCCTAAGCAAATACCCCTCACACATCCCACAGGATCCTCTCACGCGTATTCGGCATC

2 ATTCCCTAAGCAAATACCCCTCACACATCCCACAGGATCCTCTCACGCGTATTCGGCATC

10 ATTCCCTAAGCAAATACCCCTCACACATCCCACAGGATCCTCTCACGCGTATTCGGCATC

17 ATTCCCTAAGCAAATACCCCTCACACATCCCACAGGATCCTCTCACGCGTATTCGGCATC

14 ATTCCCTAAGCAAATACCCCTCACACATCCCACAGGATCCTCTCACGCGTATTCGGCATC

13 ATTCCCTAAGCAAATACCCCTCACACATCCCACAGGATCCTCTCACGCGTATTCGGCATC

12 ATTCCCTAAGCAAATACCCCTCACACATCCCACAGGATCCTCTCACGCGTATTCGGCATC

8 ATTCCCTAAGCAAATACCCCTCACACATCCCACAGGATCCTCTCACGCGTATTCGGCATC

7 ATTCCCTAAGCAAATACCCCTCACACATCCCACAGGATCCTCTCACGCGTATTCGGCATC

19 ATTCCCTAAGCAAATACCCCTCACACATCCCACAGGATCCTCTCACGCGTATTCGGCATC

18 ATTCCCTAAGCAAATACCCCTCACACATCCCACAGGATCCTCTCACGCGTATTCGGCATC

22 ATTCCCTAAGCAAATACCCCTCACACATCCCACAGGATCCTCTCACGCGTATTCGGCATC

21 ATTCCCTAAGCAAATACCCCTCACACATCCCACAGGATCCTCTCACGCGTATTCGGCATC

9 ATTCCCTAAGCAAATACCCCTCACACATCCCACAGGATCCTCTCACGCGTATTCGGCATC

6 ATTCCCTAAGCAAATACCCCTCACACATCCCACAGGATCCTCTCACGCGTATTCGGCATC

5 ATTCCCTAAGCAAATACCCCTCACACATCCCACAGGATCCTCTCACGCGTATTCGGCATC

4 ATTCCCTAAGCAAATACCCCTCACACATCCCACAGGATCCTCTCACGCGTATTCGGCATC

3 ATTCCCTAAGCAAATACCCCTCACACATCCCACAGGATCCTCTCACGCGTATTCGGCATC

20 ATTCCCTAAGCAAATACCCCTCACACATCCCACAGGATCCTCTCACGCGTATTCGGCATC

************************************************************

**>>>>>>>>>>>>>>>>>>>>>>>>>>>>>>>>>>>>>>>>>>>>>>>>>>>>>>>>>>>>**

1 ATTATGGATCTCATCAAAGCTGAGGAAGAATTGAGCTTAATGAAGGTTAGTCACTCCGCT

16 ATTATGGATCTCATCAAAGCTGAGGAAGAATTGAGCTTAATGAAGGTTAGTCACTCCGCT

15 ATTATGGATCTCATCAAAGCTGAGGAAGAATTGAGCTTAATGAAGGTTAGTCACTCCGCT

2 ATTATGGATCTCATCAAAGCTGAGGAAGAATTGAGCTTAATGAAGGTTAGTCACTCCGCT

10 ATTATGGATCTCATCAAAGCTGAGGAAGAATTGAGCTTAATGAAGGTTAGTCACTCCGCT

17 ATTATGGATCTCATCAAAGCTGAGGAAGAATTGAGCTTAATGAAGGTTAGTCACTCCGCT

14 ATTATGGATCTCATCAAAGCTGAGGAAGAATTGAGCTTAATGAAGGTTAGTCACTCCGCT

13 ATTATGGATCTCATCAAAGCTGAGGAAGAATTGAGCTTAATGAAGGTTAGTCACTCCGCT

12 ATTATGGATCTCATCAAAGCTGAGGAAGAATTGAGCTTAATGAAGGTTAGTCACTCCGCT

8 ATTATGGATCTCATCAAAGCTGAGGAAGAATTGAGCTTAATGAAGGTTAGTCACTCCGCT

7 ATTATGGATCTCATCAAAGCTGAGGAAGAATTGAGCTTAATGAAGGTTAGTCACTCCGCT

19 ATTATGGATCTCATCAAAGCTGAGGAAGAATTGAGCTTAATGAAGGTTAGTCACTCCGCT

18 ATTATGGATCTCATCAAAGCTGAGGAAGAATTGAGCTTAATGAAGGTTAGTCACTCCGCT

22 ATTATGGATCTCATCAAAGCTGAGGAAGAATTGAGCTTAATGAAGGTTAGTCACTCCGCT

21 ATTATGGATCTCATCAAAGCTGAGGAAGAATTGAGCTTAATGAAGGTTAGTCACTCCGCT

9 ATTATGGATCTCATCAAAGCTGAGGAAGAATTGAGCTTAATGAAGGTTAGTCACTCCGCT

6 ATTATGGATCTCATCAAAGCTGAGGAAGAATTGAGCTTAATGAAGGTTAGTCACTCCGCT

5 ATTATGGATCTCATCAAAGCTGAGGAAGAATTGAGCTTAATGAAGGTTAGTCACTCCGCT

4 ATTATGGATCTCATCAAAGCTGAGGAAGAATTGAGCTTAATGAAGGTTAGTCACTCCGCT

3 ATTATGGATCTCATCAAAGCTGAGGAAGAATTGAGCTTAATGAAGGTTAGTCACTCCGCT

20 ATTATGGATCTCATCAAAGCTGAGGAAGAATTGAGCTTAATGAAGGTTAGTCACTCCGCT

************************************************************

**>>>>>>>>>>>>>>>>>>>>>>>>>>>>>>>>>>>>>>>>>>>>>>>>>>>>>>>>>>>>**

1 AATAATATCTGATTTTATCACTAATCCACCTACAGAAGGACGGTGACTCCTCATCCGCTG

16 AATAATATCCGATTTTATCACTAATCCACCTACAGAAGGACGGTGACTCCTCATCCGCTG

15 AATAATATCTGATTTTATCACTAATCCACCTACAGAAGGACGGTGACTCCTCATCCGCTG

2 AATAATATCTGATTTTATCACTAATCCACCTACAGAAGGACGGTGACTCCTCATCCGCTG

10 AATAATATCTGATTTTATCACTAATCCACCTACAGAAGGACGGTGACTCCTCATCCGCTG

17 AATAATATCTGATTTTATCACTAATCCACCTACAGAAGGACGGTGACTCCTCATCCGCTG

14 AATAATATCTGATTTTATCACTAATCCACCTACAGAAGGACGGTGACTCCTCATCCGCTG

13 AATAATATCTGATTTTATCACTAATCCACCTACAGAAGGACGGTGACTCCTCATCCGCTG

12 AATAATATCTGATTTTATCACTAATCCACCTACAGAAGGACGGTGACTCCTCATCCGCTG

8 AATAATATCTGATTTTATCACTAATCCACCTACAGAAGGACGGTGACTCCTCATCCGCTG

7 AATAATATCTGATTTTATCACTAATCCACCTACAGAAGGACGGTGACTCCTCATCCGCTG

19 AATAATATCTGATTTTATCACTAATCCACCTACAGAAGGACGGTGACTCCTCATCCGCTG

18 AATAATATCTGATTTTATCACTAATCCACCTACAGAAGGACGGTGACTCCTCATCCGCTG

22 AATAATATCTGATTTTATCACTAATCCACCTACAGAAGGACGGTGACTCCTCATCCGCTG

21 AATAATATCTGATTTTATCACTAATCCACCTACAGAAGGACGGTGACTCCTCATCCGCTG

9 AATAATATCTGATTTTATCACTAATCCACCTACAGAAGGACGGTGACTCCTCATCCGCTG

6 AATAATATCTGATTTTATCACTAATCCACCTACAGAAGGACGGTGACTCCTCATCCGCTG

5 AATAATATCTGATTTTATCACTAATCCACCTACAGAAGGACGGTGACTCCTCATCCGCTG

4 AATAATATCTGATTTTATCACTAATCCACCTACAGAAGGACGGTGACTCCTCATCCGCTG

3 AATAATATCTGATTTTATCACTAATCCACCTACAGAAGGACGGTGACTCCTCATCCGCTG

20 AATAATATCTGATTTTATCACTAATCCACCTACAGAAGGACGGTGACTCCTCATCCGCTG

*********.**************************************************

**>>>>>>>>>>>>>>>>>>>>>>>>>>>>>>>>>>>>>>>>>>>>>>>>>>>>>>>>>>>>**

1 AAACCTTTAAGAAACAACTTGACTCAAAAGATCGTGAAATTAGTAAGCACACCTTGAATT

16 AAACCTTTAAGAAACAACTTGACTCAAAAGATCGTGAAATTAGTAAGCACACCTTGAATT

15 AAACCTTTAAGAAACAACTTGACTCAAAAGATCGTGAAATTAGTAAGCACACCTTGAATT

2 AAACCTTTAAGAAACAACTTGACTCAAAAGATCGTGAAATTAGTAAGCACACCTTGAATT

10 AAACCTTTAAGAAACAACTTGACTCAAAAGATCGTGAAATTAGTAAGCACACCTTGAATT

17 AAACCTTTAAGAAACAACTTGACTCAAAAGATCGTGAAATTAGTAAGCACACCTTGAATT

14 AAACCTTTAAGAAACAACTTGACTCAAAAGATCGTGAAATTAGTAAGCACACCTTGAATT

13 AAACCTTTAAGAAACAACTTGACTCAAAAGATCGTGAAATTAGTAAGCACACCTTGAATT

12 AAACCTTTAAGAAACAACTTGACTCAAAAGATCGTGAAATTAGTAAGCACACCTTGAATT

8 AAACCTTTAAGAAACAACTTGACTCAAAAGATCGTGAAATTAGTAAGCACACCTTGAATT

7 AAACCTTTAAGAAACAACTTGACTCAAAAGATCGTGAAATTAGTAAGCACACCTTGAATT

19 AAACCTTTAAGAAACAACTTGACTCAAAAGATCGTGAAATTAGTAAGCACACCTTGAATT

18 AAACCTTTAAGAAACAACTTGACTCAAAAGATCGTGAAATTAGTAAGCACACCTTGAATT

22 AAACCTTTAAGAAACAACTTGACTCAAAAGATCGTGAAATTAGTAAGCACACCTTGAATT

21 AAACCTTTAAGAAACAACTTGACTCAAAAGATCGTGAAATTAGTAAGCACACCTTGAATT

9 AAACCTTTAAGAAACAACTTGACTCAAAAGATCGTGAAATTAGTAAGCACACCTTGAATT

6 AAACCTTTAAGAAACAACTTGACTCAAAAGATCGTGAAATTAGTAAGCACACCTTGAATT

5 AAACCTTTAAGAAACAACTTGACTCAAAAGATCGTGAAATTAGTAAGCACACCTTGAATT

4 AAACCTTTAAGAAACAACTTGACTCAAAAGATCGTGAAATTAGTAAGCACACCTTGAATT

3 AAACCTTTAAGAAACAACTTGACTCAAAAGATCGTGAAATTAGTAAGCACACCTTGAATT

20 AAACCTTTAAGAAACAACTTGACTCAAAAGATCGTGAAATTAGTAAGCACACCTTGAATT

************************************************************

**>>>>>>>>>>>>>>>>>>>>>>>>>>>>>>>>>>>>>>>>>>>>>>>>>>>>>>>>>>>>**

1 TCTCCATTCACTTTGCTAAC-TTCTCTCTAGACATCCTCAAAAAGCAATCTACTCAAAAT

16 TCTCCATTCACTTTGCTAAC-TTCTCTCTAGACATCCTCAAAAAGCAATCTACTCAAAAT

15 TCTCCATTCACTTTGCTAAC-TTCTCTCTAGACATCCTCAAAAAGCAATCTACTCAAAAT

2 TCTCCATTCACTTTGCTAAC-TTCTCTCTAGACATCCTCAAAAAGCAATCTACTCAAAAT

10 TCTCCATTCACTTTGCTAAC-TTCTCTCTAGACATCCTCAAAAAGCAATCTACTCAAAAT

17 TCTCCATTCACTTTGCTAACTTTCTCTCTAGACATCCTCAAAAAGCAATCTACTCAAAAT

14 TCTCCATTCACTTTGCTAACTTTCTCTCTAGACATCCTCAAAAAGCAATCTACTCAAAAT

13 TCTCCATTCACTTTGCTAACTTTCTCTCTAGACATCCTCAAAAAGCAATCTACTCAAAAT

12 TCTCCATTCACTTTGCTAACTTTCTCTCTAGACATCCTCAAAAAGCAATCTACTCAAAAT

8 TCTCCATTCACTTTGCTAACTTTCTCTCTAGACATCCTCAAAAAGCAATCTACTCAAAAT

7 TCTCCATTCACTTTGCTAACTTTCTCTCTAGACATCCTCAAAAAGCAATCTACTCAAAAT

19 TCTCCATTCACTTTGCTAACTTTCTCTCTAGACATCCTCAAAAAGCAATCTACTCAAAAT

18 TCTCCATTCACTTTGCTAACTTTCTCTCTAGACATCCTCAAAAAGCAATCTACTCAAAAT

22 TCTCCATTCACTTTGCTAAC-TTCTCTCTAGACATCCTCAAAAAGCAATCTACTCAAAAT

21 TCTCCATTCACTTTGCTAAC-TTCTCTCTAGACATCCTCAAAAAGCAATCTACTCAAAAT

9 TCTCCATTCACTTTGCTAAC-TTCTCTCTAGACATCCTCAAAAAGCAATCTACTCAAAAT

6 TCTCCATTCACTTTGCTAAC-TTCTCTCTAGACATCCTCAAAAAGCAATCTACTCAAAAT

5 TCTCCATTCACTTTGCTAAC-TTCTCTCTAGACATCCTCAAAAAGCAATCTACTCAAAAT

4 TCTCCATTCACTTTGCTAAC-TTCTCTCTAGACATCCTCAAAAAGCAATCTACTCAAAAT

3 TCTCCATTCACTTTGCTAAC-TTCTCTCTAGACATCCTCAAAAAGCAATCTACTCAAAAT

20 TCTCCATTCACTTTGCTAAC-TTCTCTCTAGACATCCTCAAAAAGCAATCTACTCAAAAT

******************** ***************************************

**>>>>>>>>>>>>>>>>>>>>>>>>>>>>>>>>>>>>>>>>>>>>>>>>>>>>>>>>>>>>**

1 AATCAAGCTATGAACGATCTCGTCGACAACGCTAATACTTCATCCGGCTCAAAGAAAGAC

16 AATCAAGCTATGAACGATCTCGTCGACAACGCTAATACTTCATCCGGCTCAAAGAAAGAC

15 AATCAAGCTATGAACGATCTCGTCGACAACGCTAATACTTCATCCGGCTCAAAGAAAGAC

2 AATCAAGCTATGAACGATCTCGTCGACAACGCTAATACTTCATCCGGCTCAAAGAAAGAC

10 AATCAAGCTATGAACGATCTTGTCGACAACGCGAATACTTCATCCGGCTCAAAGAAAGAC

17 AATCAAGCTATGAACGATCTTGTCGACAACGCGAATACTTCATCCGGCTCAAAGAAAGAC

14 AATCAAGCTATGAACGATCTTGTCGACAACGCGAATACTTCATCCGGCTCAAAGAAAGAC

13 AATCAAGCTATGAACGATCTTGTCGACAACGCGAATACTTCATCCGGCTCAAAGAAAGAC

12 AATCAAGCTATGAACGATCTTGTCGACAACGCGAATACTTCATCCGGCTCAAAGAAAGAC

8 AATCAAGCTATGAACGATCTTGTCGACAACGCGAATACTTCATCCGGCTCAAAGAAAGAC

7 AATCAAGCTATGAACGATCTTGTCGACAACGCGAATACTTCATCCGGCTCAAAGAAAGAC

19 AATCAAGCTATGAACGATCTTGTCGACAACGCGAATACTTCATCCGGCTCAAAGAAAGAC

18 AATCAAGCTATGAACGATCTTGTCGACAACGCGAATACTTCATCCGGCTCAAAGAAAGAC

22 AATCAAGCTATGAACGATCTTGTCGACAACGCGAATACTTCATCCGGCTCAAAGAAAGAC

21 AATCAAGCTATGAACGATCTTGTCGACAACGCGAATACTTCATCCGGCTCAAAGAAAGAC

9 AATCAAGCTATGAACGATCTTGTCGACAACGCGAATACTTCATCCGGCTCAAAGAAAGAC

6 AATCAAGCTATGAACGATCTTGTCGACAACGCGAATACTTCATCCGGCTCAAAGAAAGAC

5 AATCAAGCTATGAACGATCTTGTCGACAACGCGAATACTTCATCCGGCTCAAAGAAAGAC

4 AATCAAGCTATGAACGATCTTGTCGACAACGCGAATACTTCATCCGGCTCAAAGAAAGAC

3 AATCAAGCTATGAACGATCTTGTCGACAACGCGAATACTTCATCCGGCTCAAAGAAAGAC

20 AATCAAGCTATGAACGATCTTGTCGACAACGCGAATACTTCATCCGGCTCAAAGAAAGAC

********************.*********** ***************************

**>>>>>>>>>>>>>>>>>>>>>>>>>>>>>>>>>>>>>>>>>>>>>>>>>>>>>>>>>>>>**

1 AAATAATGGCCAAAAATGTAGTTGTTGTAATGATATATTGCTACTTACTATATTTTATGT

16 AAATAATGGCCAAAAATGTAGTTGTTGTAATGATATATTGCTACTTACTATTTTTTATGT

15 AAATAATGGCCAAAAATGTAGTTGTTGTAATGATATATTGCTACTTACTATTTTTTATGT

2 AAATAATGGCCAAAAATGTAGTTGTTGTAATGATATATTGCTACTTACTATTTTTTATGT

10 AA----------------------------------------------------------

17 AA----------------------------------------------------------

14 AA----------------------------------------------------------

13 AA----------------------------------------------------------

12 AA----------------------------------------------------------

8 AA----------------------------------------------------------

7 AA----------------------------------------------------------

19 AA----------------------------------------------------------

18 AA----------------------------------------------------------

22 AA----------------------------------------------------------

21 AA----------------------------------------------------------

9 AA----------------------------------------------------------

6 AA----------------------------------------------------------

5 AA----------------------------------------------------------

4 AA----------------------------------------------------------

3 AA----------------------------------------------------------

20 AA----------------------------------------------------------

**

**>>>>>>>>>>>>>>>>>>>>>>>>>>>>>>>>>>>>>>>>>>>>>>>>>>>>>>>>>>>>**

**region only present in 1, 2, 15, 16, no similarity to sequences in GenBank**

1 GTTTCAGGTAAAACAATTGATCATAGGCTACAAAATAAATAAGTACACAATACTGATTTT

16 GTTTCAGGTAAAACAATTGATCATAGGCTACAAAATAAATAAGTACACAATACTGATTTT

15 GTTTCAGGTAAAACAATTGATCATAGGCTACAAAATAAATAAGTACACAATACTGATTTT

2 GTTTCAGGTAAAACAATTGATCATAGGCTACAAAATAAATAAGTACACAATACTGATTTT

10 -------GTAAAACAATTAATTGT------------------------------------

17 -------GTAAAACAATTAATTGT------------------------------------

14 -------GTAAAACAATTAATTGT------------------------------------

13 -------GTAAAACAATTAATTGT------------------------------------

12 -------GTAAAACAATTAATTGT------------------------------------

8 -------GTAAAACAATTAATTGT------------------------------------

7 -------GTAAAACAATTAATTGT------------------------------------

19 -------GTAAAACAATTAATTGT------------------------------------

18 -------GTAAAACAATTAATTGT------------------------------------

22 -------GTAAAACAATTAATTGT------------------------------------

21 -------GTAAAACAATTAATTGT------------------------------------

9 -------GTAAAACAATTAATTGT------------------------------------

6 -------GTAAAACAATTAATTGT------------------------------------

5 -------GTAAAACAATTAATTGT------------------------------------

4 -------GTAAAACAATTAATTGT------------------------------------

3 -------GTAAAACAATTAATTGT------------------------------------

20 -------GTAAAACAATTAATTGT------------------------------------

***********.**..*

**>>>>>>BAP31**

**region only present in 1, 2, 15, 16, no similarity to sequences in GenBank**

1 ATATGCTATTTTTAAGAAGAATACTGGGATCAATGAAGTCTACTTCACGTAAAAATTCAT

16 ATATGCTATTTTTAAGAAGAATACTGGGATCAATGAAGTCTACTTCACGTAAAAATTCAT

15 ATATGCTATTTTTAAGAAGAATACTGGGATCAATGAAGTCTACTTCACGTAAAAATTCAT

2 ATATGCTATTTTTAAGAAGAATACTGGGATCAATGAAGTCTACTTCACGTAAAAATTCAT

10 ------------------------------------------------------------

17 ------------------------------------------------------------

14 ------------------------------------------------------------

13 ------------------------------------------------------------

12 ------------------------------------------------------------

8 ------------------------------------------------------------

7 ------------------------------------------------------------

19 ------------------------------------------------------------

18 ------------------------------------------------------------

22 ------------------------------------------------------------

21 ------------------------------------------------------------

9 ------------------------------------------------------------

6 ------------------------------------------------------------

5 ------------------------------------------------------------

4 ------------------------------------------------------------

3 ------------------------------------------------------------

20 ------------------------------------------------------------

**region only present in 1, 2, 15, 16, no similarity to sequences in GenBank**

1 AGCTATTGTGATTCAACTGTTCCGTAAGTTTTTCTAGGGAATCATCACTAGCTGATTGGG

16 AGCTATTGTGATTCAACTGTTCCGTAAGTTTTTCTAGGGAATCATCACTAGCTGATTGGG

15 AGCTATTGTGATTCAACTGTTCCGTAAGTTTTTCTAGGGAATCATCACTAGCTGATGGGG

2 AGCTATTGTGATTCAACTGTTCCGTAAGTTTTTCTAGGGAATCATCACTAGCTGATGGGG

10 ------------------------------------------------------------

17 ------------------------------------------------------------

14 ------------------------------------------------------------

13 ------------------------------------------------------------

12 ------------------------------------------------------------

8 ------------------------------------------------------------

7 ------------------------------------------------------------

19 ------------------------------------------------------------

18 ------------------------------------------------------------

22 ------------------------------------------------------------

21 ------------------------------------------------------------

9 ------------------------------------------------------------

6 ------------------------------------------------------------

5 ------------------------------------------------------------

4 ------------------------------------------------------------

3 ------------------------------------------------------------

20 ------------------------------------------------------------

**region only present in 1, 2, 15, 16, no similarity to sequences in GenBank**

1 GTATAGAAGTTATATTTTGATAAGTCAAATGATTTTGTAGGTGGTTTTGGTATAGAAGCA

16 GTATAGAAGTTATATTTTGATAAGTCAAATGATTTTGTAGGTGGTTTTGGTATAGAAGCA

15 GTATAGAAGTTATATTTTGATAAGTCAAATGATTTTGTAGGTGGTTTTGGTATAGAAGCA

2 GTATAGAAGTTATATTTTGATAAGTCAAATGATTTTGTAGGTGGTTTTGGTATAGAAGCA

10 ------------------------------------------------------------

17 ------------------------------------------------------------

14 ------------------------------------------------------------

13 ------------------------------------------------------------

12 ------------------------------------------------------------

8 ------------------------------------------------------------

7 ------------------------------------------------------------

19 ------------------------------------------------------------

18 ------------------------------------------------------------

22 ------------------------------------------------------------

21 ------------------------------------------------------------

9 ------------------------------------------------------------

6 ------------------------------------------------------------

5 ------------------------------------------------------------

4 ------------------------------------------------------------

3 ------------------------------------------------------------

20 ------------------------------------------------------------

**region only present in 1, 2, 15, 16, no similarity to sequences in GenBank**

1 TATGCCTCATTTGCTCTTGAAACTTGTCAATTGATGTTTCCAACACAAATTTTGTATTAC

16 TATGCCTCATTTGCTCTTGAAACTTGTCAATTGATGTTTCCAACACAAATTTTGTATTAC

15 TATGCCTCATTTGCTCTTGAAACTTGTCAATTGATGTTTCCAACACTAATTTTGTATTAC

2 TATGCCTCATTTGCTCTTGAAACTTGTCAATTGATGTTTCCAACACTAATTTTGTATTAC

10 ------------------------------------------------------------

17 ------------------------------------------------------------

14 ------------------------------------------------------------

13 ------------------------------------------------------------

12 ------------------------------------------------------------

8 ------------------------------------------------------------

7 ------------------------------------------------------------

19 ------------------------------------------------------------

18 ------------------------------------------------------------

22 ------------------------------------------------------------

21 ------------------------------------------------------------

9 ------------------------------------------------------------

6 ------------------------------------------------------------

5 ------------------------------------------------------------

4 ------------------------------------------------------------

3 ------------------------------------------------------------

20 ------------------------------------------------------------

**region only present in 1, 2, 15, 16, no similarity to sequences in GenBank**

1 CTTTCAGTCTTTTGTTCTCACTTTCGAGTCTTTCGATACGCTCTTTCTGATTTTGGCGGA

16 CTTTCAGTCTTTTGTTCTCACTTTCGAGTCTTTCGATACGCTCTTTCTGATTTTGGCGGA

15 CTTTCAGTCTTTTGTTCTCACTTTCGAGTCTTTCGATACGCTCTTTCTGATTTTGGCGGA

2 CTTTCAGTCTTTTGTTCTCACTTTCGAGTCTTTCGATACGCTCTTTCTGATTTTGGCGGA

10 ------------------------------------------------------------

17 ------------------------------------------------------------

14 ------------------------------------------------------------

13 ------------------------------------------------------------

12 ------------------------------------------------------------

8 ------------------------------------------------------------

7 ------------------------------------------------------------

19 ------------------------------------------------------------

18 ------------------------------------------------------------

22 ------------------------------------------------------------

21 ------------------------------------------------------------

9 ------------------------------------------------------------

6 ------------------------------------------------------------

5 ------------------------------------------------------------

4 ------------------------------------------------------------

3 ------------------------------------------------------------

20 ------------------------------------------------------------

**region only present in 1, 2, 15, 16, no similarity to sequences in GenBank**

1 AACGTCGACCATTTTCTGAAATATGTGAGAAATACTTTCAGTGAGTGTTACCGCCTAACC

16 AACGTCGACCATTTTCTGAAATATGTGAGAAATACTTTCAGTGAGTGTTACCGCCTAACC

15 AACGTCGACCATTTTCTGAAATATGTGAGAAATACTTTCAGTGAGTGTTACCGCCTAACC

2 AACGTCGACCATTTTCTGAAATATGTGAGAAATACTTTCAGTGAGTGTTACCGCCTAACC

10 ------------------------------------------------------------

17 ------------------------------------------------------------

14 ------------------------------------------------------------

13 ------------------------------------------------------------

12 ------------------------------------------------------------

8 ------------------------------------------------------------

7 ------------------------------------------------------------

19 ------------------------------------------------------------

18 ------------------------------------------------------------

22 ------------------------------------------------------------

21 ------------------------------------------------------------

9 ------------------------------------------------------------

6 ------------------------------------------------------------

5 ------------------------------------------------------------

4 ------------------------------------------------------------

3 ------------------------------------------------------------

20 ------------------------------------------------------------

**region only present in 1, 2, 15, 16, no similarity to sequences in GenBank**

1 TTGGCTCCTAACATCACGTGAGCATGGTAATCATATCGTATAAGAACCTACCGAGTGGCG

16 TTGGCTCCTAACATCACGTGAGCATGGTAATCATATCGTATAAGAACCTACCGAGTGGCG

15 TTGGCTCCTAACATCACGTGAGCATGGTAATCATATCGTATAAGAACCTACCGAGTGGCG

2 TTGGCTCCTAACATCACGTGAGCATGGTAATCATATCGTATAAGAACCTACCGAGTGGCG

10 ------------------------------------------------------------

17 ------------------------------------------------------------

14 ------------------------------------------------------------

13 ------------------------------------------------------------

12 ------------------------------------------------------------

8 ------------------------------------------------------------

7 ------------------------------------------------------------

19 ------------------------------------------------------------

18 ------------------------------------------------------------

22 ------------------------------------------------------------

21 ------------------------------------------------------------

9 ------------------------------------------------------------

6 ------------------------------------------------------------

5 ------------------------------------------------------------

4 ------------------------------------------------------------

3 ------------------------------------------------------------

20 ------------------------------------------------------------

**region only present in 1, 2, 15, 16, no similarity to sequences in GenBank**

1 GGATTTAGATCATTGCGAGGACGACCGCGACCTCTCAGCAAGATTGTCTTCTCACTCATA

16 GGATTTAGATCATTGCGAGGACGACCGCGACCTCTTAGCAAGATTGTCTTCTCACTCATA

15 GGATTTAGATCATTGCGAGGACGACCGCGACCTCTTAGCAAGATTGTCTTCTCACTCATA

2 GGATTTAGATCATTGCGAGGACGACCGCGACCTCTTAGCAAGATTGTCTTCTCACTCATA

10 ------------------------------------------------------------

17 ------------------------------------------------------------

14 ------------------------------------------------------------

13 ------------------------------------------------------------

12 ------------------------------------------------------------

8 ------------------------------------------------------------

7 ------------------------------------------------------------

19 ------------------------------------------------------------

18 ------------------------------------------------------------

22 ------------------------------------------------------------

21 ------------------------------------------------------------

9 ------------------------------------------------------------

6 ------------------------------------------------------------

5 ------------------------------------------------------------

4 ------------------------------------------------------------

3 ------------------------------------------------------------

20 ------------------------------------------------------------

**region only present in 1, 2, 15, 16, no similarity to sequences in GenBank**

1 CTTGATGATTGAGGTAGTAGCTAAAGCGGAGTCGAAAATCGTATGACTAATAAGACGTGC

16 CTTGATGATTGAGGTAGTAGCTAAAGCGGAGTCGAAAATCGTATGACTAATAAGACGTGC

15 CTTGATGATTGAGGTAGTAGCTAAAGCGGAGTCGAAAATCGTATGACTAATAAGACGTGC

2 CTTGATGATTGAGGTAGTAGCTAAAGCGGAGTCGAAAATCGTATGACTAATAAGACGTGC

10 --------------------------------------------------TAGGAC----

17 --------------------------------------------------TAGGAC----

14 --------------------------------------------------TAGGAC----

13 --------------------------------------------------TAGGAC----

12 --------------------------------------------------TAGGAC----

8 --------------------------------------------------TAGGAC----

7 --------------------------------------------------TAGGAC----

19 --------------------------------------------------TAGGAC----

18 --------------------------------------------------TAGGAC----

22 --------------------------------------------------TAGGAC----

21 --------------------------------------------------TAGGAC----

9 --------------------------------------------------TAGGAC----

6 --------------------------------------------------TAGGAC----

5 --------------------------------------------------TAGGAC----

4 --------------------------------------------------TAGGAC----

3 --------------------------------------------------TAGGAC----

20 --------------------------------------------------TAGGAC----

**.***

**region only present in 1, 2, 15, 16, no similarity to sequences in GenBank**

1 ACGTGCATAATGCAACTGTCCGCTTGTTTAGGTACCTTTATTGGTGAGGACAATGAGATT

16 ACGTGCATAATGCAACTGTCCGCTTGTTTAGGTACCTTTATTGGTGAGGACAATGAGATT

15 ACGTGCATAATGCAACTGTCCGCTTGTTTAGGTACCTTTATTGGTGAGGACAATGAGATT

2 ACGTGCATAATGCAACTGTCCGCTTGTTTAGGTACCTTTATTGGTGAGGACAATGAGATT

10 ------------------------------------------------------------

17 ------------------------------------------------------------

14 ------------------------------------------------------------

13 ------------------------------------------------------------

12 ------------------------------------------------------------

8 ------------------------------------------------------------

7 ------------------------------------------------------------

19 ------------------------------------------------------------

18 ------------------------------------------------------------

22 ------------------------------------------------------------

21 ------------------------------------------------------------

9 ------------------------------------------------------------

6 ------------------------------------------------------------

5 ------------------------------------------------------------

4 ------------------------------------------------------------

3 ------------------------------------------------------------

20 ------------------------------------------------------------

**region only present in 1, 2, 15, 16, no similarity to sequences in GenBank**

1 TACAAATATATAAATGTAATGTTATGCGAAAAACAATGAACATGCGTGATAAGGTTCAAC

16 TACAAATATATAAATGTAATGTTATGCGAAAAACAATGAACATGCGTGATAAGGTTCAAC

15 TACAAATATATAAATGTAATGTTATGCGAAAAACAATGAACATGCGTGATAAGGTTCAAC

2 TACAAATATATAAATGTAATGTTATGCGAAAAACAATGAACATGCGTGATAAGGTTCAAC

10 ------------------------------------------------------------

17 ------------------------------------------------------------

14 ------------------------------------------------------------

13 ------------------------------------------------------------

12 ------------------------------------------------------------

8 ------------------------------------------------------------

7 ------------------------------------------------------------

19 ------------------------------------------------------------

18 ------------------------------------------------------------

22 ------------------------------------------------------------

21 ------------------------------------------------------------

9 ------------------------------------------------------------

6 ------------------------------------------------------------

5 ------------------------------------------------------------

4 ------------------------------------------------------------

3 ------------------------------------------------------------

20 ------------------------------------------------------------

**region only present in 1, 2, 15, 16, no similarity to sequences in GenBank**

1 GTTTCATTGTTTGATATAGATAAGATTACTTCAAAATTTCAGGATAATACCGCATAAAAG

16 GTTTCATTGTTTGATATAGATAAGATTACTTCAAAATTTCAGGATAATACCGCATAAAAG

15 GTTTCATTGTTTGATATAGATAAGATTACTTCAAAATTTCAGGATAATACCGCATAAAAG

2 GTTTCATTGTTTGATATAGATAAGATTACTTCAAAATTTCAGGATAATACCGCATAAAAG

10 ------------------------------------------------------------

17 ------------------------------------------------------------

14 ------------------------------------------------------------

13 ------------------------------------------------------------

12 ------------------------------------------------------------

8 ------------------------------------------------------------

7 ------------------------------------------------------------

19 ------------------------------------------------------------

18 ------------------------------------------------------------

22 ------------------------------------------------------------

21 ------------------------------------------------------------

9 ------------------------------------------------------------

6 ------------------------------------------------------------

5 ------------------------------------------------------------

4 ------------------------------------------------------------

3 ------------------------------------------------------------

20 ------------------------------------------------------------

**region only present in 1, 2, 15, 16, no similarity to sequences in GenBank**

1 CGCCGAGCTTCACTTTTCACTCATCAAGATAGAAAGAAACACTTAGCAATGGAACCAGAA

16 CGCCGAGCTTCACTTTTCACTCATCAAGATAGAAAGAAACACTTAGCAATGGAACCAGAA

15 CGCCGAGCTTCACTTTTCACTCATCAAGATAGAAAGAAACACTTAGCAATGGAACCAGAA

2 CGCCGAGCTTCACTTTTCACTCATCAAGATAGAAAGAAACACTTAGCAATGGAACCAGAA

10 ------------------------------------------------------------

17 ------------------------------------------------------------

14 ------------------------------------------------------------

13 ------------------------------------------------------------

12 ------------------------------------------------------------

8 ------------------------------------------------------------

7 ------------------------------------------------------------

19 ------------------------------------------------------------

18 ------------------------------------------------------------

22 ------------------------------------------------------------

21 ------------------------------------------------------------

9 ------------------------------------------------------------

6 ------------------------------------------------------------

5 ------------------------------------------------------------

4 ------------------------------------------------------------

3 ------------------------------------------------------------

20 ------------------------------------------------------------

**possible pheromone**

**region only present in 1, 2, 15, 16, no similarity to sequences in GenBank**

1 ACACTGAAGAACATTTTTTTCCAAGAAGCCAGCCAAGACCACTTGAAAGACGAAGCAATC

16 ACACTGAAGAACATTTTTTTCCAAGAAGCCAGCCAAGACCACTTGAAAGACGAAGCAATC

15 ACACTGAAGAACATTTTTTTCCAAGAAGCCAGCCAAGACCACTTGAAAGACGAAGCAATC

2 ACACTGAAGAACATTTTTTTCCAAGAAGCCAGCCAAGACCACTTGAAAGACGAAGCAATC

10 ------------------------------------------------------------

17 ------------------------------------------------------------

14 ------------------------------------------------------------

13 ------------------------------------------------------------

12 ------------------------------------------------------------

8 ------------------------------------------------------------

7 ------------------------------------------------------------

19 ------------------------------------------------------------

18 ------------------------------------------------------------

22 ------------------------------------------------------------

21 ------------------------------------------------------------

9 ------------------------------------------------------------

6 ------------------------------------------------------------

5 ------------------------------------------------------------

4 ------------------------------------------------------------

3 ------------------------------------------------------------

20 ------------------------------------------------------------

**precursor MEPETLKNIFFQEASQDHLKDEAILFETSIGMPEISSEDLANSNNPINDS**

**region only present in 1, 2, 15, 16, no similarity to sequences in GenBank**

1 CTTTTTGAAACAAGCATTGGAATGCCTGAAATATCAAGTGAGGACTTGGCCAACTCAAAC

16 CTTTTTGAAACAAGCATTGGAATGCCTGAAATATCAAGTGAGGACTTGGCCAACTCAAAC

15 CTTTTTGAAACAAGCATTGGAATGCCTGAAATATCAAGTGAGGACTTGGCCAACTCAAAC

2 CTTTTTGAAACAAGCATTGGAATGCCTGAAATATCAAGTGAGGACTTGGCCAACTCAAAC

10 ------------------------------------------------------------

17 ------------------------------------------------------------

14 ------------------------------------------------------------

13 ------------------------------------------------------------

12 ------------------------------------------------------------

8 ------------------------------------------------------------

7 ------------------------------------------------------------

19 ------------------------------------------------------------

18 ------------------------------------------------------------

22 ------------------------------------------------------------

21 ------------------------------------------------------------

9 ------------------------------------------------------------

6 ------------------------------------------------------------

5 ------------------------------------------------------------

4 ------------------------------------------------------------

3 ------------------------------------------------------------

20 ------------------------------------------------------------

**TGGDNADTMYCIIV >>>>>>>>>>>>>>>>>>>>>>>>>>>>>>>>>>>>>>>>>>>>>**

**region only present in 1, 2, 15, 16, no similarity to sequences in GenBank**

1 AATCCTATCAACGACAGTACAGGTGGAGATAATGCTGATACCATGTACTGTATTATTGTC

16 AATCCTATCAACGACAGTACAGGTGGAGATAATGCTGATACCATGTACTGTATTATTGTC

15 AATCCTATCAACGACAGTACAGGTGGAGATAATGCTGATACCATGTACTGTATTATTGTC

2 AATCCTATCAACGACAGTACAGGTGGAGATAATGCTGATACCATGTACTGTATTATTGTC

10 ------------------------------------------------------------

17 ------------------------------------------------------------

14 ------------------------------------------------------------

13 ------------------------------------------------------------

12 ------------------------------------------------------------

8 ------------------------------------------------------------

7 ------------------------------------------------------------

19 ------------------------------------------------------------

18 ------------------------------------------------------------

22 ------------------------------------------------------------

21 ------------------------------------------------------------

9 ------------------------------------------------------------

6 ------------------------------------------------------------

5 ------------------------------------------------------------

4 ------------------------------------------------------------

3 ------------------------------------------------------------

20 ------------------------------------------------------------

**region only present in 1, 2, 15, 16, no similarity to sequences in GenBank**

1 TAAGTAGGTAATAGTAGTTCTGCACACAATCTTCATACTAATTTGGAGTAGAAAATGATT

16 TAAGTAGGTAATAGTAGTTCTGCACACAATCTTCATACTAATTTGGAGTAGAAAATGATT

15 TAAGTAGGTAATAGTAGTTCTGCACACAATCTTCATACTAATTTGGAGTAGAAAATGATT

2 TAAGTAGGTAATAGTAGTTCTGCACACAATCTTCATACTAATTTGGAGTAGAAAATGATT

10 ------------------------------------------------------------

17 ------------------------------------------------------------

14 ------------------------------------------------------------

13 ------------------------------------------------------------

12 ------------------------------------------------------------

8 ------------------------------------------------------------

7 ------------------------------------------------------------

19 ------------------------------------------------------------

18 ------------------------------------------------------------

22 ------------------------------------------------------------

21 ------------------------------------------------------------

9 ------------------------------------------------------------

6 ------------------------------------------------------------

5 ------------------------------------------------------------

4 ------------------------------------------------------------

3 ------------------------------------------------------------

20 ------------------------------------------------------------

**Ph?**

**region only present in 1, 2, 15, 16, no similarity to sequences in GenBank**

1 GTGGGAAATAATTAGACAGTGAATACATGTATACATTATGCAAATAATTCAGGCGCTTGA

16 GTGGGAAATAATTAGACAGTGAATACATGTATACATTATGCAAATAATTCAGGCGCTTGA

15 GTGGGAAATAATTAGACAGTGAATACATGTATACATTATGCAAATAATTCAGGCGCTTGA

2 GTGGGAAATAATTAGACAGTGAATACATGTATACATTATGCAAATAATTCAGGCGCTTGA

10 -----------------------------------TTATGTAAATAATTCAGGCGCTTGG

17 -----------------------------------TTATGTAAATAATTCAGGCGCTTGG

14 -----------------------------------TTATGTAAATAATTCAGGCGCTTGG

13 -----------------------------------TTATGTAAATAATTCAGGCGCTTGG

12 -----------------------------------TTATGTAAATAATTCAGGCGCTTGG

8 -----------------------------------TTATGTAAATAATTCAGGCGCTTGG

7 -----------------------------------TTATGTAAATAATTCAGGCGCTTGG

19 -----------------------------------TTATGTAAATAATTCAGGCGCTTGG

18 -----------------------------------TTATGTAAATAATTCAGGCGCTTGG

22 -----------------------------------TTATGTAAATAATTCAGGCGCTTGG

21 -----------------------------------TTATGTAAATAATTCAGGCGCTTGG

9 -----------------------------------TTATGTAAATAATTCAGGCGCTTGG

6 -----------------------------------TTATGTAAATAATTCAGGCGCTTGG

5 -----------------------------------TTATGTAAATAATTCAGGCGCTTGG

4 -----------------------------------TTATGTAAATAATTCAGGCGCTTGG

3 -----------------------------------TTATGTAAATAATTCAGGCGCTTGG

20 -----------------------------------TTATGTAAATAATTCAGGCGCTTGG

*****.******************.

**region only present in 1, 2, 15, 16, no**

**similarity to sequences in GenBank**

1 CTATTTACATCAACTGCTACATTCTCTACCTTAAACCTCCATTCATTCAACCGCGAGGCT

16 CTATTTACATCAACTGCTACATTCTCTACCTTAAACCTCCATTCATTCAACCGCGAGGCT

15 CTATTTACATCAACTGCTACATTCTCTACCTTAAACCTCCATTCATTCAACCGCGAGGCT

2 CTATTTACATCAACTGCTACATTCTCTACCTTAAACCTCCATTCATTCAACCGCGAGGCT

10 CTATTTACATTGACTGCCACATTTTCCACCTTAGATCTCCATTCATTCAACCGCGAGGCC

17 CTATTTACATTGACTGCCACATTTTCCACCTTAGATCTCCATTCATTCAACCGCGAGGCC

14 CTATTTACATTGACTGCCACATTTTCCACCTTAGATCTCCATTCATTCAACCGCGAGGCC

13 CTATTTACATTGACTGCCACATTTTCCACCTTAGATCTCCATTCATTCAACCGCGAGGCC

12 CTATTTACATTGACTGCCACATTTTCCACCTTAGATCTCCATTCATTCAACCGCGAGGCC

8 CTATTTACATTGACTGCCACATTTTCCACCTTAGATCTCCATTCATTCAACCGCGAGGCC

7 CTATTTACATTGACTGCCACATTTTCCACCTTAGATCTCCATTCATTCAACCGCGAGGCC

19 CTATTTACATTGACTGCCACATTTTCCACCTTAGATCTCCATTCATTCAACCGCGAGGCC

18 CTATTTACATTGACTGCCACATTTTCCACCTTAGATCTCCATTCATTCAACCGCGAGGCC

22 CTATTTACATTGACTGCCACATTTTCCACCTTAGATCTCCATTCATTCAACCGCGAGGCC

21 CTATTTACATTGACTGCCACATTTTCCACCTTAGATCTCCATTCATTCAACCGCGAGGCC

9 CTATTTACATTGACTGCCACATTTTCCACCTTAGATCTCCATTCATTCAACCGCGAGGCC

6 CTATTTACATTGACTGCCACATTTTCCACCTTAGATCTCCATTCATTCAACCGCGAGGCC

5 CTATTTACATTGACTGCCACATTTTCCACCTTAGATCTCCATTCATTCAACCGCGAGGCC

4 CTATTTACATTGACTGCCACATTTTCCACCTTAGATCTCCATTCATTCAACCGCGAGGCC

3 CTATTTACATTGACTGCCACATTTTCCACCTTAGATCTCCATTCATTCAACCGCGAGGCC

20 CTATTTACATTGACTGCCACATTTTCCACCTTAGATCTCCATTCATTCAACCGCGAGGCC

**********..*****.*****.**.******.*.***********************.

1 AAAGATTTGATTTGATTCTTGTCCAAAACCCTTGGTTGTACCCAATTGATGCTAGCTGTA

16 AAAGATTTGATTTGATTCTTGTCCAAAACCCTTGGTTGTACCCAATTGATGCTAGCTGTA

15 AAAGATTTGATTTGATTCTTGTCCAAAACCCTTGGTTGTACCCAATTGATGCTAGCTGTA

2 AAAGATTTGATTTGATTCTTGTCCAAAACCCTTGGTTGTACCCAATTGATGCTAGCTGTA

10 AAAGATTTGATTTGATTCTTGTCCAAAACCCTAGGTTGTACCCAATTGATGCTAGCTGTA

17 AAAGATTTGATTTGATTCTTGTCCAAAACCCTAGGTTGTACCCAATTGATGCTAGCTGTA

14 AAAGATTTGATTTGATTCTTGTCCAAAACCCTAGGTTGTACCCAATTGATGCTAGCTGTA

13 AAAGATTTGATTTGATTCTTGTCCAAAACCCTAGGTTGTACCCAATTGATGCTAGCTGTA

12 AAAGATTTGATTTGATTCTTGTCCAAAACCCTAGGTTGTACCCAATTGATGCTAGCTGTA

8 AAAGATTTGATTTGATTCTTGTCCAAAACCCTAGGTTGTACCCAATTGATGCTAGCTGTA

7 AAAGATTTGATTTGATTCTTGTCCAAAACCCTAGGTTGTACCCAATTGATGCTAGCTGTA

19 AAAGATTTGATTTGATTCTTGTCCAAAACCCTAGGTTGTACCCAATTGATGCTAGCTGTA

18 AAAGATTTGATTTGATTCTTGTCCAAAACCCTAGGTTGTACCCAATTGATGCTAGCTGTA

22 AAAGATTTGATTTGATTCTTGTCCAAAACCCTAGGTTGTACCCAATTGATGCTAGCTGTA

21 AAAGATTTGATTTGATTCTTGTCCAAAACCCTAGGTTGTACCCAATTGATGCTAGCTGTA

9 AAAGATTTGATTTGATTCTTGTCCAAAACCCTAGGTTGTACCCAATTGATGCTAGCTGTA

6 AAAGATTTGATTTGATTCTTGTCCAAAACCCTAGGTTGTACCCAATTGATGCTAGCTGTA

5 AAAGATTTGATTTGATTCTTGTCCAAAACCCTAGGTTGTACCCAATTGATGCTAGCTGTA

4 AAAGATTTGATTTGATTCTTGTCCAAAACCCTAGGTTGTACCCAATTGATGCTAGCTGTA

3 AAAGATTTGATTTGATTCTTGTCCAAAACCCTAGGTTGTACCCAATTGATGCTAGCTGTA

20 AAAGATTTGATTTGATTCTTGTCCAAAACCCTAGGTTGTACCCAATTGATGCTAGCTGTA

******************************** ***************************

1 CCTGAGACTTGATCGATGGAGCCACGAATGAGTTTCAAACTAAATTGATATGTGAGTAAT

16 CCTGAGACTTGATCGATGGAGCCACGAATGAGTTTCAAACTAAATTTATATGTGAGTAAT

15 CCTGAGACTTGATCGATGGAGCCACGAATGAGTTTCAAACTAAATTGATATGTGAGTAAT

2 CCTGAGACTTGATCGATGGAGCCACGAATGAGTTTCAAACTAAATTGATATGTGAGTAAT

10 CTTGAGACTTGATCGATGGTGCCACGAATAAGATTCAAACTTAAGTGATCTGTGAGTGGT

17 CTTGAGACTTGATCGATGGTGCCACGAATAAGATTCAAACTTAAGTGATCTGTGAGTGGT

14 CTTGAGACTTGATCGATGGTGCCACGAATAAGATTCAAACTTAAGTGATCTGTGAGTGGT

13 CTTGAGACTTGATCGATGGTGCCACGAATAAGATTCAAACTTAAGTGATCTGTGAGTGGT

12 CTTGAGACTTGATCGATGGTGCCACGAATAAGATTCAAACTTAAGTGATCTGTGAGTGGT

8 CTTGAGACTTGATCGATGGTGCCACGAATAAGATTCAAACTTAAGTGATCTGTGAGTGGT

7 CTTGAGACTTGATCGATGGTGCCACGAATAAGATTCAAACTTAAGTGATCTGTGAGTGGT

19 CTTGAGACTTGATCGATGGTGCCACGAATAAGATTCAAACTTAAGTGATCTGTGAGTGGT

18 CTTGAGACTTGATCGATGGTGCCACGAATAAGATTCAAACTTAAGTGATCTGTGAGTGGT

22 CTTGAGACTTGATCGATGGTGCCACGAATAAGATTCAAACTTAAGTGATCTGTGAGTGGT

21 CTTGAGACTTGATCGATGGTGCCACGAATAAGATTCAAACTTAAGTGATCTGTGAGTGGT

9 CTTGAGACTTGATCGATGGTGCCACGAATAAGATTCAAACTTAAGTGATCTGTGAGTGGT

6 CTTGAGACTTGATCGATGGTGCCACGAATAAGATTCAAACTTAAGTGATCTGTGAGTGGT

5 CTTGAGACTTGATCGATGGTGCCACGAATAAGATTCAAACTTAAGTGATCTGTGAGTGGT

4 CTTGAGACTTGATCGATGGTGCCACGAATAAGATTCAAACTTAAGTGATCTGTGAGTGGT

3 CTTGAGACTTGATCGATGGTGCCACGAATAAGATTCAAACTTAAGTGATCTGTGAGTGGT

20 CTTGAGACTTGATCGATGGTGCCACGAATAAGATTCAAACTTAAGTGATCTGTGAGTGGT

*.***************** *********.** ******** ** * ** *******..*

1 TATCATCTTCATGCTT---GAAACTCACGACAATGCTTTCATAATCAAATGTTCAACCTC

16 TATCATCTCCATGCTT---GAAACTCACGACAATGCTTTCATAATCAAATGTTCAACCTC

15 TATCATCTTCATGCTT---GAAACTCACGACAATGCTTTCATAATCAAATGTTCAACCTC

2 TATCATCTTCATGCTT---GAAACTCACGACAATGCTTTCATAATCAAATGTTCAACCTC

10 TGATAT-TTCATACTTTTGAAAACTCACGACAATGCTTTCATAATCAAATGTTCAACCTC

17 TGATAT-TTCATACTTTTGAAAACTCACGACAATGCTTTCATAATCAAATGTTCAACCTC

14 TGATAT-TTCATACTTTTGAAAACTCACGACAATGCTTTCATAATCAAATGTTCAACCTC

13 TGATAT-TTCATACTTTTGAAAACTCACGACAATGCTTTCATAATCAAATGTTCAACCTC

12 TGATAT-TTCATACTTTTGAAAACTCACGACAATGCTTTCATAATCAAATGTTCAACCTC

8 TGATAT-TTCATACTTTTGAAAACTCACGACAATGCTTTCATAATCAAATGTTCAACCTC

7 TGATAT-TTCATACTTTTGAAAACTCACGACAATGCTTTCATAATCAAATGTTCAACCTC

19 TGATAT-TTCATACTTTTGAAAACTCACGACAATGCTTTCATAATCAAATGTTCAACCTC

18 TGATAT-TTCATACTTTTGAAAACTCACGACAATGCTTTCATAATCAAATGTTCAACCTC

22 TGATAT-TTCATACTTTTGAAAACTCACGACAATGCTTTCATAATCAAATGTTCAACCTC

21 TGATAT-TTCATACTTTTGAAAACTCACGACAATGCTTTCATAATCAAATGTTCAACCTC

9 TGATAT-TTCATACTTTTGAAAACTCACGACAATGCTTTCATAATCAAATGTTCAACCTC

6 TGATAT-TTCATACTTTTGAAAACTCACGACAATGCTTTCATAATCAAATGTTCAACCTC

5 TGATAT-TTCATACTTTTGAAAACTCACGACAATGCTTTCATAATCAAATGTTCAACCTC

4 TGATAT-TTCATACTTTTGAAAACTCACGACAATGCTTTCATAATCAAATGTTCAACCTC

3 TGATAT-TTCATACTTTTGAAAACTCACGACAATGCTTTCATAATCAAATGTTCAACCTC

20 TGATAT-TTCATACTTTTGAAAACTCACGACAATGCTTTCATAATCAAATGTTCAACCTC

*. .** *.***.*** .****************************************

1 ATCGGATGGTAGCTTTGTCTCAGCACCAATGGTAGAGAATGACATAACGCGGTCCTTAGA

16 ATCGGATGGTAGCTTTGTCTCAGCACCAATGGTAGAGAATGACATAACGCGGTCCTTAGA

15 ATCGGATGGTAGCTTTGTCTCAGCACCAATGGTAGAGAATGACATAACGCGGTCCTTAGA

2 ATCGGATGGTAGCTTTGTCTCAGCACCAATGGTAGAGAATGACATAACGCGGTCCTTAGA

10 ATCTGACGGTAACTTTGTCTCAGCACCAATGGTAGAGAATGACAAAACGCGGTCTTTTGA

17 ATCTGACGGTAACTTTGTCTCAGCACCAATGGTAGAGAATGACAAAACGCGGTCTTTTGA

14 ATCTGACGGTAACTTTGTCTCAGCACCAATGGTAGAGAATGACAAAACGCGGTCTTTTGA

13 ATCTGACGGTAACTTTGTCTCAGCACCAATGGTAGAGAATGACAAAACGCGGTCTTTTGA

12 ATCTGACGGTAACTTTGTCTCAGCACCAATGGTAGAGAATGACAAAACGCGGTCTTTTGA

8 ATCTGACGGTAACTTTGTCTCAGCACCAATGGTAGAGAATGACAAAACGCGGTCTTTTGA

7 ATCTGACGGTAACTTTGTCTCAGCACCAATGGTAGAGAATGACAAAACGCGGTCTTTTGA

19 ATCTGACGGTAACTTTGTCTCAGCACCAATGGTAGAGAATGACAAAACGCGGTCTTTTGA

18 ATCTGACGGTAACTTTGTCTCAGCACCAATGGTAGAGAATGACAAAACGCGGTCTTTTGA

22 ATCTGACGGTAACTTTGTCTCAGCACCAATGGTAGAGAATGACAAAACGCGGTCTTTTGA

21 ATCTGACGGTAACTTTGTCTCAGCACCAATGGTAGAGAATGACAAAACGCGGTCTTTTGA

9 ATCTGACGGTAACTTTGTCTCAGCACCAATGGTAGAGAATGACAAAACGCGGTCTTTTGA

6 ATCTGACGGTAACTTTGTCTCAGCACCAATGGTAGAGAATGACAAAACGCGGTCTTTTGA

5 ATCTGACGGTAACTTTGTCTCAGCACCAATGGTAGAGAATGACAAAACGCGGTCTTTTGA

4 ATCTGACGGTAACTTTGTCTCAGCACCAATGGTAGAGAATGACAAAACGCGGTCTTTTGA

3 ATCTGACGGTAACTTTGTCTCAGCACCAATGGTAGAGAATGACAAAACGCGGTCTTTTGA

20 ATCTGACGGTAACTTTGTCTCAGCACCAATGGTAGAGAATGACAAAACGCGGTCTTTTGA

*** **.****.******************************** *********.** **

1 TGATCTCTTGAAGATACATTCGATTAATGCCATTAAGCATATCTTCTGACGAAGGAAAGC

16 TGATCTCTTGAAGATACATTCGATTAATGCCATTAAGCATATCTTCTGACGAAGGAAAGC

15 TGATCTCTTGAAGATACATTCGATTAATGCCATTAAGCATATCTTCTGACGAAGGAAAGC

2 TGATCTCTTGAAGATACATTCGATTAATGCCATTAAGCATATCTTCTGACGAAGGAAAGC

10 TGATCTCTTGAAGATACATTCGATTAATGCCATTAAGCATATCTTCTGACGAAGGAAAGC

17 TGATCTCTTGAAGATACATTCGATTAATGCCATTAAGCATATCTTCTGACGAAGGAAAGC

14 TGATCTCTTGAAGATACATTCGATTAATGCCATTAAGCATATCTTCTGACGAAGGAAAGC

13 TGATCTCTTGAAGATACATTCGATTAATGCCATTAAGCATATCTTCTGACGAAGGAAAGC

12 TGATCTCTTGAAGATACATTCGATTAATGCCATTAAGCATATCTTCTGACGAAGGAAAGC

8 TGATCTCTTGAAGATACATTCGATTAATGCCATTAAGCATATCTTCTGACGAAGGAAAGC

7 TGATCTCTTGAAGATACATTCGATTAATGCCATTAAGCATATCTTCTGACGAAGGAAAGC

19 TGATCTCTTGAAGATACATTCGATTAATGCCATTAAGCATATCTTCTGACGAAGGAAAGC

18 TGATCTCTTGAAGATACATTCGATTAATGCCATTAAGCATATCTTCTGACGAAGGAAAGC

22 TGATCTCTTGAAGATACATTCGATTAATGCCATTAAGCATATCTTCTGACGAAGGAAAGC

21 TGATCTCTTGAAGATACATTCGATTAATGCCATTAAGCATATCTTCTGACGAAGGAAAGC

9 TGATCTCTTGAAGATACATTCGATTAATGCCATTAAGCATATCTTCTGACGAAGGAAAGC

6 TGATCTCTTGAAGATACATTCGATTAATGCCATTAAGCATATCTTCTGACGAAGGAAAGC

5 TGATCTCTTGAAGATACATTCGATTAATGCCATTAAGCATATCTTCTGACGAAGGAAAGC

4 TGATCTCTTGAAGATACATTCGATTAATGCCATTAAGCATATCTTCTGACGAAGGAAAGC

3 TGATCTCTTGAAGATACATTCGATTAATGCCATTAAGCATATCTTCTGACGAAGGAAAGC

20 TGATCTCTTGAAGATACATTCGATTAATGCCATTAAGCATATCTTCTGACGAAGGAAAGC

************************************************************

1 TTGACTTTCTTGAAGAATTGACTGTACAAAATGTTTACTCATTGTAGCATGACTAGATGG

16 TTGACTTTCTTGAAGAATTGACTGTACAAAATGTTTACTCATTGTAGCATGACTAGATGG

15 TTGACTTTCTTGAAGAATTGACTGTACAAAATGTTTACTCATTGTAGCATGACTAGATGG

2 TTGACTTTCTTGAAGAATTGACTGTACAAAATGTTTACTCATTGTAGCATGACTAGATGG

10 CTGACTTTCTTGAAGAATAGACTGTACAAAATGTTTATTCATTGTAGCATGACTAGACAG

17 CTGACTTTCTTGAAGAATAGACTGTACAAAATGTTTATTCATTGTAGCATGACTAGACGG

14 CTGACTTTCTTGAAGAATAGACTGTACAAAATGTTTATTCATTGTAGCATGACTAGACGG

13 CTGACTTTCTTGAAGAATAGACTGTACAAAATGTTTATTCATTGTAGCATGACTAGACGG

12 CTGACTTTCTTGAAGAATAGACTGTACAAAATGTTTATTCATTGTAGCATGACTAGACGG

8 CTGACTTTCTTGAAGAATAGACTGTACAAAATGTTTATTCATTGTAGCATGACTAGACGG

7 CTGACTTTCTTGAAGAATAGACTGTACAAAATGTTTATTCATTGTAGCATGACTAGACGG

19 CTGACTTTCTTGAAGAATAGACTGTACAAAATGTTTATTCATTGTAGCATGACTAGACGG

18 CTGACTTTCTTGAAGAATAGACTGTACAAAATGTTTATTCATTGTAGCATGACTAGACGG

22 CTGACTTTCTTGAAGAATAGACTGTACAAAATGTTTATTCATTGTAGCATGACTAGACGG

21 CTGACTTTCTTGAAGAATAGACTGTACAAAATGTTTATTCATTGTAGCATGACTAGACGG

9 CTGACTTTCTTGAAGAATAGACTGTACAAAATGTTTATTCATTGTAGCATGACTAGACGG

6 CTGACTTTCTTGAAGAATAGACTGTACAAAATGTTTATTCATTGTAGCATGACTAGACGG

5 CTGACTTTCTTGAAGAATAGACTGTACAAAATGTTTATTCATTGTAGCATGACTAGACGG

4 CTGACTTTCTTGAAGAATAGACTGTACAAAATGTTTATTCATTGTAGCATGACTAGACGG

3 CTGACTTTCTTGAAGAATAGACTGTACAAAATGTTTATTCATTGTAGCATGACTAGACGG

20 CTGACTTTCTTGAAGAATAGACTGTACAAAATGTTTATTCATTGTAGCATGACTAGACGG

.***************** ******************.*******************..*

1 GAAACTTACCTCATTTGCTAACTTGTTTGCGTTGAGCTCGAACTTCGCTATGTCGCCTTC

16 GAAACTTACCTCATTTGCTAACTTGTTTGCGTTGAGCTCGAACTTCGCTATGTCGCCTTC

15 GAAACTTACCTCATTTGCTAACTTGTTTGCGTTGAGCTCGAACTTCGCTATGTCGCCTTC

2 GAAACTTACCTCATTTGCTAACTTGTTTGCGTTGAGCTCGAACTTCGCTATGTCGCCTTC

10 GAAACTTACCTCATTTGCTAACTTGTTTGCGTTGAGCTCGAACTTTGCTATGTCACCTTC

17 GAAACTTACCTCATTTGCTAACTTGTTTGCGTTGAGCTCGAACTTTGCTATGTCACCTTC

14 GAAACTTACCTCATTTGCTAACTTGTTTGCGTTGAGCTCGAACTTTGCTATGTCACCTTC

13 GAAACTTACCTCATTTGCTAACTTGTTTGCGTTGAGCTCGAACTTTGCTATGTCACCTTC

12 GAAACTTACCTCATTTGCTAACTTGTTTGCGTTGAGCTCGAACTTTGCTATGTCACCTTC

8 GAAACTTACCTCATTTGCTAACTTGTTTGCGTTGAGCTCGAACTTTGCTATGTCACCTTC

7 GAAACTTACCTCATTTGCTAACTTGTTTGCGTTGAGCTCGAACTTTGCTATGTCACCTTC

19 GAAACTTACCTCATTTGCTAACTTGTTTGCGTTGAGCTCGAACTTTGCTATGTCACCTTC

18 GAAACTTACCTCATTTGCTAACTTGTTTGCGTTGAGCTCGAACTTTGCTATGTCACCTTC

22 GAAACTTACCTCATTTGCTAACTTGTTTGCGTTGAGCTCGAACTTTGCTATGTCACCTTC

21 GAAACTTACCTCATTTGCTAACTTGTTTGCGTTGAGCTCGAACTTTGCTATGTCACCTTC

9 GAAACTTACCTCATTTGCTAACTTGTTTGCGTTGAGCTCGAACTTTGCTATGTCACCTTC

6 GAAACTTACCTCATTTGCTAACTTGTTTGCGTTGAGCTCGAACTTTGCTATGTCACCTTC

5 GAAACTTACCTCATTTGCTAACTTGTTTGCGTTGAGCTCGAACTTTGCTATGTCACCTTC

4 GAAACTTACCTCATTTGCTAACTTGTTTGCGTTGAGCTCGAACTTTGCTATGTCACCTTC

3 GAAACTTACCTCATTTGCTAACTTGTTTGCGTTGAGCTCGAACTTTGCTATGTCACCTTC

20 GAAACTTACCTCATTTGCTAACTTGTTTGCGTTGAGCTCGAACTTTGCTATGTCACCTTC

*********************************************.********.*****

1 ATTAAAAATGTAGATAATGTCCTTTAGCCATTCTAGATCACCACCATTCAAAGCATTCAA

16 ATTAAAAATGTAGATAATGTCCTTTAGCCATTCTAGATCACCACCATTCAAAGCATTCAA

15 ATTAAAAATGTAGATAATGTCCTTTAGCCATTCTAGATCACCACCATTCAAAGCATTCAA

2 ATTAAAAATGTAGATAATGTCCTTTAGCCATTCTAGATCACCACCATTCAAAGCATTCAA

10 ATTAAAAATGTAGATAATGTCCTTCAACCATTCTAGATCACTACCATTCAAAGCATTCAA

17 ATTAAAAATGTAGATAATGTCCTTCAACCATTCTAGATCACTACCATTCAAAGCATTCAA

14 ATTAAAAATGTAGATAATGTCCTTCAACCATTCTAGATCACTACCATTCAAAGCATTCAA

13 ATTAAAAATGTAGATAATGTCCTTCAACCATTCTAGATCACTACCATTCAAAGCATTCAA

12 ATTAAAAATGTAGATAATGTCCTTCAACCATTCTAGATCACTACCATTCAAAGCATTCAA

8 ATTAAAAATGTAGATAATGTCCTTCAACCATTCTAGATCACTACCATTCAAAGCATTCAA

7 ATTAAAAATGTAGATAATGTCCTTCAACCATTCTAGATCACTACCATTCAAAGCATTCAA

19 ATTAAAAATGTAGATAATGTCCTTCAACCATTCTAGATCACTACCATTCAAAGCATTCAA

18 ATTAAAAATGTAGATAATGTCCTTCAACCATTCTAGATCACTACCATTCAAAGCATTCAA

22 ATTAAAAATGTAGATAATGTCCTTCAACCATTCTAGATCACTACCATTCAAAGCATTCAA

21 ATTAAAAATGTAGATAATGTCCTTCAACCATTCTAGATCACTACCATTCAAAGCATTCAA

9 ATTAAAAATGTAGATAATGTCCTTCAACCATTCTAGATCACTACCATTCAAAGCATTCAA

6 ATTAAAAATGTAGATAATGTCCTTCAACCATTCTAGATCACTACCATTCAAAGCATTCAA

5 ATTAAAAATGTAGATAATGTCCTTCAACCATTCTAGATCACTACCATTCAAAGCATTCAA

4 ATTAAAAATGTAGATAATGTCCTTCAACCATTCTAGATCACTACCATTCAAAGCATTCAA

3 ATTAAAAATGTAGATAATGTCCTTCAACCATTCTAGATCACTACCATTCAAAGCATTCAA

20 ATTAAAAATGTAGATAATGTCCTTCAACCATTCTAGATCACTACCATTCAAAGCATTCAA

************************.*.**************.******************

1 GACCGGATGTGTTAACTGATGTGCTCGTAAGCCTCTTTCTGAATAGGAAATTGCGATGAC

16 GACCGGATGTGTTAACTGATGTGCTCGTAAGCCTCTTTCTGAATAGGAAATTGCGATGAC

15 GACCGGATGTGTTAACTGATGTGCTCGTAAGCCTCTTTCTGAATAGGAAATTGCGATGAC

2 GACCGGATGTGTTAACTGATGTGCTCGTAAGCCTCTTTCTGAATAGGAAATTGCGATGAC

10 GACAGGATGAGTTAACTGGT-TACTCGTAAGCTCTTTCTGAAATGAAAAATTGCGATGAC

17 GACAGGATGAGTTAACTGGT-TACTCGTAAGCTCTTTCTGAAATGAAAAATTGCGATGAC

14 GACAGGATGAGTTAACTGGT-TACTCGTAAGCTCTTTCTGAAATGAAAAATTGCGATGAC

13 GACAGGATGAGTTAACTGGT-TACTCGTAAGCTCTTTCTGAAATGAAAAATTGCGATGAC

12 GACAGGATGAGTTAACTGGT-TACTCGTAAGCTCTTTCTGAAATGAAAAATTGCGATGAC

8 GACAGGATGAGTTAACTGGT-TACTCGTAAGCTCTTTCTGAAATGAAAAATTGCGATGAC

7 GACAGGATGAGTTAACTGGT-TACTCGTAAGCTCTTTCTGAAATGAAAAATTGCGATGAC

19 GACAGGATGAGTTAACTGGT-TACTCGTAAGCTCTTTCTGAAATGAAAAATTGCGATGAC

18 GACAGGATGAGTTAACTGGT-TACTCGTAAGCTCTTTCTGAAATGAAAAATTGCGATGAC

22 GACAGGATGAGTTAACTGGT-TACTCGTAAGCTCTTTCTGAAATGAAAAATTGCGATGAC

21 GACAGGATGAGTTAACTGGT-TACTCGTAAGCTCTTTCTGAAATGAAAAATTGCGATGAC

9 GACAGGATGAGTTAACTGGT-TACTCGTAAGCTCTTTCTGAAATGAAAAATTGCGATGAC

6 GACAGGATGAGTTAACTGGT-TACTCGTAAGCTCTTTCTGAAATGAAAAATTGCGATGAC

5 GACAGGATGAGTTAACTGGT-TACTCGTAAGCTCTTTCTGAAATGAAAAATTGCGATGAC

4 GACAGGATGAGTTAACTGGT-TACTCGTAAGCTCTTTCTGAAATGAAAAATTGCGATGAC

3 GACAGGATGAGTTAACTGGT-TACTCGTAAGCTCTTTCTGAAATGAAAAATTGCGATGAC

20 GACAGGATGAGTTAACTGGT-TACTCGTAAGCTCTTTCTGAAATGAAAAATTGCGATGAC

*** ***** ********.* *.*********...**.. .***...*************

1 TAACCAATTCACCAAAGTTATAGATGGTATCACCTAATAAAGCAGCGATAGCCAAATCAT

16 TAACCAATTCACCAAAGTTATAGATGGTATCACCTAATAAAGCAGCGATAGCCAAATCAT

15 TAACCAATTCACCAAAGTTATAGATGGTATCACCTAATAAAGCAGCGATAGCCAAATCAT

2 TAACCAATTCACCAAAGTTATAGATGGTATCACCTAATAAAGCAGCGATAGCCAAATCAT

10 TAACCAATTCACCAAAGTTATAGATAGTATCGCCTAATAAAGCAGCGATAGCCAAATCAT

17 TAACCAATTCACCAAAGTTATAGATAGTATCGCCTAATAAAGCAGCGATAGCCAAATCAT

14 TAACCAATTCACCAAAGTTATAGATAGTATCGCCTAATAAAGCAGCGATAGCCAAATCAT

13 TAACCAATTCACCAAAGTTATAGATAGTATCGCCTAATAAAGCAGCGATAGCCAAATCAT

12 TAACCAATTCACCAAAGTTATAGATAGTATCGCCTAATAAAGCAGCGATAGCCAAATCAT

8 TAACCAATTCACCAAAGTTATAGATAGTATCGCCTAATAAAGCAGCGATAGCCAAATCAT

7 TAACCAATTCACCAAAGTTATAGATAGTATCGCCTAATAAAGCAGCGATAGCCAAATCAT

19 TAACCAATTCACCAAAGTTATAGATAGTATCGCCTAATAAAGCAGCGATAGCCAAATCAT

18 TAACCAATTCACCAAAGTTATAGATAGTATCGCCTAATAAAGCAGCGATAGCCAAATCAT

22 TAACCAATTCACCAAAGTTATAGATAGTATCGCCTAATAAAGCAGCGATAGCCAAATCAT

21 TAACCAATTCACCAAAGTTATAGATAGTATCGCCTAATAAAGCAGCGATAGCCAAATCAT

9 TAACCAATTCACCAAAGTTATAGATAGTATCGCCTAATAAAGCAGCGATAGCCAAATCAT

6 TAACCAATTCACCAAAGTTATAGATAGTATCGCCTAATAAAGCAGCGATAGCCAAATCAT

5 TAACCAATTCACCAAAGTTATAGATAGTATCGCCTAATAAAGCAGCGATAGCCAAATCAT

4 TAACCAATTCACCAAAGTTATAGATAGTATCGCCTAATAAAGCAGCGATAGCCAAATCAT

3 TAACCAATTCACCAAAGTTATAGATAGTATCGCCTAATAAAGCAGCGATAGCCAAATCAT

20 TAACCAATTCACCAAAGTTATAGATAGTATCGCCTAATAAAGCAGCGATAGCCAAATCAT

*************************.*****.****************************

1 GACTTTTTTGAATCTGTTCAACCTTGTCAAGTTGATCTAAGCCAGCACACGAGATATACA

16 GACTTTTTTGAATCTGTTCAACCTTGTCAAGTTGATCTAAGCCAGCACACGAGATATACA

15 GACTTTTTTGAATCTGTTCAACCTTGTCAAGTTGATCTAAGCCAGCACACGAGATATACA

2 GACTTTTTTGAATCTGTTCAACCTTGTCAAGTTGATCTAAGCCAGCACACGAGATATACA

10 GACTTTTTTGAATCTGTTCAACTTTGTCAAGTTGATCTAAGCCAGCACATGAGATGTACA

17 GACTTTTTTGAATCTGTTCAACTTTGTCAAGTTGATCTAAGCCAGCACATGAGATGTACA

14 GACTTTTTTGAATCTGTTCAACTTTGTCAAGTTGATCTAAGCCAGCACATGAGATGTACA

13 GACTTTTTTGAATCTGTTCAACTTTGTCAAGTTGATCTAAGCCAGCACATGAGATGTACA

12 GACTTTTTTGAATCTGTTCAACTTTGTCAAGTTGATCTAAGCCAGCACATGAGATGTACA

8 GACTTTTTTGAATCTGTTCAACTTTGTCAAGTTGATCTAAGCCAGCACATGAGATGTACA

7 GACTTTTTTGAATCTGTTCAACTTTGTCAAGTTGATCTAAGCCAGCACATGAGATGTACA

19 GACTTTTTTGAATCTGTTCAACTTTGTCAAGTTGATCTAAGCCAGCACATGAGATGTACA

18 GACTTTTTTGAATCTGTTCAACTTTGTCAAGTTGATCTAAGCCAGCACATGAGATGTACA

22 GACTTTTTTGAATCTGTTCAACTTTGTCAAGTTGATCTAAGCCAGCACATGAGATGTACA

21 GACTTTTTTGAATCTGTTCAACTTTGTCAAGTTGATCTAAGCCAGCACATGAGATGTACA

9 GACTTTTTTGAATCTGTTCAACTTTGTCAAGTTGATCTAAGCCAGCACATGAGATGTACA

6 GACTTTTTTGAATCTGTTCAACTTTGTCAAGTTGATCTAAGCCAGCACATGAGATGTACA

5 GACTTTTTTGAATCTGTTCAACTTTGTCAAGTTGATCTAAGCCAGCACATGAGATGTACA

4 GACTTTTTTGAATCTGTTCAACTTTGTCAAGTTGATCTAAGCCAGCACATGAGATGTACA

3 GACTTTTTTGAATCTGTTCAACTTTGTCAAGTTGATCTAAGCCAGCACATGAGATGTACA

20 GACTTTTTTGAATCTGTTCAACTTTGTCAAGTTGATCTAAGCCAGCACATGAGATGTACA

**********************.**************************.*****.****

1 ATAGGGTTGTACGGTAGAATGCTGCATATTCGGCCTTAGACTGTGAGACATTCAGT---C

16 ATAGGGTTGTACGGTAGAATGCTGCATATTCGGCCTTAGACTGTGAGACATTCAGT---C

15 ATAGGGTTGTACGGTAGAATGCTGCATATTCGGCCTTAGACTGTGAGACATTCAGT---C

2 ATAGGGTTGTACGGTAGAATGCTGCATATTCGGCCTTAGACTGTGAGACATTCAGT---C

10 ACAAGGTTGTACGGTAGAATGCTGCATATTCGGCCTTAGACTGTGAGTCATCTAGTCAAC

17 ACAAGGTTGTACGGTAGAATGCTGCATATTCGGCCTTAGACTGTGAGTCATCTAGTCAAC

14 ACAAGGTTGTACGGTAGAATGCTGCATATTCGGCCTTAGACTGTGAGTCATCTAGTCAAC

13 ACAAGGTTGTACGGTAGAATGCTGCATATTCGGCCTTAGACTGTGAGTCATCTAGTCAAC

12 ACAAGGTTGTACGGTAGAATGCTGCATATTCGGCCTTAGACTGTGAGTCATCTAGTCAAC

8 ACAAGGTTGTACGGTAGAATGCTGCATATTCGGCCTTAGACTGTGAGTCATCTAGTCAAC

7 ACAAGGTTGTACGGTAGAATGCTGCATATTCGGCCTTAGACTGTGAGTCATCTAGTCAAC

19 ACAAGGTTGTACGGTAGAATGCTGCATATTCGGCCTTAGACTGTGAGTCATCTAGTCAAC

18 ACAAGGTTGTACGGTAGAATGCTGCATATTCGGCCTTAGACTGTGAGTCATCTAGTCAAC

22 ACAAGGTTGTACGGTAGAATGCTGCATATTCGGCCTTAGACTGTGAGTCATCTAGTCAAC

21 ACAAGGTTGTACGGTAGAATGCTGCATATTCGGCCTTAGACTGTGAGTCATCTAGTCAAC

9 ACAAGGTTGTACGGTAGAATGCTGCATATTCGGCCTTAGACTGTGAGTCATCTAGTCAAC

6 ACAAGGTTGTACGGTAGAATGCTGCATATTCGGCCTTAGACTGTGAGTCATCTAGTCAAC

5 ACAAGGTTGTACGGTAGAATGCTGCATATTCGGCCTTAGACTGTGAGTCATCTAGTCAAC

4 ACAAGGTTGTACGGTAGAATGCTGCATATTCGGCCTTAGACTGTGAGTCATCTAGTCAAC

3 ACAAGGTTGTACGGTAGAATGCTGCATATTCGGCCTTAGACTGTGAGTCATCTAGTCAAC

20 ACAAGGTTGTACGGTAGAATGCTGCATATTCGGCCTTAGACTGTGAGTCATCTAGTCAAC

*.*.******************************************* ***..*** *

1 AAAGTGTGAAAAGAAAAGATTGAAGCTATAATTACCTTAAAATAATCACCGCTAACACGC

16 AAAGTGTGAAAAGAAAAGATTGAAGCTATAATTACCTTAAAATAATCACCGCTAACACGC

15 AAAGTGTGAAAAGAAAAGATTGAAGCTATAATTACCTTAAAATAATCACCGCTAACACGC

2 AAAGTGTGAAAAGAAAAGATTGAAGCTATAATTACCTTAAAATAATCACCGCTAACACGC

10 AAAGTGTAAAAATAAAAAATTAAAGCTATAATTACCTTGTAAAAATCACCGCTAACACGC

17 AAAGTGTAAAAATAAAAAATTAAAGCTATAATTACCTTGTAAAAATCACCGCTAACACGC

14 AAAGTGTAAAAATAAAAAATTAAAGCTATAATTACCTTGTAAAAATCACCGCTAACACGC

13 AAAGTGTAAAAATAAAAAATTAAAGCTATAATTACCTTGTAAAAATCACCGCTAACACGC

12 AAAGTGTAAAAATAAAAAATTAAAGCTATAATTACCTTGTAAAAATCACCGCTAACACGC

8 AAAGTGTAAAAATAAAAAATTAAAGCTATAATTACCTTGTAAAAATCACCGCTAACACGC

7 AAAGTGTAAAAATAAAAAATTAAAGCTATAATTACCTTGTAAAAATCACCGCTAACACGC

19 AAAGTGTAAAAATAAAAAATTAAAGCTATAATTACCTTGTAAAAATCACCGCTAACACGC

18 AAAGTGTAAAAATAAAAAATTAAAGCTATAATTACCTTGTAAAAATCACCGCTAACACGC

22 AAAGTGTAAAAATAAAAAATTAAAGCTATAATTACCTTGTAAAAATCACCGCTAACACGC

21 AAAGTGTAAAAATAAAAAATTAAAGCTATAATTACCTTGTAAAAATCACCGCTAACACGC

9 AAAGTGTAAAAATAAAAAATTAAAGCTATAATTACCTTGTAAAAATCACCGCTAACACGC

6 AAAGTGTAAAAATAAAAAATTAAAGCTATAATTACCTTGTAAAAATCACCGCTAACACGC

5 AAAGTGTAAAAATAAAAAATTAAAGCTATAATTACCTTGTAAAAATCACCGCTAACACGC

4 AAAGTGTAAAAATAAAAAATTAAAGCTATAATTACCTTGTAAAAATCACCGCTAACACGC

3 AAAGTGTAAAAATAAAAAATTAAAGCTATAATTACCTTGTAAAAATCACCGCTAACACGC

20 AAAGTGTAAAAATAAAAAATTAAAGCTATAATTACCTTGTAAAAATCACCGCTAACACGC

*******.**** ****.***.****************. ** *****************

1 AACCAAGCTCCTGTTACACTAGCCTCGACACCGTCCATTGAATCTAAAACACGCTCACAA

16 AACCAAGCTCCTGTTACACTAGCCTCGACACCGTCCATTGAATCTAAAACACGCTCACAA

15 AACCAAGCTCCTGTTACACTAGCCTCGACACCGTCCATTGAATCTAAAACACGCTCACAA

2 AACCAAGCTCCTGTTACACTAGCCTCGACACCGTCCATTGAATCTAAAACACGCTCACAA

10 AACCAAGCTCCTGTTACACTAGCTTCGACACCGTCCATTGAATCTAAAGTACGCTCACAA

17 AACCAAGCTCCTGTTACACTAGCTTCGACACCGTCCATTGAATCTAAAGTACGCTCACAA

14 AACCAAGCTCCTGTTACACTAGCTTCGACACCGTCCATTGAATCTAAAGTACGCTCACAA

13 AACCAAGCTCCTGTTACACTAGCTTCGACACCGTCCATTGAATCTAAAGTACGCTCACAA

12 AACCAAGCTCCTGTTACACTAGCTTCGACACCGTCCATTGAATCTAAAGTACGCTCACAA

8 AACCAAGCTCCTGTTACACTAGCTTCGACACCGTCCATTGAATCTAAAGTACGCTCACAA

7 AACCAAGCTCCTGTTACACTAGCTTCGACACCGTCCATTGAATCTAAAGTACGCTCACAA

19 AACCAAGCTCCTGTTACACTAGCTTCGACACCGTCCATTGAATCTAAAGTACGCTCACAA

18 AACCAAGCTCCTGTTACACTAGCTTCGACACCGTCCATTGAATCTAAAGTACGCTCACAA

22 AACCAAGCTCCTGTTACACTAGCTTCGACACCGTCCATTGAATCTAAAGTACGCTCACAA

21 AACCAAGCTCCTGTTACACTAGCTTCGACACCGTCCATTGAATCTAAAGTACGCTCACAA

9 AACCAAGCTCCTGTTACACTAGCTTCGACACCGTCCATTGAATCTAAAGTACGCTCACAA

6 AACCAAGCTCCTGTTACACTAGCTTCGACACCGTCCATTGAATCTAAAGTACGCTCACAA

5 AACCAAGCTCCTGTTACACTAGCTTCGACACCGTCCATTGAATCTAAAGTACGCTCACAA

4 AACCAAGCTCCTGTTACACTAGCTTCGACACCGTCCATTGAATCTAAAGTACGCTCACAA

3 AACCAAGCTCCTGTTACACTAGCTTCGACACCGTCCATTGAATCTAAAGTACGCTCACAA

20 AACCAAGCTCCTGTTACACTAGCTTCGACACCGTCCATTGAATCTAAAGTACGCTCACAA

***********************.************************..**********

1 ACGTCCATTGCTTCTTTTGCCTTATCTAAATTCCCAAGCAAAAGATCATAATGAGCGATT

16 ACGTCCATTGCTTCTTTTGCCTCATCTAAATTCCCAAGCAAAAGATCATAATGAGCGATT

15 ACGTCCATTGCTTCTTTTGCCTTATCTAAATTCCCAAGCAAAAGATCATAATGAGCGATT

2 ACGTCCATTGCTTCTTTTGCCTTATCTAAATTCCCAAGCAAAAGATCATAATGAGCGATT

10 ACGTCCATTGCTTCTTTTGCCTTATCTAAATTTCCAAGCAAGAGGTCATAATGAGCGATT

17 ACGTCCATTGCTTCTTTTGCCTTATCTAAATTTCCAAGCAAGAGGTCATAATGAGCGATT

14 ACGTCCATTGCTTCTTTTGCCTTATCTAAATTTCCAAGCAAGAGGTCATAATGAGCGATT

13 ACGTCCATTGCTTCTTTTGCCTTATCTAAATTTCCAAGCAAGAGGTCATAATGAGCGATT

12 ACGTCCATTGCTTCTTTTGCCTTATCTAAATTTCCAAGCAAGAGGTCATAATGAGCGATT

8 ACGTCCATTGCTTCTTTTGCCTTATCTAAATTTCCAAGCAAGAGGTCATAATGAGCGATT

7 ACGTCCATTGCTTCTTTTGCCTTATCTAAATTTCCAAGCAAGAGGTCATAATGAGCGATT

19 ACGTCCATTGCTTCTTTTGCCTTATCTAAATTTCCAAGCAAGAGGTCATAATGAGCGATT

18 ACGTCCATTGCTTCTTTTGCCTTATCTAAATTTCCAAGCAAGAGGTCATAATGAGCGATT

22 ACGTCCATTGCTTCTTTTGCCTTATCTAAATTTCCAAGCAAGAGGTCATAATGAGCGATT

21 ACGTCCATTGCTTCTTTTGCCTTATCTAAATTTCCAAGCAAGAGGTCATAATGAGCGATT

9 ACGTCCATTGCTTCTTTTGCCTTATCTAAATTTCCAAGCAAGAGGTCATAATGAGCGATT

6 ACGTCCATTGCTTCTTTTGCCTTATCTAAATTTCCAAGCAAGAGGTCATAATGAGCGATT

5 ACGTCCATTGCTTCTTTTGCCTTATCTAAATTTCCAAGCAAGAGGTCATAATGAGCGATT

4 ACGTCCATTGCTTCTTTTGCCTTATCTAAATTTCCAAGCAAGAGGTCATAATGAGCGATT

3 ACGTCCATTGCTTCTTTTGCCTTATCTAAATTTCCAAGCAAGAGGTCATAATGAGCGATT

20 ACGTCCATTGCTTCTTTTGCCTTATCTAAATTTCCAAGCAAGAGGTCATAATGAGCGATT

**********************.*********.********.**.***************

1 GAGATCCTAGCCAAAACGTAAGCCTCAGCTTCAGTTTTGGTTGGTGGTTGTTCTTCATCG

16 GAGATCCTAGCCAAAACGTAAGCCTCAGCTTCAGTTTTGGTTGGTGGTTGTTCTTCATCG

15 GAGATCCTAGCCAAAACGTAAGCCTCAGCTTCAGTTTTGGTTGGTGGTTGTTCTTCATCG

2 GAGATCCTAGCCAAAACGTAAGCCTCAGCTTCAGTTTTGGTTGGTGGTTGTTCTTCATCG

10 GAGATTCTAGCCAAAACGTAAGCCTCAGCTTCAGTTTTAGTTGGTGATTGTTCTTCATCG

17 GAGATTCTAGCCAAAACGTAAGCCTCAGCTTCAGTTTTAGTTGGTGATTGTTCTTCATCG

14 GAGATTCTAGCCAAAACGTAAGCCTCAGCTTCAGTTTTAGTTGGTGATTGTTCTTCATCG

13 GAGATTCTAGCCAAAACGTAAGCCTCAGCTTCAGTTTTAGTTGGTGATTGTTCTTCATCG

12 GAGATTCTAGCCAAAACGTAAGCCTCAGCTTCAGTTTTAGTTGGTGATTGTTCTTCATCG

8 GAGATTCTAGCCAAAACGTAAGCCTCAGCTTCAGTTTTAGTTGGTGATTGTTCTTCATCG

7 GAGATTCTAGCCAAAACGTAAGCCTCAGCTTCAGTTTTAGTTGGTGATTGTTCTTCATCG

19 GAGATTCTAGCCAAAACGTAAGCCTCAGCTTCAGTTTTAGTTGGTGATTGTTCTTCATCG

18 GAGATTCTAGCCAAAACGTAAGCCTCAGCTTCAGTTTTAGTTGGTGATTGTTCTTCATCG

22 GAGATTCTAGCCAAAACGTAAGCCTCAGCTTCAGTTTTAGTTGGTGATTGTTCTTCATCG

21 GAGATTCTAGCCAAAACGTAAGCCTCAGCTTCAGTTTTAGTTGGTGATTGTTCTTCATCG

9 GAGATTCTAGCCAAAACGTAAGCCTCAGCTTCAGTTTTAGTTGGTGATTGTTCTTCATCG

6 GAGATTCTAGCCAAAACGTAAGCCTCAGCTTCAGTTTTAGTTGGTGATTGTTCTTCATCG

5 GAGATTCTAGCCAAAACGTAAGCCTCAGCTTCAGTTTTAGTTGGTGATTGTTCTTCATCG

4 GAGATTCTAGCCAAAACGTAAGCCTCAGCTTCAGTTTTAGTTGGTGATTGTTCTTCATCG

3 GAGATTCTAGCCAAAACGTAAGCCTCAGCTTCAGTTTTAGTTGGTGATTGTTCTTCATCG

20 GAGATTCTAGCCAAAACGTAAGCCTCAGCTTCAGTTTTAGTTGGTGATTGTTCTTCATCG

*****.********************************.*******.*************

1 AATGGTTTAGCTGTGCTTGGTTTGAGGGAAGGTGGGAGTGCAGCACTTGGGGTTGGAAGT

16 AATGGTTTAGCTGTGCTTGGTTTGAGGGAAGGTGGGAGTGCAGCACTTGGGGTTGGAAGT

15 AATGGTTTAGCTGTGCTTGGTTTGAGGGAAGGTGGGAGTGCAGCACTTGGGGTTGGAAGT

2 AATGGTTTAGCTGTGCTTGGTTTGAGGGAAGGTGGGAGTGCAGCATTTGGGGTTGGAAGT

10 AATGGTTTAGCTGTGCTTGGTTTGAGGGAAGGTGAGAGTGCAGCTCTTGGGGTTGGAAGT

17 AATGGTTTAGCTGTGCTTGGTTTGAGGGAAGGTGAGAGTGCAGCTCTTGGGGTTGGAAGT

14 AATGGTTTAGCTGTGCTTGGTTTGAGGGAAGGTGAGAGTGCAGCTCTTGGGGTTGGAAGT

13 AATGGTTTAGCTGTGCTTGGTTTGAGGGAAGGTGAGAGTGCAGCTCTTGGGGTTGGAAGT

12 AATGGTTTAGCTGTGCTTGGTTTGAGGGAAGGTGAGAGTGCAGCTCTTGGGGTTGGAAGT

8 AATGGTTTAGCTGTGCTTGGTTTGAGGGAAGGTGAGAGTGCAGCTCTTGGGGTTGGAAGT

7 AATGGTTTAGCTGTGCTTGGTTTGAGGGAAGGTGAGAGTGCAGCTCTTGGGGTTGGAAGT

19 AATGGTTTAGCTGTGCTTGGTTTGAGGGAAGGTGAGAGTGCAGCTCTTGGGGTTGGAAGT

18 AATGGTTTAGCTGTGCTTGGTTTGAGGGAAGGTGAGAGTGCAGCTCTTGGGGTTGGAAGT

22 AATGGTTTAGCTGTGCTTGGTTTGAGGGAAGGTGAGAGTGCAGCTCTTGGGGTTGGAAGT

21 AATGGTTTAGCTGTGCTTGGTTTGAGGGAAGGTGAGAGTGCAGCTCTTGGGGTTGGAAGT

9 AATGGTTTAGCTGTGCTTGGTTTGAGGGAAGGTGAGAGTGCAGCTCTTGGGGTTGGAAGT

6 AATGGTTTAGCTGTGCTTGGTTTGAGGGAAGGTGAGAGTGCAGCTCTTGGGGTTGGAAGT

5 AATGGTTTAGCTGTGCTTGGTTTGAGGGAAGGTGAGAGTGCAGCTCTTGGGGTTGGAAGT

4 AATGGTTTAGCTGTGCTTGGTTTGAGGGAAGGTGAGAGTGCAGCTCTTGGGGTTGGAAGT

3 AATGGTTTAGCTGTGCTTGGTTTGAGGGAAGGTGAGAGTGCAGCTCTTGGGGTTGGAAGT

20 AATGGTTTAGCTGTGCTTGGTTTGAGGGAAGGTGAGAGTGCAGCTCTTGGGGTTGGAAGT

**********************************.********* .**************

1 GAGCCCAACAATGATTGCAAGAAGCTCAAAGATTCTTGTGGGTCCATTTGCTGAGCAACC

16 GAGCCCAACAATGATTGCAAGAAGCTCAAAGATTCTTGTGGGTCCATTTGCTGAGCAACC

15 GAGCCCAACAATGATTGCAAGAAGCTCAAAGATTCTTGTGGGTCCATTTGCTGAGCAACC

2 GAGCCCAACAATGATTGCAAGAAGCTCAAAGATTCTTGTGGGTCCATTTGCTGAGCAACC

10 GAGCCCAACAATGATTGCAAGAAGCTCAATGATTCTTGTGGGTCCATTTGCTGAGCAACC

17 GAGCCCAACAATGATTGCAAGAAGCTCAATGATTCTTGTGGGTCCATTTGCTGAGCAACC

14 GAGCCCAACAATGATTGCAAGAAGCTCAATGATTCTTGTGGGTCCATTTGCTGAGCAACC

13 GAGCCCAACAATGATTGCAAGAAGCTCAATGATTCTTGTGGGTCCATTTGCTGAGCAACC

12 GAGCCCAACAATGATTGCAAGAAGCTCAATGATTCTTGTGGGTCCATTTGCTGAGCAACC

8 GAGCCCAACAATGATTGCAAGAAGCTCAATGATTCTTGTGGGTCCATTTGCTGAGCAACC

7 GAGCCCAACAATGATTGCAAGAAGCTCAATGATTCTTGTGGGTCCATTTGCTGAGCAACC

19 GAGCCCAACAATGATTGCAAGAAGCTCAATGATTCTTGTGGGTCCATTTGCTGAGCAACC

18 GAGCCCAACAATGATTGCAAGAAGCTCAATGATTCTTGTGGGTCCATTTGCTGAGCAACC

22 GAGCCCAACAATGATTGCAAGAAGCTCAATGATTCTTGTGGGTCCATTTGCTGAGCAACC

21 GAGCCCAACAATGATTGCAAGAAGCTCAATGATTCTTGTGGGTCCATTTGCTGAGCAACC

9 GAGCCCAACAATGATTGCAAGAAGCTCAATGATTCTTGTGGGTCCATTTGCTGAGCAACC

6 GAGCCCAACAATGATTGCAAGAAGCTCAATGATTCTTGTGGGTCCATTTGCTGAGCAACC

5 GAGCCCAACAATGATTGCAAGAAGCTCAATGATTCTTGTGGGTCCATTTGCTGAGCAACC

4 GAGCCCAACAATGATTGCAAGAAGCTCAATGATTCTTGTGGGTCCATTTGCTGAGCAACC

3 GAGCCCAACAATGATTGCAAGAAGCTCAATGATTCTTGTGGGTCCATTTGCTGAGCAACC

20 GAGCCCAACAATGATTGCAAGAAGCTCAATGATTCTTGTGGGTCCATTTGCTGAGCAACC

***************************** ******************************

1 CGCACACCAATCTCAACCAAACGAAGCTGATTAAGCTTGTCTTTGAAACTGATAATGAAA

16 CGCACACCAATCTCAACCAAACGAAGCTGATTAAGCTTGTCTTTGAAACTGATAATGAAA

15 CGCACCCCAATCTCAACCAAACGAAGCTGATTAAGCTTGTCTTTGAAACTGATAATGAAA

2 CGCACCCCAATCTCAACCAAACGAAGCTGATTAAGCTTGTCTTTGAAACTGATAATGAAA

10 CGCACACCAATCTCGACCAAACGAAGCTGATTAAGCTTGTCTTTGAAACTGGTAATGAAA

17 CGCACACCAATCTCGACCAAACGAAGCTGATTAAGCTTGTCTTTGAAACTGGTAATGAAA

14 CGCACACCAATCTCGACCAAACGAAGCTGATTAAGCTTGTCTTTGAAACTGGTAATGAAA

13 CGCACACCAATCTCGACCAAACGAAGCTGATTAAGCTTGTCTTTGAAACTGGTAATGAAA

12 CGCACACCAATCTCGACCAAACGAAGCTGATTAAGCTTGTCTTTGAAACTGGTAATGAAA

8 CGCACACCAATCTCGACCAAACGAAGCTGATTAAGCTTGTCTTTGAAACTGGTAATGAAA

7 CGCACACCAATCTCGACCAAACGAAGCTGATTAAGCTTGTCTTTGAAACTGGTAATGAAA

19 CGCACACCAATCTCGACCAAACGAAGCTGATTAAGCTTGTCTTTGAAACTGGTAATGAAA

18 CGCACACCAATCTCGACCAAACGAAGCTGATTAAGCTTGTCTTTGAAACTGGTAATGAAA

22 CGCACACCAATCTCGACCAAACGAAGCTGATTAAGCTTGTCTTTGAAACTGGTAATGAAA

21 CGCACACCAATCTCGACCAAACGAAGCTGATTAAGCTTGTCTTTGAAACTGGTAATGAAA

9 CGCACACCAATCTCGACCAAACGAAGCTGATTAAGCTTGTCTTTGAAACTGGTAATGAAA

6 CGCACACCAATCTCGACCAAACGAAGCTGATTAAGCTTGTCTTTGAAACTGGTAATGAAA

5 CGCACACCAATCTCGACCAAACGAAGCTGATTAAGCTTGTCTTTGAAACTGGTAATGAAA

4 CGCACACCAATCTCGACCAAACGAAGCTGATTAAGCTTGTCTTTGAAACTGGTAATGAAA

3 CGCACACCAATCTCGACCAAACGAAGCTGATTAAGCTTGTCTTTGAAACTGGTAATGAAA

20 CGCACACCAATCTCGACCAAACGAAGCTGATTAAGCTTGTCTTTGAAACTGGTAATGAAA

***** ********.************************************.********

1 TTGTTCCATAGCCCTACTAAGTATTTTGATATTTCATCCTTTTGTATCAAGCCATTCAAA

16 TTGTTCCATAGCCCTACTAAGTATTTTGATATTTCATCCTTTTGTATCAAGCCATTCAAA

15 TTGTTCCATAGCCCTACTAAGTATTTTGATATTTCATCCTTTTGTATCAAGCCATTCAAA

2 TTGTTCCATAGCCCTACTAAGTATTTTGATATTTCATCCTTTTGTATCAAGCCATTCAAA

10 TTGTTCCATAGTCCCACTAAGTATTTTGATATTTCATCCTTTTGTATCAAACCTTTCAAA

17 TTGTTCCATAGTCCCACTAAGTATTTTGATATTTCATCCTTTTGTATCAAACCTTTCAAA

14 TTGTTCCATAGTCCCACTAAGTATTTTGATATTTCATCCTTTTGTATCAAACCTTTCAAA

13 TTGTTCCATAGTCCCACTAAGTATTTTGATATTTCATCCTTTTGTATCAAACCTTTCAAA

12 TTGTTCCATAGTCCCACTAAGTATTTTGATATTTCATCCTTTTGTATCAAACCTTTCAAA

8 TTGTTCCATAGTCCCACTAAGTATTTTGATATTTCATCCTTTTGTATCAAACCTTTCAAA

7 TTGTTCCATAGTCCCACTAAGTATTTTGATATTTCATCCTTTTGTATCAAACCTTTCAAA

19 TTGTTCCATAGTCCCACTAAGTATTTTGATATTTCATCCTTTTGTATCAAACCTTTCAAA

18 TTGTTCCATAGTCCCACTAAGTATTTTGATATTTCATCCTTTTGTATCAAACCTTTCAAA

22 TTGTTCCATAGTCCCACTAAGTATTTTGATATTTCATCCTTTTGTATCAAACCTTTCAAA

21 TTGTTCCATAGTCCCACTAAGTATTTTGATATTTCATCCTTTTGTATCAAACCTTTCAAA

9 TTGTTCCATAGTCCCACTAAGTATTTTGATATTTCATCCTTTTGTATCAAACCTTTCAAA

6 TTGTTCCATAGTCCCACTAAGTATTTTGATATTTCATCCTTTTGTATCAAACCTTTCAAA

5 TTGTTCCATAGTCCCACTAAGTATTTTGATATTTCATCCTTTTGTATCAAACCTTTCAAA

4 TTGTTCCATAGTCCCACTAAGTATTTTGATATTTCATCCTTTTGTATCAAACCTTTCAAA

3 TTGTTCCATAGTCCCACTAAGTATTTTGATATTTCATCCTTTTGTATCAAACCTTTCAAA

20 TTGTTCCATAGTCCCACTAAGTATTTTGATATTTCATCCTTTTGTATCAAACCTTTCAAA

***********.**.***********************************.** ******

1 GTCTTTGTGAGTTGATGGTATAAGCTTTGTGGCTAGTTAGCTTTCTTTCTTTCTGCAACT

16 GTCTTTGTGAGTTGATGGTATAAGCTTTGTGGCTAGTTAGCTTTCTTTCTTTCTGCAACT

15 GTCTTTGTGAGTTGATGGTATAAGCTTTGTGGCTAGTTAGCTTTCTTTCTTTCTGCAACT

2 GTCTTTGTGAGTTGATGGTATAAGCTTTGTGGCTAGTTAGCTTTCTTTCTTTCTGCAACT

10 GTCTTTGTGAGTTGATGGTATAAGCTTTGTGGCTAGTTAGCTTTCTTTCTTTCTGTAAAT

17 GTCTTTGTGAGTTGATGGTATAAGCTTTGTGGCTAGTTAGCTTTCTTTCTTTCTGTAAAT

14 GTCTTTGTGAGTTGATGGTATAAGCTTTGTGGCTAGTTAGCTTTCTTTCTTTCTGTAAAT

13 GTCTTTGTGAGTTGATGGTATAAGCTTTGTGGCTAGTTAGCTTTCTTTCTTTCTGTAAAT

12 GTCTTTGTGAGTTGATGGTATAAGCTTTGTGGCTAGTTAGCTTTCTTTCTTTCTGTAAAT

8 GTCTTTGTGAGTTGATGGTATAAGCTTTGTGGCTAGTTAGCTTTCTTTCTTTCTGTAAAT

7 GTCTTTGTGAGTTGATGGTATAAGCTTTGTGGCTAGTTAGCTTTCTTTCTTTCTGTAAAT

19 GTCTTTGTGAGTTGATGGTATAAGCTTTGTGGCTAGTTAGCTTTCTTTCTTTCTGTAAAT

18 GTCTTTGTGAGTTGATGGTATAAGCTTTGTGGCTAGTTAGCTTTCTTTCTTTCTGTAAAT

22 GTCTTTGTGAGTTGATGGTATAAGCTTTGTGGCTAGTTAGCTTTCTTTCTTTCTGTAAAT

21 GTCTTTGTGAGTTGATGGTATAAGCTTTGTGGCTAGTTAGCTTTCTTTCTTTCTGTAAAT

9 GTCTTTGTGAGTTGATGGTATAAGCTTTGTGGCTAGTTAGCTTTCTTTCTTTCTGTAAAT

6 GTCTTTGTGAGTTGATGGTATAAGCTTTGTGGCTAGTTAGCTTTCTTTCTTTCTGTAAAT

5 GTCTTTGTGAGTTGATGGTATAAGCTTTGTGGCTAGTTAGCTTTCTTTCTTTCTGTAAAT

4 GTCTTTGTGAGTTGATGGTATAAGCTTTGTGGCTAGTTAGCTTTCTTTCTTTCTGTAAAT

3 GTCTTTGTGAGTTGATGGTATAAGCTTTGTGGCTAGTTAGCTTTCTTTCTTTCTGTAAAT

20 GTCTTTGTGAGTTGATGGTATAAGCTTTGTGGCTAGTTAGCTTTCTTTCTTTCTGTAAAT

*******************************************************.** *

1 TGTACAAACCGCTTATTGAAATAATCCTCTAATTTACTCAAATCTTCTTGTATCGTTACC

16 TGTACAAACCGCTTATTGAAATAATCCTCTAATTTACTCAAATCTTCTTGTATCGTTACC

15 TGTACAAACCGCTTATTGAAATAATCCTCTAATTTACTCAAATCTTCTTGTATCGTTACC

2 TGTACAAACCGCTTATTGAAATAATCCTCTAATTTACTCAAATCTTCTTGTATCGTTACC

10 TGTACGAACCGCTTATTGAAATAATCCTCCAAATTCCTCAAATCTTCTTGTATCGTTACT

17 TGTACGAACCGCTTATTGAAATAATCCTCCAAATTCCTCAAATCTTCTTGTATCGTTACT

14 TGTACGAACCGCTTATTGAAATAATCCTCCAAATTCCTCAAATCTTCTTGTATCGTTACT

13 TGTACGAACCGCTTATTGAAATAATCCTCCAAATTCCTCAAATCTTCTTGTATCGTTACT

12 TGTACGAACCGCTTATTGAAATAATCCTCCAAATTCCTCAAATCTTCTTGTATCGTTACT

8 TGTACGAACCGCTTATTGAAATAATCCTCCAAATTCCTCAAATCTTCTTGTATCGTTACT

7 TGTACGAACCGCTTATTGAAATAATCCTCCAAATTCCTCAAATCTTCTTGTATCGTTACT

19 TGTACGAACCGCTTATTGAAATAATCCTCCAAATTCCTCAAATCTTCTTGTATCGTTACT

18 TGTACGAACCGCTTATTGAAATAATCCTCCAAATTCCTCAAATCTTCTTGTATCGTTACT

22 TGTACGAACCGCTTATTGAAATAATCCTCCAAATTCCTCAAATCTTCTTGTATCGTTACT

21 TGTACGAACCGCTTATTGAAATAATCCTCCAAATTCCTCAAATCTTCTTGTATCGTTACT

9 TGTACGAACCGCTTATTGAAATAATCCTCCAAATTCCTCAAATCTTCTTGTATCGTTACT

6 TGTACGAACCGCTTATTGAAATAATCCTCCAAATTCCTCAAATCTTCTTGTATCGTTACT

5 TGTACGAACCGCTTATTGAAATAATCCTCCAAATTCCTCAAATCTTCTTGTATCGTTACT

4 TGTACGAACCGCTTATTGAAATAATCCTCCAAATTCCTCAAATCTTCTTGTATCGTTACT

3 TGTACGAACCGCTTATTGAAATAATCCTCCAAATTCCTCAAATCTTCTTGTATCGTTACT

20 TGTACGAACCGCTTATTGAAATAATCCTCCAAATTCCTCAAATCTTCTTGTATCGTTACT

*****.***********************.** ** ***********************.

1 GGCGCTTCTCCTTTAATTTTATTTATGTAAGCTGTTGTACTCAT

16 GGCGCTTCTCCTTTAATTTTATTTATGTAAGCTGTTGTACTCAT

15 GGCGCTTCTCCTTTAATTTTATTTATGTAAGCTGTTGTACTCAT

2 GGCGCTTCTCCTTTAATTTTATTTATGTAAGCTGTTGTACTCAT

10 GGCGCTTCTCCTTTAATTTTATTTATATAGGATGTTGTACTCAT

17 GGCGCTTCTCCTTTAATTTTATTTATATAGGATGTTGTACTCAT

14 GGCGCTTCTCCTTTAATTTTATTTATATAGGATGTTGTACTCAT

13 GGCGCTTCTCCTTTAATTTTATTTATATAGGATGTTGTACTCAT

12 GGCGCTTCTCCTTTAATTTTATTTATATAGGATGTTGTACTCAT

8 GGCGCTTCTCCTTTAATTTTATTTATATAGGATGTTGTACTCAT

7 GGCGCTTCTCCTTTAATTTTATTTATATAGGATGTTGTACTCAT

19 GGCGCTTCTCCTTTAATTTTATTTATATAGGATGTTGTACTCAT

18 GGCGCTTCTCCTTTAATTTTATTTATATAGGATGTTGTACTCAT

22 GGCGCTTCTCCTTTAATTTTATTTATATAGGATGTTGTACTCAT

21 GGCGCTTCTCCTTTAATTTTATTTATATAGGATGTTGTACTCAT

9 GGCGCTTCTCCTTTAATTTTATTTATATAGGATGTTGTACTCAT

6 GGCGCTTCTCCTTTAATTTTATTTATATAGGATGTTGTACTCAT

5 GGCGCTTCTCCTTTAATTTTATTTATATAGGATGTTGTACTCAT

4 GGCGCTTCTCCTTTAATTTTATTTATATAGGATGTTGTACTCAT

3 GGCGCTTCTCCTTTAATTTTATTTATATAGGATGTTGTACTCAT

20 GGCGCTTCTCCTTTAATTTTATTTATATAGGATGTTGTACTCAT

**************************.**.* ************

**INVERTED REGION**

Sequences 1, 2, 15 And 16 Are in reverse-complement orientAtion.

1RC TGCTAGATATTATCCTTATGACAGACCTCAATGATTGA-------------------ACG

16RC TGCTAGATATTATCCTTATGACAGACCTCAATGATTGA-------------------ACG

15RC TGCTAGATATTATCCTTATGACAGACCTCAATGATTGA-------------------ACG

2RC TGCTAGATATTATCCTTATGACAGACCTCAATGATTGA-------------------ACG

10 --------------------------GTTGGTAGTTGAATCGACAAGGACGTCGCACACG

17 --------------------------GTTGGTAGTTGAATCGACAAGGACGTCGCACACG

14 --------------------------GTTGGTAGTTGAATCGACAAGGACGTCGCACACG

13 --------------------------GTTGGTAGTTGAATCGACAAGGACGTCGCACACG

12 --------------------------GTTGGTAGTTGAATCGACAAGGACGTCGCACACG

8 --------------------------GTTGGTAGTTGAATCGACAAGGACGTCGCACACG

7 --------------------------GTTGGTAGTTGAATCGACAAGGACGTCGCACACG

19 --------------------------GTTGGTAGTTGAATCGACAAGGACGTCGCACACG

18 --------------------------GTTGGTAGTTGAATCGACAAGGACGTCGCACACG

22 --------------------------GTTGGTAGTTGAATCGACAAGGACGTCGCACACG

21 --------------------------GTTGGTAGTTGAATCGACAAGGACGTCGCACACG

9 --------------------------GTTGGTAGTTGAATCGACAAGGACGTCGCACACG

6 --------------------------GTTGGTAGTTGAATCGACAAGGACGTCGCACACG

5 --------------------------GTTGGTAGTTGAATCGACAAGGACGTCGCACACG

4 --------------------------GTTGGTAGTTGAATCGACAAGGACGTCGCACACG

3 --------------------------GTTGGTAGTTGAATCGACAAGGACGTCGCACACG

20 --------------------------GTTGGTAGTTGAATCGACAAGGACGTCGCACACG

*...*..**** ***

1RC ACATTGTGTTATATGTGAAGATTAAACGAAGAATGGCAAAAAGGAGTACACATTATTTCT

16RC ACATTGTGTTATATGTGAAGATTAAACGAAGAATGGCAAAAAGGAGTACACATTATTTCT

15RC ACATTGTGTTATATGTGAAGATTAAACGAAGAATGGCAAAAAGGAGTACACATTATTTCT

2RC ACATTGTGTTATATGTGAAGATTAAACGAAGAATGGCAAAAAGGAGTACACATTATTTCT

10 TCATTGTGT--TATGTGAAGATTAAATGAAGAATGGCAAAAAGGAGTACACATTGTTTCT

17 TCATTGTGT--TATGTGAAGATTAAATGAAGAATGGCAAAAAGGAGTACACATTGTTTCT

14 TCATTGTGT--TATGTGAAGATTAAATGAAGAATGGCAAAAAGGAGTACACATTGTTTCT

13 TCATTGTGT--TATGTGAAGATTAAATGAAGAATGGCAAAAAGGAGTACACATTGTTTCT

12 TCATTGTGT--TATGTGAAGATTAAATGAAGAATGGCAAAAAGGAGTACACATTGTTTCT

8 TCATTGTGT--TATGTGAAGATTAAATGAAGAATGGCAAAAAGGAGTACACATTGTTTCT

7 TCATTGTGT--TATGTGAAGATTAAATGAAGAATGGCAAAAAGGAGTACACATTGTTTCT

19 TCATTGTGT--TATGTGAAGATTAAATGAAGAATGGCAAAAAGGAGTACACATTGTTTCT

18 TCATTGTGT--TATGTGAAGATTAAATGAAGAATGGCAAAAAGGAGTACACATTGTTTCT

22 TCATTGTGT--TATGTGAAGATTAAATGAAGAATGGCAAAAAGGAGTACACATTGTTTCT

21 TCATTGTGT--TATGTGAAGATTAAATGAAGAATGGCAAAAAGGAGTACACATTGTTTCT

9 TCATTGTGT--TATGTGAAGATTAAATGAAGAATGGCAAAAAGGAGTACACATTGTTTCT

6 TCATTGTGT--TATGTGAAGATTAAATGAAGAATGGCAAAAAGGAGTACACATTGTTTCT

5 TCATTGTGT--TATGTGAAGATTAAATGAAGAATGGCAAAAAGGAGTACACATTGTTTCT

4 TCATTGTGT--TATGTGAAGATTAAATGAAGAATGGCAAAAAGGAGTACACATTGTTTCT

3 TCATTGTGT--TATGTGAAGATTAAATGAAGAATGGCAAAAAGGAGTACACATTGTTTCT

20 TCATTGTGT--TATGTGAAGATTAAATGAAGAATGGCAAAAAGGAGTACACATTGTTTCT

******** ***************.***************************.*****

1RC ATAATTTTTATATTCGCTTCCAAAGAACTTGATCAGATAATTCTCTTCATCTAAATTCGG

16RC ATAATTTTTATATTCGCTTCCAAAGAACTTGATCAGATAATTCTCTTCATCTAAATTCGG

15RC ATAATTTTTATATTCGCTTCCAAAGAACTTGATCAGATAATTCTCTTCATCTAAATTCGG

2RC ATAATTTTTATATTCGCTTCCAAAGAACTTGATCAGATAATTCTCTTCATCTAAATTCGG

10 GTAATTTTTATATTCGTTTCCAAAGAACTTGATGAGATATTTCTCTTCATCTAAAGTCGG

17 GTAATTTTTATATTCGTTTCCAAAGAACTTGATGAGATATTTCTCTTCATCTAAAGTCGG

14 GTAATTTTTATATTCGTTTCCAAAGAACTTGATGAGATATTTCTCTTCATCTAAAGTCGG

13 GTAATTTTTATATTCGTTTCCAAAGAACTTGATGAGATATTTCTCTTCATCTAAAGTCGG

12 GTAATTTTTATATTCGTTTCCAAAGAACTTGATGAGATATTTCTCTTCATCTAAAGTCGG

8 GTAATTTTTATATTCGTTTCCAAAGAACTTGATGAGATATTTCTCTTCATCTAAAGTCGG

7 GTAATTTTTATATTCGTTTCCAAAGAACTTGATGAGATATTTCTCTTCATCTAAAGTCGG

19 GTAATTTTTATATTCGTTTCCAAAGAACTTGATGAGATATTTCTCTTCATCTAAAGTCGG

18 GTAATTTTTATATTCGTTTCCAAAGAACTTGATGAGATATTTCTCTTCATCTAAAGTCGG

22 GTAATTTTTATATTCGTTTCCAAAGAACTTGATGAGATATTTCTCTTCATCTAAAGTCGG

21 GTAATTTTTATATTCGTTTCCAAAGAACTTGATGAGATATTTCTCTTCATCTAAAGTCGG

9 GTAATTTTTATATTCGTTTCCAAAGAACTTGATGAGATATTTCTCTTCATCTAAAGTCGG

6 GTAATTTTTATATTCGTTTCCAAAGAACTTGATGAGATATTTCTCTTCATCTAAAGTCGG

5 GTAATTTTTATATTCGTTTCCAAAGAACTTGATGAGATATTTCTCTTCATCTAAAGTCGG

4 GTAATTTTTATATTCGTTTCCAAAGAACTTGATGAGATATTTCTCTTCATCTAAAGTCGG

3 GTAATTTTTATATTCGTTTCCAAAGAACTTGATGAGATATTTCTCTTCATCTAAAGTCGG

20 GTAATTTTTATATTCGTTTCCAAAGAACTTGATGAGATATTTCTCTTCATCTAAAGTCGG

.***************.**************** ***** *************** ****

1RC TTCAATTGAGAATCGAAGGAAAAAGTTTCACTTACGTTTAATCCGGTCAGAAAAGAATCT

16RC TTCAATTGAGAATCGAAGGAAAAAGTTTCACTTACGTTTAATCCGGTCAGAAAAGAATCT

15RC TTCAATTGAGAATCGAAGGAAAAAGTTTCACTTACGTTTAATCCGGTCAGAAAAGAATCT

2RC TTCAATTGAGAATCGAAGGAAAAAGTTTCACTTACGTTTAATCCGGTCAGAAAAGAATCT

10 TTCAATTGAGTATCGATGAAAAAGG-TTCACTTACGTTTAATCCGGTCAGAAAAGAATCT

17 TTCAATTGAGTATCGATGAAAAAGG-TTCACTTACGTTTAATCCGGTCAGAAAAGAATCT

14 TTCAATTGAGTATCGATGAAAAAGG-TTCACTTACGTTTAATCCGGTCAGAAAAGAATCT

13 TTCAATTGAGTATCGATGAAAAAGG-TTCACTTACGTTTAATCCGGTCAGAAAAGAATCT

12 TTCAATTGAGTATCGATGAAAAAGG-TTCACTTACGTTTAATCCGGTCAGAAAAGAATCT

8 TTCAATTGAGTATCGATGAAAAAGG-TTCACTTACGTTTAATCCGGTCAGAAAAGAATCT

7 TTCAATTGAGTATCGATGAAAAAGG-TTCACTTACGTTTAATCCGGTCAGAAAAGAATCT

19 TTCAATTGAGTATCGATGAAAAAGG-TTCACTTACGTTTAATCCGGTCAGAAAAGAATCT

18 TTCAATTGAGTATCGATGAAAAAGG-TTCACTTACGTTTAATCCGGTCAGAAAAGAATCT

22 TTCAATTGAGTATCGATGAAAAAGG-TTCACTTACGTTTAATCCGGTCAGAAAAGAATCT

21 TTCAATTGAGTATCGATGAAAAAGG-TTCACTTACGTTTAATCCGGTCAGAAAAGAATCT

9 TTCAATTGAGTATCGATGAAAAAGG-TTCACTTACGTTTAATCCGGTCAGAAAAGAATCT

6 TTCAATTGAGTATCGATGAAAAAGG-TTCACTTACGTTTAATCCGGTCAGAAAAGAATCT

5 TTCAATTGAGTATCGATGAAAAAGG-TTCACTTACGTTTAATCCGGTCAGAAAAGAATCT

4 TTCAATTGAGTATCGATGAAAAAGG-TTCACTTACGTTTAATCCGGTCAGAAAAGAATCT

3 TTCAATTGAGTATCGATGAAAAAGG-TTCACTTACGTTTAATCCGGTCAGAAAAGAATCT

20 TTCAATTGAGTATCGATGAAAAAGG-TTCACTTACGTTTAATCCGGTCAGAAAAGAATCT

********** ***** *.****.* **********************************

1RC TTGTAAGATGATGGCGAATCCCAAGAAAGATAATGGATTCGTTAGAATCATTTGAGTGCC

16RC TTGTAAGATGATGGCGAATCCCAAGAAAGATAATGGATTCGTTAGAATCATTTGAGTGCC

15RC TTGTAAGATGATGGCGAATCCCAAGAAAGATAATGGATTCGTTAGAATCATTTGAGTGCC

2RC TTGTAAGATGATGGCGAATCCCAAGAAAGATAATGGATTCGTTAGAATCATTTGAGTGCC

10 TTGTAGGATGATTGCGAATCCCAAGAAAGATAAGGGATTCGTTAAAATCATTTGAGTGCC

17 TTGTAGGATGATTGCGAATCCCAAGAAAGATAAGGGATTCGTTAAAATCATTTGAGTGCC

14 TTGTAGGATGATTGCGAATCCCAAGAAAGATAAGGGATTCGTTAAAATCATTTGAGTGCC

13 TTGTAGGATGATTGCGAATCCCAAGAAAGATAAGGGATTCGTTAAAATCATTTGAGTGCC

12 TTGTAGGATGATTGCGAATCCCAAGAAAGATAAGGGATTCGTTAAAATCATTTGAGTGCC

8 TTGTAGGATGATTGCGAATCCCAAGAAAGATAAGGGATTCGTTAAAATCATTTGAGTGCC

7 TTGTAGGATGATTGCGAATCCCAAGAAAGATAAGGGATTCGTTAAAATCATTTGAGTGCC

19 TTGTAGGATGATTGCGAATCCCAAGAAAGATAAGGGATTCGTTAAAATCATTTGAGTGCC

18 TTGTAGGATGATTGCGAATCCCAAGAAAGATAAGGGATTCGTTAAAATCATTTGAGTGCC

22 TTGTAGGATGATTGCGAATCCCAAGAAAGATAAGGGATTCGTTAAAATCATTTGAGTGCC

21 TTGTAGGATGATTGCGAATCCCAAGAAAGATAAGGGATTCGTTAAAATCATTTGAGTGCC

9 TTGTAGGATGATTGCGAATCCCAAGAAAGATAAGGGATTCGTTAAAATCATTTGAGTGCC

6 TTGTAGGATGATTGCGAATCCCAAGAAAGATAAGGGATTCGTTAAAATCATTTGAGTGCC

5 TTGTAGGATGATTGCGAATCCCAAGAAAGATAAGGGATTCGTTAAAATCATTTGAGTGCC

4 TTGTAGGATGATTGCGAATCCCAAGAAAGATAAGGGATTCGTTAAAATCATTTGAGTGCC

3 TTGTAGGATGATTGCGAATCCCAAGAAAGATAAGGGATTCGTTAAAATCATTTGAGTGCC

20 TTGTAGGATGATTGCGAATCCCAAGAAAGATAAGGGATTCGTTAAAATCATTTGAGTGCC

*****.****** ******************** **********.***************

1RC TAAAGCCCACAGGTAGAAACCTAAATAGGATGGATGGCGAACAAAGCTGTTTTATTCAGT

16RC TAAAGCCCACAGGTAGAAACCTAAATAGGATGGATGGCGAACAAAGCTGTTTTATTCAGT

15RC TAAAGCCCACAGGTAGAAACCTAAATAGGATGGATGGCGAACAAAGCTGTTTTATTCAGT

2RC TAAAGCCCACAGGTAGAAACCTAAATAGGATGGATGGCGAACAAAGCTGTTTTATTCAGT

10 TAAAGCCCACAGGTAGAAACCCAAATAGGATGGATGACGAACAAAGCTGTTTTATTCAGT

17 TAAAGCCCACAGGTAGAAACCCAAATAGGATGGATGACGAACAAAGCTGTTTTATTCAGT

14 TAAAGCCCACAGGTAGAAACCCAAATAGGATGGATGACGAACAAAGCTGTTTTATTCAGT

13 TAAAGCCCACAGGTAGAAACCCAAATAGGATGGATGACGAACAAAGCTGTTTTATTCAGT

12 TAAAGCCCACAGGTAGAAACCCAAATAGGATGGATGACGAACAAAGCTGTTTTATTCAGT

8 TAAAGCCCACAGGTAGAAACCCAAATAGGATGGATGACGAACAAAGCTGTTTTATTCAGT

7 TAAAGCCCACAGGTAGAAACCCAAATAGGATGGATGACGAACAAAGCTGTTTTATTCAGT

19 TAAAGCCCACAGGTAGAAACCCAAATAGGATGGATGACGAACAAAGCTGTTTTATTCAGT

18 TAAAGCCCACAGGTAGAAACCCAAATAGGATGGATGACGAACAAAGCTGTTTTATTCAGT

22 TAAAGCCCACAGGTAGAAACCCAAATAGGATGGATGACGAACAAAGCTGTTTTATTCAGT

21 TAAAGCCCACAAGTAGAAACCCAAATAGGATGGATGACGAACAAAGCTGTTTTATTCAGT

9 TAAAGCCCACAAGTAGAAACCCAAATAGGATGGATGACGAACAAAGCTGTTTTATTCAGT

6 TAAAGCCCACAAGTAGAAACCCAAATAGGATGGATGACGAACAAAGCTGTTTTATTCAGT

5 TAAAGCCCACAAGTAGAAACCCAAATAGGATGGATGACGAACAAAGCTGTTTTATTCAGT

4 TAAAGCCCACAAGTAGAAACCCAAATAGGATGGATGACGAACAAAGCTGTTTTATTCAGT

3 TAAAGCCCACAAGTAGAAACCCAAATAGGATGGATGACGAACAAAGCTGTTTTATTCAGT

20 TAAAGCCCACAAGTAGAAACCCAAATAGGATGGATGACGAACAAAGCTGTTTTATTCAGT

***********.*********.**************.***********************

1RC GTTTGACAAAAGGCCAATGAGACATTGCATACCTATATACGCCAGAAGTAACCAGCTCAT

16RC GTTTGACAAAAGGCCAATGAGACATTGCATACCTATATACGCCAGAAGTAACCAGCTCAT

15RC GTTTGACAAAAGGCCAATGAGACATTGCATACCTATATACGCCAGAAGTAACCAGCTCAT

2RC GTTTGACAAAAGGCCAATGAGACATTGCATACCTATATACGCCAGAAGTAACCAGCTCAT

10 GTTTGACAAAAGGCAAAAGAGGTATTACATACCTATATACGCCCGAAGTAACCAGTGCAT

17 GTTTGACAAAAGGCAAAAGAGGTATTACATACCTATATACGCCCGAAGTAACCAGTGCAT

14 GTTTGACAAAAGGCAAAAGAGGTATTACATACCTATATACGCCCGAAGTAACCAGTGCAT

13 GTTTGACAAAAGGCAAAAGAGGTATTACATACCTATATACGCCCGAAGTAACCAGTGCAT

12 GTTTGACAAAAGGCAAAAGAGGTATTACATACCTATATACGCCCGAAGTAACCAGTGCAT

8 GTTTGACAAAAGGCAAAAGAGGTATTACATACCTATATACGCCCGAAGTAACCAGTGCAT

7 GTTTGACAAAAGGCAAAAGAGGTATTACATACCTATATACGCCCGAAGTAACCAGTGCAT

19 GTTTGACAAAAGGCAAAAGAGGTATTACATACCTATATACGCCCGAAGTAACCAGTGCAT

18 GTTTGACAAAAGGCAAAAGAGGTATTACATACCTATATACGCCCGAAGTAACCAGTGCAT

22 GTTTGACAAAAGGCAAAAGAGGTATTACATACCTATATACGCCCGAAGTAACCAGTGCAT

21 GTTTGACAAAAGGCAAAAGAGGTATTACATACCTATATACGCCCGAAGTAACCAGTGCAT

9 GTTTGACAAAAGGCAAAAGAGGTATTACATACCTATATACGCCCGAAGTAACCAGTGCAT

6 GTTTGACAAAAGGCAAAAGAGGTATTACATACCTATATACGCCCGAAGTAACCAGTGCAT

5 GTTTGACAAAAGGCAAAAGAGGTATTACATACCTATATACGCCCGAAGTAACCAGTGCAT

4 GTTTGACAAAAGGCAAAAGAGGTATTACATACCTATATACGCCCGAAGTAACCAGTGCAT

3 GTTTGACAAAAGGCAAAAGAGGTATTACATACCTATATACGCCCGAAGTAACCAGTGCAT

20 GTTTGACAAAAGGCAAAAGAGGTATTACATACCTATATACGCCCGAAGTAACCAGTGCAT

************** ** ***..***.**************** ***********. ***

1RC GATCTTTTTGCTTAAAAGACGCAACATGGTGTGAGAAGCTTTCATTCGCGTGTACCATAG

16RC GATCTTTTTGCTTAAAAGACGCAACATGGTGTGAGAAGCTTTCATTCGCGTGTACCATAG

15RC GATCTTTTTGCTTAAAAGACGCAACATGGTGTGAGAAGCTTTCATTCGCGTGTACCATAG

2RC GATCTTTTTGCTTAAAAGACGCAACATGGTGTGAGAAGCTTTCATTCGCGTGTACCATAG

10 GATCTTTTTGCTTGAAAGACGCAACGTGGTGTGAAAAGCTCTCATTCGCGTGTACCATAG

17 GATCTTTTTGCTTGAAAGACGCAACGTGGTGTGAAAAGCTCTCATTCGCGTGTACCATAG

14 GATCTTTTTGCTTGAAAGACGCAACGTGGTGTGAAAAGCTCTCATTCGCGTGTACCATAG

13 GATCTTTTTGCTTGAAAGACGCAACGTGGTGTGAAAAGCTCTCATTCGCGTGTACCATAG

12 GATCTTTTTGCTTGAAAGACGCAACGTGGTGTGAAAAGCTCTCATTCGCGTGTACCATAG

8 GATCTTTTTGCTTGAAAGACGCAACGTGGTGTGAAAAGCTCTCATTCGCGTGTACCATAG

7 GATCTTTTTGCTTGAAAGACGCAACGTGGTGTGAAAAGCTCTCATTCGCGTGTACCATAG

19 GATCTTTTTGCTTGAAAGACGCAACGTGGTGTGAAAAGCTCTCATTCGCGTGTACCATAG

18 GATCTTTTTGCTTGAAAGACGCAACGTGGTGTGAAAAGCTCTCATTCGCGTGTACCATAG

22 GATCTTTTTGCTTGAAAGACGCAACGTGGTGTGAAAAGCTCTCATTCGCGTGTACCATAG

21 GATCTTTTTGCTTGAAAGACGCAACGTGGTGTGAAAAGCTCTCATTCGCGTGTACCATAG

9 GATCTTTTTGCTTGAAAGACGCAACGTGGTGTGAAAAGCTCTCATTCGCGTGTACCATAG

6 GATCTTTTTGCTTGAAAGACGCAACGTGGTGTGAAAAGCTCTCATTCGCGTGTACCATAG

5 GATCTTTTTGCTTGAAAGACGCAACGTGGTGTGAAAAGCTCTCATTCGCGTGTACCATAG

4 GATCTTTTTGCTTGAAAGACGCAACGTGGTGTGAAAAGCTCTCATTCGCGTGTACCATAG

3 GATCTTTTTGCTTGAAAGACGCAACGTGGTGTGAAAAGCTCTCATTCGCGTGTACCATAG

20 GATCTTTTTGCTTGAAAGACGCAACGTGGTGTGAAAAGCTCTCATTCGCGTGTACCATAG

*************.***********.********.*****.*******************

1RC CTAATGTACGCAACAACTGTCCAACTACGACTAGGCAAAAACCTGAATGACATTATTTTA

16RC CTAATGTACGCAACAACTGTCCAACTACGACTAGGCAAAAACCTGAATGACATTATTTTA

15RC CTAATGTACGCAACAACTGTCCAACTACGACTAGGCAAAAACCTGAATGACATTATTTTA

2RC CTAATGTACGCAACAACTGTCCAACTACGACTAGGCAAAAACCTGAATGACATTATTTTA

10 CTAACGTGCGTAGCAGCTGTCCAACTACGACTAAGCAAAAACCTTAATGATATCATTATA

17 CTAACGTGCGTAGCAGCTGTCCAACTACGACTAAGCAAAAACCTTAATGATATCATTATA

14 CTAACGTGCGTAGCAGCTGTCCAACTACGACTAAGCAAAAACCTTAATGATATCATTATA

13 CTAACGTGCGTAGCAGCTGTCCAACTACGACTAAGCAAAAACCTTAATGATATCATTATA

12 CTAACGTGCGTAGCAGCTGTCCAACTACGACTAAGCAAAAACCTTAATGATATCATTATA

8 CTAACGTGCGTAGCAGCTGTCCAACTACGACTAAGCAAAAACCTTAATGATATCATTATA

7 CTAACGTGCGTAGCAGCTGTCCAACTACGACTAAGCAAAAACCTTAATGATATCATTATA

19 CTAACGTGCGTAGCAGCTGTCCAACTACGACTAAGCAAAAACCTTAATGATATCATTATA

18 CTAACGTGCGTAGCAGCTGTCCAACTACGACTAAGCAAAAACCTTAATGATATCATTATA

22 CTAACGTGCGTAGCAGCTGTCCAACTACGACTAAGCAAAAACCTTAATGATATCATTATA

21 CTAACGTGCGTAGCAGCTGTCCAACTACGACTAAGCAAAAACCTTAATGATATCATTATA

9 CTAACGTGCGTAGCAGCTGTCCAACTACGACTAAGCAAAAACCTTAATGATATCATTATA

6 CTAACGTGCGTAGCAGCTGTCCAACTACGACTAAGCAAAAACCTTAATGATATCATTATA

5 CTAACGTGCGTAGCAGCTGTCCAACTACGACTAAGCAAAAACCTTAATGATATCATTATA

4 CTAACGTGCGTAGCAGCTGTCCAACTACGACTAAGCAAAAACCTTAATGATATCATTATA

3 CTAACGTGCGTAGCAGCTGTCCAACTACGACTAAGCAAAAACCTTAATGATATCATTATA

20 CTAACGTGCGTAGCAGCTGTCCAACTACGACTAAGCAAAAACCTTAATGATATCATTATA

****.**.**.*.**.*****************.********** *****.**.*** **

1RC AGTTTCATTAAGCCAAAAATTGAGCATACCTAATATTATCGGCCATTTATTGGAAGAGAC

16RC AGTTTCATTAAGCCAAAAATTGAGCATACCTAATATTATCGGCCATTTATTGGAAGAGAC

15RC AGTTTCATTAAGCCAAAAATTGAGCATACCTAATATTATCGGCCATTTATTGGAAGAGAC

2RC AGTTTCATTAAGCCAAAAATTGAGCATACCTAATATTATCGGCCATTTATTGGAAGAGAC

10 CGTTTTGTTAAGCCAAAATTTGAGCATACCTAATATTATCGGCCATTTATTGGAAGAGAC

17 CGTTTTGTTAAGCCAAAATTTGAGCATACCTAATATTATCGGCCATTTATTGGAAGAGAC

14 CGTTTTGTTAAGCCAAAATTTGAGCATACCTAATATTATCGGCCATTTATTGGAAGAGAC

13 CGTTTTGTTAAGCCAAAATTTGAGCATACCTAATATTATCGGCCATTTATTGGAAGAGAC

12 CGTTTTGTTAAGCCAAAATTTGAGCATACCTAATATTATCGGCCATTTATTGGAAGAGAC

8 CGTTTTGTTAAGCCAAAATTTGAGCATACCTAATATTATCGGCCATTTATTGGAAGAGAC

7 CGTTTTGTTAAGCCAAAATTTGAGCATACCTAATATTATCGGCCATTTATTGGAAGAGAC

19 CGTTTTGTTAAGCCAAAATTTGAGCATACCTAATATTATCGGCCATTTATTGGAAGAGAC

18 CGTTTTGTTAAGCCAAAATTTGAGCATACCTAATATTATCGGCCATTTATTGGAAGAGAC

22 CGTTTTGTTAAGCCAAAATTTGAGCATACCTAATATTATCGGCCATTTATTGGAAGAGAC

21 CGTTTTGTTAAGCCAAAATTTGAGCATACCTAATATTATCGGCCATTTATTGGAAGAGAC

9 CGTTTTGTTAAGCCAAAATTTGAGCATACCTAATATTATCGGCCATTTATTGGAAGAGAC

6 CGTTTTGTTAAGCCAAAATTTGAGCATACCTAATATTATCGGCCATTTATTGGAAGAGAC

5 CGTTTTGTTAAGCCAAAATTTGAGCATACCTAATATTATCGGCCATTTATTGGAAGAGAC

4 CGTTTTGTTAAGCCAAAATTTGAGCATACCTAATATTATCGGCCATTTATTGGAAGAGAC

3 CGTTTTGTTAAGCCAAAATTTGAGCATACCTAATATTATCGGCCATTTATTGGAAGAGAC

20 CGTTTTGTTAAGCCAAAATTTGAGCATACCTAATATTATCGGCCATTTATTGGAAGAGAC

****..*********** *****************************************

1RC TGAGGGAAAAAGCCACACTAAAGCGATATGCGACACAAGGCCAAAAGAGTGAGCAACGAA

16RC TGAGGGAAAAAGCCACACTAAAGCGATATGCGACACAAGGCCAAAAGAGTGAGCAACGAA

15RC TGAGGGAAAAAGCCACACTAAAGCGATATGCGACACAAGGCCAAAAGAGTGAGCAACGAA

2RC TGAGGGAAAAAGCCACACTAAAGCGATATGCGACACAAGGCCAAAAGAGTGAGCAACGAA

10 TGAGGGAAAAAGCCACACTAAAGCGATGTGCGACACAAGGCCGAAAGAGTGAGCAACGAA

17 TGAGGGAAAAAGCCACACTAAAGCGATGTGCGACACAAGGCCGAAAGAGTGAGCAACGAA

14 TGAGGGAAAAAGCCACACTAAAGCGATGTGCGACACAAGGCCGAAAGAGTGAGCAACGAA

13 TGAGGGAAAAAGCCACACTAAAGCGATGTGCGACACAAGGCCGAAAGAGTGAGCAACGAA

12 TGAGGGAAAAAGCCACACTAAAGCGATGTGCGACACAAGGCCGAAAGAGTGAGCAACGAA

8 TGAGGGAAAAAGCCACACTAAAGCGATGTGCGACACAAGGCCGAAAGAGTGAGCAACGAA

7 TGAGGGAAAAAGCCACACTAAAGCGATGTGCGACACAAGGCCGAAAGAGTGAGCAACGAA

19 TGAGGGAAAAAGCCACACTAAAGCGATGTGCGACACAAGGCCGAAAGAGTGAGCAACGAA

18 TGAGGGAAAAAGCCACACTAAAGCGATGTGCGACACAAGGCCGAAAGAGTGAGCAACGAA

22 TGAGGGAAAAAGCCACACTAAAGCGATGTGCGACACAAGGCCGAAAGAGTGAGCAACGAA

21 TGAGGGAAAAAGCCACACTAAAGCGATGTGCGACACAAGGCCGAAAGAGTGAGCAACGAA

9 TGAGGGAAAAAGCCACACTAAAGCGATGTGCGACACAAGGCCGAAAGAGTGAGCAACGAA

6 TGAGGGAAAAAGCCACACTAAAGCGATGTGCGACACAAGGCCGAAAGAGTGAGCAACGAA

5 TGAGGGAAAAAGCCACACTAAAGCGATGTGCGACACAAGGCCGAAAGAGTGAGCAACGAA

4 TGAGGGAAAAAGCCACACTAAAGCGATGTGCGACACAAGGCCGAAAGAGTGAGCAACGAA

3 TGAGGGAAAAAGCCACACTAAAGCGATGTGCGACACAAGGCCGAAAGAGTGAGCAACGAA

20 TGAGGGAAAAAGCCACACTAAAGCGATGTGCGACACAAGGCCGAAAGAGTGAGCAACGAA

***************************.**************.*****************

1RC GTAACTGTAGCCATTGTTTAATAAATAAGCTGTCGGTTTATTAGCTGAATGAATTGGTGT

16RC GTAACTGTAGCCATTGTTTAATAAATAAGCTGTCGGTTTATTAGCTGAATGAATTGGTGT

15RC GTAACTGTAGCCATTGTTTAATAAATAAGCTGTCGGTTTATTAGCTGAATGAATTGGTGT

2RC GTAACTGTAGCCATTGTTTAATAAATAAGCTGTCGGTTTATTAGCTGAATGAATTGGTGT

10 GTAACTGTAGCCATTGTTTAATAAATAAGCTGTCGGTTGATTAGCCGAACGAACTGTTGT

17 GTAACTGTAGCCATTGTTTAATAAATAAGCTGTCGGTTGATTAGCCGAACGAACTGTTGT

14 GTAACTGTAGCCATTGTTTAATAAATAAGCTGTCGGTTGATTAGCCGAACGAACTGTTGT

13 GTAACTGTAGCCATTGTTTAATAAATAAGCTGTCGGTTGATTAGCCGAACGAACTGTTGT

12 GTAACTGTAGCCATTGTTTAATAAATAAGCTGTCGGTTGATTAGCCGAACGAACTGTTGT

8 GTAACTGTAGCCATTGTTTAATAAATAAGCTGTCGGTTGATTAGCCGAACGAACTGTTGT

7 GTAACTGTAGCCATTGTTTAATAAATAAGCTGTCGGTTGATTAGCCGAACGAACTGTTGT

19 GTAACTGTAGCCATTGTTTAATAAATAAGCTGTCGGTTGATTAGCCGAACGAACTGTTGT

18 GTAACTGTAGCCATTGTTTAATAAATAAGCTGTCGGTTGATTAGCCGAACGAACTGTTGT

22 GTAACTGTAGCCATTGTTTAATAAATAAGCTGTCGGTTGATTAGCCGAACGAACTGTTGT

21 GTAACTGTAGCCATTGTTTAATAAATAAGCTGTCGGTTGATTAGCCGAACGAACTGTTGT

9 GTAACTGTAGCCATTGTTTAATAAATAAGCTGTCGGTTGATTAGCCGAACGAACTGTTGT

6 GTAACTGTAGCCATTGTTTAATAAATAAGCTGTCGGTTGATTAGCCGAACGAACTGTTGT

5 GTAACTGTAGCCATTGTTTAATAAATAAGCTGTCGGTTGATTAGCCGAACGAACTGTTGT

4 GTAACTGTAGCCATTGTTTAATAAATAAGCTGTCGGTTGATTAGCCGAACGAACTGTTGT

3 GTAACTGTAGCCATTGTTTAATAAATAAGCTGTCGGTTGATTAGCCGAACGAACTGTTGT

20 GTAACTGTAGCCATTGTTTAATAAATAAGCTGTCGGTTGATTAGCCGAACGAACTGTTGT

************************************** ******.***.***.** ***

1RC ACGCATTATAACTAACAATCAACTGATGTTTTTGTTGTATTCCAACCTGCTGTGCTGAGG

16RC ACGCATTATAACTAACAATCAACTGATGTTTTTGTTGTATTCCAACCTGCTGTGCTGAGG

15RC ACGCATTATAACTAACAATCAACTGATGTTTTTGTTGTATTCCAACCTGCTGTGCTGAGG

2RC ACGCATTATAACTAACAATCAACTGATGTTTTTGTTGTATTCCAACCTGCTGTGCTGAGG

10 ACGCATTATAACTAACAATCGACTGATGTTTTTGTTGTATTCCAGCCTGCTGTACTGAGG

17 ACGCATTATAACTAACAATCGACTGATGTTTTTGTTGTATTCCAGCCTGCTGTACTGAGG

14 ACGCATTATAACTAACAATCGACTGATGTTTTTGTTGTATTCCAGCCTGCTGTACTGAGG

13 ACGCATTATAACTAACAATCGACTGATGTTTTTGTTGTATTCCAGCCTGCTGTACTGAGG

12 ACGCATTATAACTAACAATCGACTGATGTTTTTGTTGTATTCCAGCCTGCTGTACTGAGG

8 ACGCATTATAACTAACAATCGACTGATGTTTTTGTTGTATTCCAGCCTGCTGTACTGAGG

7 ACGCATTATAACTAACAATCGACTGATGTTTTTGTTGTATTCCAGCCTGCTGTACTGAGG

19 ACGCATTATAACTAACAATCGACTGATGTTTTTGTTGTATTCCAGCCTGCTGTACTGAGG

18 ACGCATTATAACTAACAATCGACTGATGTTTTTGTTGTATTCCAGCCTGCTGTACTGAGG

22 ACGCATTATAACTAACAATCGACTGATGTTTTTGTTGTATTCCAGCCTGCTGTACTGAGG

21 ACGCATTATAACTAACAATCGACTGATGTTTTTGTTGTATTCCAGCCTGCTGTACTGAGG

9 ACGCATTATAACTAACAATCGACTGATGTTTTTGTTGTATTCCAGCCTGCTGTACTGAGG

6 ACGCATTATAACTAACAATCGACTGATGTTTTTGTTGTATTCCAGCCTGCTGTACTGAGG

5 ACGCATTATAACTAACAATCGACTGATGTTTTTGTTGTATTCCAGCCTGCTGTACTGAGG

4 ACGCATTATAACTAACAATCGACTGATGTTTTTGTTGTATTCCAGCCTGCTGTACTGAGG

3 ACGCATTATAACTAACAATCGACTGATGTTTTTGTTGTATTCCAGCCTGCTGTACTGAGG

20 ACGCATTATAACTAACAATCGACTGATGTTTTTGTTGTATTCCAGCCTGCTGTACTGAGG

********************.***********************.********.******

1RC AATTCCCAGAGATGAAACCAACAAAGTACAACAATATAGAGAGAAATTTGCGCTGATCCT

16RC AATTCCCAGAGATGAAACCAACAAAGTACAACAATATAGAGAGAAATTTGCGCTGATCCT

15RC AATTCCCAGAGATGAAACCAACAAAGTACAACAATATAGAGAGAAATTTGCGCTGATCCT

2RC AATTCCCAGAGATGAAACCAACAAAGTACAACAATATAGAGAGAAATTTGCGCTGATCCT

10 AATTCCCAGAGATGAAACCAACAAAGTACAACAATATAGAGAGAAGTTTGCGCTGATCCT

17 AATTCCCAGAGATGAAACCAACAAAGTACAACAATATAGAGAGAAGTTTGCGCTGATCCT

14 AATTCCCAGAGATGAAACCAACAAAGTACAACAATATAGAGAGAAGTTTGCGCTGATCCT

13 AATTCCCAGAGATGAAACCAACAAAGTACAACAATATAGAGAGAAGTTTGCGCTGATCCT

12 AATTCCCAGAGATGAAACCAACAAAGTACAACAATATAGAGAGAAGTTTGCGCTGATCCT

8 AATTCCCAGAGATGAAACCAACAAAGTACAACAATATAGAGAGAAGTTTGCGCTGATCCT

7 AATTCCCAGAGATGAAACCAACAAAGTACAACAATATAGAGAGAAGTTTGCGCTGATCCT

19 AATTCCCAGAGATGAAACCAACAAAGTACAACAATATAGAGAGAAGTTTGCGCTGATCCT

18 AATTCCCAGAGATGAAACCAACAAAGTACAACAATATAGAGAGAAGTTTGCGCTGATCCT

22 AATTCCCAGAGATGAAACCAACAAAGTACAACAATATAGAGAGAAGTTTGCGCTGATCCT

21 AATTCCCAGAGATGAAACCAACAAAGTACAACAATATAGAGAGAAGTTTGCGCTGATCCT

9 AATTCCCAGAGATGAAACCAACAAAGTACAACAATATAGAGAGAAGTTTGCGCTGATCCT

6 AATTCCCAGAGATGAAACCAACAAAGTACAACAATATAGAGAGAAGTTTGCGCTGATCCT

5 AATTCCCAGAGATGAAACCAACAAAGTACAACAATATAGAGAGAAGTTTGCGCTGATCCT

4 AATTCCCAGAGATGAAACCAACAAAGTACAACAATATAGAGAGAAGTTTGCGCTGATCCT

3 AATTCCCAGAGATGAAACCAACAAAGTACAACAATATAGAGAGAAGTTTGCGCTGATCCT

20 AATTCCCAGAGATGAAACCAACAAAGTACAACAATATAGAGAGAAGTTTGCGCTGATCCT

*********************************************.**************

1RC CTGAAATAGCCAATAAATATACCAAGATGAACGTCTATAGCTGATATACCGGGAACCAAA

16RC CTGAAATAGCCAATAAATATACCAAGATGAACGTCTATAGCTGATATACCGGGAACCAAA

15RC CTGAAATAGCCAATAAATATACCAAGATGAACGTCTATAGCTGATATACCGGGAACCAAA

2RC CTGAAATAGCCAATAAATATACCAAGATGAACGTCTATAGCTGATATACCGGGAACCAAA

10 CTGAAATAACCAATAAATATACCAAGATGAACGTTTATAGCTGATATACCGGGAACCAAA

17 CTGAAATAACCAATAAATATACCAAGATGAACGTTTATAGCTGATATACCGGGAACCAAA

14 CTGAAATAACCAATAAATATACCAAGATGAACGTTTATAGCTGATATACCGGGAACCAAA

13 CTGAAATAACCAATAAATATACCAAGATGAACGTTTATAGCTGATATACCGGGAACCAAA

12 CTGAAATAACCAATAAATATACCAAGATGAACGTTTATAGCTGATATACCGGGAACCAAA

8 CTGAAATAACCAATAAATATACCAAGATGAACGTTTATAGCTGATATACCGGGAACCAAA

7 CTGAAATAACCAATAAATATACCAAGATGAACGTTTATAGCTGATATACCGGGAACCAAA

19 CTGAAATAACCAATAAATATACCAAGATGAACGTTTATAGCTGATATACCGGGAACCAAA

18 CTGAAATAACCAATAAATATACCAAGATGAACGTTTATAGCTGATATACCGGGAACCAAA

22 CTGAAATAACCAATAAATATACCAAGATGAACGTTTATAGCTGATATACCGGGAACCAAA

21 CTGAAATAACCAATAAATATACCAAGATGAACGTTTATAGCTGATATACCGGGAACCAAA

9 CTGAAATAACCAATAAATATACCAAGATGAACGTTTATAGCTGATATACCGGGAACCAAA

6 CTGAAATAACCAATAAATATACCAAGATGAACGTTTATAGCTGATATACCGGGAACCAAA

5 CTGAAATAACCAATAAATATACCAAGATGAACGTTTATAGCTGATATACCGGGAACCAAA

4 CTGAAATAACCAATAAATATACCAAGATGAACGTTTATAGCTGATATACCGGGAACCAAA

3 CTGAAATAACCAATAAATATACCAAGATGAACGTTTATAGCTGATATACCGGGAACCAAA

20 CTGAAATAACCAATAAATATACCAAGATGAACGTTTATAGCTGATATACCGGGAACCAAA

********.*************************.*************************

1RC TTCGAACCGAAGAAAGCGCCTAAAGTAAAAGCGATAAGAGAAATAGCGAAAGAAGTGTTT

16RC TTCGAACCGAAGAAAGCGCCTAAAGTAAAAGCGATAAGAGAAATAGCGAAAGAAGTGTTT

15RC TTCGAACCGAAGAAAGCGCCTAAAGTAAAAGCGATAAGAGAAATAGCGAAAGAAGTGTTT

2RC TTCGAACCGAAGAAAGCGCCTAAAGTAAAAGCGATAAGAGAAATAGCGAAAGAAGTGTTT

10 TTCGAACCGAAGAAAGCGCCTAAAGTAAAAGCGATAAGAGAAATATTGAAAGCAGTGTTT

17 TTCGAACCGAAGAAAGCGCCTAAAGTAAAAGCGATAAGAGAAATATTGAAAGCAGTGTTT

14 TTCGAACCGAAGAAAGCGCCTAAAGTAAAAGCGATAAGAGAAATATTGAAAGCAGTGTTT

13 TTCGAACCGAAGAAAGCGCCTAAAGTAAAAGCGATAAGAGAAATATTGAAAGCAGTGTTT

12 TTCGAACCGAAGAAAGCGCCTAAAGTAAAAGCGATAAGAGAAATATTGAAAGCAGTGTTT

8 TTCGAACCGAAGAAAGCGCCTAAAGTAAAAGCGATAAGAGAAATATTGAAAGCAGTGTTT

7 TTCGAACCGAAGAAAGCGCCTAAAGTAAAAGCGATAAGAGAAATATTGAAAGCAGTGTTT

19 TTCGAACCGAAGAAAGCGCCTAAAGTAAAAGCGATAAGAGAAATATTGAAAGCAGTGTTT

18 TTCGAACCGAAGAAAGCGCCTAAAGTAAAAGCGATAAGAGAAATATTGAAAGCAGTGTTT

22 TTCGAACCGAAGAAAGCGCCTAAAGTAAAAGCGATAAGAGAAATATTGAAAGCAGTGTTT

21 TTCGAACCGAAGAAAGCGCCTAAAGTAAAAGCGATAAGAGAAATATTGAAAGCAGTGTTT

9 TTCGAACCGAAGAAAGCGCCTAAAGTAAAAGCGATAAGAGAAATATTGAAAGCAGTGTTT

6 TTCGAACCGAAGAAAGCGCCTAAAGTAAAAGCGATAAGAGAAATATTGAAAGCAGTGTTT

5 TTCGAACCGAAGAAAGCGCCTAAAGTAAAAGCGATAAGAGAAATATTGAAAGCAGTGTTT

4 TTCGAACCGAAGAAAGCGCCTAAAGTAAAAGCGATAAGAGAAATATTGAAAGCAGTGTTT

3 TTCGAACCGAAGAAAGCGCCTAAAGTAAAAGCGATAAGAGAAATATTGAAAGCAGTGTTT

20 TTCGAACCGAAGAAAGCGCCTAAAGTAAAAGCGATAAGAGAAATATTGAAAGCAGTGTTT

********************************************* .***** *******

1RC GGGAGACTGCCGTTAAGTCTTGGAGGACAATCAATCTGATTGGCGTATAGCGCAGATCTG

16RC GGGAGACTGCCGTTAAGTCTTGGAGGACAATCAATCTGATTGGCGTATAGCGCAGATCTG

15RC GGGAGACTGCCGTTAAGTCTTGGAGGACAATCAATCTGATTGGCGTATAGCGCAGATCTG

2RC GGGAGACTGCCGTTAAGTCTTGGAGGACAATCAATCTGATTGGCGTATAGCGCAGATCTG

10 GGGAGACTGCCGTTAAGTCTTGGAGGGTAGTCAATCTGATTGGCGTATAACGCAGATCTG

17 GGGAGACTGCCGTTAAGTCTTGGAGGGTAGTCAATCTGATTGGCGTATAACGCAGATCTG

14 GGGAGACTGCCGTTAAGTCTTGGAGGGTAGTCAATCTGATTGGCGTATAACGCAGATCTG

13 GGGAGACTGCCGTTAAGTCTTGGAGGGTAGTCAATCTGATTGGCGTATAACGCAGATCTG

12 GGGAGACTGCCGTTAAGTCTTGGAGGGTAGTCAATCTGATTGGCGTATAACGCAGATCTG

8 GGGAGACTGCCGTTAAGTCTTGGAGGGTAGTCAATCTGATTGGCGTATAACGCAGATCTG

7 GGGAGACTGCCGTTAAGTCTTGGAGGGTAGTCAATCTGATTGGCGTATAACGCAGATCTG

19 GGGAGACTGCCGTTAAGTCTTGGAGGGTAGTCAATCTGATTGGCGTATAACGCAGATCTG

18 GGGAGACTGCCGTTAAGTCTTGGAGGGTAGTCAATCTGATTGGCGTATAACGCAGATCTG

22 GGGAGACTGCCGTTAAGTCTTGGAGGGTAGTCAATCTGATTGGCGTATAACGCAGATCTG

21 GGGAGACTGCCGTTAAGTCTTGGAGGGTAGTCAATCTGATTGGCGTATAACGCAGATCTG

9 GGGAGACTGCCGTTAAGTCTTGGAGGGTAGTCAATCTGATTGGCGTATAACGCAGATCTG

6 GGGAGACTGCCGTTAAGTCTTGGAGGGTAGTCAATCTGATTGGCGTATAACGCAGATCTG

5 GGGAGACTGCCGTTAAGTCTTGGAGGGTAGTCAATCTGATTGGCGTATAACGCAGATCTG

4 GGGAGACTGCCGTTAAGTCTTGGAGGGTAGTCAATCTGATTGGCGTATAACGCAGATCTG

3 GGGAGACTGCCGTTAAGTCTTGGAGGGTAGTCAATCTGATTGGCGTATAACGCAGATCTG

20 GGGAGACTGCCGTTAAGTCTTGGAGGGTAGTCAATCTGATTGGCGTATAACGCAGATCTG

**************************..*.*******************.**********

1RC GGTATCTTAGTAGACGACATGAGGACAATGGAGTAAGTGTTGAAAAGGACAGCTGAGAAA

16RC GGTATCTTAGTAGACGACATGAGGACAATGGAGTAAGTGTTGAAAAGGACAGCTGAGAAA

15RC GGTATCTTAGTAGACGACATGAGGACAATGGAGTAAGTGTTGAAAAGGACAGCTGAGAAA

2RC GGTATCTTAGTAGACGACATGAGGACAATGGAGTAAGTGTTGAAAAGGACAGCTGAGAAA

10 GGTATCTTAGTAGACGACATGAGGACAATGGAGTAAGTGTTGAAAA-GACAGTTGAGAAA

17 GGTATCTTAGTAGACGACATGAGGACAATGGAGTAAGTGTTGAAAA-GACAGTTGAGAAA

14 GGTATCTTAGTAGACGACATGAGGACAATGGAGTAAGTGTTGAAAA-GACAGTTGAGAAA

13 GGTATCTTAGTAGACGACATGAGGACAATGGAGTAAGTGTTGAAAA-GACAGTTGAGAAA

12 GGTATCTTAGTAGACGACATGAGGACAATGGAGTAAGTGTTGAAAA-GACAGTTGAGAAA

8 GGTATCTTAGTAGACGACATGAGGACAATGGAGTAAGTGTTGAAAA-GACAGTTGAGAAA

7 GGTATCTTAGTAGACGACATGAGGACAATGGAGTAAGTGTTGAAAA-GACAGTTGAGAAA

19 GGTATCTTAGTAGACGACATGAGGACAATGGAGTAAGTGTTGAAAA-GACAGTTGAGAAA

18 GGTATCTTAGTAGACGACATGAGGACAATGGAGTAAGTGTTGAAAA-GACAGTTGAGAAA

22 GGTATCTTAGTAGACGACATGAGGACAATGGAGTAAGTGTTGAAAA-GACAGTTGAGAAA

21 GGTATCTTAGTAGACGACATGAGGACAATGGAGTAAGTGTTGAAAA-GACAGTTGAGAAA

9 GGTATCTTAGTAGACGACATGAGGACAATGGAGTAAGTGTTGAAAA-GACAGTTGAGAAA

6 GGTATCTTAGTAGACGACATGAGGACAATGGAGTAAGTGTTGAAAA-GACAGTTGAGAAA

5 GGTATCTTAGTAGACGACATGAGGACAATGGAGTAAGTGTTGAAAA-GACAGTTGAGAAA

4 GGTATCTTAGTAGACGACATGAGGACAATGGAGTAAGTGTTGAAAA-GACAGTTGAGAAA

3 GGTATCTTAGTAGACGACATGAGGACAATGGAGTAAGTGTTGAAAA-GACAGTTGAGAAA

20 GGTATCTTAGTAGACGACATGAGGACAATGGAGTAAGTGTTGAAAA-GACAGTTGAGAAA

********************************************** *****.*******

1RC ATAACAGAAAATATAACAAAAGCTTTCAGGCTTTTAAATTATCAACAGCGATGTCTTTCT

16RC ATAACAGAAAATATAACAAAAGCTTTCAGGCTTTTAAATTATCAACAGCGATGTCTTTCT

15RC ATAACAGAAAATATAACAAAAGCTTTCAGGCTTTTAAATTATCAACAGCGATGTCTTTCT

2RC ATAACAGAAAATATAACAAAAGCTTTCAGGCTTTTAAATTATCAACAGCGATGTCTTTCT

10 ATAACATAAAATACAACAAAAGCTTTCAGGCTTTTAAATTATCAACAGCGATGTCTTTCT

17 ATAACATAAAATACAACAAAAGCTTTCAGGCTTTTAAATTATCAACAGCGATGTCTTTCT

14 ATAACATAAAATACAACAAAAGCTTTCAGGCTTTTAAATTATCAACAGCGATGTCTTTCT

13 ATAACATAAAATACAACAAAAGCTTTCAGGCTTTTAAATTATCAACAGCGATGTCTTTCT

12 ATAACATAAAATACAACAAAAGCTTTCAGGCTTTTAAATTATCAACAGCGATGTCTTTCT

8 ATAACATAAAATACAACAAAAGCTTTCAGGCTTTTAAATTATCAACAGCGATGTCTTTCT

7 ATAACATAAAATACAACAAAAGCTTTCAGGCTTTTAAATTATCAACAGCGATGTCTTTCT

19 ATAACATAAAATACAACAAAAGCTTTCAGGCTTTTAAATTATCAACAGCGATGTCTTTCT

18 ATAACATAAAATACAACAAAAGCTTTCAGGCTTTTAAATTATCAACAGCGATGTCTTTCT

22 ATAACATAAAATACAACAAAAGCTTTCAGGCTTTTAAATTATCAACAGCGATGTCTTTCT

21 ATAACATAAAATACAACAAAAGCTTTCAGGCTTTTAAATTATCAACAGCGATGTCTTTCT

9 ATAACATAAAATACAACAAAAGCTTTCAGGCTTTTAAATTATCAACAGCGATGTCTTTCT

6 ATAACATAAAATACAACAAAAGCTTTCAGGCTTTTAAATTATCAACAGCGATGTCTTTCT

5 ATAACATAAAATACAACAAAAGCTTTCAGGCTTTTAAATTATCAACAGCGATGTCTTTCT

4 ATAACATAAAATACAACAAAAGCTTTCAGGCTTTTAAATTATCAACAGCGATGTCTTTCT

3 ATAACATAAAATACAACAAAAGCTTTCAGGCTTTTAAATTATCAACAGCGATGTCTTTCT

20 ATAACATAAAATACAACAAAAGCTTTCAGGCTTTTAAATTATCAACAGCGATGTCTTTCT

****** ******.**********************************************

1RC GACCAAAACCATCATCGTTAGCAAGTCCACTGATGACTACTATTCCCTCAAGACTCTCTC

16RC GACCAAAACCATCATCGTTAGCAAGTCCACTGATGACTACTATTCCCTCAAGACTCTCTC

15RC GACCAAAACCATCATCGTTAGCAAGTCCACTGATGACTACTATTCCCTCAAGACTCTCTC

2RC GACCAAAACCATCATCGTTAGCAAGTCCACTGATGACTACTATTCCCTCAAGACTCTCTC

10 GACCAAAACCATCATCTTTAGCAAGTCCACTGATGACTACTATTCCCTCAAGACTCTCTC

17 GACCAAAACCATCATCTTTAGCAAGTCCACTGATGACTACTATTCCCTCAAGACTCTCTC

14 GACCAAAACCATCATCTTTAGCAAGTCCACTGATGACTACTATTCCCTCAAGACTCTCTC

13 GACCAAAACCATCATCTTTAGCAAGTCCACTGATGACTACTATTCCCTCAAGACTCTCTC

12 GACCAAAACCATCATCTTTAGCAAGTCCACTGATGACTACTATTCCCTCAAGACTCTCTC

8 GACCAAAACCATCATCTTTAGCAAGTCCACTGATGACTACTATTCCCTCAAGACTCTCTC

7 GACCAAAACCATCATCTTTAGCAAGTCCACTGATGACTACTATTCCCTCAAGACTCTCTC

19 GACCAAAACCATCATCTTTAGCAAGTCCACTGATGACTACTATTCCCTCAAGACTCTCTC

18 GACCAAAACCATCATCTTTAGCAAGTCCACTGATGACTACTATTCCCTCAAGACTCTCTC

22 GACCAAAACCATCATCTTTAGCAAGTCCACTGATGACTACTATTCCCTCAAGACTCTCTC

21 GACCAAAACCATCATCTTTAGCAAGTCCACTGATGACTACTATTCCCTCAAGACTCTCTC

9 GACCAAAACCATCATCTTTAGCAAGTCCACTGATGACTACTATTCCCTCAAGACTCTCTC

6 GACCAAAACCATCATCTTTAGCAAGTCCACTGATGACTACTATTCCCTCAAGACTCTCTC

5 GACCAAAACCATCATCTTTAGCAAGTCCACTGATGACTACTATTCCCTCAAGACTCTCTC

4 GACCAAAACCATCATCTTTAGCAAGTCCACTGATGACTACTATTCCCTCAAGACTCTCTC

3 GACCAAAACCATCATCTTTAGCAAGTCCACTGATGACTACTATTCCCTCAAGACTCTCTC

20 GACCAAAACCATCATCTTTAGCAAGTCCACTGATGACTACTATTCCCTCAAGACTCTCTC

**************** *******************************************

1RC AAGTTGCAAGATCATCAAACTCATTAGCTGAAGCTCATGCAAGAGCTAGAACGCTTTATC

16RC AAGTTGCAAGATCATCAAACTCATTAGCTGAAGCTCATGCAAGAGCTAGAACGCTTTATC

15RC AAGTTGCAAGATCATCAAACTCATTAGCTGAAGCTCATGCAAGAGCTAGAACGCTTTATC

2RC AAGTTGCAAGATCATCAAACTCATTAGCTGAAGCTCATGCAAGAGCTAGAACGCTTTATC

10 AAGTTGCAAGATCATCAAACTCATTAGCTGAAGCTCATGCAAGAGCTAGAACGCTTTATC

17 AAGTTGCAAGATCATCAAACTCATTAGCTGAAGCTCATGCAAGAGCTAGAACGCTTTATC

14 AAGTTGCAAGATCATCAAACTCATTAGCTGAAGCTCATGCAAGAGCTAGAACGCTTTATC

13 AAGTTGCAAGATCATCAAACTCATTAGCTGAAGCTCATGCAAGAGCTAGAACGCTTTATC

12 AAGTTGCAAGATCATCAAACTCATTAGCTGAAGCTCATGCAAGAGCTAGAACGCTTTATC

8 AAGTTGCAAGATCATCAAACTCATTAGCTGAAGCTCATGCAAGAGCTAGAACGCTTTATC

7 AAGTTGCAAGATCATCAAACTCATTAGCTGAAGCTCATGCAAGAGCTAGAACGCTTTATC

19 AAGTTGCAAGATCATCAAACTCATTAGCTGAAGCTCATGCAAGAGCTAGAACGCTTTATC

18 AAGTTGCAAGATCATCAAACTCATTAGCTGAAGCTCATGCAAGAGCTAGAACGCTTTATC

22 AAGTTGCAAGATCATCAAACTCATTAGCTGAAGCTCATGCAAGAGCTAGAACGCTTTATC

21 AAGTTGCAAGATCATCAAACTCATTAGCTGAAGCTCATGCAAGAGCTAGAACGCTTTATC

9 AAGTTGCAAGATCATCAAACTCATTAGCTGAAGCTCATGCAAGAGCTAGAACGCTTTATC

6 AAGTTGCAAGATCATCAAACTCATTAGCTGAAGCTCATGCAAGAGCTAGAACGCTTTATC

5 AAGTTGCAAGATCATCAAACTCATTAGCTGAAGCTCATGCAAGAGCTAGAACGCTTTATC

4 AAGTTGCAAGATCATCAAACTCATTAGCTGAAGCTCATGCAAGAGCTAGAACGCTTTATC

3 AAGTTGCAAGATCATCAAACTCATTAGCTGAAGCTCATGCAAGAGCTAGAACGCTTTATC

20 AAGTTGCAAGATCATCAAACTCATTAGCTGAAGCTCATGCAAGAGCTAGAACGCTTTATC

************************************************************

1RC GTGACTTCTATCGTAGCGTGAGTACCTCAAACGACGAGTAGGCCGATGAGGCTCAACGGA

16RC GTGACTTCTATCGCAGCGTGAGTACCTCAAACGACGAGTAGGCCGATGAGGCTCAACGGA

15RC GTGACTTCTATCGCAGCGTGAGTACCTCAAACGACGAGTAGGCCGACGAGGCTCAACGGA

2RC GTGACTTCTATCGCAGCGTGAGTACCTCAAACGACGAGTAGGCCGACGAGGCTCAACGGA

10 GTGACTTCTATCGCAGCGTGAGTACCTCAAACGACGAGTAGGCCGATGAGGCTCAACGGA

17 GTGACTTCTATCGCAGCGTGAGTACCTCAAACGACGAGTAGGCCGATGAGGCTCAACGGA

14 GTGACTTCTATCGCAGCGTGAGTACCTCAAACGACGAGTAGGCCGATGAGGCTCAACGGA

13 GTGACTTCTATCGCAGCGTGAGTACCTCAAACGACGAGTAGGCCGATGAGGCTCAACGGA

12 GTGACTTCTATCGCAGCGTGAGTACCTCAAACGACGAGTAGGCCGATGAGGCTCAACGGA

8 GTGACTTCTATCGCAGCGTGAGTACCTCAAACGACGAGTAGGCCGATGAGGCTCAACGGA

7 GTGACTTCTATCGCAGCGTGAGTACCTCAAACGACGAGTAGGCCGATGAGGCTCAACGGA

19 GTGACTTCTATCGCAGCGTGAGTACCTCAAACGACGAGTAGGCCGATGAGGCTCAACGGA

18 GTGACTTCTATCGCAGCGTGAGTACCTCAAACGACGAGTAGGCCGATGAGGCTCAACGGA

22 GTGACTTCTATCGCAGCGTGAGTACCTCAAACGACGAGTAGGCCGATGAGGCTCAACGGA

21 GTGACTTCTATCGCAGCGTGAGTACCTCAAACGACGAGTAGGCCGATGAGGCTCAACGGA

9 GTGACTTCTATCGCAGCGTGAGTACCTCAAACGACGAGTAGGCCGATGAGGCTCAACGGA

6 GTGACTTCTATCGCAGCGTGAGTACCTCAAACGACGAGTAGGCCGATGAGGCTCAACGGA

5 GTGACTTCTATCGCAGCGTGAGTACCTCAAACGACGAGTAGGCCGATGAGGCTCAACGGA

4 GTGACTTCTATCGCAGCGTGAGTACCTCAAACGACGAGTAGGCCGATGAGGCTCAACGGA

3 GTGACTTCTATCGCAGCGTGAGTACCTCAAACGACGAGTAGGCCGATGAGGCTCAACGGA

20 GTGACTTCTATCGCAGCGTGAGTACCTCAAACGACGAGTAGGCCGATGAGGCTCAACGGA

*************.********************************.*************

1RC TTGAATAGGCTCCAAGCATCTGTGCATTATACTCACTTAACATTCCACCTTCACAAATAA

16RC TTGAATAGGCTCCAAGCATCTGTGCATTATACTCACTTAACATTCCACCTTCACAAATAA

15RC TTGAATAGGCTCCAAGCATCTGTGCATTATACTCACTTAACATTCCACCTTCACAAATAA

2RC TTGAATAGGCTCCAAGCATCTGTGCATTATACTCACTTAACATTCCACCTTCACAAATAA

10 TTGAATAGGCTCCAAGCATCTGTGCATTATACTCACTTAACATTCCACCTTCACAAATAA

17 TTGAATAGGCTCCAAGCATCTGTGCATTATACTCACTTAACATTCCACCTTCACAAATAA

14 TTGAATAGGCTCCAAGCATCTGTGCATTATACTCACTTAACATTCCACCTTCACAAATAA

13 TTGAATAGGCTCCAAGCATCTGTGCATTATACTCACTTAACATTCCACCTTCACAAATAA

12 TTGAATAGGCTCCAAGCATCTGTGCATTATACTCACTTAACATTCCACCTTCACAAATAA

8 TTGAATAGGCTCCAAGCATCTGTGCATTATACTCACTTAACATTCCACCTTCACAAATAA

7 TTGAATAGGCTCCAAGCATCTGTGCATTATACTCACTTAACATTCCACCTTCACAAATAA

19 TTGAATAGGCTCCAAGCATCTGTGCATTATACTCACTTAACATTCCACCTTCACAAATAA

18 TTGAATAGGCTCCAAGCATCTGTGCATTATACTCACTTAACATTCCACCTTCACAAATAA

22 TTGAATAGGCTCCAAGCATCTGTGCATTATACTCACTTAACATTCCACCTTCACAAATAA

21 TTGAATAGGCTCCAAGCATCTGTGCATTATACTCACTTAACATTCCACCTTCACAAATAA

9 TTGAATAGGCTCCAAGCATCTGTGCATTATACTCACTTAACATTCCACCTTCACAAATAA

6 TTGAATAGGCTCCAAGCATCTGTGCATTATACTCACTTAACATTCCACCTTCACAAATAA

5 TTGAATAGGCTCCAAGCATCTGTGCATTATACTCACTTAACATTCCACCTTCACAAATAA

4 TTGAATAGGCTCCAAGCATCTGTGCATTATACTCACTTAACATTCCACCTTCACAAATAA

3 TTGAATAGGCTCCAAGCATCTGTGCATTATACTCACTTAACATTCCACCTTCACAAATAA

20 TTGAATAGGCTCCAAGCATCTGTGCATTATACTCACTTAACATTCCACCTTCACAAATAA

************************************************************

1RC GAGCAAAGATTCGTCAGGAATTTGAGAAGAACAGAAATTTGGATGATTTGAATGTTATTG

16RC GAGCAAAGATTCGTCAGGAATTTGAGAAGAACAGAAATTTGGATGATTTGAATGTTATTG

15RC GAGCAAAGATTCGTCAGGAATTTGAGAAGAACAGAAATTTGGATGATTTGAATGTTATTG

2RC GAGCAAAGATTCGTCAGGAATTTGAGAAGAACAGAAATTTGGATGATTTGAATGTTATTG

10 GAGCAAAGATTCGTCAGGAATTTGAGAAGAACAGAAATTTGGATGATTTGAATGTTATTG

17 GAGCAAAGATTCGTCAGGAATTTGAGAAGAACAGAAATTTGGATGATTTGAATGTTATTG

14 GAGCAAAGATTCGTCAGGAATTTGAGAAGAACAGAAATTTGGATGATTTGAATGTTATTG

13 GAGCAAAGATTCGTCAGGAATTTGAGAAGAACAGAAATTTGGATGATTTGAATGTTATTG

12 GAGCAAAGATTCGTCAGGAATTTGAGAAGAACAGAAATTTGGATGATTTGAATGTTATTG

8 GAGCAAAGATTCGTCAGGAATTTGAGAAGAACAGAAATTTGGATGATTTGAATGTTATTG

7 GAGCAAAGATTCGTCAGGAATTTGAGAAGAACAGAAATTTGGATGATTTGAATGTTATTG

19 GAGCAAAGATTCGTCAGGAATTTGAGAAGAACAGAAATTTGGATGATTTGAATGTTATTG

18 GAGCAAAGATTCGTCAGGAATTTGAGAAGAACAGAAATTTGGATGATTTGAATGTTATTG

22 GAGCAAAGATTCGTCAGGAATTTGAGAAGAACAGAAATTTGGATGATTTGAATGTTATTG

21 GAGCAAAGATTCGTCAGGAATTTGAGAAGAACAGAAATTTGGATGATTTGAATGTTATTG

9 GAGCAAAGATTCGTCAGGAATTTGAGAAGAACAGAAATTTGGATGATTTGAATGTTATTG

6 GAGCAAAGATTCGTCAGGAATTTGAGAAGAACAGAAATTTGGATGATTTGAATGTTATTG

5 GAGCAAAGATTCGTCAGGAATTTGAGAAGAACAGAAATTTGGATGATTTGAATGTTATTG

4 GAGCAAAGATTCGTCAGGAATTTGAGAAGAACAGAAATTTGGATGATTTGAATGTTATTG

3 GAGCAAAGATTCGTCAGGAATTTGAGAAGAACAGAAATTTGGATGATTTGAATGTTATTG

20 GAGCAAAGATTCGTCAGGAATTTGAGAAGAACAGAAATTTGGATGATTTGAATGTTATTG

************************************************************

1RC ACTTAGTCTTATTCAAAGGCAGGCAGGAGTATCAGGAGACGATGAATGCATGGAAGCAAG

16RC ACTTAGTCTTATTCAAAGGCAGGCAGGAGTATCAGGAGACGATGAATGCATGGAAGCAAG

15RC ACTTAGTCTTATTCAAAGGCAGGCAGGAGTATCAGGAGACGATGAATGCATGGAAGCAAG

2RC ACTTAGTCTTATTCAAAGGCAGGCAGGAGTATCAGGAGACGATGAATGCATGGAAGCAAG

10 ACTTAGTCTTATTCAAAGGCAGGCAAGAGTATCAGGAGACGATGAATGCATGGAAGCAAG

17 ACTTAGTCTTATTCAAAGGCAGGCAAGAGTATCAGGAGACGATGAATGCATGGAAGCAAG

14 ACTTAGTCTTATTCAAAGGCAGGCAAGAGTATCAGGAGACGATGAATGCATGGAAGCAAG

13 ACTTAGTCTTATTCAAAGGCAGGCAAGAGTATCAGGAGACGATGAATGCATGGAAGCAAG

12 ACTTAGTCTTATTCAAAGGCAGGCAAGAGTATCAGGAGACGATGAATGCATGGAAGCAAG

8 ACTTAGTCTTATTCAAAGGCAGGCAAGAGTATCAGGAGACGATGAATGCATGGAAGCAAG

7 ACTTAGTCTTATTCAAAGGCAGGCAAGAGTATCAGGAGACGATGAATGCATGGAAGCAAG

19 ACTTAGTCTTATTCAAAGGCAGGCAAGAGTATCAGGAGACGATGAATGCATGGAAGCAAG

18 ACTTAGTCTTATTCAAAGGCAGGCAAGAGTATCAGGAGACGATGAATGCATGGAAGCAAG

22 ACTTAGTCTTATTCAAAGGCAGGCAAGAGTATCAGGAGACGATGAATGCATGGAAGCAAG

21 ACTTAGTCTTATTCAAAGGCAGGCAAGAGTATCAGGAGACGATGAATGCATGGAAGCAAG

9 ACTTAGTCTTATTCAAAGGCAGGCAAGAGTATCAGGAGACGATGAATGCATGGAAGCAAG

6 ACTTAGTCTTATTCAAAGGCAGGCAAGAGTATCAGGAGACGATGAATGCATGGAAGCAAG

5 ACTTAGTCTTATTCAAAGGCAGGCAAGAGTATCAGGAGACGATGAATGCATGGAAGCAAG

4 ACTTAGTCTTATTCAAAGGCAGGCAAGAGTATCAGGAGACGATGAATGCATGGAAGCAAG

3 ACTTAGTCTTATTCAAAGGCAGGCAAGAGTATCAGGAGACGATGAATGCATGGAAGCAAG

20 ACTTAGTCTTATTCAAAGGCAGGCAAGAGTATCAGGAGACGATGAATGCATGGAAGCAAG

*************************.**********************************

1RC AGTCTCATATCATGCGCTGGTTCGCTTTGGAAGAGGTACGTTTCCTTATCTTTCAATTTC

16RC AGTCTCATATCATGCGCTGGTTCGCTTTGGAAGAGGTACGTTTCCTTATCTTTCAATTTC

15RC AGTCTCATATCATGCGCTGGTTCGCTTTGGAAGAGGTACGTTTCCTTATCTTTCAATTTC

2RC AGTCTCATATCATGCGCTGGTTCGCTTTGGAAGAGGTACGTTTCCTTATCTTTCAATTTC

10 AGTCTCATATAATGCGCTGGTTCGCTTTGGAAGAGGTATGTTTCCTTATCTTTCAATTTC

17 AGTCTCATATAATGCGCTGGTTCGCTTTGGAAGAGGTATGTTTCCTTATCTTTCAATTTC

14 AGTCTCATATAATGCGCTGGTTCGCTTTGGAAGAGGTATGTTTCCTTATCTTTCAATTTC

13 AGTCTCATATAATGCGCTGGTTCGCTTTGGAAGAGGTATGTTTCCTTATCTTTCAATTTC

12 AGTCTCATATAATGCGCTGGTTCGCTTTGGAAGAGGTATGTTTCCTTATCTTTCAATTTC

8 AGTCTCATATAATGCGCTGGTTCGCTTTGGAAGAGGTATGTTTCCTTATCTTTCAATTTC

7 AGTCTCATATAATGCGCTGGTTCGCTTTGGAAGAGGTATGTTTCCTTATCTTTCAATTTC

19 AGTCTCATATAATGCGCTGGTTCGCTTTGGAAGAGGTATGTTTCCTTATCTTTCAATTTC

18 AGTCTCATATAATGCGCTGGTTCGCTTTGGAAGAGGTATGTTTCCTTATCTTTCAATTTC

22 AGTCTCATATAATGCGCTGGTTCGCTTTGGAAGAGGTATGTTTCCTTATCTTTCAATTTC

21 AGTCTCATATAATGCGCTGGTTCGCTTTGGAAGAGGTATGTTTCCTTATCTTTCAATTTC

9 AGTCTCATATAATGCGCTGGTTCGCTTTGGAAGAGGTATGTTTCCTTATCTTTCAATTTC

6 AGTCTCATATAATGCGCTGGTTCGCTTTGGAAGAGGTATGTTTCCTTATCTTTCAATTTC

5 AGTCTCATATAATGCGCTGGTTCGCTTTGGAAGAGGTATGTTTCCTTATCTTTCAATTTC

4 AGTCTCATATAATGCGCTGGTTCGCTTTGGAAGAGGTATGTTTCCTTATCTTTCAATTTC

3 AGTCTCATATAATGCGCTGGTTCGCTTTGGAAGAGGTATGTTTCCTTATCTTTCAATTTC

20 AGTCTCATATAATGCGCTGGTTCGCTTTGGAAGAGGTATGTTTCCTTATCTTTCAATTTC

********** ***************************.*********************

1RC ATCTATTTAACTCCACCACAGGCTCCACGTTAGTTAACAAAAAACTTGAGATAACAGTCT

16RC ATCTATTTAACTCCACCACAGGCTCCACGTTAGTTAACAAAACACTTGAGATAACAGTCT

15RC ATCTATTTAACTCCACCACAGGCTCCACGTTAGTTAACAAAACACTTGAGATAACAGTCT

2RC ATCTATTTAACTCCACCACAGGCTCCACGTTAGTTAACAAAACACTTGAGATAACAGTCT

10 ATCTATTTAACTCCACCACAGGCTCCACGTTAGTTAACAAAACACTTGAGATAACGGTCT

17 ATCTATTTAACTCCACCACAGGCTCCACGTTAGTTAACAAAACACTTGAGATAACGGTCT

14 ATCTATTTAACTCCACCACAGGCTCCACGTTAGTTAACAAAACACTTGAGATAACGGTCT

13 ATCTATTTAACTCCACCACAGGCTCCACGTTAGTTAACAAAACACTTGAGATAACGGTCT

12 ATCTATTTAACTCCACCACAGGCTCCACGTTAGTTAACAAAACACTTGAGATAACGGTCT

8 ATCTATTTAACTCCACCACAGGCTCCACGTTAGTTAACAAAACACTTGAGATAACGGTCT

7 ATCTATTTAACTCCACCACAGGCTCCACGTTAGTTAACAAAACACTTGAGATAACGGTCT

19 ATCTATTTAACTCCACCACAGGCTCCACGTTAGTTAACAAAACACTTGAGATAACGGTCT

18 ATCTATTTAACTCCACCACAGGCTCCACGTTAGTTAACAAAACACTTGAGATAACGGTCT

22 ATCTATTTAACTCCACCACAGGCTCCACGTTAGTTAACAAAACACTTGAGATAACGGTCT

21 ATCTATTTAACTCCACCACAGGCTCCACGTTAGTTAACAAAACACTTGAGATAACGGTCT

9 ATCTATTTAACTCCACCACAGGCTCCACGTTAGTTAACAAAACACTTGAGATAACGGTCT

6 ATCTATTTAACTCCACCACAGGCTCCACGTTAGTTAACAAAACACTTGAGATAACGGTCT

5 ATCTATTTAACTCCACCACAGGCTCCACGTTAGTTAACAAAACACTTGAGATAACGGTCT

4 ATCTATTTAACTCCACCACAGGCTCCACGTTAGTTAACAAAACACTTGAGATAACGGTCT

3 ATCTATTTAACTCCACCACAGGCTCCACGTTAGTTAACAAAACACTTGAGATAACGGTCT

20 ATCTATTTAACTCCACCACAGGCTCCACGTTAGTTAACAAAACACTTGAGATAACGGTCT

****************************************** ************.****

1RC GACACTCTAAAACAGCTAGACCTCAAACATTCCTCGAAAAATTCTACGCTGGGTATGTTT

16RC GACACTCTAAAACAGCTAGACCTCAAACATTCCTCGAAAAATTCTACGCTGGGTATGTTT

15RC GACACTCTAAAACAGCTAGACCTCAAACATTCCTCGAAAAATTCTACGCTGGGTATGTTT

2RC GACACTCTAAAACAGCTAGACCTCAAACATTCCTCGAAAAATTCTACGCTGGGTATGTTT

10 GACACTCTAAAACAGCTAGACCTCAAACATTCCTCGAAAAATTCTATGCAGGGTATGTCT

17 GACACTCTAAAACAGCTAGACCTCAAACATTCCTCGAAAAATTCTATGCAGGGTATGTCT

14 GACACTCTAAAACAGCTAGACCTCAAACATTCCTCGAAAAATTCTATGCAGGGTATGTCT

13 GACACTCTAAAACAGCTAGACCTCAAACATTCCTCGAAAAATTCTATGCAGGGTATGTCT

12 GACACTCTAAAACAGCTAGACCTCAAACATTCCTCGAAAAATTCTATGCAGGGTATGTCT

8 GACACTCTAAAACAGCTAGACCTCAAACATTCCTCGAAAAATTCTATGCAGGGTATGTCT

7 GACACTCTAAAACAGCTAGACCTCAAACATTCCTCGAAAAATTCTATGCAGGGTATGTCT

19 GACACTCTAAAACAGCTAGACCTCAAACATTCCTCGAAAAATTCTATGCAGGGTATGTCT

18 GACACTCTAAAACAGCTAGACCTCAAACATTCCTCGAAAAATTCTATGCAGGGTATGTCT

22 GACACTCTAAAACAGCTAGACCTCAAACATTCCTCGAAAAATTCTATGCAGGGTATGTCT

21 GACACTCTAAAACAGCTAGACCTCAAACATTCCTCGAAAAATTCTATGCAGGGTATGTCT

9 GACACTCTAAAACAGCTAGACCTCAAACATTCCTCGAAAAATTCTATGCAGGGTATGTCT

6 GACACTCTAAAACAGCTAGACCTCAAACATTCCTCGAAAAATTCTATGCAGGGTATGTCT

5 GACACTCTAAAACAGCTAGACCTCAAACATTCCTCGAAAAATTCTATGCAGGGTATGTCT

4 GACACTCTAAAACAGCTAGACCTCAAACATTCCTCGAAAAATTCTATGCAGGGTATGTCT

3 GACACTCTAAAACAGCTAGACCTCAAACATTCCTCGAAAAATTCTATGCAGGGTATGTCT

20 GACACTCTAAAACAGCTAGACCTCAAACATTCCTCGAAAAATTCTATGCAGGGTATGTCT

**********************************************.** ********.*

1RC CCTTCTACTCTCTTTATCTT-ACCTTAATTCCTATTTAGTCGCGACGAAGACCAAGTTTA

16RC CCTTCTACTCTCTTTATCTT-ACCTTAATTCCTATTTAGTCGCGACGAAGACCAAGTTTA

15RC CCTTCTACTCTCTTTATCTT-ACCTTAATTCCTATTTAGTCGCGACGAAGACCAAGTTTA

2RC CCTTCTACTCTCTTTATCTT-ACCTTAATTCCTATTTAGTCGCGACGAAGACCAAGTTTA

10 CCTTCTACTCTCTTGATCTTCAGCTTAATTCCTGTTTAGTCGCGACGAAGACCAAGTTTA

17 CCTTCTACTCTCTTGATCTTCAGCTTAATTCCTGTTTAGTCGCGACGAAGACCAAGTTTA

14 CCTTCTACTCTCTTGATCTTCAGCTTAATTCCTGTTTAGTCGCGACGAAGACCAAGTTTA

13 CCTTCTACTCTCTTGATCTTCAGCTTAATTCCTGTTTAGTCGCGACGAAGACCAAGTTTA

12 CCTTCTACTCTCTTGATCTTCAGCTTAATTCCTGTTTAGTCGCGACGAAGACCAAGTTTA

8 CCTTCTACTCTCTTGATCTTCAGCTTAATTCCTGTTTAGTCGCGACGAAGACCAAGTTTA

7 CCTTCTACTCTCTTGATCTTCAGCTTAATTCCTGTTTAGTCGCGACGAAGACCAAGTTTA

19 CCTTCTACTCTCTTGATCTTCAGCTTAATTCCTGTTTAGTCGCGACGAAGACCAAGTTTA

18 CCTTCTACTCTCTTGATCTTCAGCTTAATTCCTGTTTAGTCGCGACGAAGACCAAGTTTA

22 CCTTCTACTCTCTTGATCTTCAGCTTAATTCCTGTTTAGTCGCGACGAAGACCAAGTTTA

21 CCTTCTACTCTCTTGATCTTCAGCTTAATTCCTGTTTAGTCGCGACGAAGACCAAGTTTA

9 CCTTCTACTCTCTTGATCTTCAGCTTAATTCCTGTTTAGTCGCGACGAAGACCAAGTTTA

6 CCTTCTACTCTCTTGATCTTCAGCTTAATTCCTGTTTAGTCGCGACGAAGACCAAGTTTA

5 CCTTCTACTCTCTTGATCTTCAGCTTAATTCCTGTTTAGTCGCGACGAAGACCAAGTTTA

4 CCTTCTACTCTCTTGATCTTCAGCTTAATTCCTGTTTAGTCGCGACGAAGACCAAGTTTA

3 CCTTCTACTCTCTTGATCTTCAGCTTAATTCCTGTTTAGTCGCGACGAAGACCAAGTTTA

20 CCTTCTACTCTCTTGATCTTCAGCTTAATTCCTGTTTAGTCGCGACGAAGACCAAGTTTA

************** ***** * **********.**************************

1RC ATTCTAATACATGACTTTTTTACATTTATAAATGCAAATGGTATCACATCTAAACCCTAC

16RC ATTCTAATACATGACTTTTTTACATTTATAAATGCAAATGGTATCACATCTAAACCCTAC

15RC ATTCTAATACATGACTTTTTTACATTTATAAATGCAAATGGTATCACATCTAAACCCTAG

2RC ATTCTAATACATGACTTTTTTACATTTATAAATGCAAATGGTATCACATCTAAACCCTAC

10 ATTATAATACATCACTTTTCTAC----AAGAATACATTTTGAAAAACATTTTGA------

17 ATTATAATACATCACTTTTCTAC----AAGAATACATTTTGAAAAACATTTTGA------

14 ATTATAATACATCACTTTTCTAC----AAGAATACATTTTGAAAAACATTTTGA------

13 ATTATAATACATCACTTTTCTAC----AAGAATACATTTTGAAAAACATTTTGA------

12 ATTATAATACATCACTTTTCTAC----AAGAATACATTTTGAAAAACATTTTGA------

8 ATTATAATACATCACTTTTCTAC----AAGAATACATTTTGAAAAACATTTTGA------

7 ATTATAATACATCACTTTTCTAC----AAGAATACATTTTGAAAAACATTTTGA------

19 ATTATAATACATCACTTTTCTAC----AAGAATACATTTTGAAAAACATTTTGA------

18 ATTATAATACATCACTTTTCTAC----AAGAATACATTTTGAAAAACATTTTGA------

22 ATTATAATACATCACTTTTCTAC----AAGAATACATTTTGAAAAACATTTTGA------

21 ATTATAATACATCACTTTTCTAC----AAGAATACATTTTGAAAAACATTTTGA------

9 ATTATAATACATCACTTTTCTAC----AAGAATACATTTTGAAAAACATTTTGA------

6 ATTATAATACATCACTTTTCTAC----AAGAATACATTTTGAAAAACATTTTGA------

5 ATTATAATACATCACTTTTCTAC----AAGAATACATTTTGAAAAACATTTTGA------

4 ATTATAATACATCACTTTTCTAC----AAGAATACATTTTGAAAAACATTTTGA------

3 ATTATAATACATCACTTTTCTAC----AAGAATACATTTTGAAAAACATTTTGA------

20 ATTATAATACATCACTTTTCTAC----AAGAATACATTTTGAAAAACATTTTGA------

*** ******** ******.*** * .***.** * * * ****.* .*

**STE3-approximate<<<<<<<<<<<<<<<<<**

1RC TAAACTTCACTTGAATTAAGGTCATCTGAACCAGTCAAAATGGAAGCTCGTATTTATCAA

16RC TAAACTTCACTTGAATTAAGGTCATCTGAACCAGTCAAAATGGAAGCTCGTATTTATCAA

15RC TAAACTTCACTTGAATTAAGGTCATCTGAACCAGTCAAAATGGAAGCTCGTATTTATCAA

2RC TAAACTTCACTTGAATTAAGGTCATCTGAACCAGTCAAAATGGAAGCTCGTATTTATCAA

10 ------TTATCTATGATAAAGT-GTCTAGGCTCTTAAGACTGGGAGCTCATAATCATTGA

17 ------TTATCTATGATAAAGT-GTCTAGGCTCTTAAGACTGGGAGCTCATAATCATTGA

14 ------TTATCTATGATAAAGT-GTCTAGGCTCTTAAGACTGGGAGCTCATAATCATTGA

13 ------TTATCTATGATAAAGT-GTCTAGGCTCTTAAGACTGGGAGCTCATAATCATTGA

12 ------TTATCTATGATAAAGT-GTCTAGGCTCTTAAGACTGGGAGCTCATAATCATTGA

8 ------TTATCTATGATAAAGT-GTCTAGGCTCTTAAGACTGGGAGCTCATAATCATTGA

7 ------TTATCTATGATAAAGT-GTCTAGGCTCTTAAGACTGGGAGCTCATAATCATTGA

19 ------TTATCTATGATAAAGT-GTCTAGGCTCTTAAGACTGGGAGCTCATAATCATTGA

18 ------TTATCTATGATAAAGT-GTCTAGGCTCTTAAGACTGGGAGCTCATAATCATTGA

22 ------TTATCTATGATAAAGT-GTCTAGGCTCTTAAGACTGGGAGCTCATAATCATTGA

21 ------TTATCTATGATAAAGT-GTCTAGGCTCTTAAGACTGGGAGCTCATAATCATTGA

9 ------TTATCTATGATAAAGT-GTCTAGGCTCTTAAGACTGGGAGCTCATAATCATTGA

6 ------TTATCTATGATAAAGT-GTCTAGGCTCTTAAGACTGGGAGCTCATAATCATTGA

5 ------TTATCTATGATAAAGT-GTCTAGGCTCTTAAGACTGGGAGCTCATAATCATTGA

4 ------TTATCTATGATAAAGT-GTCTAGGCTCTTAAGACTGGGAGCTCATAATCATTGA

3 ------TTATCTATGATAAAGT-GTCTAGGCTCTTAAGACTGGGAGCTCATAATCATTGA

20 ------TTGTCTATGATAAAGT-GTCTAGGCTCTTAAGACTGGGAGCTCATAATCATTGA

*....*. . ***.** .***...*. * *.* ***.*****.** *.**..*

**<<<<<<<<<<<<<<<<<<<<<<<<<<<<<<<<<<<<<<<<<<<<<<<<<<<<<<<<<<<<**

1RC AATGTGCTCCTGTCTTACCCGCGGTACCTTTGATTAAAGAATCTTGTCTATCAGATTGAT

16RC AATGTGCTCCTGTCTTACCCGCGGTACCTTTGATTAAAGAATCTTGTCTATCAGATTGAT

15RC AATGTGCTCCTGTCTTACCCGCGGTACCTTTGATTAAAGAATCTTGTCTATCAGATTGAT

2RC AATGTGCTCCTGTCTTACCCGCGGTACCTTTGATTAAAGAATCTTGTCTATCAGATTGAT

10 CGTTGTTTTTGCTTTTAACTGTATTTTCCTTCTCGTAGTTATCTATCCTA----------

17 CGTTGTTTTTGCTTTTAACTGTATTTTCCTTCTCGTAGTTATCTATCCTA----------

14 CGTTGTTTTTGCTTTTAACTGTATTTTCCTTCTCGTAGTTATCTATCCTA----------

13 CGTTGTTTTTGCTTTTAACTGTATTTTCCTTCTCGTAGTTATCTATCCTA----------

12 CGTTGTTTTTGCTTTTAACTGTATTTTCCTTCTCGTAGTTATCTATCCTA----------

8 CGTTGTTTTTGCTTTTAACTGTATTTTCCTTCTCGTAGTTATCTATCCTA----------

7 CGTTGTTTTTGCTTTTAACTGTATTTTCCTTCTCGTAGTTATCTATCCTA----------

19 CGTTGTTTTTGCTTTTAACTGTATTTTCCTTCTCGTAGTTATCTATCCTA----------

18 CGTTGTTTTTGCTTTTAACTGTATTTTCCTTCTCGTAGTTATCTATCCTA----------

22 CGTTGTTTTTGCTTTTAACTGTATTTTCCTTCTCGTAGTTATCTATCCTA----------

21 CGTTGTTTTTGCTTTTAACTGTATTTTCCTTCTCGTAGTTATCTATCCTA----------

9 CGTTGTTTTTGCTTTTAACTGTATTTTCCTTCTCGTAGTTATCTATCCTA----------

6 CGTTGTTTTTGCTTTTAACTGTATTTTCCTTCTCGTAGTTATCTATCCTA----------

5 CGTTGTTTTTGCTTTTAACTGTATTTTCCTTCTCGTAGTTATCTATCCTA----------

4 CGTTGTTTTTGCTTTTAACTGTATTTTCCTTCTCGTAGTTATCTATCCTA----------

3 CGTTGTTTTTGCTTTTAACTGTATTTTCCTTCTCGTAGTTATCTATCCTA----------

20 CGTTGTTTTTGCTTTTAACTGTATTTTCCTTCTCGTAGTTATCTATCCTA----------

.* .*.. *.*** *.*.. * .*.** . *. **** .***

**<<<<<<<<<<<<<<<<<<<<<<<<<<<<<<<<<<<<<<<<<<<<<<<<<<<<<<<<<<<<**

1RC CAGAAAAGTCATAAGCTGGCGGTGTAGATGACTCCGA-----TTCCCTTCTTCCTGATAT

16RC CAGAAAAGTCATAAGCTGGCGGTGTAGATGACTCCGA-----TTCCCTTCTTCCTGATAT

15RC CAGAAAAGTCATAAGCTGGCGGTGTAGATGACTCCGA-----TTCCCTTCTTCCTGATAT

2RC CAGAAAAGTCATAAGCTGGCGGTGTAGATGACTCCGA-----TTCCCTTCTTCCTGATAT

10 -GGACTAGGAGGAAACGAATAGTTCGAAAGGCTTGAAATACGATCGTTTCTCTGTGAGAA

17 -GGACTAGGAGGAAACGAATAGTTCGAAAGGCTTGAAATACGATCGTTTCTCTGTGAGAA

14 -GGACTAGGAGGAAACGAATAGTTCGAAAGGCTTGAAATACGATCGTTTCTCTGTGAGAA

13 -GGACTAGGAGGAAACGAATAGTTCGAAAGGCTTGAAATACGATCGTTTCTCTGTGAGAA

12 -GGACTAGGAGGAAACGAATAGTTCGAAAGGCTTGAAATACGATCGTTTCTCTGTGAGAA

8 -GGACTAGGAGGAAACGAATAGTTCGAAAGGCTTGAAATACGATCGTTTCTCTGTGAGAA

7 -GGACTAGGAGGAAACGAATAGTTCGAAAGGCTTGAAATACGATCGTTTCTCTGTGAGAA

19 -GGACTAGGAGGAAACGAATAGTTCGAAAGGCTTGAAATACGATCGTTTCTCTGTGAGAA

18 -GGACTAGGAGGAAACGAATAGTTCGAAAGGCTTGAAATACGATCGTTTCTCTGTGAGAA

22 -GGACTAGGAGGAAACGAATAGTTCGAAAGGCTTGAAATACGATCGCTTCTCTGTGAGAA

21 -GGACTAGGAGGAAACGAATAGTTCGAAAGGCTTGAAATACGATCGTTTCTCTGTGAGAA

9 -GGACTAGGAGGAAACGAATAGTTCGAAAGGCTTGAAATACGATCGTTTCTCTGTGAGAA

6 -GGACTAGGAGGAAACGAATAGTTCGAAAGGCTTGAAATACGATCGTTTCTCTGTGAGAA

5 -GGACTAGGAGGAAACGAATAGTTCGAAAGGCTTGAAATACGATCGTTTCTCTGTGAGAA

4 -GGACTAGGAGGAAACGAATAGTTCGAAAGGCTTGAAATACGATCGTTTCTCTGTGAGAA

3 -GGACTAGGAGGAAACGAATAGTTCGAAAGGCTTGAAATACGATCGTTTCTCTGTGAGAA

20 -GGACTAGGAGGAAACGAATAGTTCGAAAGGCTTGAAATACGATCGTTTCTCTGTGAGAA

.** ** . **.* ....** ...* *.**. .* ** .****.. *** *

**<<<<<<<<<<<<<<<<<<<<<<<<<<<<<<<<<<<<<<<<<<<<<<<<<<<<<<<<<<<<**

1RC TTTCTCATAG---------------------CATTTCATTATCACTTTTC----------

16RC TTTCTCATAG---------------------CATTTCATTATCACTTTTC----------

15RC TTTCTCATAG---------------------CATTTCATTATCACTTTTC----------

2RC TTTCTCATAG---------------------CATTTCATTATCACTTTTC----------

10 CCTATAACAAATCAGTCTGAAGTCTGAGACCAACCTCAAACTCACTGTCTAAACTTCGGC

17 CCTATAACAAATCAGTCTGAAGTCTGAGACCAACCTCAAACTCACTGTCTAAACTTCGGC

14 CCTATAACAAATCAGTCTGAAGTCTGAGACCAACCTCAAACTCACTGTCTAAACTTCGGC

13 CCTATAACAAATCAGTCTGAAGTCTGAGACCAACCTCAAACTCACTGTCTAAACTTCGGC

12 CCTATAACAAATCAGTCTGAAGTCTGAGACCAACCTCAAACTCACTGTCTAAACTTCGGC

8 CCTATAACAAATCAGTCTGAAGTCTGAGACCAACCTCAAACTCACTGTCTAAACTTCGGC

7 CCTATAACAAATCAGTCTGAAGTCTGAGACCAACCTCAAACTCACTGTCTAAACTTCGGC

19 CCTATAACAAATCAGTCTGAAGTCTGAGACCAACCTCAAACTCACTGTCTAAACTTCGGC

18 CCTATAACAAATCAGTCTGAAGTCTGAGACCAACCTCAAACTCACTGTCTAAACTTCGGC

22 CCTATAACAAATCAGTCTGAAGTCTGAGACCAACCTCAAACTCACTGTCTAAACTTCGGC

21 CCTATAACAAATCAGTCTGAAGTCTGAGACCAACCTCAAACTCACTGTCTAAACTTCGGC

9 CCTATAACAAATCAGTCTGAAGTCTGAGACCAACCTCAAACTCACTGTCTAAACTTCGGC

6 CCTATAACAAATCAGTCTGAAGTCTGAGACCAACCTCAAACTCACTGTCTAAACTTCGGC

5 CCTATAACAAATCAGTCTGAAGTCTGAGACCAACCTCAAACTCACTGTCTAAACTTCGGC

4 CCTATAACAAATCAGTCTGAAGTCTGAGACCAACCTCAAACTCACTGTCTAAACTTCGGC

3 CCTATAACAAATCAGTCTGAAGTCTGAGACCAACCTCAAACTCACTGTCTAAACTTCGGC

20 CCTATAACAAATCAGTCTGAAGTCTGAGACCAACCTCAAACTCACTGTCTAAACTTCGGC

..* * *.*. *..*** ***** *..

**<<<<<<<<<<<<<<<<<<<<<<<<<<<<<<<<<<<<<<<<<<<<<<<<<<<<<<<<<<<<**

1RC -------------TGACAAATAAATACCATGATACGTACTCCTTCCTTGCTTCTGCGCCA

16RC -------------TGACAAATAAATACCATGATACGTACTCCTTCCTTGCTTCTGCGCCA

15RC -------------TGACAAATAAATACCATGATACGTACTCCTTCCTTGCTTCTGCGCCA

2RC -------------TGACAAATAAATACCATGATACGTACTCCTTCCTTGCTTCTGCGCCA

10 TTCAGGCAAGGCATGAAAATGCAACTCAATCTAATGTACTCTTTTCTAGCCTCCTCTGAA

17 TTCAGGCAAGGCATGAAAATGCAACTCAATCTAATGTACTCTTTTCTAGCCTCCTCTGAA

14 TTCAGGCAAGGCATGAAAATGCAACTCAATCTAATGTACTCTTTTCTAGCCTCCTCTGAA

13 TTCAGGCAAGGCATGAAAATGCAACTCAATCTAATGTACTCTTTTCTAGCCTCCTCTGAA

12 TTCAGGCAAGGCATGAAAATGCAACTCAATCTAATGTACTCTTTTCTAGCCTCCTCTGAA

8 TTCAGGCAAGGCATGAAAATGCAACTCAATCTAATGTACTCTTTTCTAGCCTCCTCTGAA

7 TTCAGGCAAGGCATGAAAATGCAACTCAATCTAATGTACTCTTTTCTAGCCTCCTCTGAA

19 TTCAGGCAAGGCATGAAAATGCAACTCAATCTAATGTACTCTTTTCTAGCCTCCTCTGAA

18 TTCAGGCAAGGCATGAAAATGCAACTCAATCTAATGTACTCTTTTCTAGCCTCCTCTGAA

22 TTCAGGCAAGGCATGAAAATGCAACTCAATCTAATGTACTCTTTTCTAGCCTCCTCTGAA

21 TTCAGGCAAGGCATGAAAATGCAACTCAATCTAATGTACTCTTTTCTAGCCTCCTCTGAA

9 TTCAGGCAAGGCATGAAAATGCAACTCAATCTAATGTACTCTTTTCTAGCCTCCTCTGAA

6 TTCAGGCAAGGCATGAAAATGCAACTCAATCTAATGTACTCTTTTCTAGCCTCCTCTGAA

5 TTCAGGCAAGGCATGAAAATGCAACTCAATCTAATGTACTCTTTTCTAGCCTCCTCTGAA

4 TTCAGGCAAGGCATGAAAATGCAACTCAATCTAATGTACTCTTTTCTAGCCTCCTCTGAA

3 TTCAGGCAAGGCATGAAAATGCAACTCAATCTAATGTACTCTTTTCTAGCCTCCTCTGAA

20 TTCAGGCAAGGCATGAAAATGCAACTCAATCTAATGTACTCTTTTCTAGCCTCCTCTGAA

*** ** **. * ** *.******.**.** **.**. * *

**<<<<<<<<<<<<<<<<<<<<<<<<<<<<<<<<<<<<<<<<<<<<<<<<<<<<<<<<<<<<**

1RC ATTCCGAAAAACCCGAAAAATATGAAGCTTGAGATAGGGGTCAGCAACCATACAATTTGA

16RC ATTCCGAAAAACCCGAAAAATATGAAGCTTGAGATAGGGGTCAGCAACCATACAATTTGA

15RC ATTCCGAAAAACCCGAAAAATATGAAGCTTGAGATAGGGGTCAGCAACCATACAATTTGA

2RC ATTCCGAAAAACCCGAAAAATATGAAGCTTGAGATAGGGGTCAGCAACCATACAATTTGA

10 AGTCCAAAGAACAAGAAGAAGATAATAGATGACA---AGATTGGC-ACTATCCAGCTCG-

17 AGTCCAAAGAACAAGAAGAAGATAATAGATGACA---AGATTGGC-ACTATCCAGCTCG-

14 AGTCCAAAGAACAAGAAGAAGATAATAGATGACA---AGATTGGC-ACTATCCAGCTCG-

13 AGTCCAAAGAACAAGAAGAAGATAATAGATGACA---AGATTGGC-ACTATCCAGCTCG-

12 AGTCCAAAGAACAAGAAGAAGATAATAGATGACA---AGATTGGC-ACTATCCAGCTCG-

8 AGTCCAAAGAACAAGAAGAAGATAATAGATGACA---AGATTGGC-ACTATCCAGCTCG-

7 AGTCCAAAGAACAAGAAGAAGATAATAGATGACA---AGATTGGC-ACTATCCAGCTCG-

19 AGTCCAAAGAACAAGAAGAAGATAATAGATGACA---AGATTGGC-ACTATCCAGCTCG-

18 AGTCCAAAGAACAAGAAGAAGATAATAGATGACA---AGATTGGC-ACTATCCAGCTCG-

22 AGTCCAAAGAACAAGAAGAAGATAATAGATGACA---AGATTGGC-ACTATCCAGCTCG-

21 AGTCCAAAGAACAAGAAGAAGATAATAGATGACA---AGATTGGC-ACTATCCAGCTCG-

9 AGTCCAAAGAACAAGAAGAAGATAATAGATGACA---AGATTGGC-ACTATCCAGCTCG-

6 AGTCCAAAGAACAAGAAGAAGATAATAGATGACA---AGATTGGC-ACTATCCAGCTCG-

5 AGTCCAAAGAACAAGAAGAAGATAATAGATGACA---AGATTGGC-ACTATCCAGCTCG-

4 AGTCCAAAGAACAAGAAGAAGATAATAGATGACA---AGATTGGC-ACTATCCAGCTCG-

3 AGTCCAAAGAACAAGAAGAAGATAATAGATGACA---AGATTGGC-ACTATCCAGCTCG-

20 AGTCCAAAGAACAAGAAGAAGATAATAGATGACA---AGATTGGC-ACTATCCAGCTCG-

* ***.**.*** ***.** **.* . *** * .*.*..** **.** **..*.*

**<<<<<<<<<<<<<<<<<<<<<<<<<<<<<<<<<<<<<<<<<<<<<<<<<<<<<<<<<<<<**

1RC ATCTGTGAAGACGCTCTGTCTGGCATAAG---TTTCAG----ACTATATTGTCCTATCCT

16RC ATCTGTGAAGACGCTCTGTCTGGCATAAG---TTTCAG----ACTATATTGTCCTATCCT

15RC ATCTGTGAAGACGCTCTGTCTGGCATAAG---TTTCAG----ACTATATTGTCCTATGCT

2RC ATCTGTGAAGACGCTCTGTCTGGCATAAG---TTTCAG----ACTATATTGTCCTATCCT

10 --CTATATTTACGCTTAGTTGATGTTGAGTCATTTCAGATTTACTAAGTCGATAGATGAC

17 --CTATATTTACGCTTAGTTGATGTTGAGTCATTTCAGATTTACTAAGTCGATAGATGAC

14 --CTATATTTACGCTTAGTTGATGTTGAGTCATTTCAGATTTACTAAGTCGATAGATGAC

13 --CTATATTTACGCTTAGTTGATGTTGAGTCATTTCAGATTTACTAAGTCGATAGATGAC

12 --CTATATTTACGCTTAGTTGATGTTGAGTCATTTCAGATTTACTAAGTCGATAGATGAC

8 --CTATATTTACGCTTAGTTGATGTTGAGTCATTTCAGATTTACTAAGTCGATAGATGAC

7 --CTATATTTACGCTTAGTTGATGTTGAGTCATTTCAGATTTACTAAGTCGATAGATGAC

19 --CTATATTTACGCTTAGTTGATGTTGAGTCATTTCAGATTTACTAAGTCGATAGATGAC

18 --CTATATTTACGCTTAGTTGATGTTGAGTCATTTCAGATTTACTAAGTCGATAGATGAC

22 --CTATATTTACGCTTAGTTGATGTTGAGTCATTTCAGATTTACTAAGTCGATAGATGAC

21 --CTATATTTACGCTTAGTTGATGTTGAGTCATTTCAGATTTACTTAGTCGATAGATGAC

9 --CTATATTTACGCTTAGTTGATGTTGAGTCATTTCAGATTTACTTAGTCGATAGATGAC

6 --CTATATTTACGCTTAGTTGATGTTGAGTCATTTCAGATTTACTTAGTCGATAGATGAC

5 --CTATATTTACGCTTAGTTGATGTTGAGTCATTTCAGATTTACTTAGTCGATAGATGAC

4 --CTATATTTACGCTTAGTTGATGTTGAGTCATTTCAGATTTACTTAGTCGATAGATGAC

3 --CTATATTTACGCTTAGTTGATGTTGAGTCATTTCAGATTTACTTAGTCGATAGATGAC

20 --CTATATTTACGCTTAGTTGATGTTGAGTCATTTCAGATTTACTTAGTCGATAGATGAC

**.*. *****. **. . *.** ****** *** .*.* . ** .

**<<<<<<<<<<<<<<<<<<<<<<<<<<<<<<<<<<<<<<<<<<<<<<<<<<<<<<<<<<<<**

1RC TGAAAATTCTGCATGAACGTAATCCCAACCCTTATACGG-ACTAATTCTTGCTGATGTGG

16RC TGAAAATTCTGCATGAACGTAATCCCAACCCTTATACGG-ACTAATTCTTGCTGATGTGG

15RC TGAAAATTCTGCATGAACGTAATCCCAACCCTTATACGG-ACTAATTCTTGCTGATGTGG

2RC TGAAAATTCTGCATGAACGTAATCCCAACCCTTATACGG-ACTAATTCTTGCTGATGTGG

10 GGAAAAGTGAGCGTGAACTTGGTTCCAGCTATTATAAGGTAGTAAATGTCCGTTATTTGC

17 GGAAAAGTGAGCGTGAACTTGGTTCCAGCTATTATAAGGTAGTAAATGTCCGTTATTTGC

14 GGAAAAGTGAGCGTGAACTTGGTTCCAGCTATTATAAGGTAGTAAATGTCCGTTATTTGC

13 GGAAAAGTGAGCGTGAACTTGGTTCCAGCTATTATAAGGTAGTAAATGTCCGTTATTTGC

12 GGAAAAGTGAGCGTGAACTTGGTTCCAGCTATTATAAGGTAGTAAATGTCCGTTATTTGC

8 GGAAAAGTGAGCGTGAACTTGGTTCCAGCTATTATAAGGTAGTAAATGTCCGTTATTTGC

7 GGAAAAGTGAGCGTGAACTTGGTTCCAGCTATTATAAGGTAGTAAATGTCCGTTATTTGC

19 GGAAAAGTGAGCGTGAACTTGGTTCCAGCTATTATAAGGTAGTAAATGTCCGTTATTTGC

18 GGAAAAGTGAGCGTGAACTTGGTTCCAGCTATTATAAGGTAGTAAATGTCCGTTATTTGC

22 GGAAAAGTGAGCGTGAACTTGGTTCCAGCTATTATAAGGTAGTAAATGTCCGTTATTTGC

21 GGAAAAGTGAGCGTGAACTTGGTTCCAGCTATTATAAGGTAGTAAATGTCCGTTATTTGC

9 GGAAAAGTGAGCGTGAACTTGGTTCCAGCTATTATAAGGTAGTAAATGTCCGTTATTTGC

6 GGAAAAGTGAGCGTGAACTTGGTTCCAGCTATTATAAGGTAGTAAATGTCCGTTATTTGC

5 GGAAAAGTGAGCGTGAACTTGGTTCCAGCTATTATAAGGTAGTAAATGTCCGTTATTTGC

4 GGAAAAGTGAGCGTGAACTTGGTTCCAGCTATTATAAGGTAGTAAATGTCCGTTATTTGC

3 GGAAAAGTGAGCGTGAACTTGGTTCCAGCTATTATAAGGTAGTAAATGTCCGTTATTTGC

20 GGAAAAGTGAGCGTGAACTTGGTTCCAGCTATTATAAGGTAGTAAATGTCCGTTATTTGC

***** * **.***** *..*.***.*. ***** ** * *** * *. * ** **

**<<<<<<<<<<<<<<<<<<<<<<<<<<<<<<<<<<<<<<<<<<<<<<<<<<<<<<<<<<<<**

1RC TGTTGTAAACAAGAGCGACGAGGTTGAAAACTAGGTTTAATACAATCAATGTTGAT----

16RC TGTTGTAAACAAGAGCGACGAGGTTGAAAACTAGGTTTAATACAATCAATGTTGAT----

15RC TGTTGTAAACAAGAGCGACGAGGTTGAAAACTAGGTTTAATACAATCAATGTTGAT----

2RC TGTTGTAAACAAGAGCGACGAGGTTGAAAACTAGGTTTAATACAATCAATGTTGAT----

10 T----TGAATGTTGACGACTAGTTGAGCTATTGCGGCTGCGACAGTGAGAGTAAGCATCA

17 T----TGAATGTTGACGACTAGTTGAGCTATTGCGGCTGCGACAGTGAGAGTAAGCATCA

14 T----TGAATGTTGACGACTAGTTGAGCTATTGCGGCTGCGACAGTGAGAGTAAGCATCA

13 T----TGAATGTTGACGACTAGTTGAGCTATTGCGGCTGCGACAGTGAGAGTAAGCATCA

12 T----TGAATGTTGACGACTAGTTGAGCTATTGCGGCTGCGACAGTGAGAGTAAGCATCA

8 T----TGAATGTTGACGACTAGTTGAGCTATTGCGGCTGCGACAGTGAGAGTAAGCATCA

7 T----TGAATGTTGACGACTAGTTGAGCTATTGCGGCTGCGACAGTGAGAGTAAGCATCA

19 T----TGAATGTTGACGACTAGTTGAGCTATTGCGGCTGCGACAGTGAGAGTAAGCATCA

18 T----TGAATGTTGACGACTAGTTGAGCTATTGCGGCTGCGACAGTGAGAGTAAGCATCA

22 T----TGAATGTTGACGACTAGTTGAGCTATTGCGGCTGCGACAGTGAGAGTAAGCATCA

21 T----TGAATGTTGACGACTAGTTGAGCTATTGCGGCTGCGACAGTGAGAGTAAGCATCA

9 T----TGAATGTTGACGACTAGTTGAGCTATTGCGGCTGCGACAGTGAGAGTAAGCATCA

6 T----TGAATGTTGACGACTAGTTGAGCTATTGCGGCTGCGACAGTGAGAGTAAGCATCA

5 T----TGAATGTTGACGACTAGTTGAGCTATTGCGGCTGCGACAGTGAGAGTAAGCATCA

4 T----TGAATGTTGACGACTAGTTGAGCTATTGCGGCTGCGACAGTGAGAGTAAGCATCA

3 T----TGAATGTTGACGACTAGTTGAGCTATTGCGGCTGCGACAGTGAGAGTAAGCATCA

20 T----TGAATGTTGACGACTAGTTGAGCTATTGCGGCTGCGACAGTGAGAGTAAGCATCA

* *.**.. ..**** ** * .. *.*. * .*. ***.* *. ** ...

**<<<<<<<<<<<<<<<<<<<<<<<<<<<<<<<<<<<<<<<<<<<<<<<<<<<<<<<<<<<<**

1RC -----GTAAGCCCTAGAAGTCGATAGAATCTACTCGCAGTAAAATTGGTAGAGTTTCTAA

16RC -----GTAAGCCCTAGAAGTCGATAGAATCTACTCGCAGTAAAATTGGTAGAGTTTCTAA

15RC -----GTAAGCCCTAGAAGTCGATAGAATCTACTCGCAGTAAAATTGGTAGAGTTTCTAA

2RC -----GTAAGCCCTAGAAGTCGATAGAATCTACTCGCAGTAAAATTGGTAGAGTTTCTAA

10 CCACGGCAAGTCCTATCAAACGGAAGTATCTACTCTTAGTGAGACCG-------------

17 CCACGGCAAGTCCTATCAAACGGAAGTATCTACTCTTAGTGAGACCG-------------

14 CCACGGCAAGTCCTATCAAACGGAAGTATCTACTCTTAGTGAGACCG-------------

13 CCACGGCAAGTCCTATCAAACGGAAGTATCTACTCTTAGTGAGACCG-------------

12 CCACGGCAAGTCCTATCAAACGGAAGTATCTACTCTTAGTGAGACCG-------------

8 CCACGGCAAGTCCTATCAAACGGAAGTATCTACTCTTAGTGAGACCG-------------

7 CCACGGCAAGTCCTATCAAACGGAAGTATCTACTCTTAGTGAGACCG-------------

19 CCACGGCAAGTCCTATCAAACGGAAGTATCTACTCTTAGTGAGACCG-------------

18 CCACGGCAAGTCCTATCAAACGGAAGTATCTACTCTTAGTGAGACCG-------------

22 CCACGGCAAGTCCTATCAAACGGAAGTATCTACTCTTAGTGAGACCG-------------

21 CCACGGCAAGTCCTATCAAACGGAAGTATCTACTCTTAGTGAGACCG-------------

9 CCACGGCAAGTCCTATCAAACGGAAGTATCTACTCTTAGTGAGACCG-------------

6 CCACGGCAAGTCCTATCAAACGGAAGTATCTACTCTTAGTGAGACCG-------------

5 CCACGGCAAGTCCTATCAAACGGAAGTATCTACTCTTAGTGAGACCG-------------

4 CCACGGCAAGTCCTATCAAACGGAAGTATCTACTCTTAGTGAGACCG-------------

3 CCACGGCAAGTCCTATCAAACGGAAGTATCTACTCTTAGTGAGACCG-------------

20 CCACGGCAAGTCCTATCAAACGGAAGTATCTACTCTTAGTGAGACCG-------------

*.***.**** *. **. ** ******** .***.*.*..*

**<<<<<<<<<<<<<<<<<<<<<<<<<<<<<<<<<<<<<<<<<<<<<<<<<<<<<<<<<<<<**

1RC AAATGTCATCACTTCGTAATCTACGTTTAATGAGATGGAATATTGCGAAAGCTTCGTAAA

16RC AAATGTCATCACTTCGTAATCTACGTTTAATGAGATGGAATATTGCGAAAGCTTCGTAAA

15RC AAATGTCATCACTTCGTAATCTACGTTTAATGAGATGGAATATTGCGAAAGCTTCGTAAA

2RC AAATGTCATCACTTCGTAATCTACGTTTAATGAGATGGAATATTGCGAAAGCTTCGTAAA

10 -TGCGTTTTCATTTTGAA-------------------CAAGTCTGTGAATGTTTCTTTAT

17 -TGCGTTTTCATTTTGAA-------------------CAAGTCTGTGAATGTTTCTTTAT

14 -TGCGTTTTCATTTTGAA-------------------CAAGTCTGTGAATGTTTCTTTAT

13 -TGCGTTTTCATTTTGAA-------------------CAAGTCTGTGAATGTTTCTTTAT

12 -TGCGTTTTCATTTTGAA-------------------CAAGTCTGTGAATGTTTCTTTAT

8 -TGCGTTTTCATTTTGAA-------------------CAAGTCTGTGAATGTTTCTTTAT

7 -TGCGTTTTCATTTTGAA-------------------CAAGTCTGTGAATGTTTCTTTAT

19 -TGCGTTTTCATTTTGAA-------------------CAAGTCTGTGAATGTTTCTTTAT

18 -TGCGTTTTCATTTTGAA-------------------CAAGTCTGTGAATGTTTCTTTAT

22 -TGCGTTTTCATTTTGAA-------------------CAAGTCTGTGAATGTTTCTTTAT

21 -TGCGTTTTCATTTTGAA-------------------CAAGTCTGTGAATGTTTCTTTAT

9 -TGCGTTTTCATTTTGAA-------------------CAAGTCTGTGAATGTTTCTTTAT

6 -TGCGTTTTCATTTTGAA-------------------CAAGTCTGTGAATGTTTCTTTAT

5 -TGCGTTTTCATTTTGAA-------------------CAAGTCTGTGAATGTTTCTTTAT

4 -TGCGTTTTCATTTTGAA-------------------CAAGTCTGTGAATGTTTCTTTAT

3 -TGCGTTTTCATTTTGAA-------------------CAAGTCTGTGAATGTTTCTTTAT

20 -TGCGTTTTCATTTTGAA-------------------CAAGTCTGTGAATGTTTCTTTAT

..**. ***.**.* * ** .**.*** *.*** * *

**<<<<<<<<<<<<<<<<<<<<<<<<<<<<<<<<<<<<<<<<<<<<<<<<<<<<<<<<<<<<**

1RC GT---TAGATACTTC------------CTACTAAGAGCATACTTTACGCACAGCCATAAA

16RC GT---TAGATACTTC------------CTACTAAGAGCATACTTTACGCACAGCCATAAA

15RC GT---TAGATACTTC------------CTACTAAGAGCATACTTTACGCACAGCCATAAA

2RC GT---TAGATACTTC------------CTACTAAGAGCATACTTTACGCACAGCCATAAA

10 GCTGGTAGAAACTTCGGATTGTCTTGACTATCGAAAATGGGCATTATGTAATCCTTCAAA

17 GCTGGTAGAAACTTCGGATTGTCTTGACTATCGAAAATGGGCATTATGTAATCCTTCAAA

14 GCTGGTAGAAACTTCGGATTGTCTTGACTATCGAAAATGGGCATTATGTAATCCTTCAAA

13 GCTGGTAGAAACTTCGGATTGTCTTGACTATCGAAAATGGGCATTATGTAATCCTTCAAA

12 GCTGGTAGAAACTTCGGATTGTCTTGACTATCGAAAATGGGCATTATGTAATCCTTCAAA

8 GCTGGTAGAAACTTCGGATTGTCTTGACTATCGAAAATGGGCATTATGTAATCCTTCAAA

7 GCTGGTAGAAACTTCGGATTGTCTTGACTATCGAAAATGGGCATTATGTAATCCTTCAAA

19 GCTGGTAGAAACTTCGGATTGTCTTGACTATCGAAAATGGGCATTATGTAATCCTTCAAA

18 GCTGGTAGAAACTTCGGATTGTCTTGACTATCGAAAATGGGCATTATGTAATCCTTCAAA

22 GCTGGTAGAAACTTCGGATTGTCTTGACTATCGAAAATGGGCATTATGTAATCCTTCAAA

21 GCTGGTAGAAACTTCGGATTGTCTTGACTATCGAAAATGGGCATTATGTAATCCTTCAAA

9 GCTGGTAGAAACTTCGGATTGTCTTGACTATCGAAAATGGGCATTATGTAATCCTTCAAA

6 GCTGGTAGAAACTTCGGATTGTCTTGACTATCGAAAATGGGCATTATGTAATCCTTCAAA

5 GCTGGTAGAAACTTCGGATTGTCTTGACTATCGAAAATGGGCATTATGTAATCCTTCAAA

4 GCTGGTAGAAACTTCGGATTGTCTTGACTATCGAAAATGGGCATTATGTAATCCTTCAAA

3 GCTGGTAGAAACTTCGGATTGTCTTGACTATCGAAAATGGGCATTATGTAATCCTTCAAA

20 GCTGGTAGAAACTTCGGATTGTCTTGACTATCGAAAATGGGCATTATGTAATCCTTCAAA

*. **** ***** ***...*.*... .* ***.*.* *. .***

**<<<<<<<<<<<<<<<<<<<<<<<<<<<<<<<<<<<<<<<<<<<<<<<<<<<<<<<<<<<<**

1RC ACGC---------TGAAAAGATAGCCAATGCTATCG-------GCACAGCATTGAGGACG

16RC ACGC---------TGAAAAGATAGCCAATGCTATCG-------GCACAGCATTGAGGACG

15RC ACGC---------TGAAAAGATAGCCAATGCTATCG-------GCACAGCATTGAGGACG

2RC ACGC---------TGAAAAGATAGCCAATGCTATCG-------GCACAGCATTGAGGACG

10 ATGTAAACTCACATGAGTAAAAAGCCGCTACCATTGCAAAAATAAATGGAAGAAGGGACG

17 ATGTAAACTCACATGAGTAAAAAGCCGCTACCATTGCAAAAATAAATGGAAGAAGGGACG

14 ATGTAAACTCACATGAGTAAAAAGCCGCTACCATTGCAAAAATAAATGGAAGAAGGGACG

13 ATGTAAACTCACATGAGTAAAAAGCCGCTACCATTGCAAAAATAAATGGAAGAAGGGACG

12 ATGTAAACTCACATGAGTAAAAAGCCGCTACCATTGCAAAAATAAATGGAAGAAGGGACG

8 ATGTAAACTCACATGAGTAAAAAGCCGCTACCATTGCAAAAATAAATGGAAGAAGGGACG

7 ATGTAAACTCACATGAGTAAAAAGCCGCTACCATTGCAAAAATAAATGGAAGAAGGGACG

19 ATGTAAACTCACATGAGTAAAAAGCCGCTACCATTGCAAAAATAAATGGAAGAAGGGACG

18 ATGTAAACTCACATGAGTAAAAAGCCGCTACCATTGCAAAAATAAATGGAAGAAGGGACG

22 ATGTAAACTCACATGAGTAAAAAGCCGCTACCATTGCAAAAATAAATGGAAGAAGGGACG

21 ATGTAAACTCACATGAGTAAAAAGCCGCTACCATTGCAAAAATAAATGGAAGAAGGGACG

9 ATGTAAACTCACATGAGTAAAAAGCCGCTACCATTGCAAAAATAAATGGAAGAAGGGACG

6 ATGTAAACTCACATGAGTAAAAAGCCGCTACCATTGCAAAAATAAATGGAAGAAGGGACG

5 ATGTAAACTCACATGAGTAAAAAGCCGCTACCATTGCAAAAATAAATGGAAGAAGGGACG

4 ATGTAAACTCACATGAGTAAAAAGCCGCTACCATTGCAAAAATAAATGGAAGAAGGGACG

3 ATGTAAACTCACATGAGTAAAAAGCCGCTACCATTGCAAAAATAAATGGAAGAAGGGACG

20 ATGTAAACTCACATGAGTAAAAAGCCGCTACCATTGCAAAAATAAATGGAAGAAGGGACG

*.*. ***. *.* ****. *.*.**.* . *..* * ..*****

**<<<<<<<<<<<<<<<<<<<<<<<<<<<<<<<<<<<<<<<<<<<<<<<<<<<<<<<<<<<<**

1RC ATTATAC------------TGCCTGCTGTATTAAAAGTGTACTGAAAGCATCCTACGTTC

16RC ATTATAC------------TGCCTGCTGTATTAAAAGTGTACTGGAAGCATCCTACGTTC

15RC ATTATAC------------TGCCTGCTGTATTAAAAGTGTACTGGAAGCATCCTACGTTC

2RC ATTATAC------------TGCCTGCTGTATTAAAAGTGTACTGGAAGCATCCTACGTTC

10 TGTGAACGAGAGAAAGCCATGAAGGCCAAACCACAGGCATA-------CAACCAATACCT

17 TGTGAACGAGAGAAAGCCATGAAGGCCAAACCACAGGCATA-------CAACCAATACCT

14 TGTGAACGAGAGAAAGCCATGAAGGCCAAACCACAGGCATA-------CAACCAATACCT

13 TGTGAACGAGAGAAAGCCATGAAGGCCAAACCACAGGCATA-------CAACCAATACCT

12 TGTGAACGAGAGAAAGCCATGAAGGCCAAACCACAGGCATA-------CAACCAATACCT

8 TGTGAACGAGAGAAAGCCATGAAGGCCAAACCACAGGCATA-------CAACCAATACCT

7 TGTGAACGAGAGAAAGCCATGAAGGCCAAACCACAGGCATA-------CAACCAATACCT

19 TGTGAACGAGAGAAAGCCATGAAGGCCAAACCACAGGCATA-------CAACCAATACCT

18 TGTGAACGAGAGAAAGCCATGAAGGCCAAACCACAGGCATA-------CAACCAATACCT

22 TGTGAACGAGAGAAAGCCATGAAGGCCAAACCACAGGCATA-------CAACCAATACCT

21 TGTGAACGAGAGAAAGCCATGAAGGCCAAACCACAGGCATA-------CAACCAATACCT

9 TGTGAACGAGAGAAAGCCATGAAGGCCAAACCACAGGCATA-------CAACCAATACCT

6 TGTGAACGAGAGAAAGCCATGAAGGCCAAACCACAGGCATA-------CAACCAATACCT

5 TGTGAACGAGAGAAAGCCATGAAGGCCAAACCACAGGCATA-------CAACCAATACCT

4 TGTGAACGAGAGAAAGCCATGAAGGCCAAACCACAGGCATA-------CAACCAATACCT

3 TGTGAACGAGAGAAAGCCATGAAGGCCAAACCACAGGCATA-------CAACCAATACCT

20 TGTGAACGAGAGAAAGCCATGAAGGCCAAACCACAGGCATA-------CAACCAATACCT

*. ** ** **.. *..* *.*..** ** ** *.....

**<<<<<<<<<<<<<<<<<<<<<<<<<<<<<<<<<<<<<<<<<<<<<<<<<<<<<<<<<<<<**

1RC TCATAGATGTTAAAGCGATGACCCTGGAATACTGCATGCAGAGCCATAACAAGTACTGGT

16RC TCATAGATGTTAAAGCGATGACCCTGGAATACTGCATGCAGAGCCATAACAAGTACTGGT

15RC TCATAGATGTTAAAGCGATGACCTTGGAATACTGCATGCAGAGCCATAACAAGTACTGGT

2RC TCATAGATGTTAAAGCGATGACCTTGGAATACTGCATGCAGAGCCATAACAAGTACTGGT

10 TCGATGAGGTTGAATCGTCGATCTTGAACTACCAAGTGTAGTACCATATAGATGATAGGG

17 TCGACGAGGTTGAATCGTCGATCTTGAACTACCAAGTGTAGTACCATATAGATGATAGGG

14 TCGACGAGGTTGAATCGTCGATCTTGAACTACCAAGTGTAGTACCATATAGATGATAGGG

13 TCGACGAGGTTGAATCGTCGATCTTGAACTACCAAGTGTAGTACCATATAGATGATAGGG

12 TCGACGAGGTTGAATCGTCGATCTTGAACTACCAAGTGTAGTACCATATAGATGATAGGG

8 TCGACGAGGTTGAATCGTCGATCTTGAACTACCAAGTGTAGTACCATATAGATGATAGGG

7 TCGACGAGGTTGAATCGTCGATCTTGAACTACCAAGTGTAGTACCATATAGATGATAGGG

19 TCGACGAGGTTGAATCGTCGATCTTGAACTACCAAGTGTAGTACCATATAGATGATAGGG

18 TCGACGAGGTTGAATCGTCGATCTTGAACTACCAAGTGTAGTACCATATAGATGATAGGG

22 TCGATGAGGTTGAATCGTCGATCTTGAACTACCAAGTGTAGTACCATATAGATGATAGGG

21 TCGATGAGGTTGAATCGTCGATCTTGAACTACCAAGTGTAGTACCATATAGATGATAGGG

9 TCGATGAGGTTGAATCGTCGATCTTGAACTACCAAGTGTAGTACCATATAGATGATAGGG

6 TCGATGAGGTTGAATCGTCGATCTTGAACTACCAAGTGTAGTACCATATAGATGATAGGG

5 TCGATGAGGTTGAATCGTCGATCTTGAACTACCAAGTGTAGTACCATATAGATGATAGGG

4 TCGATGAGGTTGAATCGTCGATCTTGAACTACCAAGTGTAGTACCATATAGATGATAGGG

3 TCGATGAGGTTGAATCGTCGATCTTGAACTACCAAGTGTAGTACCATATAGATGATAGGG

20 TCGATGAGGTTGAATCGTCGATCTTGAACTACCAAGTGTAGTACCATATAGATGATAGGG

**. ** ***.** ** .**.*.**.* ***.. .**.** .***** .* *. **

**<<<<<<<<<<<<<<<<<<<<<<<<<<<<<<<<<<<<<<<<<<<<<<<<<<<<<<<<<<<<**

1RC AAAGCAAGACATAAAACCAAATCAAAAATGAGACGATTCTTCTTCTCTTTTGCGGTAGCT

16RC AAAGCAAGACATAAAACCAAATCAAAAATGAGACGATTCTTCTTCTCTTTTGCGGTAGCT

15RC AAAGCAAGACATAAAACCAAATCAAAAATGAGACGATTCTTCTTCTCTTTTGCGGTAGCT

2RC AAAGCAAGACATAAAACCAAATCAAAAATGAGACGATTCTTCTTCTCTTTTGCGGTAGCT

10 ACAAGAACACCAAACGCTAGTTCGATCAAGAGCTTTTTTTGCCTGTCCTTCCTGCTTGTA

17 ACAAGAACACCAAACGCTAGTTCGATCAAGAGCTTTTTTTGCCTGTCCTTCCTGCTTGTA

14 ACAAGAACACCAAACGCTAGTTCGATCAAGAGCTTTTTTTGCCTGTCCTTCCTGCTTGTA

13 ACAAGAACACCAAACGCTAGTTCGATCAAGAGCTTTTTTTGCCTGTCCTTCCTGCTTGTA

12 ACAAGAACACCAAACGCTAGTTCGATCAAGAGCTTTTTTTGCCTGTCCTTCCTGCTTGTA

8 ACAAGAACACCAAACGCTAGTTCGATCAAGAGCTTTTTTTGCCTGTCCTTCCTGCTTGTA

7 ACAAGAACACCAAACGCTAGTTCGATCAAGAGCTTTTTTTGCCTGTCCTTCCTGCTTGTA

19 ACAAGAACACCAAACGCTAGTTCGATCAAGAGCTTTTTTTGCCTGTCCTTCCTGCTTGTA

18 ACAAGAACACCAAACGCTAGTTCGATCAAGAGCTTTTTTTGCCTGTCCTTCCTGCTTGTA

22 ACAAGAACACCAAACGCTAGTTCGATCAAGAGCTTTTTTTGCCTGTCCTTCCTGCTTGTA

21 ACAAGAACACCAAACGCTAGTTCGATCAAGAGCTTTTTTTGCCTGTCCTTCCTGCTTGTA

9 ACAAGAACACCAAACGCTAGTTCGATCAAGAGCTTTTTTTGCCTGTCCTTCCTGCTTGTA

6 ACAAGAACACCAAACGCTAGTTCGATCAAGAGCTTTTTTTGCCTGTCCTTCCTGCTTGTA

5 ACAAGAACACCAAACGCTAGTTCGATCAAGAGCTTTTTTTGCCTGTCCTTCCTGCTTGTA

4 ACAAGAACACCAAACGCTAGTTCGATCAAGAGCTTTTTTTGCCTGTCCTTCCTGCTTGTA

3 ACAAGAACACCAAACGCTAGTTCGATCAAGAGCTTTTTTTGCCTGTCCTTCCTGCTTGTA

20 ACAAGAACACCAAACGCTAGTTCGATCAAGAGCTTTTTTTGCCTGTCCTTCCTGCTTGTA

* *. ** ** ** .*.*. **.* * *** . **.* *.* **.**. .* * *.

**<<<<<<<<<<<<<<<<<<<<<<<<<<<<<<<<<<<<<<<<<<<<<<<<<<<<<<<<<<<<**

1RC CGAACCACATCAGAAGAAGACACGTTAGCAAGGAAACGGTTGATGCAAAGACATGCTGCA

16RC CGAACCACATCAGAAGAAGACACGTTAGCAAGGAAACGGTTGATGCAAAGACATGCTGCA

15RC CGAACCACATCAGAAGAAGACACGTTAGCAAGGAAACGGTTGATGCAAAGACATGCTGCA

2RC CGAACCACATCAGAAGAAGACACGTTAGCAAGGAAACGGTTGATGCAAAGACATGCTGCA

10 TTAACTACAGATGCTGTTGCTATGTTTGACAGGTTGCGCAAAATACATGCTGCTGTGGTC

17 TTAACTACAGATGCTGTTGCTATGTTTGACAGGTTGCGCAAAATACATGCTGCTGTGGTC

14 TTAACTACAGATGCTGTTGCTATGTTTGACAGGTTGCGCAAAATACATGCTGCTGTGGTC

13 TTAACTACAGATGCTGTTGCTATGTTTGACAGGTTGCGCAAAATACATGCTGCTGTGGTC

12 TTAACTACAGATGCTGTTGCTATGTTTGACAGGTTGCGCAAAATACATGCTGCTGTGGTC

8 TTAACTACAGATGCTGTTGCTATGTTTGACAGGTTGCGCAAAATACATGCTGCTGTGGTC

7 TTAACTACAGATGCTGTTGCTATGTTTGACAGGTTGCGCAAAATACATGCTGCTGTGGTC

19 TTAACTACAGATGCTGTTGCTATGTTTGACAGGTTGCGCAAAATACATGCTGCTGTGGTC

18 TTAACTACAGATGCTGTTGCTATGTTTGACAGGTTGCGCAAAATACATGCTGCTGTGGTC

22 TTAACTACAGATGCTGTTGCTATGTTTGACAGGTTGCGCAAAATACATGCTGCTGTGGTC

21 TTAACTACAGATGCTGTTGCTATGTTTGACAGGTTGCGCAAAATACATGCTGCTGTGGTC

9 TTAACTACAGATGCTGTTGCTATGTTTGACAGGTTGCGCAAAATACATGCTGCTGTGGTC

6 TTAACTACAGATGCTGTTGCTATGTTTGACAGGTTGCGCAAAATACATGCTGCTGTGGTC

5 TTAACTACAGATGCTGTTGCTATGTTTGACAGGTTGCGCAAAATACATGCTGCTGTGGTC

4 TTAACTACAGATGCTGTTGCTATGTTTGACAGGTTGCGCAAAATACATGCTGCTGTGGTC

3 TTAACTACAGATGCTGTTGCTATGTTTGACAGGTTGCGCAAAATACATGCTGCTGTGGTC

20 TTAACTACAGATGCTGTTGCTATGTTTGACAGGTTGCGCAAAATACATGCTGCTGTGGTC

. ***.*** * * * .*.*** * *** .** .**.** . **. *.

**<<<<<<<<<<<<<<<<<<<<<<<<<<<<<<<<<<<<<<<<<<<<<<<<<<<<<<<<<<<<**

1RC GGTAATGCAATAGAGTTTCCTATGTTTATCTTTATTCCTGTAGAAAGTTAACAAAGTCTC

16RC GGTAATGCAATAGAGTTTCCTATGTTTATCTTTATTCCTGTAGAAAGTTAACAAAGTCTC

15RC GGTAATGCAATAGAGTTTCCTATGTTTATCTTTATTCCTGTAGAAAGTTAACAAAGTCTC

2RC GGTAATGCAATAGAGTTTCCTATGTTTATCTTTATTCCTGTAGAAAGTTAACAAAGTCTC

10 GGTATGGCGATATTTGCTCCGATTACCAGCTTTGTTGCTGCTTCGATTAATAATAGTCAC

17 GGTATGGCGATATTTGCTCCGATTATCAGCTTTGTTGCTGCTTCGATTAATAATAGTCAC

14 GGTATGGCGATATTTGCTCCGATTATCAGCTTTGTTGCTGCTTCGATTAATAATAGTCAC

13 GGTATGGCGATATTTGCTCCGATTATCAGCTTTGTTGCTGCTTCGATTAATAATAGTCAC

12 GGTATGGCGATATTTGCTCCGATTATCAGCTTTGTTGCTGCTTCGATTAATAATAGTCAC

8 GGTATGGCGATATTTGCTCCGATTATCAGCTTTGTTGCTGCTTCGATTAATAATAGTCAC

7 GGTATGGCGATATTTGCTCCGATTATCAGCTTTGTTGCTGCTTCGATTAATAATAGTCAC

19 GGTATGGCGATATTTGCTCCGATTATCAGCTTTGTTGCTGCTTCGATTAATAATAGTCAC

18 GGTATGGCGATATTTGCTCCGATTATCAGCTTTGTTGCTGCTTCGATTAATAATAGTCAC

22 GGTATGGCGATATTTGCTCCGATTACCAGCTTTGTTGCTGCTTCGATTAATAATAGTCAC

21 GGTATGGCGATATTTGCTCCGATTATCAGCTTTGTTGCTGCTTCGATTAATAATAGTCAC

9 GGTATGGCGATATTTGCTCCGATTATCAGCTTTGTTGCTGCTTCGATTAATAATAGTCAC

6 GGTATGGCGATATTTGCTCCGATTATCAGCTTTGTTGCTGCTTCGATTAATAATAGTCAC

5 GGTATGGCGATATTTGCTCCGATTATCAGCTTTGTTGCTGCTTCGATTAATAATAGTCAC

4 GGTATGGCGATATTTGCTCCGATTATCAGCTTTGTTGCTGCTTCGATTAATAATAGTCAC

3 GGTATGGCGATATTTGCTCCGATTATCAGCTTTGTTGCTGCTTCGATTAATAATAGTCAC

20 GGTATGGCGATATTTGCTCCGATTATCAGCTTTGTTGCTGCTTCGATTAATAATAGTCAC

**** **.*** .*** ** ..* ****.** ***. .* * * * **** *

**<<<<<<<<<<<<<<<<<<<<<<<<<<<<<<<<<<<<<<<<<<<<<<<<<<<<<<<<<<<<**

1RC AATTAGGCTGAGCCGACTCACCTATATCGCACCACACGGGAATCTTGATATCCACGTTGC

16RC AATTAGGCTGAGCCGACTCACCTATATCGCACCACACGGGAATCTTGATATCCACGTTGC

15RC AATTAGGCTGAGCCGACTCACCTATATCGCACCACACGGGAATCTTGATATCCACGTTGC

2RC AATTAGGCTGAGCCGACTCACCTATATCGCACCACACGGGAATCTTGATATCCACGTTGC

10 TGTTATTCCGTCTGTACTCACTAATGTCACACCAAACTGGAGTACGAATGTCAACATTAT

17 TGTTATTCCGTCTGTACTCACTAATGTCACACCAAACTGGAGTACGAATGTCAACATTAT

14 TGTTATTCCGTCTGTACTCACTAATGTCACACCAAACTGGAGTACGAATGTCAACATTAT

13 TGTTATTCCGTCTGTACTCACTAATGTCACACCAAACTGGAGTACGAATGTCAACATTAT

12 TGTTATTCCGTCTGTACTCACTAATGTCACACCAAACTGGAGTACGAATGTCAACATTAT

8 TGTTATTCCGTCTGTACTCACTAATGTCACACCAAACTGGAGTACGAATGTCAACATTAT

7 TGTTATTCCGTCTGTACTCACTAATGTCACACCAAACTGGAGTACGAATGTCAACATTAT

19 TGTTATTCCGTCTGTACTCACTAATGTCACACCAAACTGGAGTACGAATGTCAACATTAT

18 TGTTATTCCGTCTGTACTCACTAATGTCACACCAAACTGGAGTACGAATGTCAACATTAT

22 TGTTATTCCGTCTGTACTCACTAATGTCACACCAAACTGGAGTACGAATGTCAACATTAT

21 TGTTATTCCGTCTGTACTCACTAATGTCACACCAAACTGGAGTACGAATGTCAACATTAT

9 TGTTATTCCGTCTGTACTCACTAATGTCACACCAAACTGGAGTACGAATGTCAACATTAT

6 TGTTATTCCGTCTGTACTCACTAATGTCACACCAAACTGGAGTACGAATGTCAACATTAT

5 TGTTATTCCGTCTGTACTCACTAATGTCACACCAAACTGGAGTACGAATGTCAACATTAT

4 TGTTATTCCGTCTGTACTCACTAATGTCACACCAAACTGGAGTACGAATGTCAACATTAT

3 TGTTATTCCGTCTGTACTCACTAATGTCACACCAAACTGGAGTACGAATGTCAACATTAT

20 TGTTATTCCGTCTGTACTCACTAATGTCACACCAAACTGGAGTACGAATGTCAACATTAT

.*** *.* . ******. **.**.***** ** ***.* . .**.** **.**..

**<<<<<<<<<<<<<<<<<<<<<<<<<<<<<<<<<<<<<<<<<<<<<<<<<<<<<<<<<<<<**

1RC CTCTCCAGACAAAGCTGTTGACTGTTTGAAATAAATTGACCAAAAAGAGCCAAACCACTA

16RC CTCTCCAGACAAAGCTGTTGACTGTTTGAAATAAATTGACCAAAAAGAGCCAAACCACTA

15RC CTCTCCAGACAAAGCTGTTGACTGTTTGAAATAAATTGACCAAAAAGAGCCAAACCACTA

2RC CTCTCCAGACAAAGCTGTTGACTGTTTGAAATAAATTGACCAAAAAGAGCCAAACCACTA

10 CGTGCCAAACGATTGAGTTGATAAAATAAGTGAAGTTTACAATTGCAAGCCAAGCAATAA

17 CGTGCCAAACGATTGAGTTGATAAAATAAGTGAAGTTTACAATTGCAAGCCAAGCAATAA

14 CGTGCCAAACGATTGAGTTGATAAAATAAGTGAAGTTTACAATTGCAAGCCAAGCAATAA

13 CGTGCCAAACGATTGAGTTGATAAAATAAGTGAAGTTTACAATTGCAAGCCAAGCAATAA

12 CGTGCCAAACGATTGAGTTGATAAAATAAGTGAAGTTTACAATTGCAAGCCAAGCAATAA

8 CGTGCCAAACGATTGAGTTGATAAAATAAGTGAAGTTTACAATTGCAAGCCAAGCAATAA

7 CGTGCCAAACGATTGAGTTGATAAAATAAGTGAAGTTTACAATTGCAAGCCAAGCAATAA

19 CGTGCCAAACGATTGAGTTGATAAAATAAGTGAAGTTTACAATTGCAAGCCAAGCAATAA

18 CGTGCCAAACGATTGAGTTGATAAAATAAGTGAAGTTTACAATTGCAAGCCAAGCAATAA

22 CGTGCCAAACGATTGAGTTGATAAAATAAGTGAAGTTTACAATTGCAAGCCAAGCAATAA

21 CGTGCCAAACGATTGAGTTGATAAAATAAGTGAAGTTTACAATTGCAAGCCAAGCAATAA

9 CGTGCCAAACGATTGAGTTGATAAAATAAGTGAAGTTTACAATTGCAAGCCAAGCAATAA

6 CGTGCCAAACGATTGAGTTGATAAAATAAGTGAAGTTTACAATTGCAAGCCAAGCAATAA

5 CGTGCCAAACGATTGAGTTGATAAAATAAGTGAAGTTTACAATTGCAAGCCAAGCAATAA

4 CGTGCCAAACGATTGAGTTGATAAAATAAGTGAAGTTTACAATTGCAAGCCAAGCAATAA

3 CGTGCCAAACGATTGAGTTGATAAAATAAGTGAAGTTTACAATTGCAAGCCAAGCAATAA

20 CGTGCCAAACGATTGAGTTGATAAAATAAGTGAAGTTTACAATTGCAAGCCAAGCAATAA

* . ***.**.* *****. . *.*. **.** ** * . .******.* *. *

**<<<<<<<<<<<<<<<<<<<<<<<<<<<<<<<<<<<<<<<<<<<<<<<<<<<<<<<<<<<<**

1RC ATGCGACAGCCGAATGATTTTGAGCTTTGATGTGAAAAGGAGCTGGTAGTAGTACTAAGA

16RC ATGCGACAGCCGAATGATTTTGAGCTTTGATGTGAAAAGGAGCTGGTAGTAGTACTAAGA

15RC ATGCGACAGCCGAATGATTTTGAGCTTTGATGTGAAAAGGAGCTGGTAGTAGTACTAAGA

2RC ATGCGACAGCCGAATGATTTTGAGCTTTGATGTGAAAAGGAGCTGGTAGTAGTACTAAGA

10 GAATAAGAACACTGGTATTGCGTACACGGTAGTGCCATGCGCAAGGCAATATGGTGATGA

17 GAATAAGAACACTGGTATTGCGTACACGGTAGTGCCATGCGCAAGGCAATATGGTGATGA

14 GAATAAGAACACTGGTATTGCGTACACGGTAGTGCCATGCGCAAGGCAATATGGTGATGA

13 GAATAAGAACACTGGTATTGCGTACACGGTAGTGCCATGCGCAAGGCAATATGGTGATGA

12 GAATAAGAACACTGGTATTGCGTACACGGTAGTGCCATGCGCAAGGCAATATGGTGATGA

8 GAATAAGAACACTGGTATTGCGTACACGGTAGTGCCATGCGCAAGGCAATATGGTGATGA

7 GAATAAGAACACTGGTATTGCGTACACGGTAGTGCCATGCGCAAGGCAATATGGTGATGA

19 GAATAAGAACACTGGTATTGCGTACACGGTAGTGCCATGCGCAAGGCAATATGGTGATGA

18 GAATAAGAACACTGGTATTGCGTACACGGTAGTGCCATGCGCAAGGCAATATGGTGATGA

22 GAATAAGAACACTGGTATTGCGTACACGGTAGTGCCATGCGCAAGGCAATATGGTGATGA

21 GAATAAGAACACTGGTATTGCGTACACGGTAGTGCCATGCGCAAGGCAATATGGTGATGA

9 GAATAAGAACACTGGTATTGCGTACACGGTAGTGCCATGCGCAAGGCAATATGGTGATGA

6 GAATAAGAACACTGGTATTGCGTACACGGTAGTGCCATGCGCAAGGCAATATGGTGATGA

5 GAATAAGAACACTGGTATTGCGTACACGGTAGTGCCATGCGCAAGGCAATATGGTGATGA

4 GAATAAGAACACTGGTATTGCGTACACGGTAGTGCCATGCGCAAGGCAATATGGTGATGA

3 GAATAAGAACACTGGTATTGCGTACACGGTAGTGCCATGCGCAAGGCAATATGGTGATGA

20 GAATAAGAACACTGGTATTGCGTACACGGTAGTGCCATGCGCAAGGCAATATGGTGATGA

. ...* *.* . *** .* .* . * *** * * . **.*.** .. * **

**<<<<<<<<<<<<<<<<<STE3-approximate**

1RC CTAGAGCCAATACAG-----------AGAGCAACTCGAAAAGCCAGGATGGAAACATTCT

16RC CTAGAGCCAATACAG-----------AGAGCAACTCGAAAAGCCAGGATGGAAACATTCT

15RC CTAGAGCCAATACAG-----------AGAGCAACTCGAAAAGCCAGGATGGAAACATTCT

2RC CTAGAGCCAATACAG-----------AGAGCAACTCGAAAAGCCAGGATGGAAACATTCT

10 TCACAGCGAGTATTGAAATGATTGGTAGGGCCACCAAAGAAGACATGATAG-------TC

17 TCACAGCGAGTATTGAAATGATTGGTAGGGCCACCAAAGAAGACATGATAG-------TC

14 TCACAGCGAGTATTGAAATGATTGGTAGGGCCACCAAAGAAGACATGATAG-------TC

13 TCACAGCGAGTATTGAAATGATTGGTAGGGCCACCAAAGAAGACATGATAG-------TC

12 TCACAGCGAGTATTGAAATGATTGGTAGGGCCACCAAAGAAGACATGATAG-------TC

8 TCACAGCGAGTATTGAAATGATTGGTAGGGCCACCAAAGAAGACATGATAG-------TC

7 TCACAGCGAGTATTGAAATGATTGGTAGGGCCACCAAAGAAGACATGATAG-------TC

19 TCACAGCGAGTATTGAAATGATTGGTAGGGCCACCAAAGAAGACATGATAG-------TC

18 TCACAGCGAGTATTGAAATGATTGGTAGGGCCACCAAAGAAGACATGATAG-------TC

22 TCACAGCGAGTATTGAAATGATTGGTAGGGCCACCAAAGAAGACATGATAG-------TC

21 TCACAGCGAGTATTGAAATGATTGGTAGGGCCACCAAAGAAGACATGATAG-------TC

9 TCACAGCGAGTATTGAAATGATTGGTAGGGCCACCAAAGAAGACATGATAG-------TC

6 TCACAGCGAGTATTGAAATGATTGGTAGGGCCACCAAAGAAGACATGATAG-------TC

5 TCACAGCGAGTATTGAAATGATTGGTAGGGCCACCAAAGAAGACATGATAG-------TC

4 TCACAGCGAGTATTGAAATGATTGGTAGGGCCACCAAAGAAGACATGATAG-------TC

3 TCACAGCGAGTATTGAAATGATTGGTAGGGCCACCAAAGAAGACATGATAG-------TC

20 TCACAGCGAGTATTGAAATGATTGGTAGGGCCACCAAAGAAGACATGATAG-------TC

..* *** *.**. * **.** **. .*.*** ** ***.* ..

1RC TTTCAAATTAAAC--CTGATAACAATTAGTGTAAAAGTTGTCTGTTCTCATCTT------

16RC TTTCAAATTAAAC--CTGATAACAATTAGTGTAAAAGTTGTCTGTTCTCATCTT------

15RC TTTCAAATCAAAC--CTGATAACAATTAGTGTAAAAGTTGTCTGTTCTCATCTT------

2RC TTTCAAATCAAAC--CTGATAACAATTAGTGTAAAAGTTGTCTGTTCTCATCTT------

10 GTTTAGATTATGCAACTAATATCGCTTAATCGCTTTATCATTCGTTCCCCTTCTCCGTTC

17 GTTTAGATTATGCAACTAATATCGCTTAATCGCTTTATCATTCGTTCCCTTTCTCCGTTC

14 GTTTAGATTATGCAACTAATATCGCTTAATCGCTTTATCATTCGTTCCCTTTCTCCGTTC

13 GTTTAGATTATGCAACTAATATCGCTTAATCGCTTTATCATTCGTTCCCTTTCTCCGTTC

12 GTTTAGATTATGCAACTAATATCGCTTAATCGCTTTATCATTCGTTCCCTTTCTCCGTTC

8 GTTTAGATTATGCAACTAATATCGCTTAATCGCTTTATCATTCGTTCCCTTTCTCCGTTC

7 GTTTAGATTATGCAACTAATATCGCTTAATCGCTTTATCATTCGTTCCCTTTCTCCGTTC

19 GTTTAGATTATGCAACTAATATCGCTTAATCGCTTTATCATTCGTTCCCTTTCTCCGTTC

18 GTTTAGATTATGCAACTAATATCGCTTAATCGCTTTATCATTCGTTCCCTTTCTCCGTTC

22 GTTTAGATTATGCAACTAATATCGCTTAATCGCTTTATCATTCGTTCCCCTTCTCCGTTC

21 GTTTAGATTATGCAACTAATATCGCTTAATCGCTTTATCATTCGTTCCCTTTCTCCGTTC

9 GTTTAGATTATGCAACTAATATCGCTTAATCGCTTTATCATTCGTTCCCTTTCTCCGTTC

6 GTTTAGATTATGCAACTAATATCGCTTAATCGCTTTATCATTCGTTCCCTTTCTCCGTTC

5 GTTTAGATTATGCAACTAATATCGCTTAATCGCTTTATCATTCGTTCCCTTTCTCCGTTC

4 GTTTAGATTATGCAACTAATATCGCTTAATCGCTTTATCATTCGTTCCCTTTCTCCGTTC

3 GTTTAGATTATGCAACTAATATCGCTTAATCGCTTTATCATTCGTTCCCTTTCTCCGTTC

20 GTTTAGATTATGCAACTAATATCGCTTAATCGCTTTATCATTCGTTCCCTTTCTCCGTTC

**.*.**.* .* **.*** *. ***.* .*..*..****.* *..*

1RC -------------------------CATTAATTATATTCTGAGAACAATGGAGACACCT-

16RC -------------------------CATTAATTATATTCTGAGAACAATGGAGACACCT-

15RC -------------------------CATTAATTATATTCTGAGAACAATGGAGACACCT-

2RC -------------------------CATTAATTATATTCTGAGAACAATGGAGACACCT-

10 ATTGTCTCAATTAATTTACATCGCACAATGATTTTAATGCAGGAACGATCAAGTCATGCG

17 ATTGTCTCAATTAATTTACATCGCACAATGATTTTAATGCAGGAACGATCAAGTCATGCG

14 ATTGTCTCAATTAATTTACATCGCACAATGATTTTAATGCAGGAACGATCAAGTCATGCG

13 ATTGTCTCAATTAATTTACATCGCACAATGATTTTAATGCAGGAACGATCAAGTCATGCG

12 ATTGTCTCAATTAATTTACATCGCACAATGATTTTAATGCAGGAACGATCAAGTCATGCG

8 ATTGTCTCAATTAATTTACATCGCACAATGATTTTAATGCAGGAACGATCAAGTCATGCG

7 ATTGTCTCAATTAATTTACATCGCACAATGATTTTAATGCAGGAACGATCAAGTCATGCG

19 ATTGTCTCAATTAATTTACATCGCACAATGATTTTAATGCAGGAACGATCAAGTCATGCG

18 ATTGTCTCAATTAATTTACATCGCACAATGATTTTAATGCAGGAACGATCAAGTCATGCG

22 ATTGTCTCAATTAATTTACATCGCACAATGATTTTAATGCAGGAACGATCAAGTCATGCG

21 ATTGTCTCAATTAATTTACATCGCACAATGATTTTAATGCAGGAACGATCAAGTCATGCG

9 ATTGTCTCAATTAATTTACATCGCACAATGATTTTAATGCAGGAACGATCAAGTCATGCG

6 ATTGTCTCAATTAATTTACATCGCACAATGATTTTAATGCAGGAACGATCAAGTCATGCG

5 ATTGTCTCAATTAATTTACATCGCACAATGATTTTAATGCAGGAACGATCAAGTCATGCG

4 ATTGTCTCAATTAATTTACATCGCACAATGATTTTAATGCAGGAACGATCAAGTCATGCG

3 ATTGTCTCAATTAATTTACATCGCACAATGATTTTAATGCAGGAACGATCAAGTCATGCG

20 ATTGTCTCAATTAATTTACATCGCACAATGATTTTAATGCAGGAACGATCAAGTCATGCG

** *.*** ** * ...****.** .** **. .

1RC ---------------------TATCTCATCGT----------------------------

16RC ---------------------TATCTCATCGT----------------------------

15RC ---------------------TATCTCATCGT----------------------------

2RC ---------------------TATCTCATCGT----------------------------

10 TAAAAAGAAAAAGAAAATGGAAGCCTCATTGTCCTTCTGATGACAGAGTTTTGATCAAAC

17 TAAAAAGAAAAAGAAAATGGAAGCCTCATTGTCCTTCTGATGACAGAGTTTTGATCAAAC

14 TAAAAAGAAAAAGAAAATGGAAGCCTCATTGTCCTTCTGATGACAGAGTTTTGATCAAAC

13 TAAAAAGAAAAAGAAAATGGAAGCCTCATTGTCCTTCTGATGACAGAGTTTTGATCAAAC

12 TAAAAAGAAAAAGAAAATGGAAGCCTCATTGTCCTTCTGATGACAGAGTTTTGATCAAAC

8 TAAAAAGAAAAAGAAAATGGAAGCCTCATTGTCCTTCTGATGACAGAGTTTTGATCAAAC

7 TAAAAAGAAAAAGAAAATGGAAGCCTCATTGTCCTTCTGATGACAGAGTTTTGATCAAAC

19 TAAAAAGAAAAAGAAAATGGAAGCCTCATTGTCCTTCTGATGACAGAGTTTTGATCAAAC

18 TAAAAAGAAAAAGAAAATGGAAGCCTCATTGTCCTTCTGATGACAGAGTTTTGATCAAAC

22 TAAAAAGAAAAAGAAAAAGGAAGCCTCATTGTCCTTCTGATGACAGAGTTTTGATCAAAC

21 TAAAAAGAAAAAGAAAATGGAAGCCTCATTGTCCTTCTGATGACAGAGTTTTGATCAAAC

9 TAAAAAGAAAAAGAAAATGGAAGCCTCATTGTCCTTCTGATGACAGAGTTTTGATCAAAC

6 TAAAAAGAAAAAGAAAATGGAAGCCTCATTGTCCTTCTGATGACAGAGTTTTGATCAAAC

5 TAAAAAGAAAAAGAAAATGGAAGCCTCATTGTCCTTCTGATGACAGAGTTTTGATCAAAC

4 TAAAAAGAAAAAGAAAATGGAAGCCTCATTGTCCTTCTGATGACAGAGTTTTGATCAAAC

3 TAAAAAGAAAAAGAAAATGGAAGCCTCATTGTCCTTCTGATGACAGAGTTTTGATCAAAC

20 TAAAAAGAAAAAGAAAATGGAAGCCTCATTGTCCTTCTGATGACAGAGTTTTGATCAAAC

..*****.**

1RC -----------------------GCATGAATTTGAAAATCGAGCTTGAATATTTCAACCG

16RC -----------------------GCATGAATTTGAAAATCGAGCTTGAATATTTCAACCG

15RC -----------------------GCATGAATTTGAAAATCGAGCTTGAATATTTCAACCG

2RC -----------------------GCATGAATTTGAAAATCGAGCTTGAATATTTCAACCG

10 GAGAACAGTGAAAGATAAGCGCAATAAGGTTCAGACAATGATATTTAGAGATTATAATTA

17 GAGAACAGTGAAAGATAAGCGCAATAAGGTTCAGACAATGATATTTAGAGATTATAATTA

14 GAGAACAGTGAAAGATAAGCGCAATAAGGTTCAGACAATGATATTTAGAGATTATAATTA

13 GAGAACAGTGAAAGATAAGCGCAATAAGGTTCAGACAATGATATTTAGAGATTATAATTA

12 GAGAACAGTGAAAGATAAGCGCAATAAGGTTCAGACAATGATATTTAGAGATTATAATTA

8 GAGAACAGTGAAAGATAAGCGCAATAAGGTTCAGACAATGATATTTAGAGATTATAATTA

7 GAGAACAGTGAAAGATAAGCGCAATAAGGTTCAGACAATGATATTTAGAGATTATAATTA

19 GAGAACAGTGAAAGATAAGCGCAATAAGGTTCAGACAATGATATTTAGAGATTATAATTA

18 GAGAACAGTGAAAGATAAGCGCAATAAGGTTCAGACAATGATATTTAGAGATTATAATTA

22 GAGAACAGTGAAAGATAAGCGCAATAAGGTTCAGACAATGATATTTAGAGATTATAATTA

21 GAGAACAGTGAAAGATAAGCGCAATAAGGTTCAGACAATGATATTTAGAGATTATAATTA

9 GAGAACAGTGAAAGATAAGCGCAATAAGGTTCAGACAATGATATTTAGAGATTATAATTA

6 GAGAACAGTGAAAGATAAGCGCAATAAGGTTCAGACAATGATATTTAGAGATTATAATTA

5 GAGAACAGTGAAAGATAAGCGCAATAAGGTTCAGACAATGATATTTAGAGATTATAATTA

4 GAGAACAGTGAAAGATAAGCGCAATAAGGTTCAGACAATGATATTTAGAGATTATAATTA

3 GAGAACAGTGAAAGATAAGCGCAATAAGGTTCAGACAATGATATTTAGAGATTATAATTA

20 GAGAACAGTGAAAGATAAGCGCAATAAGGTTCAGACAATGATATTTAGAGATTATAATTA

..* *. *. ** *** . ..**..* *** .**...

1RC AAAATACTTT----------------------------------------------AAAT

16RC AAAATACTTT----------------------------------------------AAAT

15RC AAAATACTTT----------------------------------------------AAAT

2RC AAAATACTTT----------------------------------------------AAAT

10 AGAGAGCTTCCTCTTGAATTTTCTACATCGTGTCACAAACGTAGCAATGATACCAGAAAT

17 AGAGAGCTTCCTCTTGAATTTTCTACATCGTGTCACAAACGTAGCAATGATACCAGAAAT

14 AGAGAGCTTCCTCTTGAATTTTCTACATCGTGTCACAAACGTAGCAATGATACCAGAAAT

13 AGAGAGCTTCCTCTTGAATTTTCTACATCGTGTCACAAACGTAGCAATGATACCAGAAAT

12 AGAGAGCTTCCTCTTGAATTTTCTACATCGTGTCACAAACGTAGCAATGATACCAGAAAT

8 AGAGAGCTTCCTCTTGAATTTTCTACATCGTGTCACAAACGTAGCAATGATACCAGAAAT

7 AGAGAGCTTCCTCTTGAATTTTCTACATCGTGTCACAAACGTAGCAATGATACCAGAAAT

19 AGAGAGCTTCCTCTTGAATTTTCTACATCGTGTCACAAACGTAGCAATGATACCAGAAAT

18 AGAGAGCTTCCTCTTGAATTTTCTACATCGTGTCACAAACGTAGCAATGATACCAGAAAT

22 AGAGAGCTTCCTCTTGAATTTTCTACATCGTGTCACAAACGTAGCAATGATACCAGAAAT

21 AGAGAGCTTCCTCTTGAATTTTCTACATCGTGTCACAAACGTAGCAATGATACCAGAAAT

9 AGAGAGCTTCCTCTTGAATTTTCTACATCGTGTCACAAACGTAGCAATGATACCAGAAAT

6 AGAGAGCTTCCTCTTGAATTTTCTACATCGTGTCACAAACGTAGCAATGATACCAGAAAT

5 AGAGAGCTTCCTCTTGAATTTTCTACATCGTGTCACAAACGTAGCAATGATACCAGAAAT

4 AGAGAGCTTCCTCTTGAATTTTCTACATCGTGTCACAAACGTAGCAATGATACCAGAAAT

3 AGAGAGCTTCCTCTTGAATTTTCTACATCGTGTCACAAACGTAGCAATGATACCAGAAAT

20 AGAGAGCTTCCTCTTGAATTTTCTACATCGTGTCACAAACGTAGCAATGATACCAGAAAT

*.*. .***. ****

**possible pheromone**

1RC TAAAGATGCATCGATTAAAAT---------------------------------------

16RC TAAAGATGCATCGATTAAAAT---------------------------------------

15RC TAAAGATGCATCGATTAAAAT---------------------------------------

2RC TAAAGATGCATCGATTAAAAT---------------------------------------

10 TGAAGTCTCACAAATCCAAGTTGACCAAGTCCACATCAACGTAGAGAGAGAAGAGCCGGG

17 TGAAGTCTCACAAATCCAAGTTGACCAAGTCCACATCAACGTAGAGAGAGAAGAGCCGGG

14 TGAAGTCTCACAAATCCAAGTTGACCAAGTCCACATCAACGTAGAGAGAGAAGAGCCGGG

13 TGAAGTCTCACAAATCCAAGTTGACCAAGTCCACATCAACGTAGAGAGAGAAGAGCCGGG

12 TGAAGTCTCACAAATCCAAGTTGACCAAGTCCACATCAACGTAGAGAGAGAAGAGCCGGG

8 TGAAGTCTCACAAATCCAAGTTGACCAAGTCCACATCAACGTAGAGAGAGAAGAGCCGGG

7 TGAAGTCTCACAAATCCAAGTTGACCAAGTCCACATCAACGTAGAGAGAGAAGAGCCGGG

19 TGAAGTCTCACAAATCCAAGTTGACCAAGTCCACATCAACGTAGAGAGAGAAGAGCCGGG

18 TGAAGTCTCACAAATCCAAGTTGACCAAGTCCACATCAACGTAGAGAGAGAAGAGCCGGG

22 TGAAGTCTCACAAATCCAAGTTGACCAAGTCCACATCAACGTAGAGAGAGAAGAGCCGGG

21 TGAAGTCTCACAAATCCAAGTTGACCAAGTCCACATCAACGTAGAGAGAGAAGAGCCGGG

9 TGAAGTCTCACAAATCCAAGTTGACCAAGTCCACATCAACGTAGAGAGAGAAGAGCCGGG

6 TGAAGTCTCACAAATCCAAGTTGACCAAGTCCACATCAACGTAGAGAGAGAAGAGCCGGG

5 TGAAGTCTCACAAATCCAAGTTGACCAAGTCCACATCAACGTAGAGAGAGAAGAGCCGGG

4 TGAAGTCTCACAAATCCAAGTTGACCAAGTCCACATCAACGTAGAGAGAGAAGAGCCGGG

3 TGAAGTCTCACAAATCCAAGTTGACCAAGTCCACATCAACGTAGAGAGAGAAGAGCCGGG

20 TGAAGTCTCACAAATCCAAGTTGACCAAGTCCACATCAACGTAGAGAGAGAAGAGCCGGG

*.*** . **. .**. **.*

**precursor MIPEIEVSQIQVDQVHINVEREEPGENETYGSSSGCIIT >>>>>>>>>>**

1RC ------------------------------------------------------------

16RC ------------------------------------------------------------

15RC ------------------------------------------------------------

2RC ------------------------------------------------------------

10 TGAAAATGAGACATATGGTAGCTCATCTGGTTGCATCATTACTTAAGGAAAAGTAAGTGT

17 TGAAAATGAGACATATGGTAGCTCATCTGGTTGCATCATTACTTAAGGAAAAGTAAGTGT

14 TGAAAATGAGACATATGGTAGCTCATCTGGTTGCATCATTACTTAAGGAAAAGTAAGTGT

13 TGAAAATGAGACATATGGTAGCTCATCTGGTTGCATCATTACTTAAGGAAAAGTAAGTGT

12 TGAAAATGAGACATATGGTAGCTCATCTGGTTGCATCATTACTTAAGGAAAAGTAAGTGT

8 TGAAAATGAGACATATGGTAGCTCATCTGGTTGCATCATTACTTAAGGAAAAGTAAGTGT

7 TGAAAATGAGACATATGGTAGCTCATCTGGTTGCATCATTACTTAAGGAAAAGTAAGTGT

19 TGAAAATGAGACATATGGTAGCTCATCTGGTTGCATCATTATTTAAGGAAAAGTAAGTGT

18 TGAAAATGAGACATATGGTAGCTCATCTGGTTGCATCATTATTTAAGGAAAAGTAAGTGT

22 TGAAAATGAGACATATGGTAGCTCATCTGGTTGCATCATTACTTAAGGAAAAGTAAGTGT

21 TGAAAATGAGACATATGGTAGCTCATCTGGTTGCATCATTACTTAAGGAAAAGTAAGTGT

9 TGAAAATGAGACATATGGTAGCTCATCTGGTTGCATCATTACTTAAGGAAAAGTAAGTGT

6 TGAAAATGAGACATATGGTAGCTCATCTGGTTGCATCATTACTTAAGGAAAAGTAAGTGT

5 TGAAAATGAGACATATGGTAGCTCATCTGGTTGCATCATTACTTAAGGAAAAGTAAGTGT

4 TGAAAATGAGACATATGGTAGCTCATCTGGTTGCATCATTACTTAAGGAAAAGTAAGTGT

3 TGAAAATGAGACATATGGTAGCTCATCTGGTTGCATCATTACTTAAGGAAAAGTAAGTGT

20 TGAAAATGAGACATATGGTAGCTCATCTGGTTGCATCATTACTTAAGGAAAAGTAAGTGT

**>>>>>>>>>>>>>>>>>>>>>>>>>>>>>>>>>>>>>>>>>>>Ph?**

1RC ------------------------------------------------------------

16RC ------------------------------------------------------------

15RC ------------------------------------------------------------

2RC ------------------------------------------------------------

10 CTACTAAGAGTCTTTGTGAATCTGACAAAGACTCCACCAGGTCTATCCAATGCAGTCTGC

17 CTACTAAGAGTCTTTGTGAATCTGACAAAGACTCCACCAGGTCTATCCAATGCAGTCTGC

14 CTACTAAGAGTCTTTGTGAATCTGACAAAGACTCCACCAGGTCTATCCAATGCAGTCTGC

13 CTACTAAGAGTCTTTGTGAATCTGACAAAGACTCCACCAGGTCTATCCAATGCAGTCTGC

12 CTACTAAGAGTCTTTGTGAATCTGACAAAGACTCCACCAGGTCTATCCAATGCAGTCTGC

8 CTACTAAGAGTCTTTGTGAATCTGACAAAGACTCCACCAGGTCTATCCAATGCAGTCTGC

7 CTACTAAGAGTCTTTGTGAATCTGACAAAGACTCCACCAGGTCTATCCAATGCAGTCTGC

19 CTACTAAGAGTCTTTGTGAATCTGACAAAGACTCCACCAGGTCTATCCAATGCAGTCTGC

18 CTACTAAGAGTCTTTGTGAATCTGACAAAGACTCCACCAGGTCTATCCAATGCAGTCTGC

22 CTACTAAGAGTCTTTGTGAATCTGACAAAGACTCCACCAGGTCTATCCAATGCAGTCTGC

21 CTACTAAGAGTCTTTGTGAATCTGACAAAGACTCCACCAGGTCTATCCAATGCAGTCTGC

9 CTACTAAGAGTCTTTGTGAATCTGACAAAGACTCCACCAGGTCTATCCAATGCAGTCTGC

6 CTACTAAGAGTCTTTGTGAATCTGACAAAGACTCCACCAGGTCTATCCAATGCAGTCTGC

5 CTACTAAGAGTCTTTGTGAATCTGACAAAGACTCCACCAGGTCTATCCAATGCAGTCTGC

4 CTACTAAGAGTCTTTGTGAATCTGACAAAGACTCCACCAGGTCTATCCAATGCAGTCTGC

3 CTACTAAGAGTCTTTGTGAATCTGACAAAGACTCCACCAGGTCTATCCAATGCAGTCTGC

20 CTACTAAGAGTCTTTGTGAATCTGACAAAGACTCCACCAGGTCTATCCAATGCAGTCTGC

1RC -----------------ACAATATACAA--------------------------------

16RC -----------------ACAATATACAA--------------------------------

15RC -----------------ACAATATACAA--------------------------------

2RC -----------------ACAATATACAA--------------------------------

10 TTTTACTTAGAACTTTGACAATATGTAAAAAGGCAATCAATGATGATAAATATAACTATA

17 TTTTACTTAGAACTTTGACAATATGTAAAAAGGCAATCAATGATGATAAATATAACTATA

14 TTTTACTTAGAACTTTGACAATATGTAAAAAGGCAATCAATGATGATAAATATAACTATA

13 TTTTACTTAGAACTTTGACAATATGTAAAAAGGCAATCAATGATGATAAATATAACTATA

12 TTTTACTTAGAACTTTGACAATATGTAAAAAGGCAATCAATGATGATAAATATAACTATA

8 TTTTACTTAGAACTTTGACAATATGTAAAAAGGCAATCAATGATGATAAATATAACTATA

7 TTTTACTTAGAACTTTGACAATATGTAAAAAGGCAATCAATGATGATAAATATAACTATA

19 TTTTACTTAGAACTTTGACAATATGTAAAAAGGCAATCAATGATGATAAATATAACTATA

18 TTTTACTTAGAACTTTGACAATATGTAAAAAGGCAATCAATGATGATAAATATAACTATA

22 TTTTACTTAGAACTTTGACAATATGTAAAAAGGCAATCAATGATGATAAATATAACTATA

21 TTTTACTTAGAACTTTGACAATATGTAAAAAGGCAATCAATGATGATAAATATAACTATA

9 TTTTACTTAGAACTTTGACAATATGTAAAAAGGCAATCAATGATGATAAATATAACTATA

6 TTTTACTTAGAACTTTGACAATATGTAAAAAGGCAATCAATGATGATAAATATAACTATA

5 TTTTACTTAGAACTTTGACAATATGTAAAAAGGCAATCAATGATGATAAATATAACTATA

4 TTTTACTTAGAACTTTGACAATATGTAAAAAGGCAATCAATGATGATAAATATAACTATA

3 TTTTACTTAGAACTTTGACAATATGTAAAAAGGCAATCAATGATGATAAATATAACTATA

20 TTTTACTTAGAACTTTGACAATATGTAAAAAGGCAATCAATGATGATAAATATAACTATA

*******..**

1RC ------------------------------------------------------------

16RC ------------------------------------------------------------

15RC ------------------------------------------------------------

2RC ------------------------------------------------------------

10 TGTCAATTATCACCTTTCCACAGATCCTATCATTGAGCCTTTTAATATACAAATAAACTA

17 TGTCAATTATCACCTTTCCACAGATCCTATCATTGAGCCTTTTAATATACAAATAAACTA

14 TGTCAATTATCACCTTTCCACAGATCCTATCATTGAGCCTTTTAATATACAAATAAACTA

13 TGTCAATTATCACCTTTCCACAGATCCTATCATTGAGCCTTTTAATATACAAATAAACTA

12 TGTCAATTATCACCTTTCCACAGATCCTATCATTGAGCCTTTTAATATACAAATAAACTA

8 TGTCAATTATCACCTTTCCACAGATCCTATCATTGAGCCTTTTAATATACAAATAAACTA

7 TGTCAATTATCACCTTTCCACAGATCCTATCATTGAGCCTTTTAATATACAAATAAACTA

19 TGTCAATTATCACCTTTCCACAGATCCTATCATTGAGCCTTTTAATATACAAATAAACTA

18 TGTCAATTATCACCTTTCCACAGATCCTATCATTGAGCCTTTTAATATACAAATAAACTA

22 TGTCAATTATCACCTTTCCACAGATCCTATCATTGAGCCTTTTAATATACAAATAAACTA

21 TGTCAATTATCACCTTTCCACAGATCCTATCATTGAGCCTTTTAATATACAAATAAACTA

9 TGTCAATTATCACCTTTCCACAGATCCTATCATTGAGCCTTTTAATATACAAATAAACTA

6 TGTCAATTATCACCTTTCCACAGATCCTATCATTGAGCCTTTTAATATACAAATAAACTA

5 TGTCAATTATCACCTTTCCACAGATCCTATCATTGAGCCTTTTAATATACAAATAAACTA

4 TGTCAATTATCACCTTTCCACAGATCCTATCATTGAGCCTTTTAATATACAAATAAACTA

3 TGTCAATTATCACCTTTCCACAGATCCTATCATTGAGCCTTTTAATATACAAATAAACTA

20 TGTCAATTATCACCTTTCCACAGATCCTATCATTGAGCCTTTTAATATACAAATAAACTA

1RC ------------------------------------------------------------

16RC ------------------------------------------------------------

15RC ------------------------------------------------------------

2RC ------------------------------------------------------------

10 ACACCTTTTGTGTTGTCTGATGGGCGAATATTAAAAAGACAGTCAATGATAAATACAACT

17 ACACCTTTTGTGTTGTCTGATGGGCGAATATTAAAAAGACAGTCAATGATAAATACAACT

14 ACACCTTTTGTGTTGTCTGATGGGCGAATATTAAAAAGACAGTCAATGATAAATACAACT

13 ACACCTTTTGTGTTGTCTGATGGGCGAATATTAAAAAGACAGTCAATGATAAATACAACT

12 ACACCTTTTGTGTTGTCTGATGGGCGAATATTAAAAAGACAGTCAATGATAAATACAACT

8 ACACCTTTTGTGTTGTCTGATGGGCGAATATTAAAAAGACAGTCAATGATAAATACAACT

7 ACACCTTTTGTGTTGTCTGATGGGCGAATATTAAAAAGACAGTCAATGATAAATACAACT

19 ACACCTTTTGTGTTGTCTGATGGGCGAATATTAAAAAGACAGTCAATGATAAATACAACT

18 ACACCTTTTGTGTTGTCTGATGGGCGAATATTAAAAAGACAGTCAATGATAAATACAACT

22 ACACCTTTTGTGTTGTCTGATGGGCGAATATTAAAAAGACAGTCAATGATAAATACAACT

21 ACACCTTTTGTGTTGTCTGATGGGCGAATATTAAAAAGACAGTCAATGATAAATACAACT

9 ACACCTTTTGTGTTGTCTGATGGGCGAATATTAAAAAGACAGTCAATGATAAATACAACT

6 ACACCTTTTGTGTTGTCTGATGGGCGAATATTAAAAAGACAGTCAATGATAAATACAACT

5 ACACCTTTTGTGTTGTCTGATGGGCGAATATTAAAAAGACAGTCAATGATAAATACAACT

4 ACACCTTTTGTGTTGTCTGATGGGCGAATATTAAAAAGACAGTCAATGATAAATACAACT

3 ACACCTTTTGTGTTGTCTGATGGGCGAATATTAAAAAGACAGTCAATGATAAATACAACT

20 ACACCTTTTGTGTTGTCTGATGGGCGAATATTAAAAAGACAGTCAATGATAAATACAACT

1RC --------ATTGTTACCTTTCCCCAAATCCTAAGGTTGAGCGGTTT---TGATAAGGTGC

16RC --------ATTGTTACCTTTCCCCAAATCCTACGGTTGAGCGGTTT---TGATAAGGTGC

15RC --------ATTGTTACCTTTCCCCAAATCCTACGGTTGAGCGGTTT---TGATAAGGTGC

2RC --------ATTGTTACCTTTCCCCAAATCCTACGGTTGAGCGGTTT---TGATAAGGTGC

10 TTACTTCTATCATCACCTTTCCCCAAATCCTACTGTTGAGCGATTTTGATGATAAGGTGC

17 TTACTTCTATCATCACCTTTCCCCAAATCCTACTGTTGAGCGATTTTGATGATAAGGTGC

14 TTACTTCTATCATCACCTTTCCCCAAATCCTACTGTTGAGCGATTTTGATGATAAGGTGC

13 TTACTTCTATCATCACCTTTCCCCAAATCCTACTGTTGAGCGATTTTGATGATAAGGTGC

12 TTACTTCTATCATCACCTTTCCCCAAATCCTACTGTTGAGCGATTTTGATGATAAGGTGC

8 TTACTTCTATCATCACCTTTCCCCAAATCCTACTGTTGAGCGATTTTGATGATAAGGTGC

7 TTACTTCTATCATCACCTTTCCCCAAATCCTACTGTTGAGCGATTTTGATGATAAGGTGC

19 TTACTTCTATCATCACCTTTCCCCAAATCCTACTGTTGAGCGATTTTGATGATAAGGTGC

18 TTACTTCTATCATCACCTTTCCCCAAATCCTACTGTTGAGCGATTTTGATGATAAGGTGC

22 TTACTTCTATCATCACCTTTCCCCAAATCCTACTGTTGAGCGATTTTGATGATAAGGTGC

21 TTACTTCTATCATCACCTTTCCCCAAATCCTACTGTTGAGCGATTTTGATGATAAGGTGC

9 TTACTTCTATCATCACCTTTCCCCAAATCCTACTGTTGAGCGATTTTGATGATAAGGTGC

6 TTACTTCTATCATCACCTTTCCCCAAATCCTACTGTTGAGCGATTTTGATGATAAGGTGC

5 TTACTTCTATCATCACCTTTCCCCAAATCCTACTGTTGAGCGATTTTGATGATAAGGTGC

4 TTACTTCTATCATCACCTTTCCCCAAATCCTACTGTTGAGCGATTTTGATGATAAGGTGC

3 TTACTTCTATCATCACCTTTCCCCAAATCCTACTGTTGAGCGATTTTGATGATAAGGTGC

20 TTACTTCTATCATCACCTTTCCCCAAATCCTACTGTTGAGCGATTTTGATGATAAGGTGC

**..*.****************** ********.*** ***********

**CAF1<<<<<<<<<<<<<<<<<<<<<<<<<<<<<<<<<<<<<<<<<<<<**

1RC TATAGGTTGAGGGCCAACGTAAGGCGTAGGTATAGGATTACTCATGGGGGTGAATGGTGC

16RC TATAGGTTGAGGGCCAACGTAAGGCGTAGGTATAGGATTACTCATGGGGGTGAATGGTGC

15RC TATAGGTTGAGGGCCAACGTAAGGCGTAGGTATAGGATTACTCATGGGGGTGAATGGTGC

2RC TATAGGTTGAGGGCCAACGTAAGGCGTAGGTATAGGATTACTCATGGGGGTGAATGGTGC

10 AATAGGTTGGGGGCCAACGTAAGGCGTAGGTATAGGGTTACTCATAGGAGTGAATGGTGC

17 AATAGGTTGGGGGCCAACGTAAGGCGTAGGTATAGGGTTACTCATAGGAGTGAATGGTGC

14 AATAGGTTGGGGGCCAACGTAAGGCGTAGGTATAGGGTTACTCATAGGAGTGAATGGTGC

13 AATAGGTTGGGGGCCAACGTAAGGCGTAGGTATAGGGTTACTCATAGGAGTGAATGGTGC

12 AATAGGTTGGGGGCCAACGTAAGGCGTAGGTATAGGGTTACTCATAGGAGTGAATGGTGC

8 AATAGGTTGGGGGCCAACGTAAGGCGTAGGTATAGGGTTACTCATAGGAGTGAATGGTGC

7 AATAGGTTGGGGGCCAACGTAAGGCGTAGGTATAGGGTTACTCATAGGAGTGAATGGTGC

19 AATAGGTTGGGGGCCAACGTAAGGCGTAGGTATAGGGTTACTCATAGGAGTGAATGGTGC

18 AATAGGTTGGGGGCCAACGTAAGGCGTAGGTATAGGGTTACTCATAGGAGTGAATGGTGC

22 AATAGGTTGGGGGCCAACGTAAGGCGTAGGTATAGGGTTACTCATAGGAGTGAATGGTGC

21 AATAGGTTGGGGGCCAACGTAAGGCGTAGGTATAGGGTTACTCATAGGAGTGAATGGTGC

9 AATAGGTTGGGGGCCAACGTAAGGCGTAGGTATAGGGTTACTCATAGGAGTGAATGGTGC

6 AATAGGTTGGGGGCCAACGTAAGGCGTAGGTATAGGGTTACTCATAGGAGTGAATGGTGC

5 AATAGGTTGGGGGCCAACGTAAGGCGTAGGTATAGGGTTACTCATAGGAGTGAATGGTGC

4 AATAGGTTGGGGGCCAACGTAAGGCGTAGGTATAGGGTTACTCATAGGAGTGAATGGTGC

3 AATAGGTTGGGGGCCAACGTAAGGCGTAGGTATAGGGTTACTCATAGGAGTGAATGGTGC

20 AATAGGTTGGGGGCCAACGTAAGGCGTAGGTATAGGGTTACTCATAGGAGTGAATGGTGC

********.**************************.********.**.***********

**<<<<<<<<<<<<<<<<<<<<<<<<<<<<<<<<<<<<<<<<<<<<<<<<<<<<<<<<<<<<**

1RC TCCTGGAGTTTGTATAGCACTAGCACCAGCTCCTATGCCGATGCCATACAAATATCCCAT

16RC TCCTGGAGTTTGTATAGCACTAGCACCAGCTCCTATGCCGATGCCATACAAATATCCCAT

15RC TCCTGGAGTTTGTATAGCACTAGCACCAGCTCCTATGCCGATGCCATACAAATATCCCAT

2RC TCCTGGAGTTTGTATAGCACTAGCACCAGCTCCTATGCCGATGCCATACAAATATCCCAT

10 TCCTGGAGTCTGTATAGCACTAGCGCCAGCTCCTATGCCGATGCCATACAAATATCCCAT

17 TCCTGGAGTCTGTATAGCACTAGCGCCAGCTCCTATGCCGATGCCATACAAATATCCCAT

14 TCCTGGAGTCTGTATAGCACTAGCGCCAGCTCCTATGCCGATGCCATACAAATATCCCAT

13 TCCTGGAGTCTGTATAGCACTAGCGCCAGCTCCTATGCCGATGCCATACAAATATCCCAT

12 TCCTGGAGTCTGTATAGCACTAGCGCCAGCTCCTATGCCGATGCCATACAAATATCCCAT

8 TCCTGGAGTCTGTATAGCACTAGCGCCAGCTCCTATGCCGATGCCATACAAATATCCCAT

7 TCCTGGAGTCTGTATAGCACTAGCGCCAGCTCCTATGCCGATGCCATACAAATATCCCAT

19 TCCTGGAGTCTGTATAGCACTAGCGCCAGCTCCTATGCCGATGCCATACAAATATCCCAT

18 TCCTGGAGTCTGTATAGCACTAGCGCCAGCTCCTATGCCGATGCCATACAAATATCCCAT

22 TCCTGGAGTCTGTATAGCACTAGCGCCAGCTCCTATGCCGATGCCATACAAATATCCCAT

21 TCCTGGAGTCTGTATAGCACTAGCGCCAGCTCCTATGCCGATGCCATACAAATATCCCAT

9 TCCTGGAGTCTGTATAGCACTAGCGCCAGCTCCTATGCCGATGCCATACAAATATCCCAT

6 TCCTGGAGTCTGTATAGCACTAGCGCCAGCTCCTATGCCGATGCCATACAAATATCCCAT

5 TCCTGGAGTCTGTATAGCACTAGCGCCAGCTCCTATGCCGATGCCATACAAATATCCCAT

4 TCCTGGAGTCTGTATAGCACTAGCGCCAGCTCCTATGCCGATGCCATACAAATATCCCAT

3 TCCTGGAGTCTGTATAGCACTAGCGCCAGCTCCTATGCCGATGCCATACAAATATCCCAT

20 TCCTGGAGTCTGTATAGCACTAGCGCCAGCTCCTATGCCGATGCCATACAAATATCCCAT

*********.**************.***********************************

**<<<<<<<<<<<<<<<<<<<<<<<<<<<<<<<<<<<<<<<<<<<<<<<<<<<<<<<<<<<<**

1RC ACAATGGAGTCTGTGTTGAGTTAGCCGAGCCTAGAACCAAGCGAAGTACCCCACAATTGT

16RC ACAATGGAGTCTGTGTTGAGTTAGCCGAGCCTAGAACCAAGCGAAGTACCCCACAATTGT

15RC ACAATGGAGTCTGTGTTGAGTTAGCCGAGCCTAGAACCAAGCGAAGTACCCCACAATTGT

2RC ACAATGGAGTCTGTGTTGAGTTAGCCGAGCCTAGAACCAAGCGAAGTACCCCACAATTGT

10 ACAATGAAGTCTGTGTTGAGTTAGCCAAGCCTAGAACCAAGCGAAGTACCCCACAATTGT

17 ACAATGAAGTCTGTGTTGAGTTAGCCAAGCCTAGAACCAAGCGAAGTACCCCACAATTGT

14 ACAATGAAGTCTGTGTTGAGTTAGCCAAGCCTAGAACCAAGCGAAGTACCCCACAATTGT

13 ACAATGAAGTCTGTGTTGAGTTAGCCAAGCCTAGAACCAAGCGAAGTACCCCACAATTGT

12 ACAATGAAGTCTGTGTTGAGTTAGCCAAGCCTAGAACCAAGCGAAGTACCCCACAATTGT

8 ACAATGAAGTCTGTGTTGAGTTAGCCAAGCCTAGAACCAAGCGAAGTACCCCACAATTGT

7 ACAATGAAGTCTGTGTTGAGTTAGCCAAGCCTAGAACCAAGCGAAGTACCCCACAATTGT

19 ACAATGAAGTCTGTGTTGAGTTAGCCAAGCCTAGAACCAAGCGAAGTACCCCACAATTGT

18 ACAATGAAGTCTGTGTTGAGTTAGCCAAGCCTAGAACCAAGCGAAGTACCCCACAATTGT

22 ACAATGAAGTCTGTGTTGAGTTAGCCAAGCCTAGAACCAAGCGAAGTACCCCACAATTGT

21 ACAATGAAGTCTGTGTTGAGTTAGCCAAGCCTAGAACCAAGCGAAGTACCCCACAATTGT

9 ACAATGAAGTCTGTGTTGAGTTAGCCAAGCCTAGAACCAAGCGAAGTACCCCACAATTGT

6 ACAATGAAGTCTGTGTTGAGTTAGCCAAGCCTAGAACCAAGCGAAGTACCCCACAATTGT

5 ACAATGAAGTCTGTGTTGAGTTAGCCAAGCCTAGAACCAAGCGAAGTACCCCACAATTGT

4 ACAATGAAGTCTGTGTTGAGTTAGCCAAGCCTAGAACCAAGCGAAGTACCCCACAATTGT

3 ACAATGAAGTCTGTGTTGAGTTAGCCAAGCCTAGAACCAAGCGAAGTACCCCACAATTGT

20 ACAATGAAGTCTGTGTTGAGTTAGCCAAGCCTAGAACCAAGCGAAGTACCCCACAATTGT

******.*******************.*********************************

**<<<<<<<<<<<<<<<<<<<<<<<<<<<<<<<<<<<<<<<<<<<<<<<<<<<<<<<<<<<<**

1RC CTGTCCCTCTCGGATGCACCATCAAAAAACTTACTGCGCATAGAGAAAAATGAAGCAGAT

16RC CTGTCCCTCTCGGATGCACCATCAAAAAACTTACTGCGCATAGAGAAAAATGAAGCAGAT

15RC CTGTCCCTCTCGGATGCACCATCAAAAAACTTACTGCGCATAGAGAAAAATGAAGCAGAT

2RC CTGTCCCTCTCGGATGCACCATCAAAAAACTTACTGCGCATAGAGAAAAATGAAGCAGAT

10 CTGTCCCTCTCAGATGCACCATCAAAAAACTTACTGCGCATAGAGAAGAATGAAGCAGAT

17 CTGTCCCTCTCAGATGCACCATCAAAAAACTTACTGCGCATAGAGAAGAATGAAGCAGAT

14 CTGTCCCTCTCAGATGCACCATCAAAAAACTTACTGCGCATAGAGAAGAATGAAGCAGAT

13 CTGTCCCTCTCAGATGCACCATCAAAAAACTTACTGCGCATAGAGAAGAATGAAGCAGAT

12 CTGTCCCTCTCAGATGCACCATCAAAAAACTTACTGCGCATAGAGAAGAATGAAGCAGAT

8 CTGTCCCTCTCAGATGCACCATCAAAAAACTTACTGCGCATAGAGAAGAATGAAGCAGAT

7 CTGTCCCTCTCAGATGCACCATCAAAAAACTTACTGCGCATAGAGAAGAATGAAGCAGAT

19 CTGTCCCTCTCAGATGAACCATCAAAAAACTTACTGCGCATAGAGAAGAATGAAGCAGAT

18 CTGTCCCTCTCAGATGAACCATCAAAAAACTTACTGCGCATAGAGAAGAATGAAGCAGAT

22 CTGTCCCTCTCAGATGCACCATCAAAAAACTTACTGCGCATAGAGAAGAATGAAGCAGAT

21 CTGTCCCTCTCAGATGCACCATCAAAAAACTTACTGCGCATAGAGAAGAATGAAGCAGAT

9 CTGTCCCTCTCAGATGCACCATCAAAAAACTTACTGCGCATAGAGAAGAATGAAGCAGAT

6 CTGTCCCTCTCAGATGCACCATCAAAAAACTTACTGCGCATAGAGAAGAATGAAGCAGAT

5 CTGTCCCTCTCAGATGCACCATCAAAAAACTTACTGCGCATAGAGAAGAATGAAGCAGAT

4 CTGTCCCTCTCAGATGCACCATCAAAAAACTTACTGCGCATAGAGAAGAATGAAGCAGAT

3 CTGTCCCTCTCAGATGCACCATCAAAAAACTTACTGCGCATAGAGAAGAATGAAGCAGAT

20 CTGTCCCTCTCAGATGCACCATCAAAAAACTTACTGCGCATAGAGAAGAATGAAGCAGAT

***********.**** ******************************.************

**<<<<<<<<<<<<<<<<<<<<<<<<<<<<<<<<<<<<<<<<<<<<<<<<<<<<<<<<<<<<**

1RC GTGACCAATGAATCTGATCCTGCTTGATGCTGTGGTCCTATCCGCATTACCTGATGAAAG

16RC GTGACCAATGAATCTGATCCTGCTTGATGCTGTGGTCCTATCCGCATTACCTGATGAAAG

15RC GTGACCAATGAATCTGATCCTGCTTGATGCTGTGGTCCTATCCGCATTACCTGATGAAAG

2RC GTGACCAATGAATCTGATCCTGCTTGATGCTGTGGTCCTATCCGCATTACCTGATGAAAG

10 GTGACCAATGAATCTGATCCTGCTTGATGCTGTGGGCCTATCCGCATTACCTGATGAAAG

17 GTGACCAATGAATCTGATCCTGCTTGATGCTGTGGGCCTATCCGCATTACCTGATGAAAG

14 GTGACCAATGAATCTGATCCTGCTTGATGCTGTGGGCCTATCCGCATTACCTGATGAAAG

13 GTGACCAATGAATCTGATCCTGCTTGATGCTGTGGGCCTATCCGCATTACCTGATGAAAG

12 GTGACCAATGAATCTGATCCTGCTTGATGCTGTGGGCCTATCCGCATTACCTGATGAAAG

8 GTGACCAATGAATCTGATCCTGCTTGATGCTGTGGGCCTATCCGCATTACCTGATGAAAG

7 GTGACCAATGAATCTGATCCTGCTTGATGCTGTGGGCCTATCCGCATTACCTGATGAAAG

19 GTGACCAATGAATCTGATCCTGCTTGATGCTGTGGGCCTATCCGCATTACCTGATGAAAG

18 GTGACCAATGAATCTGATCCTGCTTGATGCTGTGGGCCTATCCGCATTACCTGATGAAAG

22 GTGACCAATGAATCTGATCCTGCTTGATGCTGTGGGCCTATCCGCATTACCTGATGAAAG

21 GTGACCAATGAATCTGATCCTGCTTGATGCTGTGGGCCTATCCGCATTACCTGATGAAAG

9 GTGACCAATGAATCTGATCCTGCTTGATGCTGTGGGCCTATCCGCATTACCTGATGAAAG

6 GTGACCAATGAATCTGATCCTGCTTGATGCTGTGGGCCTATCCGCATTACCTGATGAAAG

5 GTGACCAATGAATCTGATCCTGCTTGATGCTGTGGGCCTATCCGCATTACCTGATGAAAG

4 GTGACCAATGAATCTGATCCTGCTTGATGCTGTGGGCCTATCCGCATTACCTGATGAAAG

3 GTGACCAATGAATCTGATCCTGCTTGATGCTGTGGGCCTATCCGCATTACCTGATGAAAG

20 GTGACCAATGAATCTGATCCTGCTTGATGCTGTGGGCCTATCCGCATTACCTGATGAAAG

*********************************** ************************

**<<<<<<<<<<<<<<<<<<<<<<<<<<<<<<<<<<<<<<<<<<<<<<<<<<<<<<<<<<<<**

1RC TCAGCCGATTCGGCATGCCGAAACAGACGTATCTAACCTGCAACTCATCTGCTATATCTT

16RC TCAGCCGATTCGGCATGCCGAAACAGACGTATCTAACCTGCAACTCATCTGCTATATCTT

15RC TCAGCCGATTCGGCATGCCGAAACAGACGTATCTAACCTGCAACTCATCTGCTATATCTT

2RC TCAGCCGATTCGGCATGCCGAAACAGACGTATCTAACCTGCAACTCATCTGCTATATCTT

10 TCAGCCGATTCGGCATGACGAAACAGACGTGTCTAACCTGCAACTCATCTGCTATATCTT

17 TCAGCCGATTCGGCATGACGAAACAGACGTGTCTAACCTGCAACTCATCTGCTATATCTT

14 TCAGCCGATTCGGCATGACGAAACAGACGTGTCTAACCTGCAACTCATCTGCTATATCTT

13 TCAGCCGATTCGGCATGACGAAACAGACGTGTCTAACCTGCAACTCATCTGCTATATCTT

12 TCAGCCGATTCGGCATGACGAAACAGACGTGTCTAACCTGCAACTCATCTGCTATATCTT

8 TCAGCCGATTCGGCATGACGAAACAGACGTGTCTAACCTGCAACTCATCTGCTATATCTT

7 TCAGCCGATTCGGCATGACGAAACAGACGTGTCTAACCTGCAACTCATCTGCTATATCTT

19 TCAGCCGATTCGGCATGACGAAACAGACGTGTCTAACCTGCAACTCATCTGCTATATCTT

18 TCAGCCGATTCGGCATGACGAAACAGACGTGTCTAACCTGCAACTCATCTGCTATATCTT

22 TCAGCCGATTCGGCATGACGAAACAGACGTGTCTAACCTGCAACTCATCTGCTATATCTT

21 TCAGCCGATTCGGCATGACGAAACAGACGTGTCTAACCTGCAACTCATCTGCTATATCTT

9 TCAGCCGATTCGGCATGACGAAACAGACGTGTCTAACCTGCAACTCATCTGCTATATCTT

6 TCAGCCGATTCGGCATGACGAAACAGACGTGTCTAACCTGCAACTCATCTGCTATATCTT

5 TCAGCCGATTCGGCATGACGAAACAGACGTGTCTAACCTGCAACTCATCTGCTATATCTT

4 TCAGCCGATTCGGCATGACGAAACAGACGTGTCTAACCTGCAACTCATCTGCTATATCTT

3 TCAGCCGATTCGGCATGACGAAACAGACGTGTCTAACCTGCAACTCATCTGCTATATCTT

20 TCAGCCGATTCGGCATGACGAAACAGACGTGTCTAACCTGCAACTCATCTGCTATATCTT

***************** ************.*****************************

**<<<<<<<<<<<<<<<<<<<<<<<<<<<<<<<<<<<<<<<<<<<<<<<<<<<<<<<<<<<<**

1RC GTAGACCACCTTTAAGAAGCCTGTTGATCTTCATAACGTACTTGATGTCGAATATGCAGG

16RC GTAGACCACCTTTAAGAAGCCTGTTGATCTTCATAACGTACTTGATGTCGAATATGCAGG

15RC GTAGACCACCTTTAAGAAGCCTGTTGATCTTCATAACGTACTTGATGTCGAATATGCAGG

2RC GTAGACCACCTTTAAGAAGCCTGTTGATCTTCATAACGTACTTGATGTCGAATATGCAGG

10 GTAGACCACCTTTAAGAAGCCTGTTGATCTTCATAACGTACTTGATGTCGAATATGCAGG

17 GTAGACCACCTTTAAGAAGCCTGTTGATCTTCATAACGTACTTGATGTCGAATATGCAGG

14 GTAGACCACCTTTAAGAAGCCTGTTGATCTTCATAACGTACTTGATGTCGAATATGCAGG

13 GTAGACCACCTTTAAGAAGCCTGTTGATCTTCATAACGTACTTGATGTCGAATATGCAGG

12 GTAGACCACCTTTAAGAAGCCTGTTGATCTTCATAACGTACTTGATGTCGAATATGCAGG

8 GTAGACCACCTTTAAGAAGCCTGTTGATCTTCATAACGTACTTGATGTCGAATATGCAGG

7 GTAGACCACCTTTAAGAAGCCTGTTGATCTTCATAACGTACTTGATGTCGAATATGCAGG

19 GTAGACCACCTTTAAGAAGCCTGTTGATCTTCATAACGTACTTGATGTCGAATATGCAGG

18 GTAGACCACCTTTAAGAAGCCTGTTGATCTTCATAACGTACTTGATGTCGAATATGCAGG

22 GTAGACCACCTTTAAGAAGCCTGTTGATCTTCATAACGTACTTGATGTCGAATATGCAGG

21 GTAGACCACCTTTAAGAAGCCTGTTGATCTTCATAACGTACTTGATGTCGAATATGCAGG

9 GTAGACCACCTTTAAGAAGCCTGTTGATCTTCATAACGTACTTGATGTCGAATATGCAGG

6 GTAGACCACCTTTAAGAAGCCTGTTGATCTTCATAACGTACTTGATGTCGAATATGCAGG

5 GTAGACCACCTTTAAGAAGCCTGTTGATCTTCATAACGTACTTGATGTCGAATATGCAGG

4 GTAGACCACCTTTAAGAAGCCTGTTGATCTTCATAACGTACTTGATGTCGAATATGCAGG

3 GTAGACCACCTTTAAGAAGCCTGTTGATCTTCATAACGTACTTGATGTCGAATATGCAGG

20 GTAGACCACCTTTAAGAAGCCTGTTGATCTTCATAACGTACTTGATGTCGAATATGCAGG

************************************************************

**<<<<<<<<<<<<<<<<<<<<<<<<<<<<<<<<<<<<<<<<<<<<<<<<<<<<<<<<<<<<**

1RC GGAACCATATGCGAAGTAGATCAAAGAAATCAGTTTCATTTGAAGGTAGTGGCAGCGCCG

16RC GGAACCATATGCGAAGTAGATCAAAGAAATCAGTTTCATTTGAAGGTAGTGGCAGCGCCG

15RC GGAACCATATGCGAAGTAGATCAAAGAAATCAGTTTCATTTGAAGGTAGTGGCAGCGCCG

2RC GGAACCATATGCGAAGTAGATCAAAGAAATCAGTTTCATTTGAAGGTAGTGGCAGCGCCG

10 GGAACCATATACGAAGTAGATCAAAAAAATCAGTTTCATTTGAAGGTAGTGGCAGCGCCG

17 GGAACCATATGCGAAGTAGATCAAAAAAATCAGTTTCATTTGAAGGTAGTGGCAGCGCCG

14 GGAACCATATGCGAAGTAGATCAAAAAAATCAGTTTCATTTGAAGGTAGTGGCAGCGCCG

13 GGAACCATATGCGAAGTAGATCAAAAAAATCAGTTTCATTTGAAGGTAGTGGCAGCGCCG

12 GGAACCATATGCGAAGTAGATCAAAAAAATCAGTTTCATTTGAAGGTAGTGGCAGCGCCG

8 GGAACCATATGCGAAGTAGATAAAAAAAATCAGTTTCATTTGAAGGTAGTGGCAGCGCCG

7 GGAACCATATGCGAAGTAGATAAAAAAAATCAGTTTCATTTGAAGGTAGTGGCAGCGCCG

19 GGAACCATATGCGAAGTAGATCAAAAAAATCAGTTTCATTTGAAGGTAGTGGCAGCGCCG

18 GGAACCATATGCGAAGTAGATCAAAAAAATCAGTTTCATTTGAAGGTAGTGGCAGCGCCG

22 GGAACCATATGCGAAGTAGATCAAAAAAATCAGTTTCATTTGAAGGTAGTGGCAGCGCCG

21 GGAACCATATGCGAAGTAGATCAAAAAAATCAGTTTCATTTGAAGGTAGTGGCAGCGCCG

9 GGAACCATATGCGAAGTAGATCAAAAAAATCAGTTTCATTTGAAGGTAGTGGCAGCGCCG

6 GGAACCATATGCGAAGTAGATCAAAAAAATCAGTTTCATTTGAAGGTAGTGGCAGCGCCG

5 GGAACCATATGCGAAGTAGATCAAAAAAATCAGTTTCATTTGAAGGTAGTGGCAGCGCCG

4 GGAACCATATGCGAAGTAGATCAAAAAAATCAGTTTCATTTGAAGGTAGTGGCAGCGCCG

3 GGAACCATATGCGAAGTAGATCAAAAAAATCAGTTTCATTTGAAGGTAGTGGCAGCGCCG

20 GGAACCATATGCGAAGTAGATCAAAAAAATCAGTTTCATTTGAAGGTAGTGGCAGCGCCG

**********.********** ***.**********************************

**<<<<<<<<<<<<<<<<<<<<<<<<<<<<<<<<<<<<<<<<<<<<<<<<<<<<<<<<<<<<**

1RC TAAGAACTTTGAGTAAGTAACCAAAGTCATATCCACTGTATCAATCAGTTGTGTACAATG

16RC TAAGAACTTTGAGTAAGTAACCAAAGTCATATCCACTGTATCAATCAGTTGTGTACAATG

15RC TAAGAACTTTGAGTAAGTAACCAAAGTCATATCCACTGTATCAATCAGTTGTGTACAATG

2RC TAAGAACTTTGAGTAAGTAACCAAAGTCATATCCACTGTATCAATCAGTTGTGTACAATG

10 TAAGAACTTTGAGTAAGTAACCAAAGTCATATCCACTGCATCAATCAGTTGCGTACTATG

17 TAAGAACTTTGAGTAAGTAACCAAAGTCATATCCACTGCATCAATCAGTTGCGTACTATG

14 TAAGAACTTTGAGTAAGTAACCAAAGTCATATCCACTGCATCAATCAGTTGCGTACTATG

13 TAAGAACTTTGAGTAAGTAACCAAAGTCATATCCACTGCATCAATCAGTTGCGTACTATG

12 TAAGAACTTTGAGTAAGTAACCAAAGTCATATCCACTGCATCAATCAGTTGCGTACTATG

8 TAAGAACTTTGAGTAAGTAACCAAAGTCATATCCACTGCATCAATCAGTTGCGTACTATG

7 TAAGAACTTTGAGTAAGTAACCAAAGTCATATCCACTGCATCAATCAGTTGCGTACTATG

19 TAAGAACTTTGAGTAAGTAACCAAAGTCATATCCACTGCATCAATCAGTTGCGTACTATG

18 TAAGAACTTTGAGTAAGTAACCAAAGTCATATCCACTGCATCAATCAGTTGCGTACTATG

22 TAAGAACTTTGAGTAAGTAACCAAAGTCATATCCACTGCATCAATCAGTTGCGTACTATG

21 TAAGAACTTTGAGTAAGTAACCAAAGTCATATCCACTGCATCAATCAGTTGCGTACTATG

9 TAAGAACTTTGAGTAAGTAACCAAAGTCATATCCACTGCATCAATCAGTTGCGTACTATG

6 TAAGAACTTTGAGTAAGTAACCAAAGTCATATCCACTGCATCAATCAGTTGCGTACTATG

5 TAAGAACTTTGAGTAAGTAACCAAAGTCATATCCACTGCATCAATCAGTTGCGTACTATG

4 TAAGAACTTTGAGTAAGTAACCAAAGTCATATCCACTGCATCAATCAGTTGCGTACTATG

3 TAAGAACTTTGAGTAAGTAACCAAAGTCATATCCACTGCATCAATCAGTTGCGTACTATG

20 TAAGAACTTTGAGTAAGTAACCAAAGTCATATCCACTGCATCAATCAGTTGCGTACTATG

**************************************.************.**** ***

**<<<<<<<<<<<<<<<<<<<<<<<<<<<<<<<<<<<<<<<<<<<<<<<<<<<<<<<<<<<<**

1RC TGCTTCCAGTCTATACATACCTATGAAACGATATCCAGTGAACATGATCAAACAGAACAA

16RC TGCTTCCAGTCTATACATACCTATGAAACGATATCCAGTGAACATGATCAAACAGAACAA

15RC TGCTTCCAGTCTATACATACCTATGAAACGATATCCAGTGAACATGATCAAACAGAACAA

2RC TGCTTCCAGTCTATACATACCTATGAAACGATATCCAGTGAACATGATCAAACAGAACAA

10 TACTTGCAGTCTATACATACCTATGAAACGATATCCAGTGAACATGATCAAACAGAACAA

17 TACTTGCAGTCTATACATACCTATGAAACGATATCCAGTGAACATGATCAAACAGAACAA

14 TACTTGCAGTCTATACATACCTATGAAACGATATCCAGTGAACATGATCAAACAGAACAA

13 TACTTGCAGTCTATACATACCTATGAAACGATATCCAGTGAACATGATCAAACAGAACAA

12 TACTTGCAGTCTATACATACCTATGAAACGATATCCAGTGAACATGATCAAACAGAACAA

8 TACTTGCAGTCTATACATACCTATGAAACGATATCCAGTGAACATGATCAAACAGAACAA

7 TACTTGCAGTCTATACATACCTATGAAACGATATCCAGTGAACATGATCAAACAGAACAA

19 TACTTGCAGTCTATACATACCTATGAAACGATATCCAGTGAACATGATCAAACAGAACAA

18 TACTTGCAGTCTATACATACCTATGAAACGATATCCAGTGAACATGATCAAACAGAACAA

22 TACTTGCAGTCTATACATACCTATGAAACGATATCCAGTGAACATGATCAAACAGAACAA

21 TACTTGCAGTCTATACATACCTATGAAACGATATCCAGTGAACATGATCAAACAGAACAA

9 TACTTGCAGTCTATACATACCTATGAAACGATATCCAGTGAACATGATCAAACAGAACAA

6 TACTTGCAGTCTATACATACCTATGAAACGATATCCAGTGAACATGATCAAACAGAACAA

5 TACTTGCAGTCTATACATACCTATGAAACGATATCCAGTGAACATGATCAAACAGAACAA

4 TACTTGCAGTCTATACATACCTATGAAACGATATCCAGTGAACATGATCAAACAGAACAA

3 TACTTGCAGTCTATACATACCTATGAAACGATATCCAGTGAACATGATCAAACAGAACAA

20 TACTTGCAGTCTATACATACCTATGAAACGATATCCAGTGAACATGATCAAACAGAACAA

*.*** ******************************************************

**<<<<<<<<<<<<<<<<<<<<<<<<<<<<<<<<<<<<<<<<<<<<<<<<<<<<<<<<<<<<**

1RC AGCCAGATGTGACTAGCTTCTCACCAAATTCTTCTATATCGATACCATTGGCTTCCAGCC

16RC AGCCAGATGTGACTAGCTTCTCACCAAATTCTTCTATATCGATACCATTGGCTTCCAGCC

15RC AGCCAGATGTGACTAGCTTCTCACCAAATTCTTCTATATCGATACCATTGGCTTCCAGCC

2RC AGCCAGATGTGACTAGCTTCTCACCAAATTCTTCTATATCGATACCATTGGCTTCCAGCC

10 AACCAGATGTGACTAGCTTCTCACCAAACTCCTCTATATCGACACCATTAGCTTCCAGCC

17 AACCAGATGTGACTAGCTTCTCACCAAATTCCTCTATATCGACACCATTAGCTTCCAGCC

14 AACCAGATGTGACTAGCTTCTCACCAAATTCCTCTATATCGACACCATTAGCTTCCAGCC

13 AACCAGATGTGACTAGCTTCTCACCAAATTCCTCTATATCGACACCATTAGCTTCCAGCC

12 AACCAGATGTGACTAGCTTCTCACCAAATTCCTCTATATCGACACCATTAGCTTCCAGCC

8 AACCAGATGTGACTAGCTTCTCACCAAATTCCTCTATATCGACACCATTAGCTTCCAGCC

7 AACCAGATGTGACTAGCTTCTCACCAAATTCCTCTATATCGACACCATTAGCTTCCAGCC

19 AACCAGATGTGACTAGCTTCTCACCAAATTCCTCTATATCGACACCATTAGCTTCCAGCC

18 AACCAGATGTGACTAGCTTCTCACCAAATTCCTCTATATCGACACCATTAGCTTCCAGCC

22 AACCAGATGTGACTAGCTTCTCACCAAACTCCTCTATATCGACACCATTAGCTTCCAGCC

21 AACCAGATGTGACTAGCTTCTCACCAAATTCCTCTATATCGACACCATTAGCTTCCAGCC

9 AACCAGATGTGACTAGCTTCTCACCAAATTCCTCTATATCGACACCATTAGCTTCCAGCC

6 AACCAGATGTGACTAGCTTCTCACCAAATTCCTCTATATCGACACCATTAGCTTCCAGCC

5 AACCAGATGTGACTAGCTTCTCACCAAATTCCTCTATATCGACACCATTAGCTTCCAGCC

4 AACCAGATGTGACTAGCTTCTCACCAAATTCCTCTATATCGACACCATTAGCTTCCAGCC

3 AACCAGATGTGACTAGCTTCTCACCAAATTCCTCTATATCGACACCATTAGCTTCCAGCC

20 AACCAGATGTGACTAGCTTCTCACCAAATTCCTCTATATCGACACCATTAGCTTCCAGCC

*.**************************.**.**********.******.**********

**<<<<<<<<<<<<<<<<<<<<<<<<<<<<<<<<<<<<<<<<<<<<<<<<<<<<<<<<<<<<**

1RC TCTTGAAATTGATCCCAGATTTCGTGAGTAATTCTATTGAATCGGGTGCGTACATATCAT

16RC TCTTGAAATTGATCCCAGATTTCGTGAGTAATTCTATTGAATCGGGTGCGTACATATCAT

15RC TCTTGAAATTGATCCCAGATTTCGTGAGTAATTCTATTGAATCGGGTGCGTACATATCAT

2RC TCTTGAAATTGATCCCAGATTTCGTGAGTAATTCTATTGAATCGGGTGCGTACATATCAT

10 TCTTGAAATTTATCCCAGATTTCGTGAGTAATTCTATTGAATCGGGTGCGTACATATCAT

17 TCTTGAAATTTATCCCAGATTTCGTGAGTAATTCTATTGAATCGGGTGCGTACATATCAT

14 TCTTGAAATTTATCCCAGATTTCGTGAGTAATTCTATTGAATCGGGTGCGTACATATCAT

13 TCTTGAAATTTATCCCAGATTTCGTGAGTAATTCTATTGAATCGGGTGCGTACATATCAT

12 TCTTGAAATTTATCCCAGATTTCGTGAGTAATTCTATTGAATCGGGTGCGTACATATCAT

8 TCTTGAAATTTATCCCAGATTTCGTGAGTAATTCTATTGAATCGGGTGCGTACATATCAT

7 TCTTGAAATTTATCCCAGATTTCGTGAGTAATTCTATTGAATCGGGTGCGTACATATCAT

19 TCTTGAAATTTATCCCAGATTTCGTGAGTAATTCTATTGAATCGGGTGCGTACATATCAT

18 TCTTGAAATTTATCCCAGATTTCGTGAGTAATTCTATTGAATCGGGTGCGTACATATCAT

22 TCTTGAAATTTATCCCAGATTTCGTGAGTAATTCTATTGAATCGGGTGCGTACATATCAT

21 TCTTGAAATTTATCCCAGATTTCGTGAGTAATTCTATTGAATCGGGTGCGTACATATCAT

9 TCTTGAAATTTATCCCAGATTTCGTGAGTAATTCTATTGAATCGGGTGCGTACATATCAT

6 TCTTGAAATTTATCCCAGATTTCGTGAGTAATTCTATTGAATCGGGTGCGTACATATCAT

5 TCTTGAAATTTATCCCAGATTTCGTGAGTAATTCTATTGAATCGGGTGCGTACATATCAT

4 TCTTGAAATTTATCCCAGATTTCGTGAGTAATTCTATTGAATCGGGTGCGTACATATCAT

3 TCTTGAAATTTATCCCAGATTTCGTGAGTAATTCTATTGAATCGGGTGCGTACATATCAT

20 TCTTGAAATTTATCCCAGATTTCGTGAGTAATTCTATTGAATCGGGTGCGTACATATCAT

********** *************************************************

**<<<<<<<<<<<<<<<<<<<<<<<<<<<<<<<<<<<<<<<<<<<<<<<<<<<<<<<<<<<<**

1RC CACTGCGAATGTCATGAGAAGGAGACAAAGATGATGATGAGAATACTCACTTCAAATTGA

16RC CACTGCGAATGTCATGAGAAGGAGACAAAGATGATGATGAGAATACTCACTTCAAATTGA

15RC CACTGCGAATGTCATGAGAAGGAGACAAAGATGATGATGAGAATACTCACTTCAAATTGA

2RC CACTGCGAATGTCATGAGAAGGAGACAAAGATGATGATGAGAATACTCACTTCAAATTGA

10 CACTGCGAATGTCATGAGAATGGGACAAAGATGATGGTGAGAATACTCACTTCAAATTGA

17 CACTGCGAATGTCATGAGAATGGGACAAAGATGATGGTGAGAATACTCACTTCAAATTGA

14 CACTGCGAATGTCATGAGAATGGGACAAAGATGATGGTGAGAATACTCACTTCAAATTGA

13 CACTGCGAATGTCATGAGAATGGGACAAAGATGATGGTGAGAATACTCACTTCAAATTGA

12 CACTGCGAATGTCATGAGAATGGGACAAAGATGATGGTGAGAATACTCACTTCAAATTGA

8 CACTGCGAATGTCATGAGAATGGGACAAAGATGATGGTGAGAATACTCACTTCAAATTGA

7 CACTGCGAATGTCATGAGAATGGGACAAAGATGATGGTGAGAATACTCACTTCAAATTGA

19 CACTGCGAATGTCATGAGAATGGGACAAAGATGATGGTGAGAATACTCACTTCAAATTGA

18 CACTGCGAATGTCATGAGAATGGGACAAAGATGATGGTGAGAATACTCACTTCAAATTGA

22 CACTGCGAATGTCATGAGAATGGGACAAAGATGATGGTGAGAATACTCACTTCAAATTGA

21 CACTGCGAATGTCATGAGAATGGGACAAAGATGATGGTGAGAATACTCACTTCAAATTGA

9 CACTGCGAATGTCATGAGAATGGGACAAAGATGATGGTGAGAATACTCACTTCAAATTGA

6 CACTGCGAATGTCATGAGAATGGGACAAAGATGATGGTGAGAATACTCACTTCAAATTGA

5 CACTGCGAATGTCATGAGAATGGGACAAAGATGATGGTGAGAATACTCACTTCAAATTGA

4 CACTGCGAATGTCATGAGAATGGGACAAAGATGATGGTGAGAATACTCACTTCAAATTGA

3 CACTGCGAATGTCATGAGAATGGGACAAAGATGATGGTGAGAATACTCACTTCAAATTGA

20 CACTGCGAATGTCATGAGAATGGGACAAAGATGATGGTGAGAATACTCACTTCAAATTGA

******************** *.*************.***********************

**<<<<<<<<<<<<<<<<<<<<<<<<<<<<<<<<<<<<<<<<<<<<<<<<<<<<<<<<<<<<**

1RC ACTTGAAATTGAATTGCCAAGTCGGATATTCGGGCATGTTCCCCTCTGCGTCAGCGATAG

16RC ACTTGAAATTGAATTGCCAAGTCGGATATTCGGGCATGTTCCCCTCTGCGTCAGCGATAG

15RC ACTTGAAATTGAATTGCCAAGTCGGATATTCGGGCATGTTCCCCTCTGCGTCAGCGATAG

2RC ACTTGAAATTGAATTGCCAAGTCGGATATTCGGGCATGTTCCCCTCTGCGTCAGCGATAG

10 ATTTGAAATTGAATTGCCAAGTTGGATATTCGGGCATGTTCCCCTCTGCGTCAGCGATAG

17 ATTTGAAATTGAATTGCCAAGTTGGATATTCGGGCATGTTCCCCTCTGCGTCAGCGATAG

14 ATTTGAAATTGAATTGCCAAGTTGGATATTCGGGCATGTTCCCCTCTGCGTCAGCGATAG

13 ATTTGAAATTGAATTGCCAAGTTGGATATTCGGGCATGTTCCCCTCTGCGTCAGCGATAG

12 ATTTGAAATTGAATTGCCAAGTTGGATATTCGGGCATGTTCCCCTCTGCGTCAGCGATAG

8 ATTTGAAATTGAATTGCCAAGTTGGATATTCGGGCATGTTCCCCTCTGCGTCAGCGATAG

7 ATTTGAAATTGAATTGCCAAGTTGGATATTCGGGCATGTTCCCCTCTGCGTCAGCGATAG

19 ATTTGAAATTGAATTGCCAAGTTGGATATTCGGGCATGTTCCCCTCTGCGTCAGCGATAG

18 ATTTGAAATTGAATTGCCAAGTTGGATATTCGGGCATGTTCCCCTCTGCGTCAGCGATAG

22 ATTTGAAATTGAATTGCCAAGTTGGATATTCGGGCATGTTCCCCTCTGCGTCAGCGATAG

21 ATTTGAAATTGAATTGCCAAGTTGGATATTCGGGCATGTTCCCCTCTGCGTCAGCGATAG

9 ATTTGAAATTGAATTGCCAAGTTGGATATTCGGGCATGTTCCCCTCTGCGTCAGCGATAG

6 ATTTGAAATTGAATTGCCAAGTTGGATATTCGGGCATGTTCCCCTCTGCGTCAGCGATAG

5 ATTTGAAATTGAATTGCCAAGTTGGATATTCGGGCATGTTCCCCTCTGCGTCAGCGATAG

4 ATTTGAAATTGAATTGCCAAGTTGGATATTCGGGCATGTTCCCCTCTGCGTCAGCGATAG

3 ATTTGAAATTGAATTGCCAAGTTGGATATTCGGGCATGTTCCCCTCTGCGTCAGCGATAG

20 ATTTGAAATTGAATTGCCAAGTTGGATATTCGGGCATGTTCCCCTCTGCGTCAGCGATAG

*.********************.*************************************

**<<<<<<<<<<<<<<<<<<<<<<<<<<<<<<<<<<<<<<<<<<<<<<<<<<<<<<<<<<<<**

1RC TGATACCCAATTGAATGATTTTAAGGAGATCTACGTTGCACCGTAACGTTTGGTAATGGT

16RC TGATACCCAATTGAATGATTTTAAGGAGATCTACGTTGCACCGTAACGTTTGGTAATGGT

15RC TGATACCCAATTGAATGATTTTAAGGAGATCTACGTTGCACCGTAACGTTTGGTAATGGT

2RC TGATACCCAATTGAATGATTTTAAGGAGATCTACGTTGCACCGTAACGTTTGGTAATGGT

10 TGATACCCAATTGAATGATTTTAAGGAGATCTACGTTGCACCGTAACGTTTGGTAATGGT

17 TGATACCCAATTGAATGATTTTAAGGAGATCTACGTTGCACCGTAACGTTTGGTAATGGT

14 TGATACCCAATTGAATGATTTTAAGGAGATCTACGTTGCACCGTAACGTTTGGTAATGGT

13 TGATACCCAATTGAATGATTTTAAGGAGATCTACGTTGCACCGTAACGTTTGGTAATGGT

12 TGATACCCAATTGAATGATTTTAAGGAGATCTACGTTGCACCGTAACGTTTGGTAATGGT

8 TGATACCCAATTGAATGATTTTAAGGAGATCTACGTTGCACCGTAACGTTTGGTAATGGT

7 TGATACCCAATTGAATGATTTTAAGGAGATCTACGTTGCACCGTAACGTTTGGTAATGGT

19 TGATACCCAATTGAATGATTTTAAGGAGATCTACGTTGCACCGTAACGTTTGGTAATGGT

18 TGATACCCAATTGAATGATTTTAAGGAGATCTACGTTGCACCGTAACGTTTGGTAATGGT

22 TGATACCCAATTGAATGATTTTAAGGAGATCTACGTTGCACCGTAACGTTTGGTAATGGT

21 TGATACCCAATTGAATGATTTTAAGGAGATCTACGTTGCACCGTAACGTTTGGTAATGGT

9 TGATACCCAATTGAATGATTTTAAGGAGATCTACGTTGCACCGTAACGTTTGGTAATGGT

6 TGATACCCAATTGAATGATTTTAAGGAGATCTACGTTGCACCGTAACGTTTGGTAATGGT

5 TGATACCCAATTGAATGATTTTAAGGAGATCTACGTTGCACCGTAACGTTTGGTAATGGT

4 TGATACCCAATTGAATGATTTTAAGGAGATCTACGTTGCACCGTAACGTTTGGTAATGGT

3 TGATACCCAATTGAATGATTTTAAGGAGATCTACGTTGCACCGTAACGTTTGGTAATGGT

20 TGATACCCAATTGAATGATTTTAAGGAGATCTACGTTGCACCGTAACGTTTGGTAATGGT

************************************************************

**<<<<<<<<<<<<<<<<<<<<<<<<<<<<<<<<<<<<<<<<<<<<<<<<<<<<<<<<<<<<**

1RC AATCTGAAGAATTCTTAAAGTTCCCAATCGGCCTTGCTACAACACCAGGAAATTCCGTGT

16RC AATCTGAAGAATTCTTAAAGTTCCCAATCGGCCTTGCTACAACACCAGGAAATTCCGTGT

15RC AATCTGAAGAATTCTTAAAGTTCCCAATCGGCCTTGCTACAACACCAGGAAATTCCGTGT

2RC AATCTGAAGAATTCTTAAAGTTCCCAATCGGCCTTGCTACAACACCAGGAAATTCCGTGT

10 AATCTGAAGAATTCTTAAAGTTCCCAATCGGCCTTGCTACAACACCAGGAAATTCCGTGT

17 AATCTGAAGAATTCTTAAAGTTCCCAATCGGCCTTGCTACAACACCAGGAAATTCCGTGT

14 AATCTGAAGAATTCTTAAAGTTCCCAATCGGCCTTGCTACAACACCAGGAAATTCCGTGT

13 AATCTGAAGAATTCTTAAAGTTCCCAATCGGCCTTGCTACAACACCAGGAAATTCCGTGT

12 AATCTGAAGAATTCTTAAAGTTCCCAATCGGCCTTGCTACAACACCAGGAAATTCCGTGT

8 AATCTGAAGAATTCTTAAAGTTCCCAATCGGCCTTGCTACAACACCAGGAAATTCCGTGT

7 AATCTGAAGAATTCTTAAAGTTCCCAATCGGCCTTGCTACAACACCAGGAAATTCCGTGT

19 AATCTGAAGAATTCTTAAAGTTCCCAATCGGCCTTGCTACAACACCAGGAAATTCCGTGT

18 AATCTGAAGAATTCTTAAAGTTCCCAATCGGCCTTGCTACAACACCAGGAAATTCCGTGT

22 AATCTGAAGAATTCTTAAAGTTCCCAATCGGCCTTGCTACAACACCAGGAAATTCCGTGT

21 AATCTGAAGAATTCTTAAAGTTCCCAATCGGCCTTGCTACAACACCAGGAAATTCCGTGT

9 AATCTGAAGAATTCTTAAAGTTCCCAATCGGCCTTGCTACAACACCAGGAAATTCCGTGT

6 AATCTGAAGAATTCTTAAAGTTCCCAATCGGCCTTGCTACAACACCAGGAAATTCCGTGT

5 AATCTGAAGAATTCTTAAAGTTCCCAATCGGCCTTGCTACAACACCAGGAAATTCCGTGT

4 AATCTGAAGAATTCTTAAAGTTCCCAATCGGCCTTGCTACAACACCAGGAAATTCCGTGT

3 AATCTGAAGAATTCTTAAAGTTCCCAATCGGCCTTGCTACAACACCAGGAAATTCCGTGT

20 AATCTGAAGAATTCTTAAAGTTCCCAATCGGCCTTGCTACAACACCAGGAAATTCCGTGT

************************************************************

**<<<<<<<<<<<<<<<<<<<<<<<<<<<<<<<<<<<<<<<<<<<<<<<<<<<<<<<<<<<<**

1RC CCTTGGGCTGTTAGCCTGAATGTCAAAGATGACAGCCAAACTAACCATGGACACGTATGG

16RC CCTTGGGCTGTTAGCCTGAATGTCAAAGATGACAGCCAAACTAACCATGGACACGTATGG

15RC CCTTGGGCTGTTAGCCTGAATGTCAAAGATGACAGCCAAACTAACCATGGACACGTAAGG

2RC CCTTGGGCTGTTAGCCTGAATGTCAAAGATGACAGCCAAACTAACCATGGACACGTAAGG

10 CCTAGGGCTGTTAGCCTGAGTGTCAAAGATGACAGCCAAACTAACCATGGACACGTATGG

17 CCTAGGGCTGTTAGCCTGAGTGTCAAAGATGACAGCCAAACTAACCATGGACACGTATGG

14 CCTAGGGCTGTTAGCCTGAGTGTCAAAGATGACAGCCAAACTAACCATGGACACGTATGG

13 CCTAGGGCTGTTAGCCTGAGTGTCAAAGATGACAGCCAAACTAACCATGGACACGTATGG

12 CCTAGGGCTGTTAGCCTGAGTGTCAAAGATGACAGCCAAACTAACCATGGACACGTATGG

8 CCTAGGGCTGTTAGCCTGAGTGTCAAAGATGACAGCCAAACTAACCATGGACACGTATGG

7 CCTAGGGCTGTTAGCCTGAGTGTCAAAGATGACAGCCAAACTAACCATGGACACGTATGG

19 CCTAGGGCTGTTAGCCTGAGTGTCAAAGATGACAGCCAAACTAACCATGGACACGTATGG

18 CCTAGGGCTGTTAGCCTGAGTGTCAAAGATGACAGCCAAACTAACCATGGACACGTATGG

22 CCTAGGGCTGTTAGCCTGAGTGTCAAAGATGACAGCCAAACTAACCATGGACACGTATGG

21 CCTAGGGCTGTTAGCCTGAGTGTCAAAGATGACAGCCAAACTAACCATGGACACGTATGG

9 CCTAGGGCTGTTAGCCTGAGTGTCAAAGATGACAGCCAAACTAACCATGGACACGTATGG

6 CCTAGGGCTGTTAGCCTGAGTGTCAAAGATGACAGCCAAACTAACCATGGACACGTATGG

5 CCTAGGGCTGTTAGCCTGAGTGTCAAAGATGACAGCCAAACTAACCATGGACACGTATGG

4 CCTAGGGCTGTTAGCCTGAGTGTCAAAGATGACAGCCAAACTAACCATGGACACGTATGG

3 CCTAGGGCTGTTAGCCTGAGTGTCAAAGATGACAGCCAAACTAACCATGGACACGTATGG

20 CCTAGGGCTGTTAGCCTGAGTGTCAAAGATGACAGCCAAACTAACCATGGACACGTATGG

*** ***************.************************************* **

**<<<<<<<<<<<<<<<<<<<<<<<<<<<<<<<<<<<<<<<<<<<<<<<<<<<<<<<<<<<<**

1RC ATATCGATCAACTAGTTCACGGAGAATATCTAATTCAGCATCAAGATTATCTGCCCAAAC

16RC ATATCGATCAACTAGTTCACGGAGAATATCTAATTCAGCATCAAGATTATCTGCCCAAAC

15RC ATATCGATCAACTAGTTCACGGAGAATATCTAATTCAGCATCAAGATTATCTGCCCAAAC

2RC ATATCGATCAACTAGTTCACGGAGAATATCTAATTCAGCATCAAGATTATCTGCCCAAAC

10 ATATCGATCTACTAATTCACGGAGAATATCTAATTCGGCATCAAGATTATCTGCCCAAAC

17 ATATCGATCTACTAATTCACGGAGAATATCTAATTCGGCATCAAGATTATCTGCCCAAAC

14 ATATCGATCTACTAATTCACGGAGAATATCTAATTCGGCATCAAGATTATCTGCCCAAAC

13 ATATCGATCTACTAATTCACGGAGAATATCTAATTCGGCATCAAGATTATCTGCCCAAAC

12 ATATCGATCTACTAATTCACGGAGAATATCTAATTCGGCATCAAGATTATCTGCCCAAAC

8 ATATCGATCTACTAATTCACGGAGAATATCTAATTCGGCATCAAGATTATCTGCCCAAAC

7 ATATCGATCTACTAATTCACGGAGAATATCTAATTCGGCATCAAGATTATCTGCCCAAAC

19 ATATCGATCTACTAATTCACGGAGAATATCTAATTCGGCATCAAGATTATCTGCCCAAAC

18 ATATCGATCTACTAATTCACGGAGAATATCTAATTCGGCATCAAGATTATCTGCCCAAAC

22 ATATCGATCTACTAATTCACGGAGAATATCTAATTCGGCATCAAGATTATCTGCCCAAAC

21 ATATCGATCTACTAATTCACGGAGAATATCTAATTCGGCATCAAGATTATCTGCCCAAAC

9 ATATCGATCTACTAATTCACGGAGAATATCTAATTCGGCATCAAGATTATCTGCCCAAAC

6 ATATCGATCTACTAATTCACGGAGAATATCTAATTCGGCATCAAGATTATCTGCCCAAAC

5 ATATCGATCTACTAATTCACGGAGAATATCTAATTCGGCATCAAGATTATCTGCCCAAAC

4 ATATCGATCTACTAATTCACGGAGAATATCTAATTCGGCATCAAGATTATCTGCCCAAAC

3 ATATCGATCTACTAATTCACGGAGAATATCTAATTCGGCATCAAGATTATCTGCCCAAAC

20 ATATCGATCTACTAATTCACGGAGAATATCTAATTCGGCATCAAGATTATCTGCCCAAAC

********* ****.*********************.***********************

**<<<<<<<<<<<<<<<<<<<<<<<<<<<<<<<<<<<<<<<<<<<<<<<<<<<<<<<<<<<<**

1RC CTCGCGAATCTGGCTCTGCATCTTTCTTGATAGTAGTAAATCAAGTCGACGGAATAGTCA

16RC CTCGCGAATCTGGCTCTGCATCTTTCTTGATAGTAGTAAATCAAGTCGACGGAATAGTCA

15RC CTCGCGAATCTGGCTCTGCATCTTTCTTGATAGTAGTAAATCAAGTCGACGGAATAGTCA

2RC CTCGCGAATCTGGCTCTGCATCTTTCTTGATAGTAGTAAATCAAGTCGACGGAATAGTCA

10 CTCGCGAATCTGGCTCTGCATCTTTCTTGATAGTAGTAAATCAAGTCGACGGAATAGTCT

17 CTCGCGAATCTGGCTCTGCATCTTTCTTGATAGTAGTAAATCAAGTCGACGGAATAGTCT

14 CTCGCGAATCTGGCTCTGCATCTTTCTTGATAGTAGTAAATCAAGTCGACGGAATAGTCT

13 CTCGCGAATCTGGCTCTGCATCTTTCTTGATAGTAGTAAATCAAGTCGACGGAATAGTCT

12 CTCGCGAATCTGGCTCTGCATCTTTCTTGATAGTAGTAAATCAAGTCGACGGAATAGTCT

8 CTCGCGAATCTGGCTCTGCATCTTTCTTGATAGTAGTAAATCAAGTCGACGGAATAGTCT

7 CTCGCGAATCTGGCTCTGCATCTTTCTTGATAGTAGTAAATCAAGTCGACGGAATAGTCT

19 CTCGCGAATCTGGCTCTGCATCTTTCTTGATAGTAGTAAATCAAGTCGACGGAATAGTCT

18 CTCGCGAATCTGGCTCTGCATCTTTCTTGATAGTAGTAAATCAAGTCGACGGAATAGTCT

22 CTCGCGAATCTGGCTCTGCATCTTTCTTGATAGTAGTAAATCAAGTCGACGGAATAGTCT

21 CTCGCGAATCTGGCTCTGCATCTTTCTTGATAGTAGTAAATCAAGTCGACGGAATAGTCT

9 CTCGCGAATCTGGCTCTGCATCTTTCTTGATAGTAGTAAATCAAGTCGACGGAATAGTCT

6 CTCGCGAATCTGGCTCTGCATCTTTCTTGATAGTAGTAAATCAAGTCGACGGAATAGTCT

5 CTCGCGAATCTGGCTCTGCATCTTTCTTGATAGTAGTAAATCAAGTCGACGGAATAGTCT

4 CTCGCGAATCTGGCTCTGCATCTTTCTTGATAGTAGTAAATCAAGTCGACGGAATAGTCT

3 CTCGCGAATCTGGCTCTGCATCTTTCTTGATAGTAGTAAATCAAGTCGACGGAATAGTCT

20 CTCGCGAATCTGGCTCTGCATCTTTCTTGATAGTAGTAAATCAAGTCGACGGAATAGTCT

***********************************************************

**<<<<<<<<<<<<<<<<<CAF1**

1RC AAAATTCTGAATTAAATGGTTTTACAGGATAAATACAAAGCTAAGGCTTCTCGCGCATGG

16RC AAAATTCTGAATTAAATGGTTTTACAGGATAAATACAAAGCTAAGGCTTCTCGCGCATGG

15RC AAAATTCTGAATTAAATGGTTTTACAGGATAAATACAAAGCTAAGGCTTCTCGCGCATGG

2RC AAAATTCTGAATTAAATGGTTTTACAGGATAAATACAAAGCTAAGGCTTCTCGCGCATGG

10 AAAAATCTGAATTAAATGGTTTTACAGGACAAATATAAAGCAAAGGCTTCTCGCGCATGG

17 AAAAATCTGAATTAAATGGTTTTACAGGACAAATATAAAGCAAAGGCTTCTCGCGCATGG

14 AAAAATCTGAATTAAATGGTTTTACAGGACAAATATAAAGCAAAGGCTTCTCGCGCATGG

13 AAAAATCTGAATTAAATGGTTTTACAGGACAAATATAAAGCAAAGGCTTCTCGCGCATGG

12 AAAAATCTGAATTAAATGGTTTTACAGGACAAATATAAAGCAAAGGCTTCTCGCGCATGG

8 AAAAATCTGAATTAAATGGTTTTACAGGACAAATATAAAGCAAAGGCTTCTCGCGCATGG

7 AAAAATCTGAATTAAATGGTTTTACAGGACAAATATAAAGCAAAGGCTTCTCGCGCATGG

19 AAAAATCTGAATTAAATGGTTTTACAGGACAAATATAAAGCAAAGGCTTCTCGCGCATGG

18 AAAAATCTGAATTAAATGGTTTTACAGGACAAATATAAAGCAAAGGCTTCTCGCGCATGG

22 AAAAATCTGAATTAAATGGTTTTACAGGACAAATATAAAGCAAAGGCTTCTCGCGCATGG

21 AAAAATCTGAATTAAATGGTTTTACAGGACAAATATAAAGCAAAGGCTTCTCGCGCATGG

9 AAAAATCTGAATTAAATGGTTTTACAGGACAAATATAAAGCAAAGGCTTCTCGCGCATGG

6 AAAAATCTGAATTAAATGGTTTTACAGGACAAATATAAAGCAAAGGCTTCTCGCGCATGG

5 AAAAATCTGAATTAAATGGTTTTACAGGACAAATATAAAGCAAAGGCTTCTCGCGCATGG

4 AAAAATCTGAATTAAATGGTTTTACAGGACAAATATAAAGCAAAGGCTTCTCGCGCATGG

3 AAAAATCTGAATTAAATGGTTTTACAGGACAAATATAAAGCAAAGGCTTCTCGCGCATGG

20 AAAAATCTGAATTAAATGGTTTTACAGGACAAATATAAAGCAAAGGCTTCTCGCGCATGG

**** ************************.*****.***** ******************

1RC AAATCTTCTAGAGGCTTGAGTACAGCCGCGAAACCTAGCAGGAG------ACCACCACCA

16RC AAATCGTCTAGAGGCTTGAGTACAGCCGCGAAACCTAGCAGGAG------ACCACCACCA

15RC AAATCGTCTAGAGGCTTGAGTACAGCCGCGAAACCTAGCAGGAG------ACCACCACCA

2RC AAATCGTCTAGAGGCTTGAGTACAGCCGCGAAACCTAGCAGGAG------ACCACCACCA

10 AAATCTTCTAGAGGCTTGAGTACAGCCACGAGACCTAACAGAAGACCCCCACCGCCACCA

17 AAATCTTCTAGAGGCTTGAGTACAGCCACGAGACCTAACAGAAGACCCCCACCGCCACCA

14 AAATCTTCTAGAGGCTTGAGTACAGCCACGAGACCTAACAGAAGACCCCCACCGCCACCA

13 AAATCTTCTAGAGGCTTGAGTACAGCCACGAGACCTAACAGAAGACCCCCACCGCCACCA

12 AAATCTTCTAGAGGCTTGAGTACAGCCACGAGACCTAACAGAAGACCCCCACCGCCACCA

8 AAATCTTCTAGAGGCTTGAGTACAGCCACGAGACCTAACAGAAGACCCCCACCGCCACCA

7 AAATCTTCTAGAGGCTTGAGTACAGCCACGAGACCTAACAGAAGACCCCCACCGCCACCA

19 AAATCTTCTAGAGGCTTGAGTACAGCCACGAGACCTAACAGAAGACCCCCACCGCCACCA

18 AAATCTTCTAGAGGCTTGAGTACAGCCACGAGACCTAACAGAAGACCCCCACCGCCACCA

22 AAATCTTCTAGAGGCTTGAGTACAGCCACGAGACCTAACAGAAGACCCCCACCGCCACCA

21 AAATCTTCTAGAGGCTTGAGTACAGCCACGAGACCTAACAGAAGACCCCCACCGCCACCA

9 AAATCTTCTAGAGGCTTGAGTACAGCCACGAGACCTAACAGAAGACCCCCACCGCCACCA

6 AAATCTTCTAGAGGCTTGAGTACAGCCACGAGACCTAACAGAAGACCCCCACCGCCACCA

5 AAATCTTCTAGAGGCTTGAGTACAGCCACGAGACCTAACAGAAGACCCCCACCGCCACCA

4 AAATCTTCTAGAGGCTTGAGTACAGCCACGAGACCTAACAGAAGACCCCCACCGCCACCA

3 AAATCTTCTAGAGGCTTGAGTACAGCCACGAGACCTAACAGAAGACCCCCACCGCCACCA

20 AAATCTTCTAGAGGCTTGAGTACAGCCACGAGACCTAACAGAAGACCCCCACCGCCACCA

***** *********************.***.*****.***.** ***.******

1RC CCAACACTCAATGATGAAGCATCATTCCCAGAACTTCAGAAGCAGGCTGTCCCCATTGAT

16RC CCAACACTCAATGATGAAGCATCATTCCCAGAACTTCAGAAGCAGGCTGTCCCCATTGAT

15RC CCAACACTCAATGATGAAGCATCATTCCCAGAACTTCAGAAGCAGGCTGTCCCCATTGAT

2RC CCAACACTCAATGATGAAGCATCATTCCCAGAACTTCAGAAGCAGGCTGTCCCCATTGAT

10 CCAACACTCACTGATGAAGTAGCATTCCCAGAGCTTCAGAAGCAGGATGTCCCCATTGAT

17 CCAACACTCACTGATGAAGTAGCATTCCCAGAGCTTCAGAAGCAGGATGTCCCCATTGAT

14 CCAACACTCACTGATGAAGTAGCATTCCCAGAGCTTCAGAAGCAGGATGTCCCCATTGAT

13 CCAACACTCACTGATGAAGTAGCATTCCCAGAGCTTCAGAAGCAGGATGTCCCCATTGAT

12 CCAACACTCACTGATGAAGTAGCATTCCCAGAGCTTCAGAAGCAGGATGTCCCCATTGAT

8 CCAACACTCACTGATGAAGTAGCATTCCCAGAGCTTCAGAAGCAGGATGTCCCCATTGAT

7 CCAACACTCACTGATGAAGTAGCATTCCCAGAGCTTCAGAAGCAGGATGTCCCCATTGAT

19 CCAACACTCACTGATGAAGTAGCATTCCCAGAGCTTCAGAAGCAGGATGTCCCCATTGAT

18 CCAACACTCACTGATGAAGTAGCATTCCCAGAGCTTCAGAAGCAGGATGTCCCCATTGAT

22 CCAACACTCACTGATGAAGTAGCATTCCCAGAGCTTCAGAAGCAGGATGTCCCCATTGAT

21 CCAACACTCACTGATGAAGTAGCATTCCCAGAGCTTCAGAAGCAGGATGTCCCCATTGAT

9 CCAACACTCACTGATGAAGTAGCATTCCCAGAGCTTCAGAAGCAGGATGTCCCCATTGAT

6 CCAACACTCACTGATGAAGTAGCATTCCCAGAGCTTCAGAAGCAGGATGTCCCCATTGAT

5 CCAACACTCACTGATGAAGTAGCATTCCCAGAGCTTCAGAAGCAGGATGTCCCCATTGAT

4 CCAACACTCACTGATGAAGTAGCATTCCCAGAGCTTCAGAAGCAGGATGTCCCCATTGAT

3 CCAACACTCACTGATGAAGTAGCATTCCCAGAGCTTCAGAAGCAGGATGTCCCCATTGAT

20 CCAACACTCACTGATGAAGTAGCATTCCCAGAGCTTCAGAAGCAGGATGTCCCCATTGAT

********** ********.* **********.************* *************

1RC AGTGGAAGTGGAAGTGGA------AGTGACACTGGAGACAATTCTCAGGATGAACCCAAT

16RC AGTGGAAGTGGAAGTGGA------AGTGACACTGGAGACAATTCTCAGGATGAACCCAAT

15RC AGTGGAAGTGGAAGTGGA------AGTGACACTGGAGACAATTCTCAGGATGAACCCAAT

2RC AGTGGAAGTGGAAGTGGA------AGTGACACTGGAGACAATTCTCAGGATGAACCCAAT

10 AGTGGAAGTGGAAGTGGAAGTGGGAGTGACACTGGAGACGATTCTCAGGATGAATCCAAT

17 AGTGGAAGTGGAAGTGGAAGTGGGAGTGACACTGGAGACGATTCTCAGGATGAATCCAAT

14 AGTGGAAGTGGAAGTGGAAGTGGGAGTGACACTGGAGACGATTCTCAGGATGAATCCAAT

13 AGTGGAAGTGGAAGTGGAAGTGGGAGTGACACTGGAGACGATTCTCAGGATGAATCCAAT

12 AGTGGAAGTGGAAGTGGAAGTGGGAGTGACACTGGAGACGATTCTCAGGATGAATCCAAT

8 AGTGGAAGTGGAAGTGGAAGTGGGAGTGACACTGGAGACGATTCTCAGGATGAATCCAAT

7 AGTGGAAGTGGAAGTGGAAGTGGGAGTGACACTGGAGACGATTCTCAGGATGAATCCAAT

19 AGTGGAAGTGGAAGTGGAAGTGGGAGTGACACTGGAGACGATTCTCAGGATGAATCCAAT

18 AGTGGAAGTGGAAGTGGAAGTGGGAGTGACACTGGAGACGATTCTCAGGATGAATCCAAT

22 AGTGGAAGTGGAAGTGGAAGTGGGAGTGACACTGGAGACGATTCTCAGGATGAATCCAAT

21 AGTGGAAGTGGAAGTGGAAGTGGGAGTGACACTGGAGACGATTCTCAGGATGAATCCAAT

9 AGTGGAAGTGGAAGTGGAAGTGGGAGTGACACTGGAGACGATTCTCAGGATGAATCCAAT

6 AGTGGAAGTGGAAGTGGAAGTGGGAGTGACACTGGAGACGATTCTCAGGATGAATCCAAT

5 AGTGGAAGTGGAAGTGGAAGTGGGAGTGACACTGGAGACGATTCTCAGGATGAATCCAAT

4 AGTGGAAGTGGAAGTGGAAGTGGGAGTGACACTGGAGACGATTCTCAGGATGAATCCAAT

3 AGTGGAAGTGGAAGTGGAAGTGGGAGTGACACTGGAGACGATTCTCAGGATGAATCCAAT

20 AGTGGAAGTGGAAGTGGAAGTGGGAGTGACACTGGAGACGATTCTCAGGATGAATCCAAT

****************** ***************.**************.*****

1RC TCTGCGGGTGGAGTTGCAGTTGCAGCTAGCAGTAGCAGCAACAGTGAGTTGATAAAATCT

16RC TCTGCGGGTGGAGTTGCAGTTGCAGCTAGCAGTAGCAGCAACAGTGAGTTGATAAAATCT

15RC TCTGCGGGTGGAGTTGCAGTTGCAGCTAGCAGTAGCAGCAACAGTGAGTTGATAAAATCT

2RC TCTGCGGGTGGAGTTGCAGTTGCAGCTAGCAGTAGCAGCAACAGTGAGTTGATAAAATCT

10 TCCGCGGGTGGAGTTGCAGTTGCAGCTAGCATTACTAGCAACAGTAAGTTGACAATATCT

17 TCCGCGGGTGGAGTTGCAGTTGCAGCTAGCATTACTAGCAACAGTAAGTTGACAATATCT

14 TCCGCGGGTGGAGTTGCAGTTGCAGCTAGCATTACTAGCAACAGTAAGTTGACAATATCT

13 TCCGCGGGTGGAGTTGCAGTTGCAGCTAGCATTACTAGCAACAGTAAGTTGACAATATCT

12 TCCGCGGGTGGAGTTGCAGTTGCAGCTAGCATTACTAGCAACAGTAAGTTGACAATATCT

8 TCCGCGGGTGGAGTTGCAGTTGCAGCTAGCATTACTAGCAACAGTAAGTTGACAATATCT

7 TCCGCGGGTGGAGTTGCAGTTGCAGCTAGCATTACTAGCAACAGTAAGTTGACAATATCT

19 TCCGCGGGTGGAGTTGCAGTTGCAGCTAGCATTACTAGCAACAGTAAGTTGACAATATCT

18 TCCGCGGGTGGAGTTGCAGTTGCAGCTAGCATTACTAGCAACAGTAAGTTGACAATATCT

22 TCCGCGGGTGGAGTTGCAGTTGCAGCTAGCATTACTAGCAACAGTAAGTTGACAATATCT

21 TCCGCGGGTGGAGTTGCAGTTGCAGCTAGCATTACTAGCAACAGTAAGTTGACAATATCT

9 TCCGCGGGTGGAGTTGCAGTTGCAGCTAGCATTACTAGCAACAGTAAGTTGACAATATCT

6 TCCGCGGGTGGAGTTGCAGTTGCAGCTAGCATTACTAGCAACAGTAAGTTGACAATATCT

5 TCCGCGGGTGGAGTTGCAGTTGCAGCTAGCATTACTAGCAACAGTAAGTTGACAATATCT

4 TCCGCGGGTGGAGTTGCAGTTGCAGCTAGCATTACTAGCAACAGTAAGTTGACAATATCT

3 TCCGCGGGTGGAGTTGCAGTTGCAGCTAGCATTACTAGCAACAGTAAGTTGACAATATCT

20 TCCGCGGGTGGAGTTGCAGTTGCAGCTAGCATTACTAGCAACAGTAAGTTGACAATATCT

**.**************************** ** .*********.******.** ****

1RC AATCGTCAGCTATCTCAATGAAGTAGCCGCTAGGCGATCACAGCACTCAAAGTTTTCTAG

16RC AATCGTCAGCTATCTCAATGAAGTAGCCGCTAGGCGATCACAGCACTCAAAGTTTTCTAG

15RC AATCGTCAGCTATCTCAATGAAGTAGCCGCTAGGCGATCACAGCACTCAAAGTTTTCTAG

2RC AATCGTCAGCTATCTCAATGAAGTAGCCGCTAGGCGATCACAGCACTCAAAGTTTTCTAG

10 GATCAACAGCTATCTCAATCAAGTAGCCGCTAGGCGATCACAGCACCCAAAGTTTTCTAG

17 GATCAACAGCTATCTCAATCAAGTAGCCGCTAGGCGATCACAGCACCCAAAGTTTTCTAG

14 GATCAACAGCTATCTCAATCAAGTAGCCGCTAGGCGATCACAGCACCCAAAGTTTTCTAG

13 GATCAACAGCTATCTCAATCAAGTAGCCGCTAGGCGATCACAGCACCCAAAGTTTTCTAG

12 GATCAACAGCTATCTCAATCAAGTAGCCGCTAGGCGATCACAGCACCCAAAGTTTTCTAG

8 GATCAACAGCTATCTCAATCAAGTAGCCGCTAGGCGATCACAGCACCCAAAGTTTTCTAG

7 GATCAACAGCTATCTCAATCAAGTAGCCGCTAGGCGATCACAGCACCCAAAGTTTTCTAG

19 GATCAACAGCTATCTCAATCAAGTAGCCGCTAGGCGATCACAGCACCCAAAGTTTTCTAG

18 GATCAACAGCTATCTCAATCAAGTAGCCGCTAGGCGATCACAGCACCCAAAGTTTTCTAG

22 GATCAACAGCTATCTCAATCAAGTAGCCGCTAGGCGATCACAGCACCCAAAGTTTTCTAG

21 GATCAACAGCTATCTCAATCAAGTAGCCGCTAGGCGATCACAGCACCCAAAGTTTTCTAG

9 GATCAACAGCTATCTCAATCAAGTAGCCGCTAGGCGATCACAGCACCCAAAGTTTTCTAG

6 GATCAACAGCTATCTCAATCAAGTAGCCGCTAGGCGATCACAGCACCCAAAGTTTTCTAG

5 GATCAACAGCTATCTCAATCAAGTAGCCGCTAGGCGATCACAGCACCCAAAGTTTTCTAG

4 GATCAACAGCTATCTCAATCAAGTAGCCGCTAGGCGATCACAGCACCCAAAGTTTTCTAG

3 GATCAACAGCTATCTCAATCAAGTAGCCGCTAGGCGATCACAGCACCCAAAGTTTTCTAG

20 GATCAACAGCTATCTCAATCAAGTAGCCGCTAGGCGATCACAGCACCCAAAGTTTTCTAG

.***. ************* **************************.*************

1RC ACGGCGTTTGGTAAATAACTCTAGTAGATATGATGAGCCAGACGACGAAGGCGAAGAAGG

16RC ACGGCGTTTGGTAAATAACTCTAGTAGATATGATGAGCCAGACGACGAAGGCGAAGAAGG

15RC ACGGCGTTTGGTAAATAACTCTAGTAGATATGATGAGCCAGACGACGAAGGCGAAGAAGG

2RC ACGGCGTTTGGTAAATAACTCTAGTAGATATGATGAGCCAGACGACGAAGGCGAAGAAGG

10 GCGGCGTTTGGTAAATAACTCTAGTAGATATGATGAGCCAGACGACGAAGGCGAGGAAGG

17 GCGGCGTTTGGTAAATAACTCTAGTAGATATGATGAGCCAGACGACGAAGGCGAGGAAGG

14 GCGGCGTTTGGTAAATAACTCTAGTAGATATGATGAGCCAGACGACGAAGGCGAGGAAGG

13 GCGGCGTTTGGTAAATAACTCTAGTAGATATGATGAGCCAGACGACGAAGGCGAGGAAGG

12 GCGGCGTTTGGTAAATAACTCTAGTAGATATGATGAGCCAGACGACGAAGGCGAGGAAGG

8 GCGGCGTTTGGTAAATAACTCTAGTAGATATGATGAGCCAGACGACGAAGGCGAGGAAGG

7 GCGGCGTTTGGTAAATAACTCTAGTAGATATGATGAGCCAGACGACGAAGGCGAGGAAGG

19 GCGGCGTTTGGTAAATAACTCTAGTAGATATGATGAGCCAGACGACGAAGGCGAGGAAGG

18 GCGGCGTTTGGTAAATAACTCTAGTAGATATGATGAGCCAGACGACGAAGGCGAGGAAGG

22 GCGGCGTTTGGTAAATAACTCTAGTAGATATGATGAGCCAGACGACGAAGGCGAGGAAGG

21 GCGGCGTTTGGTAAATAACTCTAGTAGATATGATGAGCCAGACGACGAAGGCGAGGAAGG

9 GCGGCGTTTGGTAAATAACTCTAGTAGATATGATGAGCCAGACGACGAAGGCGAGGAAGG

6 GCGGCGTTTGGTAAATAACTCTAGTAGATATGATGAGCCAGACGACGAAGGCGAGGAAGG

5 GCGGCGTTTGGTAAATAACTCTAGTAGATATGATGAGCCAGACGACGAAGGCGAGGAAGG

4 GCGGCGTTTGGTAAATAACTCTAGTAGATATGATGAGCCAGACGACGAAGGCGAGGAAGG

3 GCGGCGTTTGGTAAATAACTCTAGTAGATATGATGAGCCAGACGACGAAGGCGAGGAAGG

20 GCGGCGTTTGGTAAATAACTCTAGTAGATATGATGAGCCAGACGACGAAGGCGAGGAAGG

.*****************************************************.*****

1RC TTTGAATATTGATTTAACTCA-CATTATTAACTAAGGATATAGAAGGTCATTTATCAAAT

16RC TTTGAATATTGATTTAACTCA-CATTATTAACTAAGGATATAGAAGGTCATTTATCAAAT

15RC TTTGAATATTGATTTAACTCA-CATTATTAACTAAGGATATAGAAGGTCATTTATCAAAT

2RC TTTGAATATTGATTTAACTCA-CATTATTAACTAAGGATATAGAAGGTCATTTATCAAAT

10 TTTGAATATTCATTTAACTCACCTTTATTTACTAAGGATATACAAGGTCATTCATCAAAT

17 TTTGAATATTCATTTAACTCACCTTTATTTACTAAGGATATACAAGGTCATTCATCAAAT

14 TTTGAATATTCATTTAACTCACCTTTATTTACTAAGGATATACAAGGTCATTCATCAAAT

13 TTTGAATATTCATTTAACTCACCTTTATTTACTAAGGATATACAAGGTCATTCATCAAAT

12 TTTGAATATTCATTTAACTCACCTTTATTTACTAAGGATATACAAGGTCATTCATCAAAT

8 TTTGAATATTCATTTAACTCACCTTTATTTACTAAGGATATACAAGGTCATTCATCAAAT

7 TTTGAATATTCATTTAACTCACCTTTATTTACTAAGGATATACAAGGTCATTCATCAAAT

19 TTTGAATATTCATTTAACTCACCTTTATTTACTAAGGATATACAAGGTCATTCATCAAAT

18 TTTGAATATTCATTTAACTCACCTTTATTTACTAAGGATATACAAGGTCATTCATCAAAT

22 TTTGAATATTCATTTAACTCACCTTTATTTACTAAGGATATACAAGGTCATTCATCAAAT

21 TTTGAATATTCATTTAACTCACCTTTATTTACTAAGGATATACAAGGTCATTCATCAAAT

9 TTTGAATATTCATTTAACTCACCTTTATTTACTAAGGATATACAAGGTCATTCATCAAAT

6 TTTGAATATTCATTTAACTCACCTTTATTTACTAAGGATATACAAGGTCATTCATCAAAT

5 TTTGAATATTCATTTAACTCACCTTTATTTACTAAGGATATACAAGGTCATTCATCAAAT

4 TTTGAATATTCATTTAACTCACCTTTATTTACTAAGGATATACAAGGTCATTCATCAAAT

3 TTTGAATATTCATTTAACTCACCTTTATTTACTAAGGATATACAAGGTCATTCATCAAAT

20 TTTGAATATTCATTTAACTCACCTTTATTTACTAAGGATATACAAGGTCATTCATCAAAT

********** ********** * ***** ************ *********.*******

1RC GAAGAACACGATATGTCCACTTTCATGACGAAATCTGCAAATGTGGATGATGATCTTGGT

16RC GAAGAACACGATATGTCCACTTTCATGACGAAATCTGCAAATGTGGATGATGATCTTGGT

15RC GAAGAACACGATATGTCCACTTTCATGACGAAATCTGCAAATGTGGATGATGATCTTGGT

2RC GAAGAACACGATATGTCCACTTTCATGACGAAATCTGCAAATGTGGATGATGATCTTGGT

10 GAAGATTACGATATGTCCACTTTCATGACGAAATCTGCAAATGTGGATGATGATGTTGGT

17 GAAGATTACGATATGTCCACTTTCATGACGAAATCTGCAAATGTGGATGATGATGTTGGT

14 GAAGATTACGATATGTCCACTTTCATGACGAAATCTGCAAATGTGGATGATGATGTTGGT

13 GAAGATTACGATATGTCCACTTTCATGACGAAATCTGCAAATGTGGATGATGATGTTGGT

12 GAAGATTACGATATGTCCACTTTCATGACGAAATCTGCAAATGTGGATGATGATGTTGGT

8 GAAGATTACGATATGTCCACTTTCATGACGAAATCTGCAAATGTGGATGATGATGTTGGT

7 GAAGATTACGATATGTCCACTTTCATGACGAAATCTGCAAATGTGGATGATGATGTTGGT

19 GAAGATTACGATATGTCCACTTTCATGACGAAATCTGCAAATGTGGATGATGATGTTGGT

18 GAAGATTACGATATGTCCACTTTCATGACGAAATCTGCAAATGTGGATGATGATGTTGGT

22 GAAGATTACGATATGTCCACTTTCATGACGAAATCTGCAAATGTGGATGATGATGTTGGT

21 GAAGATTACGATATGTCCACTTTCATGACGAAATCTGCAAATGTGGATGATGATGTTGGT

9 GAAGATTACGATATGTCCACTTTCATGACGAAATCTGCAAATGTGGATGATGATGTTGGT

6 GAAGATTACGATATGTCCACTTTCATGACGAAATCTGCAAATGTGGATGATGATGTTGGT

5 GAAGATTACGATATGTCCACTTTCATGACGAAATCTGCAAATGTGGATGATGATGTTGGT

4 GAAGATTACGATATGTCCACTTTCATGACGAAATCTGCAAATGTGGATGATGATGTTGGT

3 GAAGATTACGATATGTCCACTTTCATGACGAAATCTGCAAATGTGGATGATGATGTTGGT

20 GAAGATTACGATATGTCCACTTTCATGACGAAATCTGCAAATGTGGATGATGATGTTGGT

***** .*********************************************** *****

1RC CAGTATAGTACACAAAAGGCCGTTAATATAGACGAGGGCGATATAGATTATGACTTAGCA

16RC CAGTATAGTACACAAAAGGCCGTTAATATAGACGAGGGCGATATAGATTATGACTTAGCA

15RC CAGTATAGTACACAAAAGGCCGTTAATATAGACGAGGGCGATATAGATTATGACTTAGCA

2RC CAGTATAGTACACAAAAGGCCGTTAATATAGACGAGGGCGATATAGATTATGACTTAGCA

10 CAATATAGTACACAGAAGGCCGTTAATATAGACGAGGGCGATATAGATTATGACTTAGCA

17 CAATATAGTACACAGAAGGCCGTTAATATAGACGAGGGCGATATAGATTATGACTTAGCA

14 CAATATAGTACACAGAAGGCCGTTAATATAGACGAGGGCGATATAGATTATGACTTAGCA

13 CAATATAGTACACAGAAGGCCGTTAATATAGACGAGGGCGATATAGATTATGACTTAGCA

12 CAATATAGTACACAGAAGGCCGTTAATATAGACGAGGGCGATATAGATTATGACTTAGCA

8 CAATATAGTACACAGAAGGCCGTTAATATAGACGAGGGCGATATAGATTATGACTTAGCA

7 CAATATAGTACACAGAAGGCCGTTAATATAGACGAGGGCGATATAGATTATGACTTAGCA

19 CAATATAGTACACAGAAGGCCGTTAATATAGACGAGGGCGATATAGATTATGATTTAGCA

18 CAATATAGTACACAGAAGGCCGTTAATATAGACGAGGGCGATATAGATTATGATTTAGCA

22 CAATATAGTACACAGAAGGCCGTTAATATAGACGAGGGCGATATAGATTATGACTTAGCA

21 CAATATAGTACACAGAAGGCCGTTAATATAGACGAGGGCGATATAGATTATGACTTAGCA

9 CAATATAGTACACAGAAGGCCGTTAATATAGACGAGGGCGATATAGATTATGACTTAGCA

6 CAATATAGTACACAGAAGGCCGTTAATATAGACGAGGGCGATATAGATTATGACTTAGCA

5 CAATATAGTACACAGAAGGCCGTTAATATAGACGAGGGCGATATAGATTATGACTTAGCA

4 CAATATAGTACACAGAAGGCCGTTAATATAGACGAGGGCGATATAGATTATGACTTAGCA

3 CAATATAGTACACAGAAGGCCGTTAATATAGACGAGGGCGATATAGATTATGACTTAGCA

20 CAATATAGTACACAGAAGGCCGTTAATATAGACGAGGGCGATATAGATTATGACTTAGCA

**.***********.**************************************.******

1RC AATAGGAATAGGAATAAAGAACGGCAGGTACTTCAAATGGACACACAGAGTACCAAAGAA

16RC AATAGGAATAGGAATAAAGAACGGCAGGTACTTCAAATGGACACACAGAGTACCAAAGAA

15RC AATAGGAATAGGAATAAAGAACGGCAGGTACTTCAAATGGACACACAGAGTACCAAAGAA

2RC AATAGGAATAGGAATAAAGAACGGCAGGTACTTCAAATGGACACACAGAGTACCAAAGAA

10 AATAGGGATAGGAATAATAATCGGCAGGTACTTCAAATGGACACACAGAGTACCAAAGAA

17 AATAGGGATAGGAATAATAATCGGCAGGTACTTCAAATGGACACACAGAGTACCAAAGAA

14 AATAGGGATAGGAATAATAATCGGCAGGTACTTCAAATGGACACACAGAGTACCAAAGAA

13 AATAGGGATAGGAATAATAATCGGCAGGTACTTCAAATGGACACACAGAGTACCAAAGAA

12 AATAGGGATAGGAATAATAATCGGCAGGTACTTCAAATGGACACACAGAGTACCAAAGAA

8 AATAGGGATAGGAATAATAATCGGCAGGTACTTCAAATGGACACACAGAGTACCAAAGAA

7 AATAGGGATAGGAATAATAATCGGCAGGTACTTCAAATGGACACACAGAGTACCAAAGAA

19 AATAGGGATAGGAATAATAATCGGCAGGTACTTCAAATGGACACACAGAGTACCAAAGAA

18 AATAGGGATAGGAATAATAATCGGCAGGTACTTCAAATGGACACACAGAGTACCAAAGAA

22 AATAGGGATAGGAATAATAATCGGCAGGTACTTCAAATGGACACACAGAGTACCAAAGAA

21 AATAGGGATAGGAATAATAATCGGCAGGTACTTCAAATGGACACACAGAGTACCAAAGAA

9 AATAGGGATAGGAATAATAATCGGCAGGTACTTCAAATGGACACACAGAGTACCAAAGAA

6 AATAGGGATAGGAATAATAATCGGCAGGTACTTCAAATGGACACACAGAGTACCAAAGAA

5 AATAGGGATAGGAATAATAATCGGCAGGTACTTCAAATGGACACACAGAGTACCAAAGAA

4 AATAGGGATAGGAATAATAATCGGCAGGTACTTCAAATGGACACACAGAGTACCAAAGAA

3 AATAGGGATAGGAATAATAATCGGCAGGTACTTCAAATGGACACACAGAGTACCAAAGAA

20 AATAGGGATAGGAATAATAATCGGCAGGTACTTCAAATGGACACACAGAGTACCAAAGAA

******.********** .* ***************************************

1RC TTAGAGGAAATGTATAAAGACAGAATCAACGCTGAAGCAGCTTGGAGTGAGTCTGCAAAC

16RC TTAGAGGAAATGTATAAAGACAGAATCAACGCTGAAGCAGCTTGGAGTGAGTCTGCAAAC

15RC TTAGAGGAAATGTATAAAGACAGAATCAACGCTGAAGCAGCTTGGAGTGAGTCTGCAAAC

2RC TTAGAGGAAATGTATAAAGACAGAATCAACGCTGAAGCAGCTTGGAGTGAGTCTGCAAAC

10 TTAAGGGAAATGCATAAAGACAGAATCAACGCTGAAGCGGGTTGGAGTGAGTCTGCAGAC

17 TTAAGGGAAATGCATAAAGACAGAATCAACGCTGAAGCGGGTTGGAGTGAGTCTGCAGAC

14 TTAAGGGAAATGCATAAAGACAGAATCAACGCTGAAGCGGGTTGGAGTGAGTCTGCAGAC

13 TTAAGGGAAATGCATAAAGACAGAATCAACGCTGAAGCGGGTTGGAGTGAGTCTGCAGAC

12 TTAAGGGAAATGCATAAAGACAGAATCAACGCTGAAGCGGGTTGGAGTGAGTCTGCAGAC

8 TTAAGGGAAATGCATAAAGACAGAATCAACGCTGAAGCGGGTTGGAGTGAGTCTGCAGAC

7 TTAAGGGAAATGCATAAAGACAGAATCAACGCTGAAGCGGGTTGGAGTGAGTCTGCAGAC

19 TTAAGGGAAATGCATAAAGACAGAATCAACGCTGAAGCGGGTTGGAGTGAGTCTGCAGAC

18 TTAAGGGAAATGCATAAAGACAGAATCAACGCTGAAGCGGGTTGGAGTGAGTCTGCAGAC

22 TTAAGGGAAATGCATAAAGACAGAATCAACGCTGAAGCGGGTTGGAGTGAGTCTGCAGAC

21 TTAAGGGAAATGCATAAAGACAGAATCAACGCTGAAGCGGGTTGGAGTGAGTCTGCAGAC

9 TTAAGGGAAATGCATAAAGACAGAATCAACGCTGAAGCGGGTTGGAGTGAGTCTGCAGAC

6 TTAAGGGAAATGCATAAAGACAGAATCAACGCTGAAGCGGGTTGGAGTGAGTCTGCAGAC

5 TTAAGGGAAATGCATAAAGACAGAATCAACGCTGAAGCGGGTTGGAGTGAGTCTGCAGAC

4 TTAAGGGAAATGCATAAAGACAGAATCAACGCTGAAGCGGGTTGGAGTGAGTCTGCAGAC

3 TTAAGGGAAATGCATAAAGACAGAATCAACGCTGAAGCGGGTTGGAGTGAGTCTGCAGAC

20 TTAAGGGAAATGCATAAAGACAGAATCAACGCTGAAGCGGGTTGGAGTGAGTCTGCAGAC

***..*******.*************************.* ****************.**

1RC CACTTTTATGACATTGCTGATTAAATTTAGACCTCAAATCTCGTCTTAGAGGCGAATCTG

16RC CACTTTTATGACATTGCTGATTAAATTTAGACCTCAAATCTCGTCTTAGAGGCGAATCTG

15RC CACTTTTATGACATTGCTGATTAAATTTAGACCTCAAATCTCGTCTTAGAGGCGAATCTG

2RC CACTTTTATGACATTGCTGATTAAATTTAGACCTCAAATCTCGTCTTAGAGGCGAATCTG

10 CACTTTTGTGACATCGCTGATTATATTTAGACCTCAAATCTCGTCTTAGAGGCGAATCTG

17 CACTTTTGTGACATCGCTGATTATATTTAGACCTCAAATCTCGTCTTAGAGGCGAATCTG

14 CACTTTTGTGACATCGCTGATTATATTTAGACCTCAAATCTCGTCTTAGAGGCGAATCTG

13 CACTTTTGTGACATCGCTGATTATATTTAGACCTCAAATCTCGTCTTAGAGGCGAATCTG

12 CACTTTTGTGACATCGCTGATTATATTTAGACCTCAAATCTCGTCTTAGAGGCGAATCTG

8 CACTTTTGTGACATCGCTGATTATATTTAGACCTCAAATCTCGTCTTAGAGGCGAATCTG

7 CACTTTTGTGACATCGCTGATTATATTTAGACCTCAAATCTCGTCTTAGAGGCGAATCTG

19 CACTTTTGTGACATCGCTGATTATATTTAGACCTCAAATCTCGTCTTAGAGGCGAATCTG

18 CACTTTTGTGACATCGCTGATTATATTTAGACCTCAAATCTCGTCTTAGAGGCGAATCTG

22 CACTTTTGTGACATCGCTGATTATATTTAGACCTCAAATCTCGTCTTAGAGGCGAATCTG

21 CACTTTTGTGACATCGCTGATTATATTTAGACCTCAAATCTCGTCTTAGAGGCGAATCTG

9 CACTTTTGTGACATCGCTGATTATATTTAGACCTCAAATCTCGTCTTAGAGGCGAATCTG

6 CACTTTTGTGACATCGCTGATTATATTTAGACCTCAAATCTCGTCTTAGAGGCGAATCTG

5 CACTTTTGTGACATCGCTGATTATATTTAGACCTCAAATCTCGTCTTAGAGGCGAATCTG

4 CACTTTTGTGACATCGCTGATTATATTTAGACCTCAAATCTCGTCTTAGAGGCGAATCTG

3 CACTTTTGTGACATCGCTGATTATATTTAGACCTCAAATCTCGTCTTAGAGGCGAATCTG

20 CACTTTTGTGACATCGCTGATTATATTTAGACCTCAAATCTCGTCTTAGAGGCGAATCTG

*******.******.******** ************************************

1RC GTAGGCAATACATTCAGCGATACGAATCTTCAAAGAAAACGTCGGATCAAAGCAACGACA

16RC GTAGGCAATACATTCAGCGATACGAATCTTCAAAGAAAACGTCGGATCAAAGCAACGACA

15RC GTAGGCAATACATTCAGCGATACGAATCTTCAAAGAAAACGTCGGATCAAAGCAACGACA

2RC GTAGGCAATACATTCAGCGATACGAATCTTCAAAGAAAACGTCGGATCAAAGCAACGACA

10 GTAGGCAATACATTCAGCGATACGAATCTTCAAAGAAAACGTCGGATCAAAGCAACGACA

17 GTAGGCAATACATTCAGCGATACGAATCTTCAAAGAAAACGTCGGATCAAAGCAACGACA

14 GTAGGCAATACATTCAGCGATACGAATCTTCAAAGAAAACGTCGGATCAAAGCAACGACA

13 GTAGGCAATACATTCAGCGATACGAATCTTCAAAGAAAACGTCGGATCAAAGCAACGACA

12 GTAGGCAATACATTCAGCGATACGAATCTTCAAAGAAAACGTCGGATCAAAGCAACGACA

8 GTAGGCAATACATTCAGCGATACGAATCTTCAAAGAAAACGTCGGATCAAAGCAACGACA

7 GTAGGCAATACATTCAGCGATACGAATCTTCAAAGAAAACGTCGGATCAAAGCAACGACA

19 GTAGGCAATACATTCAGCGATACGAATCTTCAAAGAAAACGTCGGATCAAAGCAACGACA

18 GTAGGCAATACATTCAGCGATACGAATCTTCAAAGAAAACGTCGGATCAAAGCAACGACA

22 GTAGGCAATACATTCAGCGATACGAATCTTCAAAGAAAACGTCGGATCAAAGCAACGACA

21 GTAGGCAATACATTCAGCGATACGAATCTTCAAAGAAAACGTCGGATCAAAGCAACGACA

9 GTAGGCAATACATTCAGCGATACGAATCTTCAAAGAAAACGTCGGATCAAAGCAACGACA

6 GTAGGCAATACATTCAGCGATACGAATCTTCAAAGAAAACGTCGGATCAAAGCAACGACA

5 GTAGGCAATACATTCAGCGATACGAATCTTCAAAGAAAACGTCGGATCAAAGCAACGACA

4 GTAGGCAATACATTCAGCGATACGAATCTTCAAAGAAAACGTCGGATCAAAGCAACGACA

3 GTAGGCAATACATTCAGCGATACGAATCTTCAAAGAAAACGTCGGATCAAAGCAACGACA

20 GTAGGCAATACATTCAGCGATACGAATCTTCAAAGAAAACGTCGGATCAAAGCAACGACA

************************************************************

1RC ACGACTTTTTCGACAGTGTAAGCAACACTCCGGCTTTGATAGGAATTCAAACAAACAATA

16RC ACGACTTTTTCGACAGTGTAAGCAACACTCCGGCTTTGATAGGAATTCAAACAAACAATA

15RC ACGACTTTTTCGACAGTGTAAGCAACACTCCGGCTTTGATAGGAATTCAAACAAACAATA

2RC ACGACTTTTTCGACAGTGTAAGCAACACTCCGGCTTTGATAGGAATTCAAACAAACAATA

10 ACCACTTTTTCGACAGTATAAGCAACACTCCGGCTTTGATAGGAATCCAAACAAACGATA

17 ACCACTTTTTCGACAGTATAAGCAACACTCCGGCTTTGATAGGAATCCAAACAAACAATA

14 ACCACTTTTTCGACAGTATAAGCAACACTCCGGCTTTGATAGGAATCCAAACAAACAATA

13 ACCACTTTTTCGACAGTATAAGCAACACTCCGGCTTTGATAGGAATCCAAACAAACAATA

12 ACCACTTTTTCGACAGTATAAGCAACACTCCGGCTTTGATAGGAATCCAAACAAACAATA

8 ACCACTTTTTCGACAGTATAAGCAACACTCCGGCTTTGATAGGAATCCAAACAAACAATA

7 ACCACTTTTTCGACAGTATAAGCAACACTCCGGCTTTGATAGGAATCCAAACAAACAATA

19 ACCACTTTTTCGACAGTATAAGCAACACTCCGGCTTTGATAGGAATCCAAACAAACAATA

18 ACCACTTTTTCGACAGTATAAGCAACACTCCGGCTTTGATAGGAATCCAAACAAACAATA

22 ACCACTTTTTCGACAGTATAAGCAACACTCCGGCTTTGATAGGAATCCAAACAAACGATA

21 ACCACTTTTTCGACAGTATAAGCAACACTCCGGCTTTGATAGGAATCCAAACAAACAATA

9 ACCACTTTTTCGACAGTATAAGCAACACTCCGGCTTTGATAGGAATCCAAACAAACAATA

6 ACCACTTTTTCGACAGTATAAGCAACACTCCGGCTTTGATAGGAATCCAAACAAACAATA

5 ACCACTTTTTCGACAGTATAAGCAACACTCCGGCTTTGATAGGAATCCAAACAAACAATA

4 ACCACTTTTTCGACAGTATAAGCAACACTCCGGCTTTGATAGGAATCCAAACAAACAATA

3 ACCACTTTTTCGACAGTATAAGCAACACTCCGGCTTTGATAGGAATCCAAACAAACAATA

20 ACCACTTTTTCGACAGTATAAGCAACACTCCGGCTTTGATAGGAATCCAAACAAACAATA

** **************.****************************.*********.***

1RC AGGACGACAGAGTCATACCACAAGCTGACGATTCGTCAACTGTCGTT---CAAGGTATCG

16RC AGGACGACAGAGTCATACCACAAGCTGACGATTCGTCAACTGTCGTT---CAAGGTATCG

15RC AGGACGACAGAGTCATACCACAAGCTGACGATTCGTCAACTGTCGTT---CAAGGTATCG

2RC AGGACGACAGAGTCATACCACAAGCTGACGATTCGTCAACTGTCGTT---CAAGGTATCG

10 AGGACGACAAAGTCATACCAGAAGCTAACGAATCGTCAACAGTCGTTCCACAAGGCATCG

17 AGGACGACAAAGTCATACCAGAAGCTAACGAATCGTCAACAGTCGTTCCACAAGGCATCG

14 AGGACGACAAAGTCATACCAGAAGCTAACGAATCGTCAACAGTCGTTCCACAAGGCATCG

13 AGGACGACAAAGTCATACCAGAAGCTAACGAATCGTCAACAGTCGTTCCACAAGGCATCG

12 AGGACGACAAAGTCATACCAGAAGCTAACGAATCGTCAACAGTCGTTCCACAAGGCATCG

8 AGGACGACAAAGTCATACCAGAAGCTAACGAATCGTCAACAGTCGTTCCACAAGGCATCG

7 AGGACGACAAAGTCATACCAGAAGCTAACGAATCGTCAACAGTCGTTCCACAAGGCATCG

19 AGGACGACAAAGTCATACCAGAAGCTAACGAATCGTCAACAGTCGTTCCACAAGGCATCG

18 AGGACGACAAAGTCATACCAGAAGCTAACGAATCGTCAACAGTCGTTCCACAAGGCATCG

22 AGGACGACAAAGTCATACCAGAAGCTAACGAATCGTCAACAGTCGTTCCACAAGGCATCG

21 AGGACGACAAAGTCATACCAGAAGCTAACGAATCGTCAACAGTCGTTCCACAAGGCATCG

9 AGGACGACAAAGTCATACCAGAAGCTAACGAATCGTCAACAGTCGTTCCACAAGGCATCG

6 AGGACGACAAAGTCATACCAGAAGCTAACGAATCGTCAACAGTCGTTCCACAAGGCATCG

5 AGGACGACAAAGTCATACCAGAAGCTAACGAATCGTCAACAGTCGTTCCACAAGGCATCG

4 AGGACGACAAAGTCATACCAGAAGCTAACGAATCGTCAACAGTCGTTCCACAAGGCATCG

3 AGGACGACAAAGTCATACCAGAAGCTAACGAATCGTCAACAGTCGTTCCACAAGGCATCG

20 AGGACGACAAAGTCATACCAGAAGCTAACGAATCGTCAACAGTCGTTCCACAAGGCATCG

*********.********** *****.**** ******** ****** *****.****

1RC CAAAAGATCAAGATTTTCTGGATAATTTGATTTA--AATGCATGATAATAATTGAA--AA

16RC CAAAAGATCAAGATTTTCTGGATAATTTGATTTA--AATGCATGATAATAATTGAAATAA

15RC CAAAAGATCAAGATTTTCTGGATAATTTGATTTA--AATGCATGATAATAATTGAAATAA

2RC CAAAAGATCAAGATTTTCTGGATAATTTGATTTA--AATGCATGATAATAATTGAAATAA

10 CAGAAGATCAAGATTTTCTGGATAATTTGATTTAATAATGCATGATAATAATTGAA--AT

17 CAGAAGATCAAGATTTTCTGGATAATTTGATTTAATAATGCATGATAATAATTGAA--AT

14 CAGAAGATCAAGATTTTCTGGATAATTTGATTTAATAATGCATGATAATAATTGAA--AT

13 CAGAAGATCAAGATTTTCTGGATAATTTGATTTAATAATGCATGATAATAATTGAA--AT

12 CAGAAGATCAAGATTTTCTGGATAATTTGATTTAATAATGCATGATAATAATTGAA--AT

8 CAGAAGATCAAGATTTTCTGGATAATTTGATTTAATAATGCATGATAATAATTGAA--AT

7 CAGAAGATCAAGATTTTCTGGATAATTTGATTTAATAATGCATGATAATAATTGAA--AT

19 CAGAAGATCAAGATTTTCTGGATAATTTGATTTAATAATGCATGATAATAATTGAA--AT

18 CAGAAGATCAAGATTTTCTGGATAATTTGATTTAATAATGCATGATAATAATTGAA--AT

22 CAGAAGATCAAGATTTTCTGGATAATTTGATTTAATAATGCATGATAATAATTGAA--AT

21 CAGAAGATCAAGATTTTCTGGATAATTTGATTTAATAATGCATGATAATAATTGAA--AT

9 CAGAAGATCAAGATTTTCTGGATAATTTGATTTAATAATGCATGATAATAATTGAA--AT

6 CAGAAGATCAAGATTTTCTGGATAATTTGATTTAATAATGCATGATAATAATTGAA--AT

5 CAGAAGATCAAGATTTTCTGGATAATTTGATTTAATAATGCATGATAATAATTGAA--AT

4 CAGAAGATCAAGATTTTCTGGATAATTTGATTTAATAATGCATGATAATAATTGAA--AT

3 CAGAAGATCAAGATTTTCTGGATAATTTGATTTAATAATGCATGATAATAATTGAA--AT

20 CAGAAGATCAAGATTTTCTGGATAATTTGATTTAATAATGCATGATAATAATTGAA--AT

**.******************************* ******************** *

1RC AAAAAAAACGAGCCAAACGAGTGATTTAGTGAATCAAACGTCTCGATTGATAAATAGTTC

16RC ATAAAAAACGAGCCAAACGAGTGATTTAGTGAATCAAACGTCTCGATTGATAAATAGTTC

15RC GAAAAAAACGAGCCAAACGAGTGATTTAGTGAATCAAACGTCTCGATTGATAAATAGTTC

2RC GAAAAAAACGAGCCAAACGAGTGATTTAGTGAATCAAACGTCTCGATTGATAAATAGTTC

10 AGAAGATACGAGCTAAACAAGTGATTTAGTGAATCAATCGTCTCGATTGATAAATAGTTC

17 AGAAGATACGAGCTAAACAAGTGATTTAGTGAATCAATCGTCTCGATTGATAAATAGTTC

14 AGAAGATACGAGCTAAACAAGTGATTTAGTGAATCAATCGTCTCGATTGATAAATAGTTC

13 AGAAGATACGAGCTAAACAAGTGATTTAGTGAATCAATCGTCTCGATTGATAAATAGTTC

12 AGAAGATACGAGCTAAACAAGTGATTTAGTGAATCAATCGTCTCGATTGATAAATAGTTC

8 AGAAGATACGAGCTAAACAAGTGATTTAGTGAATCAATCGTCTCGATTGATAAATAGTTC

7 AGAAGATACGAGCTAAACAAGTGATTTAGTGAATCAATCGTCTCGATTGATAAATAGTTC

19 AGAAGATACGAGCTAAACAAGTGATTTAGTGAATCAATCGTCTCGATTGATAAATAGTTC

18 AGAAGATACGAGCTAAACAAGTGATTTAGTGAATCAATCGTCTCGATTGATAAATAGTTC

22 AGAAGATACGAGCTAAACAAGTGATTTAGTGAATCAATCGTCTCGATTGATAAATAGTTC

21 AGAAGATACGAGCTAAACAAGTGATTTAGTGAATCAATCGTCTCGATTGATAAATAGTTC

9 AGAAGATACGAGCTAAACAAGTGATTTAGTGAATCAATCGTCTCGATTGATAAATAGTTC

6 AGAAGATACGAGCTAAACAAGTGATTTAGTGAATCAATCGTCTCGATTGATAAATAGTTC

5 AGAAGATACGAGCTAAACAAGTGATTTAGTGAATCAATCGTCTCGATTGATAAATAGTTC

4 AGAAGATACGAGCTAAACAAGTGATTTAGTGAATCAATCGTCTCGATTGATAAATAGTTC

3 AGAAGATACGAGCTAAACAAGTGATTTAGTGAATCAATCGTCTCGATTGATAAATAGTTC

20 AGAAGATACGAGCTAAACAAGTGATTTAGTGAATCAATCGTCTCGATTGATAAATAGTTC

. **.* ******.****.****************** **********************

1RC AAGACATCATTACGGTATTGAGATATATTCATTTGTAGATACAATTTGCAATTATTTATA

16RC AAGACATCATTACGGTATTGAGATATATTCATTTGTAGATACAATTTGCAATTATTTATA

15RC AAGACATCATTACGGTATTGAGATATATTCATTTGTAGATACAATTTGCAATTATTTATA

2RC AAGACATCATTACGGTATTGAGATATATTCATTTGTAGATACAATTTGCAATTATTTATA

10 AAGACGTCATTACGGTATTGAGATATATTCATTTGTAGATATAGTTTGCAATTATTGATA

17 AAGACGTCATTACGGTATTGAGATATATTCATTTGTAGATATAGTTTGCAATTATTGATC

14 AAGACGTCATTACGGTATTGAGATATATTCATTTGTAGATATAGTTTGCAATTATTGATC

13 AAGACGTCATTACGGTATTGAGATATATTCATTTGTAGATATAGTTTGCAATTATTGATC

12 AAGACGTCATTACGGTATTGAGATATATTCATTTGTAGATATAGTTTGCAATTATTGATC

8 AAGACGTCATTACGGTATTGAGATATATTCATTTGTAGATATAGTTTGCAATTATTGATC

7 AAGACGTCATTACGGTATTGAGATATATTCATTTGTAGATATAGTTTGCAATTATTGATC

19 AAGACGTCATTACGGTATTGAGATATATTCATTTGTAGATATAGTTTGCAATTATTGATC

18 AAGACGTCATTACGGTATTGAGATATATTCATTTGTAGATATAGTTTGCAATTATTGATC

22 AAGACGTCATTACGGTATTGAGATATATTCATTTGTAGATATAGTTTGCAATTATTGATA

21 AAGACGTCATTACGGTATTGAGATATATTCATTTGTAGATATAGTTTGCAATTATTGATA

9 AAGACGTCATTACGGTATTGAGATATATTCATTTGTAGATATAGTTTGCAATTATTGATA

6 AAGACGTCATTACGGTATTGAGATATATTCATTTGTAGATATAGTTTGCAATTATTGATA

5 AAGACGTCATTACGGTATTGAGATATATTCATTTGTAGATATAGTTTGCAATTATTGATA

4 AAGACGTCATTACGGTATTGAGATATATTCATTTGTAGATATAGTTTGCAATTATTGATA

3 AAGACGTCATTACGGTATTGAGATATATTCATTTGTAGATATAGTTTGCAATTATTGATA

20 AAGACGTCATTACGGTATTGAGATATATTCATTTGTAGATATAGTTTGCAATTATTGATA

*****.***********************************.*.************ **

1RC TATCATGCAAGAGCATCTTAATATTAACGTAGATGCTTTTTTCTCTTTCAGCCTCTCTTT

16RC TATCATGCAAGAGCATCTTAATATTAACGTAGATGCTTTTTTCTCTTTCAGCCTCTCTTT

15RC TATCATGCAAGAGCATCTTAATATTAACGTAGATGCTTTTTTCTCTTTCAGCCTCTCTTT

2RC TATCATGCAAGAGCATCTTAATATTAACGTAGATGCTTTTTTCTCTTTCAGCCTCTCTTT

10 TATCATGCAAGAGCATCTTGGTATTGACTTAGATCTTTGGTTCTCTTTCAGCGTCTCTTT

17 TATCATGCAAGAGCATCTTGGTATTGACTTAGATCTTTGGTTCTCTTTCAGCGTCTCTTT

14 TATCATGCAAGAGCATCTTGGTATTGACTTAGATCTTTGGTTCTCTTTCAGCGTCTCTTT

13 TATCATGCAAGAGCATCTTGGTATTGACTTAGATCTTTGGTTCTCTTTCAGCGTCTCTTT

12 TATCATGCAAGAGCATCTTGGTATTGACTTAGATCTTTGGTTCTCTTTCAGCGTCTCTTT

8 TATCATGCAAGAGCATCTTGGTATTGACTTAGATCTTTGGTTCTCTTTCAGCGTCTCTTT

7 TATCATGCAAGAGCATCTTGGTATTGACTTAGATCTTTGGTTCTCTTTCAGCGTCTCTTT

19 TATCATGCAAGAGCATCTTGGTATTGACTTAGATCTTTGGTTCTCTTTCAGCGTCTCTTT

18 TATCATGCAAGAGCATCTTGGTATTGACTTAGATCTTTGGTTCTCTTTCAGCGTCTCTTT

22 TATCATGCAAGAGCATCTTGGTATTGACTTAGATCTTTGGTTCTCTTTCAGCGTCTCTTT

21 TATCATGCAAGAGCATCTTGGTATTGACTTAGATCTTTGGTTCTCTTTCAGCGTCTCTTT

9 TATCATGCAAGAGCATCTTGGTATTGACTTAGATCTTTGGTTCTCTTTCAGCGTCTCTTT

6 TATCATGCAAGAGCATCTTGGTATTGACTTAGATCTTTGGTTCTCTTTCAGCGTCTCTTT

5 TATCATGCAAGAGCATCTTGGTATTGACTTAGATCTTTGGTTCTCTTTCAGCGTCTCTTT

4 TATCATGCAAGAGCATCTTGGTATTGACTTAGATCTTTGGTTCTCTTTCAGCGTCTCTTT

3 TATCATGCAAGAGCATCTTGGTATTGACTTAGATCTTTGGTTCTCTTTCAGCGTCTCTTT

20 TATCATGCAAGAGCATCTTGGTATTGACTTAGATCTTTGGTTCTCTTTCAGCGTCTCTTT

*******************..****.** ***** .** ************ *******

**HMG<<<<<<<<<<<<<<<<<<<<<<<<<<<<<**

1RC CAGCCTTTGCAATGCGGAAAGC-TTCTGGTATGGCTGCAATGAACTTAGAATACATATCG

16RC CAGCCTTTGCAATGCGGAAAGC-TTCTGGTATGGCTGCAATGAACTTAGAATACATATCG

15RC CAGCCTTTGCAATGCGGAAAGC-TTCTGGTATGGCTGCAATGAACTTAGAATACATATCG

2RC CAGCCTTTGCAATGCGGAAAGC-TTCTGGTATGGCTGCAATGAACTTAGAATACATATCG

10 CAGCCTTTGCAATGCGGAAAGCTTTCTGGTATGGCTGTAATGAAATTAGAATATATATCG

17 CAGCCTTTGCAATGCGGAAAGCTTTCTGGTATGGCTGTAATGAAATTAGAATATATATCG

14 CAGCCTTTGCAATGCGGAAAGCTTTCTGGTATGGCTGTAATGAAATTAGAATATATATCG

13 CAGCCTTTGCAATGCGGAAAGCTTTCTGGTATGGCTGTAATGAAATTAGAATATATATCG

12 CAGCCTTTGCAATGCGGAAAGCTTTCTGGTATGGCTGTAATGAAATTAGAATATATATCG

8 CAGCCTTTGCAATGCGGAAAGCTTTCTGGTATGGCTGTAATGAAATTAGAATATATATCG

7 CAGCCTTTGCAATGCGGAAAGCTTTCTGGTATGGCTGTAATGAAATTAGAATATATATCG

19 CAGCCTTTGCAATGCGGAAAGCTTTCTGGTATGGCTGTAATGAAATTAGAATATATATCG

18 CAGCCTTTGCAATGCGGAAAGCTTTCTGGTATGGCTGTAATGAAATTAGAATATATATCG

22 CAGCCTTTGCAATGCGGAAAGCTTTCTGGTATGGCTGTAATGAAATTAGAATATATATCG

21 CAGCCTTTGCAATGCGGAAAGCTTTCTGGTATGGCTGTAATGAAATTAGAATATATATCG

9 CAGCCTTTGCAATGCGGAAAGCTTTCTGGTATGGCTGTAATGAAATTAGAATATATATCG

6 CAGCCTTTGCAATGCGGAAAGCTTTCTGGTATGGCTGTAATGAAATTAGAATATATATCG

5 CAGCCTTTGCAATGCGGAAAGCTTTCTGGTATGGCTGTAATGAAATTAGAATATATATCG

4 CAGCCTTTGCAATGCGGAAAGCTTTCTGGTATGGCTGTAATGAAATTAGAATATATATCG

3 CAGCCTTTGCAATGCGGAAAGCTTTCTGGTATGGCTGTAATGAAATTAGAATATATATCG

20 CAGCCTTTGCAATGCGGAAAGCTTTCTGGTATGGCTGTAATGAAATTAGAATATATATCG

********************** **************.****** ********.******

**<<<<<<<<<<<<<<<<<<<<<<<<<<<<<<<<<<<<<<<<<<<<<<<<<<<<<<<<<<<<**

1RC AATAGCTCCAATTAATCTTAACTCTCTGACTTACCTTGAAAGATCCTTCTGGATTTGAGA

16RC AATAGCTCCAATTAATCTTAACTCTCTGACTTACCTTGAAAGATCCTTCTGGATTTGAGA

15RC AATAGCTCCAATTAATCTTAACTCTCTGACTTACCTTGAAAGATCCTTCTGGATTTGAGA

2RC AATAGCTCCAATTAATCTTAACTCTCTGACTTACCTTGAAAGATCCTTCTGGATTTGAGA

10 GTTAGCTTCAATGGCTCTTACCTCTTTATCTTCCTCTGAGAGATCCTTCCACATTTGAGA

17 GTTAGCTTCAATGGCTCTTACCTCTTTATCTTCCTCTGAGAGATCCTTCCACATTTGAGA

14 GTTAGCTTCAATGGCTCTTACCTCTTTATCTTCCTCTGAGAGATCCTTCCACATTTGAGA

13 GTTAGCTTCAATGGCTCTTACCTCTTTATCTTCCTCTGAGAGATCCTTCCACATTTGAGA

12 GTTAGCTTCAATGGCTCTTACCTCTTTATCTTCCTCTGAGAGATCCTTCCACATTTGAGA

8 GTTAGCTTCAATGGCTCTTACCTCTTTATCTTCCTCTGAGAGATCCTTCCACATTTGAGA

7 GTTAGCTTCAATGGCTCTTACCTCTTTATCTTCCTCTGAGAGATCCTTCCACATTTGAGA

19 GTTAGCTTCAATGGCTCTTACCTCTTTATCTTCCTCTGAGAGATCCTTCCACATTTGAGA

18 GTTAGCTTCAATGGCTCTTACCTCTTTATCTTCCTCTGAGAGATCCTTCCACATTTGAGA

22 GTTAGCTTCAATGGCTCTTACCTCTTTATCTTCCTCTGAGAGATCCTTCCACATTTGAGA

21 GTTAGCTTCAATGGCTCTTACCTCTTTATCTTCCTCTGAGAGATCCTTCCACATTTGAGA

9 GTTAGCTTCAATGGCTCTTACCTCTTTATCTTCCTCTGAGAGATCCTTCCACATTTGAGA

6 GTTAGCTTCAATGGCTCTTACCTCTTTATCTTCCTCTGAGAGATCCTTCCACATTTGAGA

5 GTTAGCTTCAATGGCTCTTACCTCTTTATCTTCCTCTGAGAGATCCTTCCACATTTGAGA

4 GTTAGCTTCAATGGCTCTTACCTCTTTATCTTCCTCTGAGAGATCCTTCCACATTTGAGA

3 GTTAGCTTCAATGGCTCTTACCTCTTTATCTTCCTCTGAGAGATCCTTCCACATTTGAGA

20 GTTAGCTTCAATGGCTCTTACCTCTTTATCTTCCTCTGAGAGATCCTTCCACATTTGAGA

. *****.**** . ***** ****.*. *** *..***.*********.. ********

**<<<<<<<<<<<<<<<<<<<<<<<<<<<<<<<<<<<<<<<<<<<<<<<<<<<<<<<<<<<<**

1RC AGCAGCCTTCATTGCATCTTTGGAGTCACCAAGGTCTACGGTGAGCTGCGGAATGATCTT

16RC AGCAGCCTTCATTGCATCTTTGGAGTCACCAAGGTCTACGGTGAGCTGCGGAATGATCTT

15RC AGCAGCCTTCATTGCATCTTTGGAGTCACCAAGGTCTACGGTGAGCTGCGGAATGATCTT

2RC AGCAGCCTTCATTGCATCTTTGGAGTCACCAAGGTCTACGGTGAGCTGCGGAATGATCTT

10 AGCAGCTTTCATTGCATGTTTGGAGTCACCAAGGTCTAAGGTGAGCTGTGGGATGATCTC

17 AGCAGCTTTCATTGCATGTTTGGAGTCACCAAGGTCTAAGGTGAGCTGTGGGATGATCTC

14 AGCAGCTTTCATTGCATGTTTGGAGTCACCAAGGTCTAAGGTGAGCTGTGGGATGATCTC

13 AGCAGCTTTCATTGCATGTTTGGAGTCACCAAGGTCTAAGGTGAGCTGTGGGATGATCTC

12 AGCAGCTTTCATTGCATGTTTGGAGTCACCAAGGTCTAAGGTGAGCTGTGGGATGATCTC

8 AGCAGCTTTCATTGCATGTTTGGAGTCACCAAGGTCTAAGGTGAGCTGTGGGATGATCTC

7 AGCAGCTTTCATTGCATGTTTGGAGTCACCAAGGTCTAAGGTGAGCTGTGGGATGATCTC

19 AGCAGCTTTCATTGCATGTTTGGAGTCACCAAGGTCTAAGGTGAGCTGTGGGATGATCTC

18 AGCAGCTTTCATTGCATGTTTGGAGTCACCAAGGTCTAAGGTGAGCTGTGGGATGATCTC

22 AGCAGCTTTCATTGCATGTTTGGAGTCACCAAGGTCTAAGGTGAGCTGTGGGATGATCTC

21 AGCAGCTTTCATTGCATGTTTGGAGTCACCAAGGTCTAAGGTGAGCTGTGGGATGATCTC

9 AGCAGCTTTCATTGCATGTTTGGAGTCACCAAGGTCTAAGGTGAGCTGTGGGATGATCTC

6 AGCAGCTTTCATTGCATGTTTGGAGTCACCAAGGTCTAAGGTGAGCTGTGGGATGATCTC

5 AGCAGCTTTCATTGCATGTTTGGAGTCACCAAGGTCTAAGGTGAGCTGTGGGATGATCTC

4 AGCAGCTTTCATTGCATGTTTGGAGTCACCAAGGTCTAAGGTGAGCTGTGGGATGATCTC

3 AGCAGCTTTCATTGCATGTTTGGAGTCACCAAGGTCTAAGGTGAGCTGTGGGATGATCTC

20 AGCAGCTTTCATTGCATGTTTGGAGTCACCAAGGTCTAAGGTGAGCTGTGGGATGATCTC

******.********** ******************** *********.**.*******.

**<<<<<<<<<<<<<<<<<<<<<<<<<<<<<<<<<<<<<<<<<<<<<<<<<<<<<<<<<<<<**

1RC AGACATGTGATGGTTGCCAGCATTAGCGAGTATTTTAGTTCTAGTAGCCTTATCAGCTTG

16RC AGACATGTGACGGTTGCCAGCATTAGCGAGTATTTTAGTTCTAGTAGCCTTATCAGCTTG

15RC AGACATGTGACGGTTGCCAGCATTAGCGAGTATTTTAGTTCTAGTAGCCTTATCAGCTTG

2RC AGACATGTGACGGTTGCCAGCATTAGCGAGTATTTTAGTTCTAGTAGCCTTATCAGCTTG

10 AGACATGTAACGATTGCGAGCATTAGCGAGTCCTTTAGTTCTAGTAGCTTTATCGGCTTG

17 AGACATGTAACGATTGCGAGCATTAGCGAGTCCTTTAGTTCTAGTAGCTTTATCGGCTTG

14 AGACATGTAACGATTGCGAGCATTAGCGAGTCCTTTAGTTCTAGTAGCTTTATCGGCTTG

13 AGACATGTAACGATTGCGAGCATTAGCGAGTCCTTTAGTTCTAGTAGCTTTATCGGCTTG

12 AGACATGTAACGATTGCGAGCATTAGCGAGTCCTTTAGTTCTAGTAGCTTTATCGGCTTG

8 AGACATGTAACGATTGCGAGCATTAGCGAGTCCTTTAGTTCTAGTAGCTTTATCGGCTTG

7 AGACATGTAACGATTGCGAGCATTAGCGAGTCCTTTAGTTCTAGTAGCTTTATCGGCTTG

19 AGACATGTAACGATTGCGAGCATTAGCGAGTCCTTTAGTTCTAGTAGCTTTATCGGCTTG

18 AGACATGTAACGATTGCGAGCATTAGCGAGTCCTTTAGTTCTAGTAGCTTTATCGGCTTG

22 AGACATGTAACGATTGCGAGCATTAGCGAGTCCTTTAGTTCTAGTAGCTTTATCGGCTTG

21 AGACATGTAACGATTGCGAGCATTAGCGAGTCCTTTAGTTCTAGTAGCTTTATCGGCTTG

9 AGACATGTAACGATTGCGAGCATTAGCGAGTCCTTTAGTTCTAGTAGCTTTATCGGCTTG

6 AGACATGTAACGATTGCGAGCATTAGCGAGTCCTTTAGTTCTAGTAGCTTTATCGGCTTG

5 AGACATGTAACGATTGCGAGCATTAGCGAGTCCTTTAGTTCTAGTAGCTTTATCGGCTTG

4 AGACATGTAACGATTGCGAGCATTAGCGAGTCCTTTAGTTCTAGTAGCTTTATCGGCTTG

3 AGACATGTAACGATTGCGAGCATTAGCGAGTCCTTTAGTTCTAGTAGCTTTATCGGCTTG

20 AGACATGTAACGATTGCGAGCATTAGCGAGTCCTTTAGTTCTAGTAGCTTTATCGGCTTG

********.*.*.**** ************* .***************.*****.*****

**<<<<<<<<<<<<<<<<<<<<<<<<<<<<<<<<<<<<<<<<<<<<<<<<<<<<<<<<<<<<**

1RC CTTGGCTTCACGTTCAAATATTTTCATCACTTTGCGAAGCGCTACAGGTGTTGAAAGCTG

16RC CTTGGCTTCACGTTCAAATATTTTCATCACTTTGCGAAGCGCTACAGGTGTTGAAAGCTG

15RC CTTGGCTTCACGTTCAAATATTTTCATCACTTTGCGAAGCGCTACAGGTGTTGAAAGCTG

2RC CTTGGCTTCACGTTCAAATATTTTCATCACTTTGCGAAGCGCTACAGGTGTTGAAAGCTG

10 CTTAGCTTCACGTTCAGATCTGTTCATCTCTTTGCGATACTCTGCAGGGATTGAAAGCTG

17 CTTAGCTTCACGTTCAGATCTGTTCATCTCTTTGCGATACTCTGCAGGGATTGAAAGCTG

14 CTTAGCTTCACGTTCAGATCTGTTCATCTCTTTGCGATACTCTGCAGGGATTGAAAGCTG

13 CTTAGCTTCACGTTCAGATCTGTTCATCTCTTTGCGATACTCTGCAGGGATTGAAAGCTG

12 CTTAGCTTCACGTTCAGATCTGTTCATCTCTTTGCGATACTCTGCAGGGATTGAAAGCTG

8 CTTAGCTTCACGTTCAGATCTGTTCATCTCTTTGCGATACTCTGCAGGGATTGAAAGCTG

7 CTTAGCTTCACGTTCAGATCTGTTCATCTCTTTGCGATACTCTGCAGGGATTGAAAGCTG

19 CTTAGCTTCACGTTCAGATCTGTTCATCTCTTTGCGATACTCTGCAGGGATTGAAAGCTG

18 CTTAGCTTCACGTTCAGATCTGTTCATCTCTTTGCGATACTCTGCAGGGATTGAAAGCTG

22 CTTAGCTTCACGTTCAGATCTGTTCATCTCTTTGCGATACTCTGCAGGGATTGAAAGCTG

21 CTTAGCTTCACGTTCAGATCTGTTCATCTCTTTGCGATACTCTGCAGGGATTGAAAGCTG

9 CTTAGCTTCACGTTCAGATCTGTTCATCTCTTTGCGATACTCTGCAGGGATTGAAAGCTG

6 CTTAGCTTCACGTTCAGATCTGTTCATCTCTTTGCGATACTCTGCAGGGATTGAAAGCTG

5 CTTAGCTTCACGTTCAGATCTGTTCATCTCTTTGCGATACTCTGCAGGGATTGAAAGCTG

4 CTTAGCTTCACGTTCAGATCTGTTCATCTCTTTGCGATACTCTGCAGGGATTGAAAGCTG

3 CTTAGCTTCACGTTCAGATCTGTTCATCTCTTTGCGATACTCTGCAGGGATTGAAAGCTG

20 CTTAGCTTCACGTTCAGATCTGTTCATCTCTTTGCGATACTCTGCAGGGATTGAAAGCTG

***.************.** * ****** ******** .* **.**** .**********

**<<<<<<<<<<<<<<<<<<<<<<<<<<<<<<<<<<<<<<<<<<<<<<<<<<<<<<<<<<<<**

1RC CCTGGCGCCAGATTCTCTGCGAATGTTATTGAAGCGGCTAATCTCTGTAAACATCTTGAT

16RC CCTGGCGCCAGATTCTCTGCGAATGTTATTGAAGCGGCTAATCTCTGTAAACATCTTGAT

15RC CCTGGCGCCAGATTCTCTGCGAATGTTATTGAAGCGGCTAATCTCTGTAAACATCTTGAT

2RC CCTGGCGCCAGATTCTCTGCGAATGTTATTGAAGCGGCTAATCTCTGTAAACATCTTGAT

10 CTTGGCTCCAGATTCTCTGCGAATGTTATTGATGTGGCTGATGTCTGAGAAAGTCTTGGT

17 CTTGGCTCCAGATTCTCTGCGAATGTTATTGATGTGGCTGATGTCTGAGAAAGTCTTGGT

14 CTTGGCTCCAGATTCTCTGCGAATGTTATTGATGTGGCTGATGTCTGAGAAAGTCTTGGT

13 CTTGGCTCCAGATTCTCTGCGAATGTTATTGATGTGGCTGATGTCTGAGAAAGTCTTGGT

12 CTTGGCTCCAGATTCTCTGCGAATGTTATTGATGTGGCTGATGTCTGAGAAAGTCTTGGT

8 CTTGGCTCCAGATTCTCTGCGAATGTTATTGATGTGGCTGATGTCTGAGAAAGTCTTGGT

7 CTTGGCTCCAGATTCTCTGCGAATGTTATTGATGTGGCTGATGTCTGAGAAAGTCTTGGT

19 CTTGGCTCCAGATTCTCTGCGAATGTTATTGATGTGGCTGATGTCTGAGAAAGTCTTGGT

18 CTTGGCTCCAGATTCTCTGCGAATGTTATTGATGTGGCTGATGTCTGAGAAAGTCTTGGT

22 CTTGGCTCCAGATTCTCTGCGAATGTTATTGATGTGGCTGATGTCTGAGAAAGTCTTGGT

21 CTTGGCTCCAGATTCTCTGCGAATGTTATTGATGTGGCTGATGTCTGAGAAAGTCTTGGT

9 CTTGGCTCCAGATTCTCTGCGAATGTTATTGATGTGGCTGATGTCTGAGAAAGTCTTGGT

6 CTTGGCTCCAGATTCTCTGCGAATGTTATTGATGTGGCTGATGTCTGAGAAAGTCTTGGT

5 CTTGGCTCCAGATTCTCTGCGAATGTTATTGATGTGGCTGATGTCTGAGAAAGTCTTGGT

4 CTTGGCTCCAGATTCTCTGCGAATGTTATTGATGTGGCTGATGTCTGAGAAAGTCTTGGT

3 CTTGGCTCCAGATTCTCTGCGAATGTTATTGATGTGGCTGATGTCTGAGAAAGTCTTGGT

20 CTTGGCTCCAGATTCTCTGCGAATGTTATTGATGTGGCTGATGTCTGAGAAAGTCTTGGT

*.**** ************************* *.****.** **** .** .*****.*

**<<<<<<<<<<<<<<<<<<<<<<<<<<<<<<<<<<<<<<<<<<<<<<<<<<<<<<<<<<<<**

1RC CATCCAACAGTCTCTATTTCTTCAGGCTTGCACAGCTTGTAGGCTTCATCAT-GGCGGAC

16RC CATCCAACAGTCTCTATTTCTTCAGGCTTGCACAGCTTGTAGGCTTCATCAT-GGCGGAC

15RC CATCCAACAGTCTCTATTTCTTCAGGCTTGCACAGCTTGTAGGCTTCATCAT-GGCGGAC

2RC CATCCAACAGTCTCTATTTCTTCAGGCTTGCACAGCTTGTAGGCTTCATCAT-GGCGGAC

10 CATCCACCAGTCCC--TCTCTTCAGGCTTGTAAAGCTTGTAGGCATCATGGTAAGCGGAC

17 CATCCACCAGTTCC--TCTCTTCAGGCTTGTAAAGCTTGTAGGCATCATGGTAAGCGGAC

14 CATCCACCAGTTCC--TCTCTTCAGGCTTGTAAAGCTTGTAGGCATCATGGTAAGCGGAC

13 CATCCACCAGTTCC--TCTCTTCAGGCTTGTAAAGCTTGTAGGCATCATGGTAAGCGGAC

12 CATCCACCAGTTCC--TCTCTTCAGGCTTGTAAAGCTTGTAGGCATCATGGTAAGCGGAC

8 CATCCACCAGTTCC--TCTCTTCAGGCTTGTAAAGCTTGTAGGCATCATGGTAAGCGGAC

7 CATCCACCAGTTCC--TCTCTTCAGGCTTGTAAAGCTTGTAGGCATCATGGTAAGCGGAC

19 CATCCACCAGTTCC--TCTCTTCAGGCTTGTAAAGCTTGTAGGCATCATGGTAAGCGGAC

18 CATCCACCAGTTCC--TCTCTTCAGGCTTGTAAAGCTTGTAGGCATCATGGTAAGCGGAC

22 CATCCACCAGTCCC--TCTCTTCAGGCTTGTAAAGCTTGTAGGCATCATGGTAAGCGGAC

21 CATCCACCAGTCCC--TCTCTTCAGGCTTGTAAAGCTTGTAGGCATCATGGTAAGCGGAC

9 CATCCACCAGTCCC--TCTCTTCAGGCTTGTAAAGCTTGTAGGCATCATGGTAAGCGGAC

6 CATCCACCAGTCCC--TCTCTTCAGGCTTGTAAAGCTTGTAGGCATCATGGTAAGCGGAC

5 CATCCACCAGTCCC--TCTCTTCAGGCTTGTAAAGCTTGTAGGCATCATGGTAAGCGGAC

4 CATCCACCAGTCCC--TCTCTTCAGGCTTGTAAAGCTTGTAGGCATCATGGTAAGCGGAC

3 CATCCACCAGTCCC--TCTCTTCAGGCTTGTAAAGCTTGTAGGCATCATGGTAAGCGGAC

20 CATCCACCAGTCCC--TCTCTTCAGGCTTGTAAAGCTTGTAGGCATCATGGTAAGCGGAC

****** ****..* *.************.* *********** **** .* .******

**<<<<<<<<<<<<<<<<<<<<<<<<<<<<<<<<<<<<<<<<<<<<<<<<<<<<<<<<<<<<**

1RC TCATGGGCCTTTGGTTTGTTAGACACGTGAAAGAGTGAAAAATAG------------TTT

16RC TCATGGGCCTTTGGTTTGTTAGACACGTGAAAGAGTGAAAAATAG------------TTT

15RC TCATGGGCCTTTGGTTTGTTAGACACGTGAAAGAGTGAAAAATAG------------TTT

2RC TCATGGGCCTTTGGTTTGTTAGACACGTGAAAGAGTGAAAAATAG------------TTT

10 TCATATGGCTTTGGTTTGTAAGACAGATGAAAAAGTGACAAACAAAGACAAACCTCCTTT

17 TCATATGGCTTTGGTTTGTAAGACAGATGAAAAAGTGACAAACAAAGACAAACCTCCTTT

14 TCATATGGCTTTGGTTTGTAAGACAGATGAAAAAGTGACAAACAAAGACAAACCTCCTTT

13 TCATATGGCTTTGGTTTGTAAGACAGATGAAAAAGTGACAAACAAAGACAAACCTCCTTT

12 TCATATGGCTTTGGTTTGTAAGACAGATGAAAAAGTGACAAACAAAGACAAACCTCCTTT

8 TCATATGGCTTTGGTTTGTAAGACAGATGAAAAAGTGACAAACAAAGACAAACCTCCTTT

7 TCATATGGCTTTGGTTTGTAAGACAGATGAAAAAGTGACAAACAAAGACAAACCTCCTTT

19 TCATATGGCTTTGGTTTGTAAGACAGATGAAAAAGTGACAAACAAAGACAAACCTCCTTT

18 TCATATGGCTTTGGTTTGTAAGACAGATGAAAAAGTGACAAACAAAGACAAACCTCCTTT

22 TCATATGGCTTTGGTTTGTAAGACAGATGAAAAAGTGACAAACAAAGACAAACCTCCTTT

21 TCATATGGCTTTGGTTTGTAAGACAGATGAAAAAGTGACAAACAAAGACAAACCTCCTTT

9 TCATATGGCTTTGGTTTGTAAGACAGATGAAAAAGTGACAAACAAAGACAAACCTCCTTT

6 TCATATGGCTTTGGTTTGTAAGACAGATGAAAAAGTGACAAACAAAGACAAACCTCCTTT

5 TCATATGGCTTTGGTTTGTAAGACAGATGAAAAAGTGACAAACAAAGACAAACCTCCTTT

4 TCATATGGCTTTGGTTTGTAAGACAGATGAAAAAGTGACAAACAAAGACAAACCTCCTTT

3 TCATATGGCTTTGGTTTGTAAGACAGATGAAAAAGTGACAAACAAAGACAAACCTCCTTT

20 TCATATGGCTTTGGTTTGTAAGACAGATGAAAAAGTGACAAACAAAGACAAACCTCCTTT

****. * *********** ***** .*****.***** ***.*. ***

**<<<<<<<<<<<<<<<<<<<<<<<<<<<<<<<<<<<<<<<<<<<<<<<<<<<<<<<<<<<<**

1RC TTGGTCTCAAAAATGCTTTAACATGCAGGCAAAACAACCTTTTGACCGAA-TCAATTGCG

16RC TTGGTCTCAAAAATGCTTTAACATGCAGGCAAAACAACCTTTTGACCGAA-TCAATTGCG

15RC TTGGTCTCAAAAATGCTTTAACATGCAGGCAAAACAACCTTTTGACCGAA-TCAATTGCG

2RC TTGGTCTCAAAAATGCTTTAACATGCAGGCAAAACAACCTTTTGACCGAA-TCAATTGCG

10 TGAGTCTCAAAAAGGCTTTCCCATGCAGGTGAAGCAACTTTTTGACCGAATTCAATCGGA

17 TGAGTCTCAAAAAGGCTTTCCCATGCAGGTGAAGCAACTTTTTGACCGAATTCAATCGGA

14 TGAGTCTCAAAAAGGCTTTCCCATGCAGGTGAAGCAACTTTTTGACCGAATTCAATCGGA

13 TGAGTCTCAAAAAGGCTTTCCCATGCAGGTGAAGCAACTTTTTGACCGAATTCAATCGGA

12 TGAGTCTCAAAAAGGCTTTCCCATGCAGGTGAAGCAACTTTTTGACCGAATTCAATCGGA

8 TGAGTCTCAAAAAGGCTTTCCCATGCAGGTGAAGCAACTTTTTGACCGAATTCAATCGGA

7 TGAGTCTCAAAAAGGCTTTCCCATGCAGGTGAAGCAACTTTTTGACCGAATTCAATCGGA

19 TGAGTCTCAAAAAGGCTTTCCCATGCAGGTGAAGCAACTTTTTGACCGAATTCAATCGGA

18 TGAGTCTCAAAAAGGCTTTCCCATGCAGGTGAAGCAACTTTTTGACCGAATTCAATCGGA

22 TGAGTCTCAAAAAGGCTTTCCCATGCAGGTGAAGCAACTTTTTGACCGAATTCAATCGGA

21 TGAGTCTCAAAAAGGCTTTCCCATGCAGGTGAAGCAACTTTTTGACCGAATTCAATCGGA

9 TGAGTCTCAAAAAGGCTTTCCCATGCAGGTGAAGCAACTTTTTGACCGAATTCAATCGGA

6 TGAGTCTCAAAAAGGCTTTCCCATGCAGGTGAAGCAACTTTTTGACCGAATTCAATCGGA

5 TGAGTCTCAAAAAGGCTTTCCCATGCAGGTGAAGCAACTTTTTGACCGAATTCAATCGGA

4 TGAGTCTCAAAAAGGCTTTCCCATGCAGGTGAAGCAACTTTTTGACCGAATTCAATCGGA

3 TGAGTCTCAAAAAGGCTTTCCCATGCAGGTGAAGCAACTTTTTGACCGAATTCAATCGGA

20 TGAGTCTCAAAAAGGCTTTCCCATGCAGGTGAAGCAACTTTTTGACCGAATTCAATCGGA

* .********** ***** ********..**.****.*********** *****.* .

**<<<<<<<<<<<<<<<<<<<<<<<<<<<<<<<<<<<<<<<<<<<<<<<<<<<<<<<<<<<<**

1RC ATGATCTTTGCATATGGCTTAAACTTTTTCTCTTACTCAATGAGACATTCGAAATAGTAA

16RC ATGATCTTTGCATATGGCTTAAACTTTTTCTCTTACTCAATGAGACATTCGAAATAGTAA

15RC ATGATCTTTGCATATGGCTTAAACTTTTTCTCTTACTCAATGAGACATTCGAAATAGTAA

2RC ATGATCTTTGCATATGGCTTAAACTTTTTCTCTTACTCAATGAGACATTCGAAATAGTAA

10 TTGATTTTTGCGTATGGCTTGAACTTCTTCTCTTCCTCAATGAGATAGTGCATGTAGCGC

17 TTGATTTTTGCGTATGGCTTGAACTTCTTCTCTTCCTCAATGAGATAGTGCATGTAGCGC

14 TTGATTTTTGCGTATGGCTTGAACTTCTTCTCTTCCTCAATGAGATAGTGCATGTAGCGC

13 TTGATTTTTGCGTATGGCTTGAACTTCTTCTCTTCCTCAATGAGATAGTGCATGTAGCGC

12 TTGATTTTTGCGTATGGCTTGAACTTCTTCTCTTCCTCAATGAGATAGTGCATGTAGCGC

8 TTGATTTTTGCGTATGGCTTGAACTTCTTCTCTTCCTCAATGAGATAGTGCATGTAGCGC

7 TTGATTTTTGCGTATGGCTTGAACTTCTTCTCTTCCTCAATGAGATAGTGCATGTAGCGC

19 TTGATTTTTGCGTATGGCTTGAACTTCTTCTCTTCCTCAATGAGATAGTGCATGTAGCGC

18 TTGATTTTTGCGTATGGCTTGAACTTCTTCTCTTCCTCAATGAGATAGTGCATGTAGCGC

22 TTGATCTTTGCGTATGGCTTGAACTTCTTCTCTTCCTCAATGAGATAGTGCATGTAGCGC

21 TTGATTTTTGCGTATGGCTTGAACTTCTTCTCTTCCTCAATGAGATAGTGCATGTAGCGC

9 TTGATTTTTGCGTATGGCTTGAACTTCTTCTCTTCCTCAATGAGATAGTGCATGTAGCGC

6 TTGATTTTTGCGTATGGCTTGAACTTCTTCTCTTCCTCAATGAGATAGTGCATGTAGCGC

5 TTGATTTTTGCGTATGGCTTGAACTTCTTCTCTTCCTCAATGAGATAGTGCATGTAGCGC

4 TTGATTTTTGCGTATGGCTTGAACTTCTTCTCTTCCTCAATGAGATAGTGCATGTAGCGC

3 TTGATTTTTGCGTATGGCTTGAACTTCTTCTCTTCCTCAATGAGATAGTGCATGTAGCGC

20 TTGATTTTTGCGTATGGCTTGAACTTCTTCTCTTCCTCAATGAGATAGTGCATGTAGCGC

****.*****.********.*****.******* **********.* * * .***..

**<<<<<<<<<<<<<<<<<<<<<<<<<<<<<<<<<<<<<<<<<<<<<<<<<<<<<<<<<<<<**

1RC GGGTACGACTAT------------------------------------------------

16RC GGGTACGACTAT------------------------------------------------

15RC GGGTACGACTAT------------------------------------------------

2RC GGGTACGACTAT------------------------------------------------

10 AGGTAAGGTGACTTTGGCTTCACTGGAGGATCGATTTCCGGTATGCCTGACTCGTTTATT

17 AGGTAAGGTGACTTTGGCTTCACTGGAGGATCGATTTCCGGTATGCCTGACTCGTTTATT

14 AGGTAAGGTGACTTTGGCTTCACTGGAGGATCGATTTCCGGTATGCCTGACTCGTTTATT

13 AGGTAAGGTGACTTTGGCTTCACTGGAGGATCGATTTCCGGTATGCCTGACTCGTTTATT

12 AGGTAAGGTGACTTTGGCTTCACTGGAGGATCGATTTCCGGTATGCCTGACTCGTTTATT

8 AGGTAAGGTGACTTTGGCTTCACTGGAGGATCGATTTCCGGTATGCCTGACTCGTTTATT

7 AGGTAAGGTGACTTTGGCTTCACTGGAGGATCGATTTCCGGTATGCCTGACTCGTTTATT

19 AGGTAAGGTGACTTTGGCTTCACTGGAGGATCGATTTCCGGTATGCCTGACTCGTTTATT

18 AGGTAAGGTGACTTTGGCTTCACTGGAGGATCGATTTCCGGTATGCCTGACTCGTTTATT

22 AGGTAAGGTGACTTTGGCTTCACTGGAGGATCGATTTCCGGTATGCCTGACTCGTTTATT

21 AGGTAAGGTGACTTTGGCTTCACTGGAGGATCGATTTCCGGTATGCCTGACTCGTTTATT

9 AGGTAAGGTGACTTTGGCTTCACTGGAGGATCGATTTCCGGTATGCCTGACTCGTTTATT

6 AGGTAAGGTGACTTTGGCTTCACTGGAGGATCGATTTCCGGTATGCCTGACTCGTTTATT

5 AGGTAAGGTGACTTTGGCTTCACTGGAGGATCGATTTCCGGTATGCCTGACTCGTTTATT

4 AGGTAAGGTGACTTTGGCTTCACTGGAGGATCGATTTCCGGTATGCCTGACTCGTTTATT

3 AGGTAAGGTGACTTTGGCTTCACTGGAGGATCGATTTCCGGTATGCCTGACTCGTTTATT

20 AGGTAAGGTGACTTTGGCTTCACTGGAGGATCGATTTCCGGTATGCCTGACTCGTTTATT

.**** *.. *.

**<<<<<<<<<<<<<<<<<<<<<<<<<<<<<<<<<<<<<<<<<<<<<<<<<<<<<<<<<<<<**

1RC ------------------------------------------------------------

16RC ------------------------------------------------------------

15RC ------------------------------------------------------------

2RC ------------------------------------------------------------

10 TAGCAGAAATAGTGTTCTAAACAAAGTTCACCCACGGATACGTTCTCTAAATTGGATAGG

17 TAGCAGAAATAGTGTTCTAAACAAAGTTCACCCACGGATACGTTCTCTAAATTGGATAGG

14 TAGCAGAAATAGTGTTCTAAACAAAGTTCACCCACGGATACGTTCTCTAAATTGGATAGG

13 TAGCAGAAATAGTGTTCTAAACAAAGTTCACCCACGGATACGTTCTCTAAATTGGATAGG

12 TAGCAGAAATAGTGTTCTAAACAAAGTTCACCCACGGATACGTTCTCTAAATTGGATAGG

8 TAGCAGAAATAGTGTTCTAAACAAAGTTCACCCACGGATACGTTCTCTAAATTGGATAGG

7 TAGCAGAAATAGTGTTCTAAACAAAGTTCACCCACGGATACGTTCTCTAAATTGGATAGG

19 TAGCAGAAATAGTGTTCTAAACAAAGTTCACCCACGGATACGTTCTCTAAATTGGATAGG

18 TAGCAGAAATAGTGTTCTAAACAAAGTTCACCCACGGATACGTTCTCTAAATTGGATAGG

22 TAGCAGAAATAGTGTTCTAAACAAAGTTCACCCACGGATACGTTCTCTAAATTGGATAGG

21 TAGCAGAAATAGTGTTCTAAACAAAGTTCACCCACGGATACGTTCTCTAAATTGGATAGG

9 TAGCAGAAATAGTGTTCTAAACAAAGTTCACCCACGGATACGTTCTCTAAATTGGATAGG

6 TAGCAGAAATAGTGTTCTAAACAAAGTTCACCCACGGATACGTTCTCTAAATTGGATAGG

5 TAGCAGAAATAGTGTTCTAAACAAAGTTCACCCACGGATACGTTCTCTAAATTGGATAGG

4 TAGCAGAAATAGTGTTCTAAACAAAGTTCACCCACGGATACGTTCTCTAAATTGGATAGG

3 TAGCAGAAATAGTGTTCTAAACAAAGTTCACCCACGGATACGTTCTCTAAATTGGATAGG

20 TAGCAGAAATAGTGTTCTAAACAAAGTTCACCCACGGATACGTTCTCTAAATTGGATAGG

**<<<<<<<<<<<<<<<<<<<<<<<<<<<<<<<<<<<<<<<<<<<<<<<<<<<<<<<<<<<<**

1RC ------------------------------------------------------------

16RC ------------------------------------------------------------

15RC ------------------------------------------------------------

2RC ------------------------------------------------------------

10 GGTATGGCCGAAATCACGCGTAAGCAGCGAAGGCAAAGCGCCAGCGACGCCTTTGACTGG

17 GGTATGGCCGAAATCACGCGTAAGCAGCGAAGGCAAAGCGCCAGCGACGCCTTTGACTGG

14 GGTATGGCCGAAATCACGCGTAAGCAGCGAAGGCAAAGCGCCAGCGACGCCTTTGACTGG

13 GGTATGGCCGAAATCACGCGTAAGCAGCGAAGGCAAAGCGCCAGCGACGCCTTTGACTGG

12 GGTATGGCCGAAATCACGCGTAAGCAGCGAAGGCAAAGCGCCAGCGACGCCTTTGACTGG

8 GGTATGGCCGAAATCACGCGTAAGCAGCGAAGGCAAAGCGCCAGCGACGCCTTTGACTGG

7 GGTATGGCCGAAATCACGCGTAAGCAGCGAAGGCAAAGCGCCAGCGACGCCTTTGACTGG

19 GGTATGGCCGAAATCACGCGTAAGCAGCGAAGGCAAAGCGCCAGCGACGCCTTTGACTGG

18 GGTATGGCCGAAATCACGCGTAAGCAGCGAAGGCAAAGCGCCAGCGACGCCTTTGACTGG

22 GGTATGGCCGAAATCACGCGTAAGCAGCGAAGGCAAAGCGCCAGCGACGCCTTTGACTGG

21 GGTATGGCCGAAATCACGCGTAAGCAGCGAAGGCAAAGCGCCAGCGACGCCTTTGACTGG

9 GGTATGGCCGAAATCACGCGTAAGCAGCGAAGGCAAAGCGCCAGCGACGCCTTTGACTGG

6 GGTATGGCCGAAATCACGCGTAAGCAGCGAAGGCAAAGCGCCAGCGACGCCTTTGACTGG

5 GGTATGGCCGAAATCACGCGTAAGCAGCGAAGGCAAAGCGCCAGCGACGCCTTTGACTGG

4 GGTATGGCCGAAATCACGCGTAAGCAGCGAAGGCAAAGCGCCAGCGACGCCTTTGACTGG

3 GGTATGGCCGAAATCACGCGTAAGCAGCGAAGGCAAAGCGCCAGCGACGCCTTTGACTGG

20 GGTATGGCCGAAATCACGCGTAAGCAGCGAAGGCAAAGCGCCAGCGACGCCTTTGACTGG

**<<<<<<<<<<<<<<<<<<<<<<<<<<<<<<<<<<<<<<<<<<<<<<<<<<<<<<<<<<<<**

1RC -----------------------------------GAATATCTTATGCACGTCGTTCGT-

16RC -----------------------------------GAATATCTTATGCACGTCGTTCGT-

15RC -----------------------------------GAATATCTTATGCACGTCGTTCGT-

2RC -----------------------------------GAATATCTTATGCACGTCGTTCGT-

10 TGAAAGACCTGCTTCGATCAGTTATATTCAGTGCAAAATACTTCAAGCTCCTCACCTTTG

17 TGAAAGACCTGCTTCGATCAGTTATATTCAGTGCAAAATACTTCAAGCTCCTCACCTTTG

14 TGAAAGACCTGCTTCGATCAGTTATATTCAGTGCAAAATACTTCAAGCTCCTCACCTTTG

13 TGAAAGACCTGCTTCGATCAGTTATATTCAGTGCAAAATACTTCAAGCTCCTCACCTTTG

12 TGAAAGACCTGCTTCGATCAGTTATATTCAGTGCAAAATACTTCAAGCTCCTCACCTTTG

8 TGAAAGACCTGCTTCGATCAGTTATATTCAGTGCAAAATACTTCAAGCTCCTCACCTTTG

7 TGAAAGACCTGCTTCGATCAGTTATATTCAGTGCAAAATACTTCAAGCTCCTCACCTTTG

19 TGAAAGACCTGCTTCGATCAGTTATATTCAGTGCAAAATACTTCAAGCTCCTCACCTTTG

18 TGAAAGACCTGCTTCGATCAGTTATATTCAGTGCAAAATACTTCAAGCTCCTCACCTTTG

22 TGAAAGACCTGCTTCGATCAGTTATATTCAGTGCAAAATACTTCAAGCTCCTCACCTTTG

21 TGAAAGACCTGCTTCGATCAGTTATATTCAGTGCAAAATACTTCAAGCTCCTCACCTTTG

9 TGAAAGACCTGCTTCGATCAGTTATATTCAGTGCAAAATACTTCAAGCTCCTCACCTTTG

6 TGAAAGACCTGCTTCGATCAGTTATATTCAGTGCAAAATACTTCAAGCTCCTCACCTTTG

5 TGAAAGACCTGCTTCGATCAGTTATATTCAGTGCAAAATACTTCAAGCTCCTCACCTTTG

4 TGAAAGACCTGCTTCGATCAGTTATATTCAGTGCAAAATACTTCAAGCTCCTCACCTTTG

3 TGAAAGACCTGCTTCGATCAGTTATATTCAGTGCAAAATACTTCAAGCTCCTCACCTTTG

20 TGAAAGACCTGCTTCGATCAGTTATATTCAGTGCAAAATACTTCAAGCTCCTCACCTTTG

.****..*.* ** * **.... *

**<<<<<<<<<<<<<<<<<<<<<<<<<<<<<<<<<<<<<<<<<<<<<<<<<<<<<<<<<<<<**

1RC ----------------------------------------------------TTGATGTT

16RC ----------------------------------------------------TTGATGTT

15RC ----------------------------------------------------TTGATGTT

2RC ----------------------------------------------------TTGATGTT

10 TTTGGGACGGAAGCACGTGTAAGGTTGAGTGATGCAAAGCCTTTAGCAAACATCGATGGT

17 TTTGGGACGGAAGCACGTGTAAGGTTGAGTGATGCAAAGCCTTTAGCAAACATCGATGGT

14 TTTGGGACGGAAGCACGTGTAAGGTTGAGTGATGCAAAGCCTTTAGCAAACATCGATGGT

13 TTTGGGACGGAAGCACGTGTAAGGTTGAGTGATGCAAAGCCTTTAGCAAACATCGATGGT

12 TTTGGGACGGAAGCACGTGTAAGGTTGAGTGATGCAAAGCCTTTAGCAAACATCGATGGT

8 TTTGGGACGGAAGCACGTGTAAGGTTGAGTGATGCAAAGCCTTTAGCAAACATCGATGGT

7 TTTGGGACGGAAGCACGTGTAAGGTTGAGTGATGCAAAGCCTTTAGCAAACATCGATGGT

19 TTTGGGACGGAAGCACGTGTAAGGTTGAGTGATGCAAAGCCTTTAGCAAACATCGATGGT

18 TTTGGGACGGAAGCACGTGTAAGGTTGAGTGATGCAAAGCCTTTAGCAAACATCGATGGT

22 TTTGGGACGGAAGCACGTGTAAGGTTGAGTGATGCAAAGCCTTTAGCAAACATCGATGGT

21 TTTGGGACGGAAGCACGTGTAAGGTTGAGTGATGCAAAGCCTTTAGCAAACATCGATGGT

9 TTTGGGACGGAAGCACGTGTAAGGTTGAGTGATGCAAAGCCTTTAGCAAACATCGATGGT

6 TTTGGGACGGAAGCACGTGTAAGGTTGAGTGATGCAAAGCCTTTAGCAAACATCGATGGT

5 TTTGGGACGGAAGCACGTGTAAGGTTGAGTGATGCAAAGCCTTTAGCAAACATCGATGGT

4 TTTGGGACGGAAGCACGTGTAAGGTTGAGTGATGCAAAGCCTTTAGCAAACATCGATGGT

3 TTTGGGACGGAAGCACGTGTAAGGTTGAGTGATGCAAAGCCTTTAGCAAACATCGATGGT

20 TTTGGGACGGAAGCACGTGTAAGGTTGAGTGATGCAAAGCCTTTAGCAAACATCGATGGT

*.**** *

**<<<<<<<<<<<<<<<<<<<<<<<<<<<<<<<<<<<<<<<<<<<<<<<<<<HMG**

1RC GATGAAATGT----------------------------ATTCGGCTAGAGA---------

16RC GATGAAATGT----------------------------ATTCGGCTAGAGA---------

15RC GATGAAATGT----------------------------ATTCGGCTAGAGA---------

2RC GATGAAATGT----------------------------ATTCGGCTAGAGA---------

10 GATGGAAGATGGTCAAGGGATAAAATGAGGAACCACAAATTTAAATAGAGAATAAAAAAA

17 GATGGAAGATGGTCAAGGGATAAAATGAGGAACCACAAATTTAAATAGAGAATAAAAAAA

14 GATGGAAGATGGTCAAGGGATAAAATGAGGAACCACAAATTTAAATAGAGAATAAAAAAA

13 GATGGAAGATGGTCAAGGGATAAAATGAGGAACCACAAATTTAAATAGAGAATAAAAAAA

12 GATGGAAGATGGTCAAGGGATAAAATGAGGAACCACAAATTTAAATAGAGAATAAAAAAA

8 GATGGAAGATGGTCAAGGGATAAAATGAGGAACCACAAATTTAAATAGAGAATAAAAAAA

7 GATGGAAGATGGTCAAGGGATAAAATGAGGAACCACAAATTTAAATAGAGAATAAAAAAA

19 GATGGAAGATGGTCAAGGGATAAAATGAGGAACCACAAATTTAAATAGAGAATAAAAAAA

18 GATGGAAGATGGTCAAGGGATAAAATGAGGAACCACAAATTTAAATAGAGAATAAAAAAA

22 GATGGAAGATGGTCAAGGGATAAAATGAGGAACCACAAATTTAAATAGAGAATAAAAAAA

21 GATGGAAGATGGTCAAGGGATAAAATGAGGAACCACAAATTTAAATAGAGAATAAAAAAA

9 GATGGAAGATGGTCAAGGGATAAAATGAGGAACCACAAATTTAAATAGAGAATAAAAAAA

6 GATGGAAGATGGTCAAGGGATAAAATGAGGAACCACAAATTTAAATAGAGAATAAAAAAA

5 GATGGAAGATGGTCAAGGGATAAAATGAGGAACCACAAATTTAAATAGAGAATAAAAAAA

4 GATGGAAGATGGTCAAGGGATAAAATGAGGAACCACAAATTTAAATAGAGAATAAAAAAA

3 GATGGAAGATGGTCAAGGGATAAAATGAGGAACCACAAATTTAAATAGAGAATAAAAAAA

20 GATGGAAGATGGTCAAGGGATAAAATGAGGAACCACAAATTTAAATAGAGAATAAAAAAA

****.** .* ***... ******

1RC -CATCTGATT--------------------------------------------------

16RC -CATCTGATT--------------------------------------------------

15RC -CATCTGATT--------------------------------------------------

2RC -CATCTGATT--------------------------------------------------

10 TCATCTGGTCTTTAAGTTGTGCCTTACATCACCTTTCACCGTTCCAATCCGGTACTTGGC

17 TCATCTGGTCTTTAAGTTGTGCCTTACATCACCTTTCACCGTTCCAATCCGGTACTTGGC

14 TCATCTGGTCTTTAAGTTGTGCCTTACATCACCTTTCACCGTTCCAATCCGGTACTTGGC

13 TCATCTGGTCTTTAAGTTGTGCCTTACATCACCTTTCACCGTTCCAATCCGGTACTTGGC

12 TCATCTGGTCTTTAAGTTGTGCCTTACATCACCTTTCACCGTTCCAATCCGGTACTTGGC

8 TCATCTGGTCTTTAAGTTGTGCCTTACATCACCTTTCACCGTTCCAATCCGGTACTTGGC

7 TCATCTGGTCTTTAAGTTGTGCCTTACATCACCTTTCACCGTTCCAATCCGGTACTTGGC

19 TCATCTGGTCTTTAAGTTGTGCCTTACATCACCTTTCACCGTTCCAATCCGGTACTTGGC

18 TCATCTGGTCTTTAAGTTGTGCCTTACATCACCTTTCACCGTTCCAATCCGGTACTTGGC

22 TCATCTGGTCTTTAAGTTGTGCCTTACATCACCTTTCACCGTTCCAATCCGGTACTTGGC

21 TCATCTGGTCTTTAAGTTGTGCCTTACATCACCTTTCACCGTTCCAATCCGGTACTTGGC

9 TCATCTGGTCTTTAAGTTGTGCCTTACATCACCTTTCACCGTTCCAATCCGGTACTTGGC

6 TCATCTGGTCTTTAAGTTGTGCCTTACATCACCTTTCACCGTTCCAATCCGGTACTTGGC

5 TCATCTGGTCTTTAAGTTGTGCCTTACATCACCTTTCACCGTTCCAATCCGGTACTTGGC

4 TCATCTGGTCTTTAAGTTGTGCCTTACATCACCTTTCACCGTTCCAATCCGGTACTTGGC

3 TCATCTGGTCTTTAAGTTGTGCCTTACATCACCTTTCACCGTTCCAATCCGGTACTTGGC

20 TCATCTGGTCTTTAAGTTGTGCCTTACATCACCTTTCACCGTTCCAATCCGGTACTTGGC

******.*.

1RC ---------------------TACGATTACAGCAAAAAAGAAA-----------------

16RC ---------------------TACGATTACAGCAAAAAAGAAA-----------------

15RC ---------------------TACGATTACAGCAAAAAAGAAA-----------------

2RC ---------------------TACGATTACAGCAAAAAAGAAA-----------------

10 CGAGAACCCCACAGTGAATAATACAATTAAGAAAATTAAGAAAACAATGAAAACAATGAG

17 CGAGAACCCCACAGTGAAAAATACAATTAAGAAAATTAAGAAAACAATGAAAACAATGAG

14 CGAGAACCCCACAGTGAAAAATACAATTAAGAAAATTAAGAAAACAATGAAAACAATGAG

13 CGAGAACCCCACAGTGAAAAATACAATTAAGAAAATTAAGAAAACAATGAAAACAATGAG

12 CGAGAACCCCACAGTGAAAAATACAATTAAGAAAATTAAGAAAACAATGAAAACAATGAG

8 CGAGAACCCCACAGTGAAAAATACAATTAAGAAAATTAAGAAAACAATGAAAACAATGAG

7 CGAGAACCCCACAGTGAAAAATACAATTAAGAAAATTAAGAAAACAATGAAAACAATGAG

19 CGAGAACCCCACAGTGAAAAATACAATTAAGAAAATTAAGAAAACAATGAAAACAATGAG

18 CGAGAACCCCACAGTGAAAAATACAATTAAGAAAATTAAGAAAACAATGAAAACAATGAG

22 CGAGAACCCCACAGTGAAAAATACAATTAAGAAAATTAAGAAAACAATGAAAACAATGAG

21 CGAGAACCCCACAGTGAATAATACAATTAAGAAAATTAAGAAAACAATGAAAACAATGAG

9 CGAGAACCCCACAGTGAATAATACAATTAAGAAAATTAAGAAAACAATGAAAACAATGAG

6 CGAGAACCCCACAGTGAATAATACAATTAAGAAAATTAAGAAAACAATGAAAACAATGAG

5 CGAGAACCCCACAGTGAATAATACAATTAAGAAAATTAAGAAAACAATGAAAACAATGAG

4 CGAGAACCCCACAGTGAATAATACAATTAAGAAAATTAAGAAAACAATGAAAACAATGAG

3 CGAGAACCCCACAGTGAATAATACAATTAAGAAAATTAAGAAAACAATGAAAACAATGAG

20 CGAGAACCCCACAGTGAATAATACAATTAAGAAAATTAAGAAAACAATGAAAACAATGAG

***.**** .. ** ******

1RC ----------------------------------------------------CACTGGTG

16RC ----------------------------------------------------CACTGGTG

15RC ----------------------------------------------------CACTGGTG

2RC ----------------------------------------------------CACTGGTG

10 AACAATGAAAAGTATAAGAACAAAGCGGACGAAGCGATAAGAACGATGAATTAATAAATG

17 AACAATGAAAAGTATAAGAACAAAGCGGACGAAGCGATAAGAACGATGAATTAATAAATG

14 AACAATGAAAAGTATAAGAACAAAGCGGACGAAGCGATAAGAACGATGAATTAATAAATG

13 AACAATGAAAAGTATAAGAACAAAGCGGACGAAGCGATAAGAACGATGAATTAATAAATG

12 AACAATGAAAAGTATAAGAACAAAGCGGACGAAGCGATAAGAACGATGAATTAATAAATG

8 AACAATGAAAAGTATAAGAACAAAGCGGACGAAGCGATAAGAACGATGAATTAATAAATG

7 AACAATGAAAAGTATAAGAACAAAGCGGACGAAGCGATAAGAACGATGAATTAATAAATG

19 AACAATGAAAAGTATAAGAACAAAGCGGACGAAGCGATAAGAACGATGAATTAATAAATG

18 AACAATGAAAAGTATAAGAACAAAGCGGACGAAGCGATAAGAACGATGAATTAATAAATG

22 AACAATGAAAAGTATAAGAACAAAGCGGACGAAGCGATAAGAACGATGAATTAATAAATG

21 AACAATGAAAAGTATAAGAACAAAGCGGACGAAGCGATAAGAACGATGAATTAATAAATG

9 AACAATGAAAAGTATAAGAACAAAGCGGACGAAGCGATAAGAACGATGAATTAATAAATG

6 AACAATGAAAAGTATAAGAACAAAGCGGACGAAGCGATAAGAACGATGAATTAATAAATG

5 AACAATGAAAAGTATAAGAACAAAGCGGACGAAGCGATAAGAACGATGAATTAATAAATG

4 AACAATGAAAAGTATAAGAACAAAGCGGACGAAGCGATAAGAACGATGAATTAATAAATG

3 AACAATGAAAAGTATAAGAACAAAGCGGACGAAGCGATAAGAACGATGAATTAATAAATG

20 AACAATGAAAAGTATAAGAACAAAGCGGACGAAGCGATAAGAACGATGAATTAATAAATG

*. ..**

1RC AGGCATAATTTACAATAGAGATTACAAGCTAGTGAATTGGCGGTAACTTTAGTGGCAAGT

16RC AGGCATAATTTACAATAGAGATTACAAGCTAGTGAATTGGCGGTAACTTTAGTGGCAAGT

15RC AGGCATAATTTACAATAGAGATTACAAGCTAGTGAATTGGCGGTAACTTTAGTGGCAAGT

2RC AGGCATAATTTACAATAGAGATTACAAGCTAGTGAATTGGCGGTAACTTTAGTGGCAAGT

10 ATGCACAATTTACA--AAGAATTACAAGCTGGTGATTTGGCGGTAACTTTAGTGGCAAGT

17 ATGCACAATTTACA--AAGAATTACAAGCTGGTGATTTGGCGGTAACTTTAGTGGCAAGT

14 ATGCACAATTTACA--AAGAATTACAAGCTGGTGATTTGGCGGTAACTTTAGTGGCAAGT

13 ATGCACAATTTACA--AAGAATTACAAGCTGGTGATTTGGCGGTAACTTTAGTGGCAAGT

12 ATGCACAATTTACA--AAGAATTACAAGCTGGTGATTTGGCGGTAACTTTAGTGGCAAGT

8 ATGCACAATTTACA--AAGAATTACAAGCTGGTGATTTGGCGGTAACTTTAGTGGCAAGT

7 ATGCACAATTTACA--AAGAATTACAAGCTGGTGATTTGGCGGTAACTTTAGTGGCAAGT

19 ATGCACAATTTACA--AAGAATTACAAGCTGGTGATTTGGCGGTAACTTTAGTGGCAAGT

18 ATGCACAATTTACA--AAGAATTACAAGCTGGTGATTTGGCGGTAACTTTAGTGGCAAGT

22 ATGCACAATTTACA--AAGAATTACAAGCTGGTGATTTGGCGGTAACTTTAGTGGCAAGT

21 ATGCACAATTTACA--AAGAATTACAAGCTGGTGATTTGGCGGTAACTTTAGTGGCAAGT

9 ATGCACAATTTACA--AAGAATTACAAGCTGGTGATTTGGCGGTAACTTTAGTGGCAAGT

6 ATGCACAATTTACA--AAGAATTACAAGCTGGTGATTTGGCGGTAACTTTAGTGGCAAGT

5 ATGCACAATTTACA--AAGAATTACAAGCTGGTGATTTGGCGGTAACTTTAGTGGCAAGT

4 ATGCACAATTTACA--AAGAATTACAAGCTGGTGATTTGGCGGTAACTTTAGTGGCAAGT

3 ATGCACAATTTACA--AAGAATTACAAGCTGGTGATTTGGCGGTAACTTTAGTGGCAAGT

20 ATGCACAATTTACA--AAGAATTACAAGCTGGTGATTTGGCGGTAACTTTAGTGGCAAGT

* ***.******** *...**********.**** ************************

1RC AGTGAGAGACTAAGAACAGACTCACAAGTACTGAGAGGGTGTGACCCGTGTTGCTGAACC

16RC AGTGAGAGACTAAGAACAGACTCACAAGTACTGAGAGGGTGTGACCCGTGTTGCTGAACC

15RC AGTGAGAGACTAAGAACAGACTCACAAGTACTGAGAGGGTGTGACCCGTGTTGCTGAACC

2RC AGTGAGAGACTAAGAACAGACTCACAAGTACTGAGAGGGTGTGACCCGTGTTGCTGAACC

10 AGTGAGAGACTAAGAATAGACTCACAAGTACTGAGAGGGTGTGACCCGTGTTGCTGAACC

17 AGTGAGAGACTAAGAATAGACTCACAAGTACTGAGAGGGTGTGACCCGTGTTGCTGAACC

14 AGTGAGAGACTAAGAATAGACTCACAAGTACTGAGAGGGTGTGACCCGTGTTGCTGAACC

13 AGTGAGAGACTAAGAATAGACTCACAAGTACTGAGAGGGTGTGACCCGTGTTGCTGAACC

12 AGTGAGAGACTAAGAATAGACTCACAAGTACTGAGAGGGTGTGACCCGTGTTGCTGAACC

8 AGTGAGAGACTAAGAATAGACTCACAAGTACTGAGAGGGTGTGACCCGTGTTGCTGAACC

7 AGTGAGAGACTAAGAATAGACTCACAAGTACTGAGAGGGTGTGACCCGTGTTGCTGAACC

19 AGTGAGAGACTAAGAATAGACTCACAAGTACTGAGAGGGTGTGACCCGTGTTGCTGAACC

18 AGTGAGAGACTAAGAATAGACTCACAAGTACTGAGAGGGTGTGACCCGTGTTGCTGAACC

22 AGTGAGAGACTAAGAATAGACTCACAAGTACTGAGAGGGTGTGACCCGTGTTGCTGAACC

21 AGTGAGAGACTAAGAATAGACTCACAAGTACTGAGAGGGTGTGACCCGTGTTGCTGAACC

9 AGTGAGAGACTAAGAATAGACTCACAAGTACTGAGAGGGTGTGACCCGTGTTGCTGAACC

6 AGTGAGAGACTAAGAATAGACTCACAAGTACTGAGAGGGTGTGACCCGTGTTGCTGAACC

5 AGTGAGAGACTAAGAATAGACTCACAAGTACTGAGAGGGTGTGACCCGTGTTGCTGAACC

4 AGTGAGAGACTAAGAATAGACTCACAAGTACTGAGAGGGTGTGACCCGTGTTGCTGAACC

3 AGTGAGAGACTAAGAATAGACTCACAAGTACTGAGAGGGTGTGACCCGTGTTGCTGAACC

20 AGTGAGAGACTAAGAATAGACTCACAAGTACTGAGAGGGTGTGACCCGTGTTGCTGAACC

****************.*******************************************

1RC CCTATCCTGATCTGCCACTGTGAAGATACTCAACAAATGATAAAGGAATAAGTAAAAACA

16RC CCTATCCTGATCTGCCACTGTGAAGATACTCAACAAATGATAAAGGAATAAGTAAAAACA

15RC CCTATCCTGATCTGCCACTGTGAAGATACTCAACAAATGATAAAGGAATAAGTAAAAACA

2RC CCTATCCTGATCTGCCACTGTGAAGATACTCAACAAATGATAAAGGAATAAGTAAAAACA

10 CCTATCCTGATCTGCCACTGTGAAGATACTCAACAGATGATAAAGGAACAAGTAAAAACA

17 CCTATCCTGATCTGCCACTGTGAAGATACTCAACAGATGATAAAGGAACAAGTAAAAACA

14 CCTATCCTGATCTGCCACTGTGAAGATACTCAACAGATGATAAAGGAACAAGTAAAAACA

13 CCTATCCTGATCTGCCACTGTGAAGATACTCAACAGATGATAAAGGAACAAGTAAAAACA

12 CCTATCCTGATCTGCCACTGTGAAGATACTCAACAGATGATAAAGGAACAAGTAAAAACA

8 CCTATCCTGATCTGCCACTGTGAAGATACTCAACAGATGATAAAGGAACAAGTAAAAACA

7 CCTATCCTGATCTGCCACTGTGAAGATACTCAACAGATGATAAAGGAACAAGTAAAAACA

19 CCTATCCTGATCTGCCACTGTGAAGATACTCAACAGATGATAAAGGAACAAGTAAAAACA

18 CCTATCCTGATCTGCCACTGTGAAGATACTCAACAGATGATAAAGGAACAAGTAAAAACA

22 CCTATCCTGATCTGCCACTGTGAAGATACTCAACAGATGATAAAGGAACAAGTAAAAACA

21 CCTATCCTGATCTGCCACTGTGAAGATACTCAACAGATGATAAAGGAACAAGTAAAAACA

9 CCTATCCTGATCTGCCACTGTGAAGATACTCAACAGATGATAAAGGAACAAGTAAAAACA

6 CCTATCCTGATCTGCCACTGTGAAGATACTCAACAGATGATAAAGGAACAAGTAAAAACA

5 CCTATCCTGATCTGCCACTGTGAAGATACTCAACAGATGATAAAGGAACAAGTAAAAACA

4 CCTATCCTGATCTGCCACTGTGAAGATACTCAACAGATGATAAAGGAACAAGTAAAAACA

3 CCTATCCTGATCTGCCACTGTGAAGATACTCAACAGATGATAAAGGAACAAGTAAAAACA

20 CCTATCCTGATCTGCCACTGTGAAGATACTCAACAGATGATAAAGGAACAAGTAAAAACA

***********************************.************.***********

**PPE1<<<<<<<<<<<<<<<<<<<<<<<<<<<<<<<<<<<<<<<<<<<<<<<<<<<<<<<**

1RC AACCAGCATCGAAAATCGCGAAGTTGTTCTCTGTGACAGGCTCATTCTCTTTTACTTTTA

16RC AACCAGCATCGAAAATCGCGAAGTTGTTCTCTGTTACAGGCTCATTCTCTTTTACTTTTA

15RC AACCAGCATCGAAAATCGCGAAGTTGTTCTCTGTGACAGGCTCATTCTCTTTTACTTTTA

2RC AACCAGCATCGAAAATCGCGAAGTTGTTCTCTGTGACAGGCTCATTCTCTTTTACTTTTA

10 AACCAGCATCGAAAATCGCGAAGTTGTTCTCTGTGACAGGCTCATTCTCTTTTACTTTGA

17 AACCAGCATCGAAAATCGCGAAGTTGTTCTCTGTGACAGGCTCATTCTCTTTTACTTTGA

14 AACCAGCATCGAAAATCGCGAAGTTGTTCTCTGTGACAGGCTCATTCTCTTTTACTTTGA

13 AACCAGCATCGAAAATCGCGAAGTTGTTCTCTGTGACAGGCTCATTCTCTTTTACTTTGA

12 AACCAGCATCGAAAATCGCGAAGTTGTTCTCTGTGACAGGCTCATTCTCTTTTACTTTGA

8 AACCAGCATCGAAAATCGCGAAGTTGTTCTCTGTGACAGGCTCATTCTCTTTTACTTTGA

7 AACCAGCATCGAAAATCGCGAAGTTGTTCTCTGTGACAGGCTCATTCTCTTTTACTTTGA

19 AACCAGCATCGAAAATCGCGAAGTTGTTCTCTGTGACAGGCTCATTCTCTTTTACTTTGA

18 AACCAGCATCGAAAATCGCGAAGTTGTTCTCTGTGACAGGCTCATTCTCTTTTACTTTGA

22 AACCAGCATCGAAAATCGCGAAGTTGTTCTCTGTGACAGGCTCATTCTCTTTTACTTTGA

21 AACCAGCATCGAAAATCGCGAAGTTGTTCTCTGTGACAGGCTCATTCTCTTTTACTTTGA

9 AACCAGCATCGAAAATCGCGAAGTTGTTCTCTGTGACAGGCTCATTCTCTTTTACTTTGA

6 AACCAGCATCGAAAATCGCGAAGTTGTTCTCTGTGACAGGCTCATTCTCTTTTACTTTGA

5 AACCAGCATCGAAAATCGCGAAGTTGTTCTCTGTGACAGGCTCATTCTCTTTTACTTTGA

4 AACCAGCATCGAAAATCGCGAAGTTGTTCTCTGTGACAGGCTCATTCTCTTTTACTTTGA

3 AACCAGCATCGAAAATCGCGAAGTTGTTCTCTGTGACAGGCTCATTCTCTTTTACTTTGA

20 AACCAGCATCGAAAATCGCGAAGTTGTTCTCTGTGACAGGCTCATTCTCTTTTACTTTGA

********************************** *********************** *

**<<<<<<<<<<<<<<<<<<<<<<<<<<<<<<<<<<<<<<<<<<<<<<<<<<<<<<<<<<<<**

1RC GAACACTTGCAACGTTCCCTTTGTCGAAGTAAGCAAATTGACAAAAATTGTGGAATGTTG

16RC GAACACTTGCAACGTTCCCTTTGTCGAAGTAAGCAAATTGACAAAAATTGTGGAATGTTG

15RC GAACACTTGCAACGTTCCCTTTGTCGAAGTAAGCAAATTGACAAAAATTGTGGAATGTTG

2RC GAACACTTGCAACGTTCCCTTTGTCGAAGTAAGCAAATTGACAAAAATTGTGGAATGTTG

10 GAACACTTGCAACGTTCCCTTTGTCGAAGTAAGCAAATTGACAGAAATTGTGGAATGTTG

17 GAACACTTGCAACGTTCCCTTTGTCGAAGTAAGCAAATTGACAGAAATTGTGGAATGTTG

14 GAACACTTGCAACGTTCCCTTTGTCGAAGTAAGCAAATTGACAGAAATTGTGGAATGTTG

13 GAACACTTGCAACGTTCCCTTTGTCGAAGTAAGCAAATTGACAGAAATTGTGGAATGTTG

12 GAACACTTGCAACGTTCCCTTTGTCGAAGTAAGCAAATTGACAGAAATTGTGGAATGTTG

8 GAACACTTGCAACGTTCCCTTTGTCGAAGTAAGCAAATTGACAGAAATTGTGGAATGTTG

7 GAACACTTGCAACGTTCCCTTTGTCGAAGTAAGCAAATTGACAGAAATTGTGGAATGTTG

19 GAACACTTGCAACGTTCCCTTTGTCGAAGTAAGCAAATTGACAGAAATTGTGGAATGTTG

18 GAACACTTGCAACGTTCCCTTTGTCGAAGTAAGCAAATTGACAGAAATTGTGGAATGTTG

22 GAACACTTGCAACGTTCCCTTTGTCGAAGTAAGCAAATTGACAGAAATTGTGGAATGTTG

21 GAACACTTGCAACGTTCCCTTTGTCGAAGTAAGCAAATTGACAGAAATTGTGGAATGTTG

9 GAACACTTGCAACGTTCCCTTTGTCGAAGTAAGCAAATTGACAGAAATTGTGGAATGTTG

6 GAACACTTGCAACGTTCCCTTTGTCGAAGTAAGCAAATTGACAGAAATTGTGGAATGTTG

5 GAACACTTGCAACGTTCCCTTTGTCGAAGTAAGCAAATTGACAGAAATTGTGGAATGTTG

4 GAACACTTGCAACGTTCCCTTTGTCGAAGTAAGCAAATTGACAGAAATTGTGGAATGTTG

3 GAACACTTGCAACGTTCCCTTTGTCGAAGTAAGCAAATTGACAGAAATTGTGGAATGTTG

20 GAACACTTGCAACGTTCCCTTTGTCGAAGTAAGCAAATTGACAGAAATTGTGGAATGTTG

*******************************************.****************

**<<<<<<<<<<<<<<<<<<<<<<<<<<<<<<<<<<<<<<<<<<<<<<<<<<<<<<<<<<<<**

1RC ACGAACCGCATCTATAACAGTAGTTTGGCGCAGACCACACGGTCACTAAATCATTGTCAG

16RC ACGAACCGCATCTATAACAGTAGTTTGGCGCAGACCACACGGTCACTAAATCATTGTCAG

15RC ACGAACCGCATCTATAACAGTAGTTTGGCGCAGACCACACGGTCACTAAATCATTGTCAG

2RC ACGAACCGCATCTATAACAGTAGTTTGGCGCAGACCACACGGTCACTAAATCATTGTCAG

10 ACGAACCGCATCTATAACAGTAGTTTGGCGCCGACCACACGGTCACTAAATCGTTGTCAG

17 ACGAACCGCATCTATAACAGTAGTTTGGCGCCGACCACACGGTCACTAAATCGTTGTCAG

14 ACGAACCGCATCTATAACAGTAGTTTGGCGCCGACCACACGGTCACTAAATCGTTGTCAG

13 ACGAACCGCATCTATAACAGTAGTTTGGCGCCGACCACACGGTCACTAAATCGTTGTCAG

12 ACGAACCGCATCTATAACAGTAGTTTGGCGCCGACCACACGGTCACTAAATCGTTGTCAG

8 ACGAACCGCATCTATAACAGTAGTTTGGCGCCGACCACACGGTCACTAAATCGTTGTCAG

7 ACGAACCGCATCTATAACAGTAGTTTGGCGCCGACCACACGGTCACTAAATCGTTGTCAG

19 ACGAACCGCATCTATAACAGTAGTTTGGCGCCGACCACACGGTCACTAAATCGTTGTCAG

18 ACGAACCGCATCTATAACAGTAGTTTGGCGCCGACCACACGGTCACTAAATCGTTGTCAG

22 ACGAACCGCATCTATAACAGTAGTTTGGCGCCGACCACACGGTCACTAAATCGTTGTCAG

21 ACGAACCGCATCTATAACAGTAGTTTGGCGCCGACCACACGGTCACTAAATCGTTGTCAG

9 ACGAACCGCATCTATAACAGTAGTTTGGCGCCGACCACACGGTCACTAAATCGTTGTCAG

6 ACGAACCGCATCTATAACAGTAGTTTGGCGCCGACCACACGGTCACTAAATCGTTGTCAG

5 ACGAACCGCATCTATAACAGTAGTTTGGCGCCGACCACACGGTCACTAAATCGTTGTCAG

4 ACGAACCGCATCTATAACAGTAGTTTGGCGCCGACCACACGGTCACTAAATCGTTGTCAG

3 ACGAACCGCATCTATAACAGTAGTTTGGCGCCGACCACACGGTCACTAAATCGTTGTCAG

20 ACGAACCGCATCTATAACAGTAGTTTGGCGCCGACCACACGGTCACTAAATCGTTGTCAG

******************************* ********************.*******

**<<<<<<<<<<<<<<<<<<<<<<<<<<<<<<<<<<<<<<<<<<<<<<<<<<<<<<<<<<<<**

1RC GAAACATAAATTTGCTTCTCTTGAGTGAGTCGCAGTCCAAATGAAATGTGGCAACCTACT

16RC GAAACATAAATTTGCTTCTCTTGAGTGAGTCGCAGTCCAAATGAAATGTGGCAACCTACT

15RC GAAACATAAATTTGCTTCTCTTGAGTGAGTCGCAGTCCAAATGAAATGTGGCAACCTACT

2RC GAAACATAAATTTGCTTCTCTTGAGTGAGTCGCAGTCCAAATGAAATGTGGCAACCTACT

10 GAAACATAAATTTGCTTCTCTTGAGTGAGTCGCAGTCCAAATGAAATGTGGCAACCTACT

17 GAAACATAAATTTGCTTCTCTTGAGTGAGTCGCAGTCCAAATGAAATGTGGCAACCTACT

14 GAAACATAAATTTGCTTCTCTTGAGTGAGTCGCAGTCCAAATGAAATGTGGCAACCTACT

13 GAAACATAAATTTGCTTCTCTTGAGTGAGTCGCAGTCCAAATGAAATGTGGCAACCTACT

12 GAAACATAAATTTGCTTCTCTTGAGTGAGTCGCAGTCCAAATGAAATGTGGCAACCTACT

8 GAAACATAAATTTGCTTCTCTTGAGTGAGTCGCAGTCCAAATGAAATGTGGCAACCTACT

7 GAAACATAAATTTGCTTCTCTTGAGTGAGTCGCAGTCCAAATGAAATGTGGCAACCTACT

19 GAAACATAAATTTGCTTCTCTTGAGTGAGTCGCAGTCCAAATGAAATGTGGCAACCTACT

18 GAAACATAAATTTGCTTCTCTTGAGTGAGTCGCAGTCCAAATGAAATGTGGCAACCTACT

22 GAAACATAAATTTGCTTCTCTTGAGTGAGTCGCAGTCCAAATGAAATGTGGCAACCTACT

21 GAAACATAAATTTGCTTCTCTTGAGTGAGTCGCAGTCCAAATGAAATGTGGCAACCTACT

9 GAAACATAAATTTGCTTCTCTTGAGTGAGTCGCAGTCCAAATGAAATGTGGCAACCTACT

6 GAAACATAAATTTGCTTCTCTTGAGTGAGTCGCAGTCCAAATGAAATGTGGCAACCTACT

5 GAAACATAAATTTGCTTCTCTTGAGTGAGTCGCAGTCCAAATGAAATGTGGCAACCTACT

4 GAAACATAAATTTGCTTCTCTTGAGTGAGTCGCAGTCCAAATGAAATGTGGCAACCTACT

3 GAAACATAAATTTGCTTCTCTTGAGTGAGTCGCAGTCCAAATGAAATGTGGCAACCTACT

20 GAAACATAAATTTGCTTCTCTTGAGTGAGTCGCAGTCCAAATGAAATGTGGCAACCTACT

************************************************************

**<<<<<<<<<<<<<<<<<<<<<<<<<<<<<<<<<<<<<<<<<<<<<<<<<<<<<<<<<<<<**

1RC ATCCCTCTTGGATAAGTTGATGAGCGCGCGCAATCATATCCAGTCCGTTTATGTGATTAA

16RC ATCCCTCTTGGATAAGTTGATGAGCGCGCGCAATCATATCCAGTCCGTTTATGTGATTAA

15RC ATCCCTCTTGGATAAGTTGATGAGCGCGCGCAATCATATCCAGTCCGTTTATGTGATTAA

2RC ATCCCTCTTGGATAAGTTGATGAGCGCGCGCAATCATATCCAGTCCGTTTATGTGATTAA

10 ATCCCTCTTGGATAAGTTGATGAGCGCGCGCAATCATATCCAGTCCGTTTATGTGATTAA

17 ATCCCTCTTGGATAAGTTGATGAGCGCGCGCAATCATATCCAGTCCGTTTATGTGATTAA

14 ATCCCTCTTGGATAAGTTGATGAGCGCGCGCAATCATATCCAGTCCGTTTATGTGATTAA

13 ATCCCTCTTGGATAAGTTGATGAGCGCGCGCAATCATATCCAGTCCGTTTATGTGATTAA

12 ATCCCTCTTGGATAAGTTGATGAGCGCGCGCAATCATATCCAGTCCGTTTATGTGATTAA

8 ATCCCTCTTGGATAAGTTGATGAGCGCGCGCAATCATATCCAGTCCGTTTATGTGATTAA

7 ATCCCTCTTGGATAAGTTGATGAGCGCGCGCAATCATATCCAGTCCGTTTATGTGATTAA

19 ATCCCTCTTGGATAAGTTGATGAGCGCGCGCAATCATATCCAGTCCGTTTATGTGATTAA

18 ATCCCTCTTGGATAAGTTGATGAGCGCGCGCAATCATATCCAGTCCGTTTATGTGATTAA

22 ATCCCTCTTGGATAAGTTGATGAGCGCGCGCAATCATATCCAGTCCGTTTATGTGATTAA

21 ATCCCTCTTGGATAAGTTGATGAGCGCGCGCAATCATATCCAGTCCGTTTATGTGATTAA

9 ATCCCTCTTGGATAAGTTGATGAGCGCGCGCAATCATATCCAGTCCGTTTATGTGATTAA

6 ATCCCTCTTGGATAAGTTGATGAGCGCGCGCAATCATATCCAGTCCGTTTATGTGATTAA

5 ATCCCTCTTGGATAAGTTGATGAGCGCGCGCAATCATATCCAGTCCGTTTATGTGATTAA

4 ATCCCTCTTGGATAAGTTGATGAGCGCGCGCAATCATATCCAGTCCGTTTATGTGATTAA

3 ATCCCTCTTGGATAAGTTGATGAGCGCGCGCAATCATATCCAGTCCGTTTATGTGATTAA

20 ATCCCTCTTGGATAAGTTGATGAGCGCGCGCAACCATATCCAGTCCGTTTATGTGATTAA

*********************************.**************************

**<<<<<<<<<<<<<<<<<<<<<<<<<<<<<<<<<<<<<<<<<<<<<<<<<<<<<<<<<<<<**

1RC ACTGGAGTTCAAGTTAAAACAAATATCAGCGTAGAAATGTGATATACCTCTTTCGTAACC

16RC ACTGGAGTTCAAGTTAAAACAAATATCAGCGTAGAAATGTGATATACCTCTTTCGTAACC

15RC ACTGGAGTTCAAGTTAAAACAAATATCAGCGTAGAAATGTGATATACCTCTTTCGTAACC

2RC ACTGGAGTTCAAGTTAAAACAAATATCAGCGTAGAAATGTGATATACCTCTTTCGTAACC

10 ACTGGAGTTCAAGTTAAAACAAATATCAGCGTAGAAATGTGATATACCTCTTTCGTAACC

17 ACTGGAGTTCAAGTTAAAACAAATATCAGCGTAGAAATGTGATATACCTCTTTCGTAACC

14 ACTGGAGTTCAAGTTAAAACAAATATCAGCGTAGAAATGTGATATACCTCTTTCGTAACC

13 ACTGGAGTTCAAGTTAAAACAAATATCAGCGTAGAAATGTGATATACCTCTTTCGTAACC

12 ACTGGAGTTCAAGTTAAAACAAATATCAGCGTAGAAATGTGATATACCTCTTTCGTAACC

8 ACTGGAGTTCAAGTTAAAACAAATATCAGCGTAGAAATGTGATATACCTCTTTCGTAACC

7 ACTGGAGTTCAAGTTAAAACAAATATCAGCGTAGAAATGTGATATACCTCTTTCGTAACC

19 ACTGGAGTTCAAGTTAAAACAAATATCAGCGTAGAAATGTGATATACCTCTTTCGTAACC

18 ACTGGAGTTCAAGTTAAAACAAATATCAGCGTAGAAATGTGATATACCTCTTTCGTAACC

22 ACTGGAGTTCAAGTTAAAACAAATATCAGCGTAGAAATGTGATATACCTCTTTCGTAACC

21 ACTGGAGTTCAAGTTAAAACAAATATCAGCGTAGAAATGTGATATACCTCTTTCGTAACC

9 ACTGGAGTTCAAGTTAAAACAAATATCAGCGTAGAAATGTGATATACCTCTTTCGTAACC

6 ACTGGAGTTCAAGTTAAAACAAATATCAGCGTAGAAATGTGATATACCTCTTTCGTAACC

5 ACTGGAGTTCAAGTTAAAACAAATATCAGCGTAGAAATGTGATATACCTCTTTCGTAACC

4 ACTGGAGTTCAAGTTAAAACAAATATCAGCGTAGAAATGTGATATACCTCTTTCGTAACC

3 ACTGGAGTTCAAGTTAAAACAAATATCAGCGTAGAAATGTGATATACCTCTTTCGTAACC

20 ACTGGAGTTCAAGTTAAAACAAATATCAGCGTAGAAATGTGATATACCTCTTTCGTAACC

************************************************************

**<<<<<<<<<<<<<<<<<<<<<<<<<<<<<<<<<<<<<<<<<<<<<<<<<<<<<<<<<<<<**

1RC TTATTACCAAACAGCCAACCAGCCCCTCTGGGACTAATACTCCAATGCTCAACATCATCA

16RC TTATTACCAAACAGCCAACCAGCCCCTCTGGGACTAATACTCCAATGCTCAACATCATCA

15RC TTATTACCAAACAGCCAACCAGCCCCTCTGGGACTAATACTCCAATGCTCAACATCATCA

2RC TTATTACCAAACAGCCAACCAGCCCCTCTGGGACTAATACTCCAATGCTCAACATCATCA

10 TTATTACCAAACAGCCAACCAGCCCCTCTGGGACTAATACTCCAATGCTCAACATCATCA

17 TTATTACCAAACAGCCAACCAGCCCCTCTGGGACTAATACTCCAATGCTCAACATCATCA

14 TTATTACCAAACAGCCAACCAGCCCCTCTGGGACTAATACTCCAATGCTCAACATCATCA

13 TTATTACCAAACAGCCAACCAGCCCCTCTGGGACTAATACTCCAATGCTCAACATCATCA

12 TTATTACCAAACAGCCAACCAGCCCCTCTGGGACTAATACTCCAATGCTCAACATCATCA

8 TTATTACCAAACAGCCAACCAGCCCCTCTGGGACTAATACTCCAATGCTCAACATCATCA

7 TTATTACCAAACAGCCAACCAGCCCCTCTGGGACTAATACTCCAATGCTCAACATCATCA

19 TTATTACCAAACAGCCAACCAGCCCCTCTGGGACTAATACTCCAATGCTCAACATCATCA

18 TTATTACCAAACAGCCAACCAGCCCCTCTGGGACTAATACTCCAATGCTCAACATCATCA

22 TTATTACCAAACAGCCAACCAGCCCCTCTGGGACTAATACTCCAATGCTCAACATCATCA

21 TTATTACCAAACAGCCAACCAGCCCCTCTGGGACTAATACTCCAATGCTCAACATCATCA

9 TTATTACCAAACAGCCAACCAGCCCCTCTGGGACTAATACTCCAATGCTCAACATCATCA

6 TTATTACCAAACAGCCAACCAGCCCCTCTGGGACTAATACTCCAATGCTCAACATCATCA

5 TTATTACCAAACAGCCAACCAGCCCCTCTGGGACTAATACTCCAATGCTCAACATCATCA

4 TTATTACCAAACAGCCAACCAGCCCCTCTGGGACTAATACTCCAATGCTCAACATCATCA

3 TTATTACCAAACAGCCAACCAGCCCCTCTGGGACTAATACTCCAATGCTCAACATCATCA

20 TTATTACCAAACAGCCAACCAGCCCCTCTGGGACTAATACTCCAATGCTCAACATCATCA

************************************************************

**<<<<<<<<<<<<<<<<<<<<<<<<<<<<<<<<<<<<<<<<<<<<<<<<<<<<<<<<<<<<**

1RC GGATCTGACCACATGAGATCTAAATGGTATTAACAGAGAATATGTGTCAAATCGAGCTAT

16RC GGATCTGACCACATGAGATCTAAATGGTATTAACAGAGAATATGTGTCAAATCGAGCTAT

15RC GGATCTGACCACATGAGATCTAAATGGTATTAACAGAGAATATGTGTCAAATCGAGCTAT

2RC GGATCTGACCACATGAGATCTAAATGGTATTAACAGAGAATATGTGTCAAATCGAGCTAT

10 GGATCTGACCACATGAGATCTAAATGGTATTAACAGAGAATATGTGTCAAATCGAGCTAT

17 GGATCTGACCACATGAGATCTAAATGGTATTAACAGAGAATATGTGTCAAATCGAGCTAT

14 GGATCTGACCACATGAGATCTAAATGGTATTAACAGAGAATATGTGTCAAATCGAGCTAT

13 GGATCTGACCACATGAGATCTAAATGGTATTAACAGAGAATATGTGTCAAATCGAGCTAT

12 GGATCTGACCACATGAGATCTAAATGGTATTAACAGAGAATATGTGTCAAATCGAGCTAT

8 GGATCTGACCACATGAGATCTAAATGGTATTAACAGAGAATATGTGTCAAATCGAGCTAT

7 GGATCTGACCACATGAGATCTAAATGGTATTAACAGAGAATATGTGTCAAATCGAGCTAT

19 GGATCTGACCACATGAGATCTAAATGGTATTAACAGAGAATATGTGTCAAATCGAGCTAT

18 GGATCTGACCACATGAGATCTAAATGGTATTAACAGAGAATATGTGTCAAATCGAGCTAT

22 GGATCTGACCACATGAGATCTAAATGGTATTAACAGAGAATATGTGTCAAATCGAGCTAT

21 GGATCTGACCACATGAGATCTAAATGGTATTAACAGAGAATATGTGTCAAATCGAGCTAT

9 GGATCTGACCACATGAGATCTAAATGGTATTAACAGAGAATATGTGTCAAATCGAGCTAT

6 GGATCTGACCACATGAGATCTAAATGGTATTAACAGAGAATATGTGTCAAATCGAGCTAT

5 GGATCTGACCACATGAGATCTAAATGGTATTAACAGAGAATATGTGTCAAATCGAGCTAT

4 GGATCTGACCACATGAGATCTAAATGGTATTAACAGAGAATATGTGTCAAATCGAGCTAT

3 GGATCTGACCACATGAGATCTAAATGGTATTAACAGAGAATATGTGTCAAATCGAGCTAT

20 GGATCTGACCACATGAGATCTAAATGGTATTAACAGAGAATATGTGTCAAATCGAGCTAT

************************************************************

**<<<<<<<<<<PPE1**

1RC TCACCACAGAAAGGCCCATCATGGGGTATTTCTTGCGCTCTTGCTGATTTATTCTGAGCG

16RC TCACCACAGAAAGGCCCATCATGGGGTATTTCTTGCGCTCTTGCTGATTTATTCTGAGCG

15RC TCACCACAGAAAGGCCCATCATGGGGTATTTCTTGCGCTCTTGCTGATTTATTCTGAGCG

2RC TCACCACAGAAAGGCCCATCATGGGGTATTTCTTGCGCTCTTGCTGATTTATTCTGAGCG

10 TCACCACAGAAAGGCCCATCATGGGGTATTTCTTGCGCTCTTGCTGATTTATTCTGAGCG

17 TCACCACAGAAAGGCCCATCATGGGGTATTTCTTGCGCTCTTGCTGATTTATTCTGAGCG

14 TCACCACAGAAAGGCCCATCATGGGGTATTTCTTGCGCTCTTGCTGATTTATTCTGAGCG

13 TCACCACAGAAAGGCCCATCATGGGGTATTTCTTGCGCTCTTGCTGATTTATTCTGAGCG

12 TCACCACAGAAAGGCCCATCATGGGGTATTTCTTGCGCTCTTGCTGATTTATTCTGAGCG

8 TCACCACAGAAAGGCCCATCATGGGGTATTTCTTGCGCTCTTGCTGATTTATTCTGAGCG

7 TCACCACAGAAAGGCCCATCATGGGGTATTTCTTGCGCTCTTGCTGATTTATTCTGAGCG

19 TCACCACAGAAAGGCCCATCATGGGGTATTTCTTGCGCTCTTGCTGATTTATTCTGAGCG

18 TCACCACAGAAAGGCCCATCATGGGGTATTTCTTGCGCTCTTGCTGATTTATTCTGAGCG

22 TCACCACAGAAAGGCCCATCATGGGGTATTTCTTGCGCTCTTGCTGATTTATTCTGAGCG

21 TCACCACAGAAAGGCCCATCATGGGGTATTTCTTGCGCTCTTGCTGATTTATTCTGAGCG

9 TCACCACAGAAAGGCCCATCATGGGGTATTTCTTGCGCTCTTGCTGATTTATTCTGAGCG

6 TCACCACAGAAAGGCCCATCATGGGGTATTTCTTGCGCTCTTGCTGATTTATTCTGAGCG

5 TCACCACAGAAAGGCCCATCATGGGGTATTTCTTGCGCTCTTGCTGATTTATTCTGAGCG

4 TCACCACAGAAAGGCCCATCATGGGGTATTTCTTGCGCTCTTGCTGATTTATTCTGAGCG

3 TCACCACAGAAAGGCCCATCATGGGGTATTTCTTGCGCTCTTGCTGATTTATTCTGAGCG

20 TCACCACAGAAAGGCCCATCATGGGGTATTTCTTGCGCTCTTGCTGATTTATTCTGAGCG

************************************************************

1RC GTAGAATGATTAAAATAGTATGAAGCTTACCGATCACTCTGATTTGATCTAATGTCCGCA

16RC GTAGAATGATTAAAATAGTATGAAGCTTACCGATCACTCTGATTTGATCTAATGTCCGCA

15RC GTAGAATGATTAAAATAGTATGAAGCTTACCGATCACTCTGATTTGATCTAATGTCCGCA

2RC GTAGAATGATTAAAATAGTATGAAGCTTACCGATCACTCTGATTTGATCTAATGTCCGCA

10 GTAGAATGATTAAAATAGTATGAAGCTTACCGATCACTCTGATTTGATCTAATGTCCGCA

17 GTAGAATGATTAAAATAGTATGAAGCTTACCGATCACTCTGATTTGATCTAATGTCCGCA

14 GTAGAATGATTAAAATAGTATGAAGCTTACCGATCACTCTGATTTGATCTAATGTCCGCA

13 GTAGAATGATTAAAATAGTATGAAGCTTACCGATCACTCTGATTTGATCTAATGTCCGCA

12 GTAGAATGATTAAAATAGTATGAAGCTTACCGATCACTCTGATTTGATCTAATGTCCGCA

8 GTAGAATGATTAAAATAGTATGAAGCTTACCGATCACTCTGATTTGATCTAATGTCCGCA

7 GTAGAATGATTAAAATAGTATGAAGCTTACCGATCACTCTGATTTGATCTAATGTCCGCA

19 GTAGAATGATTAAAATAGTATGAAGCTTACCGATCACTCTGATTTGATCTAATGTCCGCA

18 GTAGAATGATTAAAATAGTATGAAGCTTACCGATCACTCTGATTTGATCTAATGTCCGCA

22 GTAGAATGATTAAAATAGTATGAAGCTTACCGATCACTCTGATTTGATCTAATGTCCGCA

21 GTAGAATGATTAAAATAGTATGAAGCTTACCGATCACTCTGATTTGATCTAATGTCCGCA

9 GTAGAATGATTAAAATAGTATGAAGCTTACCGATCACTCTGATTTGATCTAATGTCCGCA

6 GTAGAATGATTAAAATAGTATGAAGCTTACCGATCACTCTGATTTGATCTAATGTCCGCA

5 GTAGAATGATTAAAATAGTATGAAGCTTACCGATCACTCTGATTTGATCTAATGTCCGCA

4 GTAGAATGATTAAAATAGTATGAAGCTTACCGATCACTCTGATTTGATCTAATGTCCGCA

3 GTAGAATGATTAAAATAGTATGAAGCTTACCGATCACTCTGATTTGATCTAATGTCCGCA

20 GTAGAATGATTAAAATAGTATGAAGCTTACCGATCACTCTGATTTGATCTAATGTCCGCA

************************************************************

1RC CGTCTGGCGACAAACCACCATGAACACAGAGTATCCTACCATCAATGATCTGAAACCTTT

16RC CGTCTGGCGACAAACCACCATGAACACAGAGTATCCTACCATCAATGATCTGAAACCTTT

15RC CGTCTGGCGACAAACCACCATGAACACAGAGTATCCTACCATCAATGATCTGAAACCTTT

2RC CGTCTGGCGACAAACCACCATGAACACAGAGTATCCTACCATCAATGATCTGAAACCTTT

10 CGTCTGGCGACAAACCACCATGAACACAGAGTATCCTACCATCAATGATCTGAAACCTTT

17 CGTCTGGCGACAAACCACCATGAACACAGAGTATCCTACCATCAATGATCTGAAACCTTT

14 CGTCTGGCGACAAACCACCATGAACACAGAGTATCCTACCATCAATGATCTGAAACCTTT

13 CGTCTGGCGACAAACCACCATGAACACAGAGTATCCTACCATCAATGATCTGAAACCTTT

12 CGTCTGGCGACAAACCACCATGAACACAGAGTATCCTACCATCAATGATCTGAAACCTTT

8 CGTCTGGCGACAAACCACCATGAACACAGAGTATCCTACCATCAATGATCTGAAACCTTT

7 CGTCTGGCGACAAACCACCATGAACACAGAGTATCCTACCATCAATGATCTGAAACCTTT

19 CGTCTGGCGACAAACCACCATGAACACAGAGTATCCTACCATCAATGATCTGAAACCTTT

18 CGTCTGGCGACAAACCACCATGAACACAGAGTATCCTACCATCAATGATCTGAAACCTTT

22 CGTCTGGCGACAAACCACCATGAACACAGAGTATCCTACCATCAATGATCTGAAACCTTT

21 CGTCTGGCGACAAACCACCATGAACACAGAGTATCCTACCATCAATGATCTGAAACCTTT

9 CGTCTGGCGACAAACCACCATGAACACAGAGTATCCTACCATCAATGATCTGAAACCTTT

6 CGTCTGGCGACAAACCACCATGAACACAGAGTATCCTACCATCAATGATCTGAAACCTTT

5 CGTCTGGCGACAAACCACCATGAACACAGAGTATCCTACCATCAATGATCTGAAACCTTT

4 CGTCTGGCGACAAACCACCATGAACACAGAGTATCCTACCATCAATGATCTGAAACCTTT

3 CGTCTGGCGACAAACCACCATGAACACAGAGTATCCTACCATCAATGATCTGAAACCTTT

20 CGTCTGGCGACAAACCACCATGAACACAGAGTATCCTACCATCAATGATCTGAAACCTTT

************************************************************

1RC AGCTGTGTTTCAAATTGGTTTGCGACTCACGGCAGCTAAATTGAGATAGTCAAATACTTG

16RC AGCTGTGTTTCAAATTGGTTTGCGACTCACGGCAGCTAAATTGAGATAGTCAAATACTTG

15RC AGCTGTGTTTCAAATTGGTTTGCGACTCACGGCAGCTAAATTGAGATAGTCAAATACTTG

2RC AGCTGTGTTTCAAATTGGTTTGCGACTCACGGCAGCTAAATTGAGATAGTCAAATACTTG

10 AGCTGTGTTTCAAATTGGTTTGCGACTCACAGCAGCTAAATTGAGATAGTCAAATACTTG

17 AGCTGTGTTTCAAATTGGTTTGCGACTCACAGCAGCTAAATTGAGATAGTCAAATACTTG

14 AGCTGTGTTTCAAATTGGTTTGCGACTCACAGCAGCTAAATTGAGATAGTCAAATACTTG

13 AGCTGTGTTTCAAATTGGTTTGCGACTCACAGCAGCTAAATTGAGATAGTCAAATACTTG

12 AGCTGTGTTTCAAATTGGTTTGCGACTCACAGCAGCTAAATTGAGATAGTCAAATACTTG

8 AGCTGTGTTTCAAATTGGTTTGCGACTCACAGCAGCTAAATTGAGATAGTCAAATACTTG

7 AGCTGTGTTTCAAATTGGTTTGCGACTCACAGCAGCTAAATTGAGATAGTCAAATACTTG

19 AGCTGTGTTTCAAATTGGTTTGCGACTCACAGCAGCTAAATTGAGATAGTCAAATACTTG

18 AGCTGTGTTTCAAATTGGTTTGCGACTCACAGCAGCTAAATTGAGATAGTCAAATACTTG

22 AGCTGTGTTTCAAATTGGTTTGCGACTCACAGCAGCTAAATTGAGATAGTCAAATACTTG

21 AGCTGTGTTTCAAATTGGTTTGCGACTCACAGCAGCTAAATTGAGATAGTCAAATACTTG

9 AGCTGTGTTTCAAATTGGTTTGCGACTCACAGCAGCTAAATTGAGATAGTCAAATACTTG

6 AGCTGTGTTTCAAATTGGTTTGCGACTCACAGCAGCTAAATTGAGATAGTCAAATACTTG

5 AGCTGTGTTTCAAATTGGTTTGCGACTCACAGCAGCTAAATTGAGATAGTCAAATACTTG

4 AGCTGTGTTTCAAATTGGTTTGCGACTCACAGCAGCTAAATTGAGATAGTCAAATACTTG

3 AGCTGTGTTTCAAATTGGTTTGCGACTCACAGCAGCTAAATTGAGATAGTCAAATACTTG

20 AGCTGTGTTTCAAATTGGTTTGCGACTCACAGCAGCTAAATTGAGATAGTCAAATACTTG

******************************.*****************************

1RC ACAACATGCTTTCCAGACGCTGGCGTTTCCATATTTCTGTTGACACTCGTCTTATACTTT

16RC ACAACATGCTTTCCAGACGCTGGCGTTTCCATATTTCTGTTGACACTCGTCTTATACTTT

15RC ACAACATGCTTTCCAGACGCTGGCGTTTCCATATTTCTGTTGACACTCGTCTTATACTTT

2RC ACAACATGCTTTCCAGACGCTGGCGTTTCCATATTTCTGTTGACACTCGTCTTATACTTT

10 ACAACATGCTTTCCAGACGCTGGCGTTTCCATATTTCTGTTGACACTCGTCTTATACTTT

17 ACAACATGCTTTCCAGACGCTGGCGTTTCCATATTTCTGTTGACACTCGTCTTATACTTT

14 ACAACATGCTTTCCAGACGCTGGCGTTTCCATATTTCTGTTGACACTCGTCTTATACTTT

13 ACAACATGCTTTCCAGACGCTGGCGTTTCCATATTTCTGTTGACACTCGTCTTATACTTT

12 ACAACATGCTTTCCAGACGCTGGCGTTTCCATATTTCTGTTGACACTCGTCTTATACTTT

8 ACAACATGCTTTCCAGACGCTGGCGTTTCCATATTTCTGTTGACACTCGTCTTATACTTT

7 ACAACATGCTTTCCAGACGCTGGCGTTTCCATATTTCTGTTGACACTCGTCTTATACTTT

19 ACAACATGCTTTCCAGACGCTGGCGTTTCCATATTTCTGTTGACACTCGTCTTATACTTT

18 ACAACATGCTTTCCAGACGCTGGCGTTTCCATATTTCTGTTGACACTCGTCTTATACTTT

22 ACAACATGCTTTCCAGACGCTGGCGTTTCCATATTTCTGTTGACACTCGTCTTATACTTT

21 ACAACATGCTTTCCAGACGCTGGCGTTTCCATATTTCTGTTGACACTCGTCTTATACTTT

9 ACAACATGCTTTCCAGACGCTGGCGTTTCCATATTTCTGTTGACACTCGTCTTATACTTT

6 ACAACATGCTTTCCAGACGCTGGCGTTTCCATATTTCTGTTGACACTCGTCTTATACTTT

5 ACAACATGCTTTCCAGACGCTGGCGTTTCCATATTTCTGTTGACACTCGTCTTATACTTT

4 ACAACATGCTTTCCAGACGCTGGCGTTTCCATATTTCTGTTGACACTCGTCTTATACTTT

3 ACAACATGCTTTCCAGACGCTGGCGTTTCCATATTTCTGTTGACACTCGTCTTATACTTT

20 ACAACATGCTTTCCAGACGCTGGCGTTTCCATATTTCTGTTGACACTCGTCTTATACTTT

************************************************************

1RC CGATGAGTGACGAAAGACCGAATGAGCAAGGCAGCTTACCATAGAATCCATATACTTGTG

16RC CGATGAGTGACGAAAGACCGAATGAGCAAGGCAGCTTACCATAGAATCCATATACTTGTG

15RC CGATGAGTGACGAAAGACCGAATGAGCAAGGCAGCTTACCATAGAATCCATATACTTGTG

2RC CGATGAGTGACGAAAGACCGAATGAGCAAGGCAGCTTACCATAGAATCCATATACTTGTG

10 CGATGAGTGACGAAAGACCGAATGAGCAAGGCAGCTTACCATAGAATCCATATACTTGTG

17 CGATGAGTGACGAAAGACCGAATGAGCAAGGCAGCTTACCATAGAATCCATATACTTGTG

14 CGATGAGTGACGAAAGACCGAATGAGCAAGGCAGCTTACCATAGAATCCATATACTTGTG

13 CGATGAGTGACGAAAGACCGAATGAGCAAGGCAGCTTACCATAGAATCCATATACTTGTG

12 CGATGAGTGACGAAAGACCGAATGAGCAAGGCAGCTTACCATAGAATCCATATACTTGTG

8 CGATGAGTGACGAAAGACCGAATGAGCAAGGCAGCTTACCATAGAATCCATATACTTGTG

7 CGATGAGTGACGAAAGACCGAATGAGCAAGGCAGCTTACCATAGAATCCATATACTTGTG

19 CGATGAGTGACGAAAGACCGAATGAGCAAGGCAGCTTACCATAGAATCCATATACTTGTG

18 CGATGAGTGACGAAAGACCGAATGAGCAAGGCAGCTTACCATAGAATCCATATACTTGTG

22 CGATGAGTGACGAAAGACCGAATGAGCAAGGCAGCTTACCATAGAATCCATATACTTGTG

21 CGATGAGTGACGAAAGACCGAATGAGCAAGGCAGCTTACCATAGAATCCATATACTTGTG

9 CGATGAGTGACGAAAGACCGAATGAGCAAGGCAGCTTACCATAGAATCCATATACTTGTG

6 CGATGAGTGACGAAAGACCGAATGAGCAAGGCAGCTTACCATAGAATCCATATACTTGTG

5 CGATGAGTGACGAAAGACCGAATGAGCAAGGCAGCTTACCATAGAATCCATATACTTGTG

4 CGATGAGTGACGAAAGACCGAATGAGCAAGGCAGCTTACCATAGAATCCATATACTTGTG

3 CGATGAGTGACGAAAGACCGAATGAGCAAGGCAGCTTACCATAGAATCCATATACTTGTG

20 CGATGAGTGACGAAAGACCGAATGAGCAAGGCAGCTTACCATAGAATCCATATACTTGTG

************************************************************

1RC TGATTTGCCTAGATTCATGATTACCGCGCAAAAGGGTTATTCTATCTGGGTACCTAATTG

16RC TGATTTGCCTAGATTCATGATTACCGCGCAAAAGGGTTATTCTATCTGGGTACCTAATTG

15RC TGATTTGCCTAGATTCATGATTACCGCGCAAAAGGGTTATTCTATCTGGGTACCTAATTG

2RC TGATTTGCCTAGATTCATGATTACCGCGCAAAAGGGTTATTCTATCTGGGTACCTAATTG

10 TGATTTGCCTAGATTCATGATTACCGCGCAAAAGGGTTATTCTATCTGGGTACCTAATTG

17 TGATTTGCCTAGATTCATGATTACCGCGCAAAAGGGTTATTCTATCTGGGTACCTAATTG

14 TGATTTGCCTAGATTCATGATTACCGCGCAAAAGGGTTATTCTATCTGGGTACCTAATTG

13 TGATTTGCCTAGATTCATGATTACCGCGCAAAAGGGTTATTCTATCTGGGTACCTAATTG

12 TGATTTGCCTAGATTCATGATTACCGCGCAAAAGGGTTATTCTATCTGGGTACCTAATTG

8 TGATTTGCCTAGATTCATGATTACCGCGCAAAAGGGTTATTCTATCTGGGTACCTAATTG

7 TGATTTGCCTAGATTCATGATTACCGCGCAAAAGGGTTATTCTATCTGGGTACCTAATTG

19 TGATTTGCCTAGATTCATGATTACCGCGCAAAAGGGTTATTCTATCTGGGTACCTAATTG

18 TGATTTGCCTAGATTCATGATTACCGCGCAAAAGGGTTATTCTATCTGGGTACCTAATTG

22 TGATTTGCCTAGATTCATGATTACCGCGCAAAAGGGTTATTCTATCTGGGTACCTAATTG

21 TGATTTGCCTAGATTCATGATTACCGCGCAAAAGGGTTATTCTATCTGGGTACCTAATTG

9 TGATTTGCCTAGATTCATGATTACCGCGCAAAAGGGTTATTCTATCTGGGTACCTAATTG

6 TGATTTGCCTAGATTCATGATTACCGCGCAAAAGGGTTATTCTATCTGGGTACCTAATTG

5 TGATTTGCCTAGATTCATGATTACCGCGCAAAAGGGTTATTCTATCTGGGTACCTAATTG

4 TGATTTGCCTAGATTCATGATTACCGCGCAAAAGGGTTATTCTATCTGGGTACCTAATTG

3 TGATTTGCCTAGATTCATGATTACCGCGCAAAAGGGTTATTCTATCTGGGTACCTAATTG

20 TGATTTGCCTAGATTCATGATTACCGCGCAAAAGGGTTATTCTATCTGGGTACCTAATTG

************************************************************

1RC ACTGTTAAATATGATTTCAAGTATAGAGTGACTAACCTCGCTTTCAACGCCATCAAGAGG

16RC ACTGTTAAATATGATTTCAAGTATAGAGTGACTAACCTCGCTTTCAACGCCATCAAGAGG

15RC ACTGTTAAATATGATTTCAAGTATAGAGTGACTAACCTCGCTTTCAACGCCATCAAGAGG

2RC ACTGTTAAATATGATTTCAAGTATAGAGTGACTAACCTCGCTTTCAACGCCATCAAGAGG

10 ACTGTTAAATATGATTTCAAGTATAGAGTGACTAACCTCGCTTTCAACGCCATCAAGAGG

17 ACTGTTAAATATGATTTCAAGTATAGAGTGACTAACCTCGCTTTCAACGCCATCAAGAGG

14 ACTGTTAAATATGATTTCAAGTATAGAGTGACTAACCTCGCTTTCAACGCCATCAAGAGG

13 ACTGTTAAATATGATTTCAAGTATAGAGTGACTAACCTCGCTTTCAACGCCATCAAGAGG

12 ACTGTTAAATATGATTTCAAGTATAGAGTGACTAACCTCGCTTTCAACGCCATCAAGAGG

8 ACTGTTAAATATGATTTCAAGTATAGAGTGACTAACCTCGCTTTCAACGCCATCAAGAGG

7 ACTGTTAAATATGATTTCAAGTATAGAGTGACTAACCTCGCTTTCAACGCCATCAAGAGG

19 ACTGTTAAATATGATTTCAAGTATAGAGTGACTAACCTCGCTTTCAACGCCATCAAGAGG

18 ACTGTTAAATATGATTTCAAGTATAGAGTGACTAACCTCGCTTTCAACGCCATCAAGAGG

22 ACTGTTAAATATGATTTCAAGTATAGAGTGACTAACCTCGCTTTCAACGCCATCAAGAGG

21 ACTGTTAAATATGATTTCAAGTATAGAGTGACTAACCTCGCTTTCAACGCCATCAAGAGG

9 ACTGTTAAATATGATTTCAAGTATAGAGTGACTAACCTCGCTTTCAACGCCATCAAGAGG

6 ACTGTTAAATATGATTTCAAGTATAGAGTGACTAACCTCGCTTTCAACGCCATCAAGAGG

5 ACTGTTAAATATGATTTCAAGTATAGAGTGACTAACCTCGCTTTCAACGCCATCAAGAGG

4 ACTGTTAAATATGATTTCAAGTATAGAGTGACTAACCTCGCTTTCAACGCCATCAAGAGG

3 ACTGTTAAATATGATTTCAAGTATAGAGTGACTAACCTCGCTTTCAACGCCATCAAGAGG

20 ACTGTTAAATATGATTTCAAGTATAGAGTGACTAACCTCGCTTTCAACGCCATCAAGAGG

************************************************************

1RC GTGAAACTTTCCAGGCTTTGATGACCTCTGTCAACGAAGTCGCCCTGTTTATGCTCAGCA

16RC GTGAAACTTTCCAGGCTTTGATGACCTCTGTCAACGAAGTCGCCCTGTTTATGCTCAGCA

15RC GTGAAACTTTCCAGGCTTTGATGACCTCTGTCAACGAAGTCGCCCTGTTTATGCTCAGCA

2RC GTGAAACTTTCCAGGCTTTGATGACCTCTGTCAACGAAGTCGCCCTGTTTATGCTCAGCA

10 GTGAAACTTTCCAGGCTTTGATGACCTCTGTCAACGAAGTCGCCCTGTTTATGCTCAGCA

17 GTGAAACTTTCCAGGCTTTGATGACCTCTGTCAACGAAGTCGCCCTGTTTATGCTCAGCA

14 GTGAAACTTTCCAGGCTTTGATGACCTCTGTCAACGAAGTCGCCCTGTTTATGCTCAGCA

13 GTGAAACTTTCCAGGCTTTGATGACCTCTGTCAACGAAGTCGCCCTGTTTATGCTCAGCA

12 GTGAAACTTTCCAGGCTTTGATGACCTCTGTCAACGAAGTCGCCCTGTTTATGCTCAGCA

8 GTGAAACTTTCCAGGCTTTGATGACCTCTGTCAACGAAGTCGCCCTGTTTATGCTCAGCA

7 GTGAAACTTTCCAGGCTTTGATGACCTCTGTCAACGAAGTCGCCCTGTTTATGCTCAGCA

19 GTAAAACTTTCCAGGCTTTGATGACCTCTGTCAACGAAGTCGCCCTGTTTATGCTCAGCA

18 GTAAAACTTTCCAGGCTTTGATGACCTCTGTCAACGAAGTCGCCCTGTTTATGCTCAGCA

22 GTGAAACTTTCCAGGCTTTGATGACCTCTGTCAACGAAGTCGCCCTGTTTATGCTCAGCA

21 GTGAAACTTTCCAGGCTTTGATGACCTCTGTCAACGAAGTCGCCCTGTTTATGCTCAGCA

9 GTGAAACTTTCCAGGCTTTGATGACCTCTGTCAACGAAGTCGCCCTGTTTATGCTCAGCA

6 GTGAAACTTTCCAGGCTTTGATGACCTCTGTCAACGAAGTCGCCCTGTTTATGCTCAGCA

5 GTGAAACTTTCCAGGCTTTGATGACCTCTGTCAACGAAGTCGCCCTGTTTATGCTCAGCA

4 GTGAAACTTTCCAGGCTTTGATGACCTCTGTCAACGAAGTCGCCCTGTTTATGCTCAGCA

3 GTGAAACTTTCCAGGCTTTGATGACCTCTGTCAACGAAGTCGCCCTGTTTATGCTCAGCA

20 GTGAAACTTTCCAGGCTTTGATGACCTCTGTCAACGAAGTCGCCCTGTTTATGCTCAGCA

**.*********************************************************

1RC CCGAGAAATGCAACAACTGAGCGCCTTACCATGAAGATATAGTTAGTATCCCTGCAATCA

16RC CCGAGAAATGCAACAACTGAGCGCCTTACCATGAAGATATAGTTAGTATCCCTGCAATCA

15RC CCGAGAAATGCAACAACTGAGCGCCTTACCATGAAGATATAGTTAGTATCCCTGCAATCA

2RC CCGAGAAATGCAACAACTGAGCGCCTTACCATGAAGATATAGTTAGTATCCCTGCAATCA

10 CCGAGAAATGCAACAACTGAGCGCCTTACCATGAAGATATAGTTGGTATCCCTGCAATCA

17 CCGAGAAATGCAACAACTGAGCGCCTTACCATGAAGATATAGTTGGTATCCCTGCAATCA

14 CCGAGAAATGCAACAACTGAGCGCCTTACCATGAAGATATAGTTGGTATCCCTGCAATCA

13 CCGAGAAATGCAACAACTGAGCGCCTTACCATGAAGATATAGTTGGTATCCCTGCAATCA

12 CCGAGAAATGCAACAACTGAGCGCCTTACCATGAAGATATAGTTGGTATCCCTGCAATCA

8 CCGAGAAATGCAACAACTGAGCGCCTTACCATGAAGATATAGTTGGTATCCCTGCAATCA

7 CCGAGAAATGCAACAACTGAGCGCCTTACCATGAAGATATAGTTGGTATCCCTGCAATCA

19 CCGAGAAATGCAACAACTGAGCGCCTTACCATGAAGATATAGTTGGTATCCCTGCAATCA

18 CCGAGAAATGCAACAACTGAGCGCCTTACCATGAAGATATAGTTGGTATCCCTGCAATCA

22 CCGAGAAATGC-ATAACTGAGCGCCTTACCATGAAGATATAGTTGGTATCCCTGCAATCA

21 CCGAGAAATGCAACAACTGAGCGCCTTACCATGAAGATATAGTTGGTATCCCTGCAATCA

9 CCGAGAAATGCAACAACTGAGCGCCTTACCATGAAGATATAGTTGGTATCCCTGCAATCA

6 CCGAGAAATGCAACAACTGAGCGCCTTACCATGAAGATATAGTTGGTATCCCTGCAATCA

5 CCGAGAAATGCAACAACTGAGCGCCTTACCATGAAGATATAGTTGGTATCCCTGCAATCA

4 CCGAGAAATGCAACAACTGAGCGCCTTACCATGAAGATATAGTTGGTATCCCTGCAATCA

3 CCGAGAAATGCAACAACTGAGCGCCTTACCATGAAGATATAGTTGGTATCCCTGCAATCA

20 CCGAGAAATGCAACAACTGAGCGCCTTACCATGAAGATATAGTTGGTATCCCTGCAATCA

*********** *.******************************.***************

1RC CCGCCGACCCGCAAAAGCTCAAGTAAATCCCAAAATTGACCATGTATATCTCCACAGACA

16RC CCGCCGACCCGCAAAAGCTCAAGTAAATCCCAAAATTGACCATGTATATCTCCACAGACA

15RC CCGCCGACCCGCAAAAGCTCAAGTAAATCCCAAAATTGACCATGTATATCTCCACAGACA

2RC CCGCCGACCCGCAAAAGCTCAAGTAAATCCCAAAATTGACCATGTATATCTCCACAGACA

10 CCGCCGACCCGCAAAAGCTCAAGTAAATCCCAAAATTGACCATGTATATCTCCACAGACA

17 CCGCCGACCCGCAAAAGCTCAAGTAAATCCCAAAATTGACCATGTATATCTCCACAGACA

14 CCGCCGACCCGCAAAAGCTCAAGTAAATCCCAAAATTGACCATGTATATCTCCACAGACA

13 CCGCCGACCCGCAAAAGCTCAAGTAAATCCCAAAATTGACCATGTATATCTCCACAGACA

12 CCGCCGACCCGCAAAAGCTCAAGTAAATCCCAAAATTGACCATGTATATCTCCACAGACA

8 CCGCCGACCCGCAAAAGCTCAAGTAAATCCCAAAATTGACCATGTATATCTCCACAGACA

7 CCGCCGACCCGCAAAAGCTCAAGTAAATCCCAAAATTGACCATGTATATCTCCACAGACA

19 CCGCCGACCCGCAAAAGCTCAAGTAAATCCCAAAATTGACCATGTATATCTCCACAGACA

18 CCGCCGACCCGCAAAAGCTCAAGTAAATCCCAAAATTGACCATGTATATCTCCACAGACA

22 CCGCCGACCCGCAAAAGCTCAAGTAAATCCCAAAATTGACCATGTATATCTCCACAGACA

21 CCGCCGACCCGCAAAAGCTCAAGTAAATCCCAAAATTGACCATGTATATCTCCACAGACA

9 CCGCCGACCCGCAAAAGCTCAAGTAAATCCCAAAATTGACCATGTATATCTCCACAGACA

6 CCGCCGACCCGCAAAAGCTCAAGTAAATCCCAAAATTGACCATGTATATCTCCACAGACA

5 CCGCCGACCCGCAAAAGCTCAAGTAAATCCCAAAATTGACCATGTATATCTCCACAGACA

4 CCGCCGACCCGCAAAAGCTCAAGTAAATCCCAAAATTGACCATGTATATCTCCACAGACA

3 CCGCCGACCCGCAAAAGCTCAAGTAAATCCCAAAATTGACCATGTATATCTCCACAGACA

20 CCGCCGACCCGCAAAAGCTCAAGTAAATCCCAAAATTGACCATGTATATCTCCACAGACA

************************************************************

1RC GTGACAGGACTCGAGACTGGTTGTATGTTTGACTCCTCTATTAGCAAACCCAGTACTACG

16RC GTGACAGGACTCGAGACTGGTTGTATGTTTGACTCCTCTATTAGCAAACCCAGTACTACG

15RC GTGACAGGACTCGAGACTGGTTGTATGTTTGACTCCTCTATTAGCAAACCCAGTACTACG

2RC GTGACAGGACTCGAGACTGGTTGTATGTTTGACTCCTCTATTAGCAAACCCAGTACTACG

10 GTGACAGGACTCGAGACTGGTTGTATGTTTGACTCCTCTATTAGCAAACCCAGTACTACG

17 GTGACAGGACTGGAGACTGGTTGTATGTTTGACTCCTCTATTAGCAAACCCAGTACTACG

14 GTGACAGGACTGGAGACTGGTTGTATGTTTGACTCCTCTATTAGCAAACCCAGTACTACG

13 GTGACAGGACTGGAGACTGGTTGTATGTTTGACTCCTCTATTAGCAAACCCAGTACTACG

12 GTGACAGGACTGGAGACTGGTTGTATGTTTGACTCCTCTATTAGCAAACCCAGTACTACG

8 GTGACAGGACTGGAGACTGGTTGTATGTTTGACTCCTCTATTAGCAAACCCAGTACTACG

7 GTGACAGGACTGGAGACTGGTTGTATGTTTGACTCCTCTATTAGCAAACCCAGTACTACG

19 GTGACAGGACTGGAGACTGGTTGTATGTTTGACTCCTCTATTAGCAAACCCAGTACTACG

18 GTGACAGGACTGGAGACTGGTTGTATGTTTGACTCCTCTATTAGCAAACCCAGTACTACG

22 GTGACAGGACTCGAGACTGGTTGTATGTTTGACTCCTCTATTAGCAAACCCAGTACTACG

21 GTGACAGGACTCGAGACTGGTTGTATGTTTGACTCCTCTATTAGCAAACCCAGTACTACG

9 GTGACAGGACTCGAGACTGGTTGTATGTTTGACTCCTCTATTAGCAAACCCAGTACTACG

6 GTGACAGGACTCGAGACTGGTTGTATGTTTGACTCCTCTATTAGCAAACCCAGTACTACG

5 GTGACAGGACTCGAGACTGGTTGTATGTTTGACTCCTCTATTAGCAAACCCAGTACTACG

4 GTGACAGGACTCGAGACTGGTTGTATGTTTGACTCCTCTATTAGCAAACCCAGTACTACG

3 GTGACAGGACTCGAGACTGGTTGTATGTTTGACTCCTCTATTAGCAAACCCAGTACTACG

20 GTGACAGGACTCGAGACTGGTTGTATGTTTGACTCCTCTATTAGCAAACCCAGTACTACG

*********** ************************************************

1RC TCGCACAAGATCTTGATTTGAGATTCAGCGAGATAGTTTCCAGATAGTATTGTATCGAGC

16RC TCGCACAAGATCTTGATTTGAGATTCAGCGAGATAGTTTCCAGATAGTATTGTATCGAGC

15RC TCGCACAAGATCTTGATTTGAGATTCAGCGAGATAGTTTCCAGATAGTATTGTATCGAGC

2RC TCGCACAAGATCTTGATTTGAGATTCAGCGAGATAGTTTCCAGATAGTATTGTATCGAGC

10 TCGCACAAGATCTTGATTTGAGATTCAGCGAGATAGTTTCCAGATAGTATTGTATCGAGC

17 TCGCACAAGATCTTGATTTGAGATTCAGCGAGATAGTTTCCAGATAGTATTGTATCGAGC

14 TCGCACAAGATCTTGATTTGAGATTCAGCGAGATAGTTTCCAGATAGTATTGTATCGAGC

13 TCGCACAAGATCTTGATTTGAGATTCAGCGAGATAGTTTCCAGATAGTATTGTATCGAGC

12 TCGCACAAGATCTTGATTTGAGATTCAGCGAGATAGTTTCCAGATAGTATTGTATCGAGC

8 TCGCACAAGATCTTGATTTGAGATTCAGCGAGATAGTTTCCAGATAGTATTGTATCGAGC

7 TCGCACAAGATCTTGATTTGAGATTCAGCGAGATAGTTTCCAGATAGTATTGTATCGAGC

19 TCGCACAAGATCTTGATTTGAGATTCAGCGAGATAGTTTCCAGATAGTATTGTATCGAGC

18 TCGCACAAGATCTTGATTTGAGATTCAGCGAGATAGTTTCCAGATAGTATTGTATCGAGC

22 TCGCACAAGATCTTGATTTGAGATTCAGCGAGATAGTTTCCAGATAGTATTGTATCGAGC

21 TCGCACAAGATCTTGATTTGAGATTCAGCGAGATAGTTTCCAGATAGTATTGTATCGAGC

9 TCGCACAAGATCTTGATTTGAGATTCAGCGAGATAGTTTCCAGATAGTATTGTATCGAGC

6 TCGCACAAGATCTTGATTTGAGATTCAGCGAGATAGTTTCCAGATAGTATTGTATCGAGC

5 TCGCACAAGATCTTGATTTGAGATTCAGCGAGATAGTTTCCAGATAGTATTGTATCGAGC

4 TCGCACAAGATCTTGATTTGAGATTCAGCGAGATAGTTTCCAGATAGTATTGTATCGAGC

3 TCGCACAAGATCTTGATTTGAGATTCAGCGAGATAGTTTCCAGATAGTATTGTATCGAGC

20 TCGCACAAGATCTTGATTTGAGATTCAGCGAGATAGTTTCCAGATAGTATTGTATCGAGC

************************************************************

1RC CATTGTTGGGGATCTACTGTCATTATCCGTGCTAGTATCTACGCGGTATATTCTACAAAA

16RC CATTGTTGGGGATCTACTGTCATTATCCGTGCTAGTATCTACGCGGTATATTCTACAAAA

15RC CATTGTTGGGGATCTACTGTCATTATCCGTGCTAGTATCTACGCGGTATATTCTACAAAA

2RC CATTGTTGGGGATCTACTGTCATTATCCGTGCTAGTATCTACGCGGTATATTCTACAAAA

10 CATTGTTGGGGATCTACTGTCATTATCCGTGCTAGTATCTACGCGGTATATTCTACAAAA

17 CATTGTTGGGGATCTACTGTCATTATCCGTGCTAGTATCTACGCGGTATATTCTACAAAA

14 CATTGTTGGGGATCTACTGTCATTATCCGTGCTAGTATCTACGCGGTATATTCTACAAAA

13 CATTGTTGGGGATCTACTGTCATTATCCGTGCTAGTATCTACGCGGTATATTCTACAAAA

12 CATTGTTGGGGATCTACTGTCATTATCCGTGCTAGTATCTACGCGGTATATTCTACAAAA

8 CATTGTTGGGGATCTACTGTCATTATCCGTGCTAGTATCTACGCGGTATATTCTACAAAA

7 CATTGTTGGGGATCTACTGTCATTATCCGTGCTAGTATCTACGCGGTATATTCTACAAAA

19 CATTGTTGGGGATCTACTGTCATTATCCGTGCTAGTATCTACGCGGTATATTCTACAAAA

18 CATTGTTGGGGATCTACTGTCATTATCCGTGCTAGTATCTACGCGGTATATTCTACAAAA

22 CATTGTTGGGGATCTACTGTCATTATCCGTGCTAGTATCTACGCGGTATATTCTACAAAA

21 CATTGTTGGGGATCTACTGTCATTATCCGTGCTAGTATCTACGCGGTATATTCTACAAAA

9 CATTGTTGGGGATCTACTGTCATTATCCGTGCTAGTATCTACGCGGTATATTCTACAAAA

6 CATTGTTGGGGATCTACTGTCATTATCCGTGCTAGTATCTACGCGGTATATTCTACAAAA

5 CATTGTTGGGGATCTACTGTCATTATCCGTGCTAGTATCTACGCGGTATATTCTACAAAA

4 CATTGTTGGGGATCTACTGTCATTATCCGTGCTAGTATCTACGCGGTATATTCTACAAAA

3 CATTGTTGGGGATCTACTGTCATTATCCGTGCTAGTATCTACGCGGTATATTCTACAAAA

20 CATTGTTGGGGATCTACTGTCATTATCCGTGCTAGTATCTACGCGGTATATTCTACAAAA

************************************************************

1RC GTAATTTGTGTTCGTCGTTTGCTCACAACTCAGTTGCCGAGTTACTGTCATGTCCGCAAA

16RC GTAATTTGTGTTCGTCGTTTGCTCACAACTCAGTTGCCGAGTTACTGTCATGTCCGCAAA

15RC GTAATTTGTGTTCGTCGTTTGCTCACAACTCAGTTGCCGAGTTACTGTCATGTCCGCAAA

2RC GTAATTTGTGTTCGTCGTTTGCTCACAACTCAGTTGCCGAGTTACTGTCATGTCCGCAAA

10 GTAATTTGTGTTCGTCGTTTGCTCACAACTCAGTTGCCGAGTTACTGTCATGTCCGCAAA

17 GTAATTTGTGTTCGTCGTTTGCTCACAACTCAGTTGCCGAGTTACTGTCATGTCCGCAAA

14 GTAATTTGTGTTCGTCGTTTGCTCACAACTCAGTTGCCGAGTTACTGTCATGTCCGCAAA

13 GTAATTTGTGTTCGTCGTTTGCTCACAACTCAGTTGCCGAGTTACTGTCATGTCCGCAAA

12 GTAATTTGTGTTCGTCGTTTGCTCACAACTCAGTTGCCGAGTTACTGTCATGTCCGCAAA

8 GTAATTTGTGTTCGTCGTTTGCTCACAACTCAGTTGCCGAGTTACTGTCATGTCCGCAAA

7 GTAATTTGTGTTCGTCGTTTGCTCACAACTCAGTTGCCGAGTTACTGTCATGTCCGCAAA

19 GTAATTTGTGTTCGTCGTTTGCTCACAACTCAGTTGCCGAGTTACTGTCATGTCCGCAAA

18 GTAATTTGTGTTCGTCGTTTGCTCACAACTCAGTTGCCGAGTTACTGTCATGTCCGCAAA

22 GTAATTTGTGTTCGTCGTTTGCTCACAACTCAGTTGCCGAGTTACTGTCATGTCCGCAAA

21 GTAATTTGTGTTCGTCGTTTGCTCACAACTCAGTTGCCGAGTTACTGTCATGTCCGCAAA

9 GTAATTTGTGTTCGTCGTTTGCTCACAACTCAGTTGCCGAGTTACTGTCATGTCCGCAAA

6 GTAATTTGTGTTCGTCGTTTGCTCACAACTCAGTTGCCGAGTTACTGTCATGTCCGCAAA

5 GTAATTTGTGTTCGTCGTTTGCTCACAACTCAGTTGCCGAGTTACTGTCATGTCCGCAAA

4 GTAATTTGTGTTCGTCGTTTGCTCACAACTCAGTTGCCGAGTTACTGTCATGTCCGCAAA

3 GTAATTTGTGTTCGTCGTTTGCTCACAACTCAGTTGCCGAGTTACTGTCATGTCCGCAAA

20 GTAATTTGTGTTCGTCGTTTGCTCACAACTCAGTTGCCGAGTTACTGTCATGTCCGCAAA

************************************************************

**PEMT>>>>>>>**

1RC GCAGCATCCATTTCTCAGAGGGCTTTCTGAGCTTCTGCCTTACCCATCTGCCCTGCAATC

16RC GCAGCATCCATTTCTCAGAGGGCTTTCTGAGCTTCTGCCTTACCCATCTGCCCTGCAATC

15RC GCAGCATCCATTTCTCAGAGGGCTTTCTGAGCTTCTGCCTTACCCATCTGCCCTGCAATC

2RC GCAGCATCCATTTCTCAGAGGGCTTTCTGAGCTTCTGCCTTACCCATCTGCCCTGCAATC

10 GCAGCATCCATTTCTCAGAGGGCTTTCTGAGCTTCTGCCTTACCCATCTGCCCTGCAATC

17 GCAGCATCCATTTCTCAGAGGGCTTTCTGAGCTTCTGCCTTACCCATCTGCCCTGCAATC

14 GCAGCATCCATTTCTCAGAGGGCTTTCTGAGCTTCTGCCTTACCCATCTGCCCTGCAATC

13 GCAGCATCCATTTCTCAGAGGGCTTTCTGAGCTTCTGCCTTACCCATCTGCCCTGCAATC

12 GCAGCATCCATTTCTCAGAGGGCTTTCTGAGCTTCTGCCTTACCCATCTGCCCTGCAATC

8 GCAGCATCCATTTCTCAGAGGGCTTTCTGAGCTTCTGCCTTACCCATCTGCCCTGCAATC

7 GCAGCATCCATTTCTCAGAGGGCTTTCTGAGCTTCTGCCTTACCCATCTGCCCTGCAATC

19 GCAGCATCCATTTCTCAGAGGGCTTTCTGAGCTTCTGCCTTACCCATCTGCCCTGCAATC

18 GCAGCATCCATTTCTCAGAGGGCTTTCTGAGCTTCTGCCTTACCCATCTGCCCTGCAATC

22 GCAGCATCCATTTCTCAGAGGGCTTTCTGAGCTTCTGCCTTACCCATCTGCCCTGCAATC

21 GCAGCATCCATTTCTCAGAGGGCTTTCTGAGCTTCTGCCTTACCCATCTGCCCTGCAATC

9 GCAGCATCCATTTCTCAGAGGGCTTTCTGAGCTTCTGCCTTACCCATCTGCCCTGCAATC

6 GCAGCATCCATTTCTCAGAGGGCTTTCTGAGCTTCTGCCTTACCCATCTGCCCTGCAATC

5 GCAGCATCCATTTCTCAGAGGGCTTTCTGAGCTTCTGCCTTACCCATCTGCCCTGCAATC

4 GCAGCATCCATTTCTCAGAGGGCTTTCTGAGCTTCTGCCTTACCCATCTGCCCTGCAATC
[truncated: 621,797 more chars]
